# Highly enantioselective sulfa-Michael addition reactions using *N*-heterocyclic carbene as a non-covalent organocatalyst†
†Electronic supplementary information (ESI) available. CCDC 1047559. For ESI and crystallographic data in CIF or other electronic format see DOI: 10.1039/c5sc00878f


**DOI:** 10.1039/c5sc00878f

**Published:** 2015-04-23

**Authors:** Jiean Chen, Sixuan Meng, Leming Wang, Hongmei Tang, Yong Huang

**Affiliations:** a Key Laboratory of Chemical Genomics , School of Chemical Biology and Biotechnology , Peking University , Shenzhen Graduate School , Shenzhen , 518055 , China . Email: huangyong@pkusz.edu.cn

## Abstract

We report the first asymmetric sulfa-Michael addition (SMA) reactions using a chiral *N*-heterocyclic carbene (NHC) as a non-covalent organocatalyst. We demonstrate that a triazolium salt derived NHC functions as an excellent Brønsted base to promote enantioselective carbon–sulfur bond formation. The reaction is applicable to a wide range of thiols and electrophilic olefins. Notably, quaternary chiral centers bearing both an S atom and a CF_3_ group were synthesized with excellent asymmetric control. Mechanistic studies suggest that the facial discrimination is likely to be guided by non-covalent interactions: hydrogen bonding and π–π stacking.

## Abstract 2

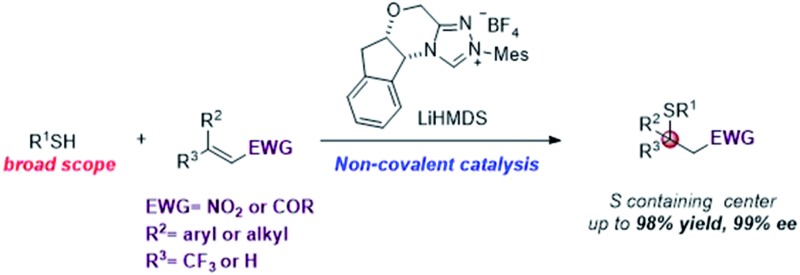
Enantioselective asymmetric sulfa-Michael addition (SMA) reactions using a chiral *N*-heterocyclic carbene as a non-covalent organocatalyst.

## Introduction

**Scheme 1:**
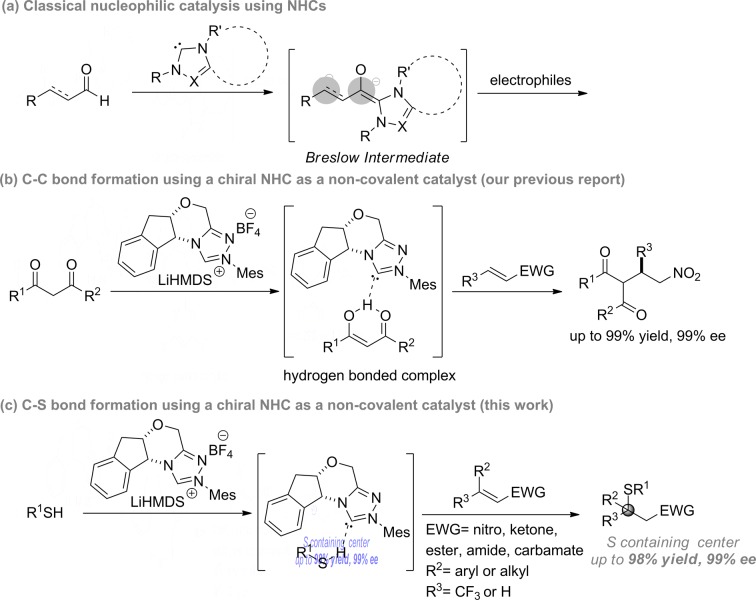
NHCs as non-covalent chiral catalysts for asymmetric reactions.

Chiral NHCs are arguably the most effective nucleophilic organocatalysts for asymmetric synthesis.^1^ Since the seminal proposal of the Breslow intermediate in the thiamine biocatalysis machinery,^2^ chiral NHCs have been extended to numerous enantioselective processes *via* Lewis base catalysis. Typically, substrate activation for these reactions is accomplished through a reversible carbon–carbon bond formation between the NHC catalyst and a carbonyl group, *e.g.* aldehyde, acyl halide/anhydride, ketene, activated ester, *etc.*^2^,^3^ This rapid equilibrium generates a chiral intermediate that can react with a wide spectrum of nucleophiles, electrophiles or dipolarophiles to form carbon–carbon and carbon–heteroatom bonds enantioselectively. In sharp contrast, asymmetric catalysis *via* non-covalent interactions is little known for NHCs, despite their strong intrinsic Brønsted basicity. There are ample racemic examples where NHCs might serve as a non-covalent Brønsted base catalyst.^4^ However, attempts to affect facial differentiation have been largely unsuccessful.4h,4i Recently, we reported the first enantioselective carbon–carbon forming reaction using NHCs as non-covalent organocatalysts (Scheme 1).^5^ However, asymmetric carbon–heteroatom bond formation remains elusive.

The asymmetric hetero-Michael reaction is an indispensable method for the synthesis of 1,2-difunctionalized two-carbon units that are privileged motifs in natural products and drug molecules.^6^ HOMO raising Brønsted base catalysis offers a very attractive strategy for 1,4-addition reactions of acidic heteroatom nucleophiles. So far, such activation has been predominantly limited to tertiary amines.6b,6d,6f,^7^ A distinct class of non-covalent catalysts, NHCs for example, would offer complementary reaction scopes and significantly broaden the capabilities of this general activation mode.

The asymmetric sulfa-Michael addition (SMA) reaction is among the most prevalent strategies to access sulfur containing chiral centers.^8^ SMA reactions have been extensively studied using either metal or amine catalysts.^9^ However, the substrate scope for these reactions is often very narrow, limited to special thiols and electron-deficient olefins. Reactions involving β,β-disubstituted olefins are particularly challenging. So far, NHCs have not been successfully used as chiral Brønsted base catalysts for SMA reactions.^10^ Herein, we extend the generic non-covalent activation of NHCs to asymmetric SMA reactions. A wide range of sulfur containing 1,2-difunctionalized ethylene synthons can be prepared with excellent optical purity. Importantly, the NHC acts as a unique non-covalent glue to link both reaction partners in a highly facial discriminating manner.

## Result and discussion

We are particularly interested in the synthesis of quaternary chiral centers containing two bio-friendly functional groups: CF_3_ and sulfur. Access to such chirality remains a considerable challenge.9q,^11^ We initiated our investigation using 2-phenylethanethiol **1a** and (*E*)-1-phenyl-1-trifluoromethyl-2-nitroethene **2a**. Although asymmetric SMA reactions involving nitroolefins are well documented,9o,^12^ the use of β-CF_3_-β-disubstituted substrates, such as **2a**, has not been successful. A recent paper reported up to 16% ee for the addition of thiophenols to β-CF_3_-β-phenyl nitroolefins using a tertiary amine/thiourea catalyst.11*b* SMA reactions are often reversible under strong base catalysis. Hexafluoroisopropanol (HFIP) was chosen to facilitate facile protonation of the primary adduct anion. Gratifyingly, we found that chiral triazolium salts were excellent NHC precatalysts for this transformation.^13^ The desired SMA product was obtained in high yield and excellent enantioselectivity. 4 Å molecular sieves were found to have a small beneficial effect on the selectivity and toluene appeared to be the most selective media for this C–S bond formation reaction.

With the optimized reaction conditions in hand, we sought to systematically examine the scopes of thiols and β-substituents of nitroolefins (Table 1). Simple aliphatic mercaptans were particularly effective. High yields and ee were observed for both primary and secondary thiols (products **3aa–3ia**). Sterically demanding tertiary mercaptans were not tolerated. When more acidic benzyl mercaptans and thiophenol were used, the ee of the product decreased slightly (products **3ka–3oa**). Crossover experiments were carried out and the C–S bond formation was not reversible under the reaction conditions.

**Table 1:** The scope of the SMA reaction involving β-CF_3_-β-aryl nitroalkenes^*a*^

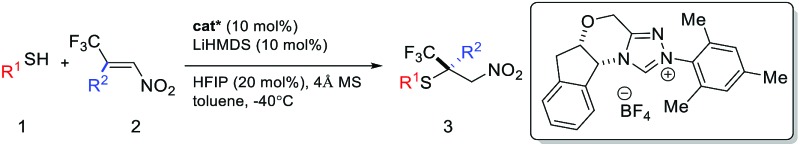
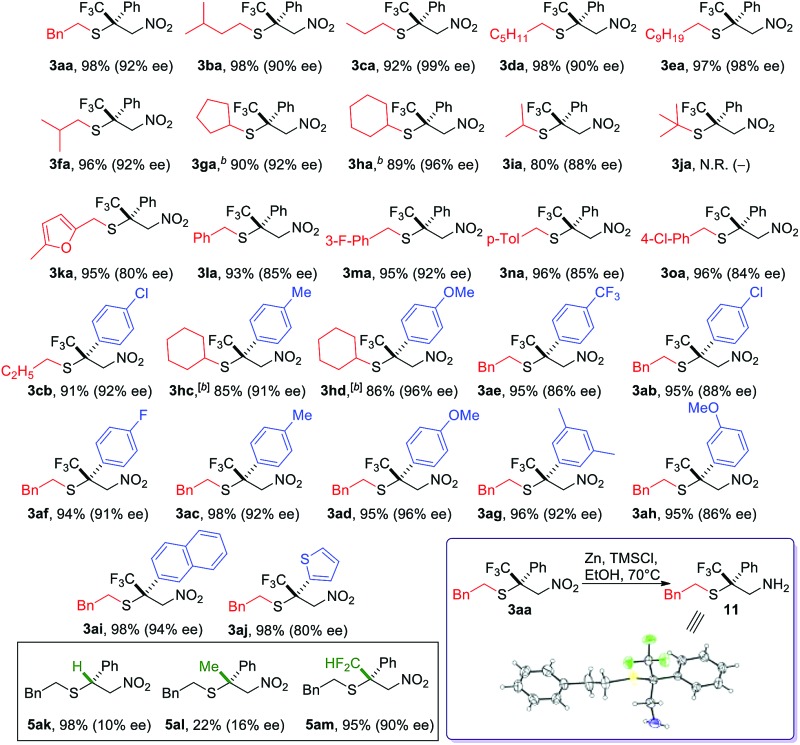

^*a*^Conditions: **1** (0.3 mmol), **2** (0.1 mmol), precatalyst (10 mol%), HFIP (20 mol%), and 4 Å MS (100 mg) in toluene (1.2 mL) at –40 °C for 6 h under an Ar atmosphere (balloon), unless otherwise noted. Isolated yield. ee was determined by chiral HPLC.

^*b*^The reaction time was 12 h.

An interesting electronic effect of the β-aryl substituent was noticed. The enantioselectivity of the reaction was sensitive to the electron density of the β-aryl group. Aryls bearing an electron-donating group afforded particularly high ee. For example, electron-rich β-*p*-methoxyphenyl nitroolefin afforded higher ee (96%, **3ad**) than the electron-poor *p*-CF_3_ phenyl analogue (86%, **3ae**). This selectivity discrepancy suggests a possible weak π–π stacking between the β-aryl and the catalyst (*vide infra*).

The role of the β-CF_3_ substituent was also investigated. When this group was removed, the reaction proceeded in high yield and poor ee (Table 1, **5ak**). Both the reactivity and selectivity were attenuated when the CF_3_ was replaced by a methyl group. The corresponding product was obtained in 22% yield with 16% ee (**5al**). Notably, when this methyl group was changed to CF_2_H, both the reactivity and selectivity were restored (**5am**). This interesting fluorine effect might be a result of hydrogen bonding or enhanced lipophilic interactions.

Compared to nitroolefins, enones are noticeably less common thiol acceptors. So far, there is only one report on an enantioselective SMA reaction involving β-CF_3_-β-disubstituted enones, in which a special class of thiols, mercaptoaldehydes, were used as the sulfur source.11*a* Reactions using simple thiols remained unknown. To our delight, the NHC catalyzed SMA reaction could be extended to β-CF_3_-β-aryl enones using benzyl mercaptans. Interestingly, HFIP had a stronger impact on the reactions of enones than olefins. In the absence of this catalytic proton shuttle, the reaction was very slow at –40 °C (43% yield, 12 h) and the product was nearly racemic (10% ee). Upon addition of a catalytic amount of HFIP (20 mol%), the reaction proceeded smoothly, even at –78 °C.

A broad scope of β-aryl groups were well tolerated (Table 2). Benzyl mercaptans showed the highest reactivity and selectivity. Aliphatic benzeneethanethiol reacted with β-CF_3_-β-anisolyl vinyl ketone in 84% yield and 87% ee (product **6ae**). The reaction did not proceed for sterically demanding alkyl mercaptans. Interestingly, the stereochemical properties of the ketone moiety had a strong influence on the selectivity. Small alkyl ketones afforded the highest conversion and ee. When a *t*-Bu ketone (*vs.* methyl ketone) was used, very little product was obtained. The reactivity of the enone was restored when a phenyl ketone was used. In this case, due to the lowered p*K*_a_ of the product, the proton shuttling was not efficient, and decreased selectivity was observed (product **6lh**).

**Table 2:** The substrate scope for β-CF_3_-β-aryl enones^*a*^

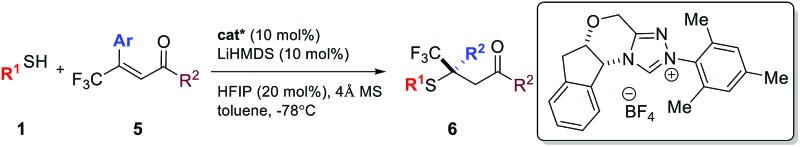
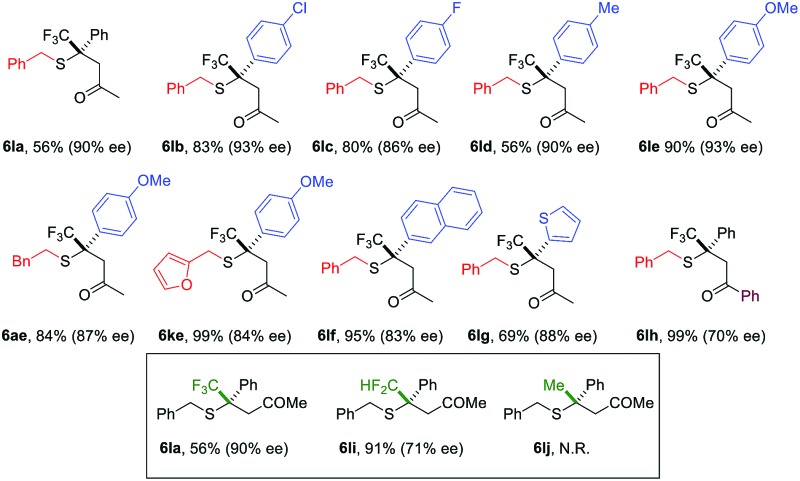

^*a*^Conditions: **1** (0.3 mmol), **5** (0.1 mmol), precatalyst (10 mol%), HFIP (20 mol%), and 4 Å MS (100 mg) in toluene (1.2 mL) at –78 °C for 48 h under an Ar atmosphere (balloon), unless otherwise noted. Isolated yield. ee was determined by chiral HPLC.

Similar to nitroolefins, the β-CF_3_ group was essential to maintain the ee for β,β-disubstituted enones. When the CF_3_ group was replaced by CF_2_H, the ee of the product decreased to 71% (Table 2, **6li**). A fluorine-free substrate did not react under the standard reaction conditions. It seemed that a delicate stereoelectronic balance at the β-carbon was required for enone substrates. The CF_3_ group not only enhances the reactivity of the enone through an inductive effect, but also provides steric bulk for facial differentiation.

Currently, reports on enantioselective SMA reactions involving enones have a very narrow scope. In most cases, only chalcones or cyclic enones gave good levels of enantioselectivity.9g,^14^ There is only one example involving simple alkyl enones in a single report using an Fe(iii)-Salen Lewis acid catalyst.9*g* In order to expand the non-covalent NHC catalysis to general enone substrates, we decided to investigate an SMA reaction using (*E*)-pent-3-en-2-one **7a**.

Under the standard reaction conditions for β-CF_3_-β-aryl enones, no reaction occurred between **7a** and benzyl mercaptan. A small amount of the SMA adduct was observed when the reaction temperature was raised to –40 °C. The ee for this product was merely 26%. Interestingly, a higher ee (54%) was observed when both the proton shuttle (HFIP) and molecular sieves were removed from the reaction. Control experiments showed that HFIP in fact had a deteriorating effect on both conversion and yield, a sharp contrast to nitroolefins and disubstituted enones. To our surprising delight, both high yield and ee were reestablished using 4 Å MS as an additive alone. The corresponding β-thioketone was formed in 92% yield and 85% ee at –78 °C. The reaction was largely affected by the inorganic base used to generate the free NHC catalyst. Only the lithium salt gave good levels of enantioselectivity. The reaction using NaHMDS yielded 20% ee. These combined results suggest that the SMA adduct anion for simple enones is basic enough to turn over the NHC catalyst in the absence of an external proton shuttle. In the case of HFIP, this strong solvating additive might have disrupted the delicate hydrogen bonded networks in the transition state. Other chiral triazolium salts were examined and Bode's scaffold afforded the best selectivity.

With the optimized conditions for simple enones in hand, we determined the reaction generality for both enones and thiols (Table 3). A high degree of asymmetric control was observed for benzyl mercaptans. Substituted benzyl and furfuryl mercaptans gave a decent to good ee. Simple aliphatic thiols reacted faster, but with lower selectivity. Various R^2^ groups were well tolerated at the β-position of the enone. In particular, sterically demanding R^2^ groups led to excellent ee. Both alkyl and aryl groups were suitable R^3^ groups for the enone. A modest electronic effect was observed for aryls. Substrates containing an electron-rich aryl group gave a higher ee than their electron-poor counterparts. We propose that π–π stacking between the NHC and the enone might be responsible for this selectivity discrepancy.

**Table 3:** The substrate scope for simple enones^*a*^

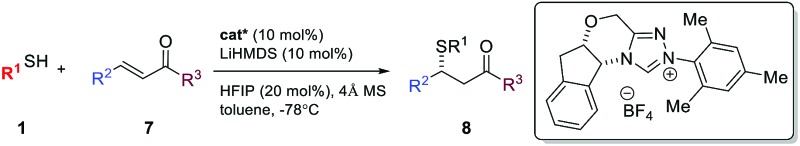
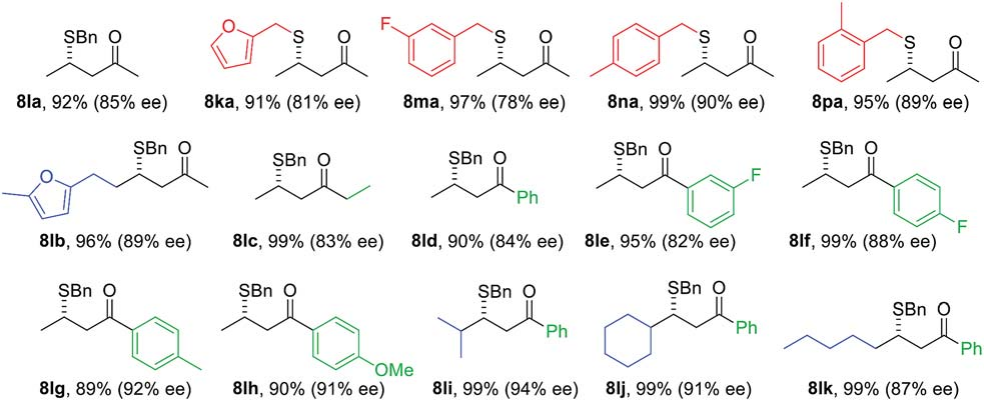

^*a*^Conditions: **1** (0.3 mmol), **5** (0.1 mmol), precatalyst (10 mol%), HFIP (20 mol%), and 4 Å MS (100 mg) in toluene (1.2 mL) at –78 °C for 48 h under an Ar atmosphere (balloon), unless otherwise noted. Isolated yield. ee was determined by chiral HPLC.

**Scheme 2:**
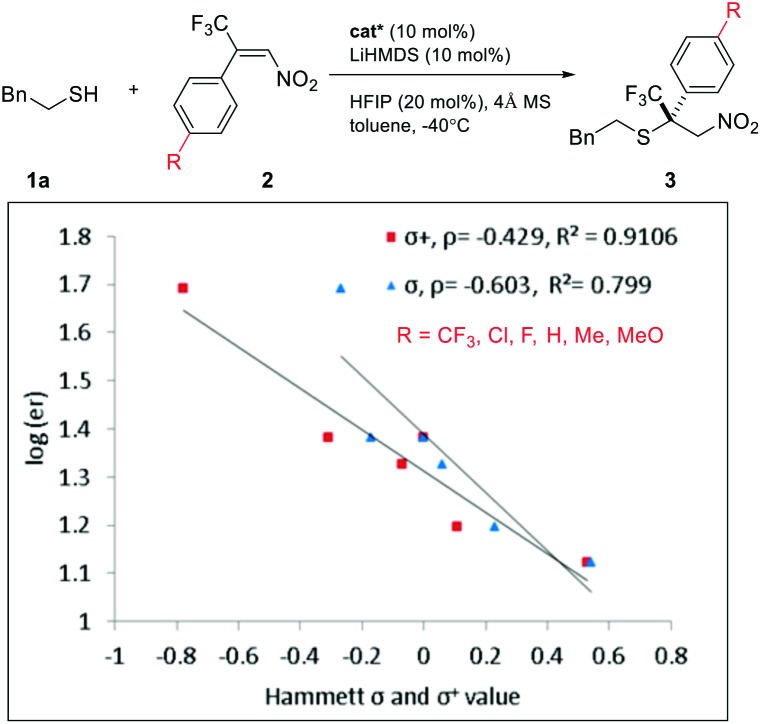
LFER between substrate electronics and enantioselectivity

Intrigued by the electronic effect of the Michael acceptors on enantioselectivity, we investigated the linear free-energy relationship (LFER) between *para*-substituents and the enantiomeric ratios of their corresponding products (Scheme 2).^15^ A LFER was obtained for nitroolefins by plotting log(er) *vs.* Hammett *σ para* values, with *ρ* = –0.6 (*R*^2^ = 0.799). The negative slope indicates a positive charge buildup or negative charge diminishment during the transition state. Additionally, a Hammett plot was constructed by plotting log(er) *vs. σ*^+^ values with *ρ* = –0.4 (*R*^2^ = 0.910). The better fit obtained with *σ*^+^ than with *σ* indicates a significant resonance contribution from electron donating groups.15a,15i,^16^ This result suggests a weak π–π stacking between the nitroolefin and the NHC heterocycle. This non-covalent interaction might bring together an intriguing sandwich-like structure for the thiol–NHC–olefin “complex”. Within this trimeric microstructure, one prochiral face of the double bond is in close proximity to the thiol for facile “intramolecular” C–S bond formation. A similar electronic effect was also observed for enones. The more electron-rich alkenes resulted in higher enantioselectivity by offering a stronger π–π stacking for the transition state leading to the major enantiomer.

**Scheme 3:**
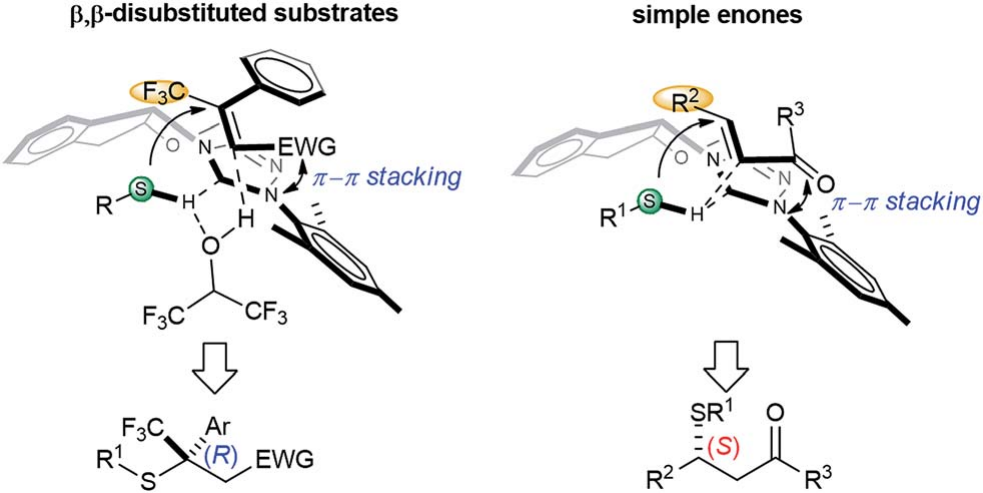
Proposed model for asymmetric induction.

Based on the aforementioned data, a simplified model for the asymmetric induction is proposed (Scheme 3). The NHC catalyst acts as a chiral Brønsted base and activates the acidic mercaptan by forming a hydrogen bonded complex. The double bond of the thiol acceptor forms a π–π stacking interaction with the catalyst. The large CF_3_ group points away from the bulky aryl group of the catalyst. This thiol–NHC–olefin 3D alignment orients the *Si*-face of the Michael acceptor in close proximity to the hydrogen bonded thiol for an “intramolecular” delivery. This model predicts the chiral center of the product as having an *R* configuration for nitroolefins, in agreement with the X-ray data. HFIP might serve as a proton shuttle to facilitate proton transfer from the thiol to the primary Michael adduct anion. The function of the NHC and HFIP resembles a protein–ligand interaction that creates a highly sophisticated microenvironment for both substrate activation and asymmetric recognition. An analogous analysis can be applied to β,β-disubstituted enones. For simple enones, the bulky R^2^ group is positioned away from the large aryl group of the NHC. In this case, direct proton transfer from the thiol to the product is more likely, due to the strong basicity of the ketone enolate. The absolute stereochemistry of the products from enones were determined by comparing the signs of optical rotation data to that of literature known compounds.9g,9q,14g

## Conclusions

In summary, we have developed a general enantioselective SMA reaction of mercaptans with various electron deficient alkenes using chiral NHCs a new class of non-covalent organocatalysts. This method is not only effective for synthesizing CF_3_ and S containing quaternary chiral centers, but is also applicable to simple enone substrates. Mechanistic studies suggested that the NHC might serve as a dual functional catalyst through non-covalent interactions: hydrogen bonding and π–π stacking. We expect that the use of NHCs as a new class of non-covalent organocatalysts will find broad application in asymmetric carbon-heteroatom bond formation reactions.

## Supplementary Material

Supplementary information

Crystal structure data
