# Supplementary material for: Highly enantioselective sulfa-Michael addition reactions using N-heterocyclic carbene as a non-covalent organocatalyst
Source: Chem Sci. 2015 Apr 23;6(7):4184–9. doi: 10.1039/c5sc00878f (PMC5707481; doi:10.1039/c5sc00878f)
Supplement: Supplementary file 1 [file SC-006-C5SC00878F-s001.pdf]

Electronic Supplementary Material (ESI) for Chemical Science.
This journal is © The Royal Society of Chemistry 2015

Highly Enantioselective Sulfa-Michael Addition Reactions Using N-heterocyclic Carbene as a Non-covalent Organo-catalyst

Jiean Chen,[‡] Sixuan Meng,[‡] Leming Wang, Hongmei Tang, and Yong Huang*
Key Laboratory of Chemical Genomics, School of Chemical Biology and Biotechnology,
Peking University, Shenzhen Graduate School, Shenzhen, 518055, China
[‡]These authors contributed equally to this work.

Support Information

Table of Contents:

General methods and materials 2
General procedure 3-4
Condition screening 5-7
Characterization 8-118
X-Ray single crystal structure of 3aa's derivative 119
Supplementary References 119

General Methods and Materials:

All solvents were distilled according to general practice prior to use. All reagents were purchased and used without further purification unless specified otherwise. Solvents for flash column chromatography were technical grade and distilled prior to use. Analytical thin-layer chromatography (TLC) was performed using Huanghai silica gel plates with HSGF 254. Visualization of the developed chromatogram was performed by UV absorbance (254 nm) and appropriate stains. Flash column chromatography was performed using Qingdao Haiyang Chemical HG/T2354-92 silica gel (200-300 mesh) with the indicated solvent system according to standard techniques. ^{1}H NMR and ^{13}C NMR data were recorded on Bruker 400 MHz (100 MHz for ^{13}C, 376MHz for ^{19}F) nuclear resonance spectrometers unless otherwise specified, respectively. Chemical shifts (δ) in ppm are reported as quoted relative to the residual signals of chloroform (^{1}H 7.26 ppm and ^{13}C 77.16 ppm). Multiplicities are described as: s (singlet), bs (broad singlet), d (doublet), t (triplet), q (quartet), m (multiplet); and coupling constants (*J*) are reported in Hertz (Hz). ^{13}C NMR spectra were recorded with total proton decoupling. Chiral HPLC was recorded on a Shimadzu LC-20A spectrometer using Daicel Chiralcel^{TM} columns. HRMS (ESI) analysis was performed by The Analytical Instrumentation Center at Peking University; Shenzhen Graduate School and (HRMS) data were reported with ion mass/charge (m/z) ratios as values in atomic mass units. β-CF_3-β-Disubstituted nitroolefins were synthesized by the procedure published by Jia Y.-X. and coworker.[1] β-CF_3-β-Disubstituted enones were synthesized by the procedure published by Cahard D. and coworker.[2] Racemic samples were prepared by the following procedure: alkene substrate (0.2 mmol, 1.0 equiv.) and mercaptan (0.6mmol, 3.0 equiv.) were dissolved in 1.2 mL of DCM and the resulting clear solution was cooled to -20 ^{o}C and DBU (6μL, 0.2 equiv.) was added slowly. The reaction mixture was stirred for 30 min and the solvent was removed under reduced pressure. The crude mixture was purified by flash column chromatography (50:1 hexanes : EtOAc).

General procedure for NHCs catalyzed Sulfa-Michael addition.

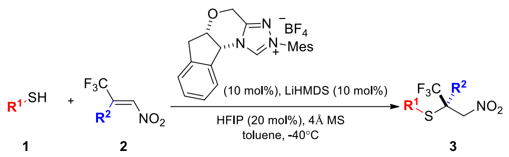

General procedure A for the NHC catalyzed Michael addition: NHC catalyst (4.2 mg, 0.1 equiv.) and 4-Å oven-dried molecular sieves (100 mg) were dissolved in dry toluene (0.6 mL) in a 10-mL test tube. The mixture was degassed and back-filled with argon (3x) before LiHMDS (1M in tetrahydrofuran (THF)/ethylbenzene, 10 µL, 0.1 equiv.) was slowly added. The reaction vessel was degassed and again back-filled with argon, and HFIP (2.2µL, 0.2 equiv.) was added through a micro-syringe. The test tube was sealed with a rubber septum and stirred at -40 °C for 1 h. Then compound **1** (0.3 mmol, 3.0 equiv.) was slowly added, and the mixture was stirred for 1 h at -40 °C. A solution of compound **2** (0.1 mmol, 1.0 equiv.) in toluene (0.6 mL) was slowly added over 30 m, and the resulting mixture was stirred at -40 °C for 6 h. Upon complete consumption of compound **2**, the reaction was filtered through silica gel and concentrated. The residue was purified by silica-gel flash-column chromatography (eluent: hexane/EtOAc=50:1) to afford the desired addition product. The enantioselectivity was determined by chiral HPLC.

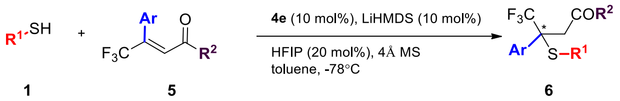

General procedure B for the NHC catalyzed Michael addition: NHC catalyst (4.2 mg, 0.1 equiv.) and 4-Å oven-dried molecular sieves (100 mg) were dissolved in dry toluene (0.6 mL) in a 10-mL test tube. The mixture was degassed and back-filled with argon (3x) before LiHMDS (1M in tetrahydrofuran (THF)/ethylbenzene, 10 µL, 0.1 equiv.) was slowly added. The reaction vessel was degassed and again back-filled with argon, and HFIP (2.2µL, 0.2 equiv.) was added through a micro-syringe. The test tube was sealed with a rubber septum and stirred at room temperature for 30 m and another 30 m for -78 °C. Then compound **1** (0.3 mmol, 3.0 equiv.) was slowly added, and the mixture was stirred for 30 min at -78 °C. A solution of compound **5** (0.1 mmol, 1.0 equiv.) in toluene (0.6 mL) was slowly added over 30 m, and the resulting mixture was stirred at -78 °C for 48 h. Upon complete consumption of compound **5**, the reaction was filtered through silica gel and concentrated. The residue was purified by silica-gel flash-column chromatography (eluent: hexane/EtOAc=20:1) to afford the desired addition product. The enantioselectivity was determined by chiral HPLC.

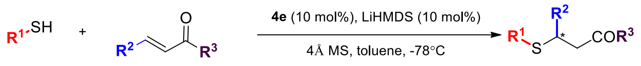

1 7 8

General procedure C for the NHC catalyzed Michael addition: NHC catalyst (4.2 mg, 0.1 equiv.) and 4-Å oven-dried molecular sieves (100 mg) were dissolved in dry toluene (0.6 mL) in a 10-mL test tube. The mixture was degassed and back-filled with argon (3x) before LiHMDS (1M in tetrahydrofuran (THF)/ethylbenzene, 10 µL, 0.1 equiv.) was slowly added. The reaction vessel was degassed and again back-filled with argon. The test tube was sealed with a rubber septum and stirred at room temperature for 30 m and another 30 m for -78 °C. Then compound **1** (0.3 mmol, 3.0 equiv.) was slowly added, and the mixture was stirred for 30 min at -78 °C. A solution of compound **7** (0.1 mmol, 1.0 equiv.) in toluene (0.6 mL) was slowly added over 30 m, and the resulting mixture was stirred at -78 °C for 48 h. Upon complete consumption of compound **7**, the reaction was filtered through silica gel and concentrated. The residue was purified by silica-gel flash-column chromatography (eluent: hexane/EtOAc=20:1) to afford the desired addition product. The enantioselectivity was determined by chiral HPLC.

Condition screening for NHCs catalyzed Sulfa-Michael addition.

Table 1. Reaction optimization for β-CF_3-β-aryl nitroalkenes

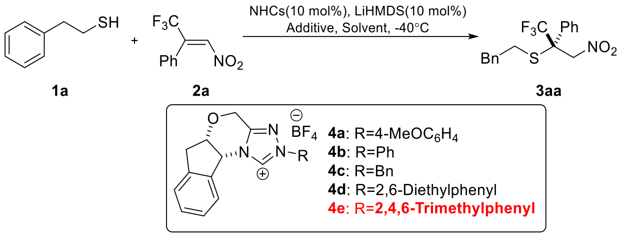

entry	catalyst	additive	solvent	ee (%)[b]
1	4a	HFIP, 4ÅMS	PhMe	-7
2	4b	HFIP, 4ÅMS	PhMe	-26
3	4c	HFIP, 4ÅMS	PhMe	12
4	4d	HFIP, 4ÅMS	PhMe	93
5	4e	HFIP, 4ÅMS	PhMe	92
6[c]	4e	—	PhMe	66
7	4e	HFIP	PhMe	90
8[d]	4e	HFIP, 4ÅMS	THF	45
9	4e	HFIP, 4ÅMS	MTBE	56
10	4e	HFIP, 4ÅMS	DCM	79
11	4e	HFIP, 4ÅMS	Et_2O	89

[a] Conditions: **1a** (0.3 mmol), **2a** (0.1 mmol), NHC precatalyst (10 mol%), HFIP (20 mol%), and 4Å molecular sieves (100 mg) in solvent (1.2 mL) at -40°C for 6 h. Quantitative conversion unless specified. [b] Determined by chiral HPLC. [c] 25% yield. [d] 50% yield.

The electronic properties of the aryl substituent of the triazolium precatalyst had little effect on reaction conversion (Table 1, entries 1-5). The desired SMA product was obtained in quantitative yield for most catalysts examined. The selectivity, on the other hand, was highly sensitive to the steric environment of the aryl group. 2,6-Dialkyl substitution was essential for high ee (Table 1, entries 4, 5). In the absence of HFIP, the reaction became very slow and modest ee was observed (Table 1, entry 6). Other acidic additives were extensively screened and only HFIP afforded better result than the additive-free condition. 4Å Molecular sieves had a small beneficial effect on the selectivity. Toluene appeared to be the most selective media for this C-S bond formation reaction.

Table 2. Reaction optimization for enones

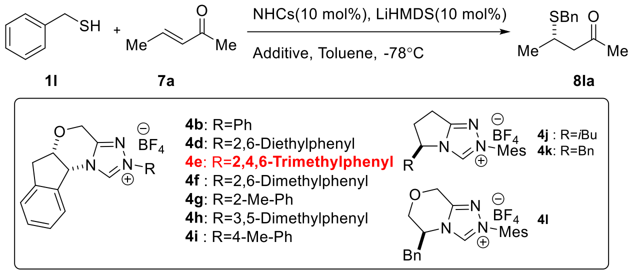

entry	catalyst	additive	solvent	yield (%)[b]	ee(%)[c]
1	4e	HFIP, 4ÅMS	Toluene	NR	-
2[d]	4e	HFIP, 4ÅMS	Toluene	<10	26
3[d]	4e	-	Toluene	<10	54
4[d]	4e	HFIP	Toluene	<10	21
5[d]	4e	4ÅMS	Toluene	92	75
6	4e	4ÅMS	Toluene	92	85
7[e]	4e	4ÅMS	Toluene	71	20
8	4e	5ÅMS	Toluene	82	85
9	4e	4ÅMS	THF	-	-
10	4e	4ÅMS	Et_2O	80	71
11	4e	4ÅMS	DCM	80	69
12	4e	4ÅMS	MeOH	77	0
13	4b	4ÅMS	Toluene	90	6
14	4d	4ÅMS	Toluene	92	84
15	4f	4ÅMS	Toluene	92	81
16	4g	4ÅMS	Toluene	92	37
17	4h	4ÅMS	Toluene	92	6
18	4i	4ÅMS	Toluene	92	6
19	4j	4ÅMS	Toluene	92	-43
20	4k	4ÅMS	Toluene	92	-49
21	4l	4ÅMS	Toluene	92	-62

[a] Conditions: **1a** (0.3 mmol), **2a** (0.1 mmol), NHC precatalyst (10 mol%), 4Å molecular sieves (100 mg) in solvent (1.2 mL) at -78 ºC for 12 h. [b] Yield was Determined by GC-MS. [c] Determined by chiral HPLC. [d] The reaction was conducted under -40 ºC. [e] NaHMDS was used to generate the free NHC catalyst.

Calculation of GC yields: Biphenyl was used as the external standard. The GC coefficient was calculated by dividing the peak areas (1.0 eq. biphenyl vs 1.0 eq. **8la**). The crude reaction was added 1.0 eq. biphenyl and passed through a plug of silica gel, which was washed thoroughly using ether. The eluent was subjected to GC and the yield was calculated based on the following formulas.

$$\frac{S(product)}{S(biphenyl)} = a \qquad Yield(GC) = \frac{S(product\ of\ reaction)}{S(biphenyl) * a}$$

Under the standard reaction condition for β-CF_3-β-aryl enones, no reaction occurred using **7a** as the reaction partner for benzyl mercaptan. A small amount of the SMA adduct was observed when the reaction temperature was raised to -40 ^{o}C. The ee for this product was merely 26% (Table 2, entry 2). We were surprised to observe a higher ee (54%) when both the proton shuttle and molecular sieves were removed from the reaction (Table 2, entry 3). Control experiments showed that HFIP had a deteriorating effect on both conversion and yield (Table 2, entry 4), a sharp contrast to nitroolefins and disubstituted enones. To our surprising delight, both high yield and ee were reestablished using 4Å MS as additive alone. Product **8la** was formed in 92% conversion with 85% ee at -78ºC. The reaction was largely affected by the inorganic base used to generate free NHC catalyst. Only lithium salt gave good level of enantioselectivity. The reaction using NaHMDS yielded 20% ee. The combined result suggests the SMA adduct anion for simple enone was basic enough to turn over the NHC catalyst without an external proton shuttle. Therefore, HFIP might disrupt the strong lithium effect through cation solvation. The selectivity remained highest in toluene. A racemic reaction occurred in methanol. Other chiral triazolium salts were examined and the Bode's scaffold afforded the best selectivity.

(R)-phenethyl(1,1,1-trifluoro-3-nitro-2-phenylpropan-2-yl)sulfane (3aa)

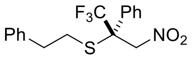

3aa

The title compound was prepared according to the general procedure A and purified by flash column chromatography (50:1 hexanes : EtOAc) to afford **3aa** (35 mg, 98%) as a colorless oil. Analytical data: IR (KBr, cm^{-1}) 2920, 2312, 1560, 1369, 1218, 1149, 696; ^{1}H NMR (500 MHz, $CDCl_3$) δ 7.64 (d, *J* = 7.5 Hz, 2H), 7.52 – 7.35 (m, 3H), 7.35 – 7.19 (m, 3H), 7.10 (d, *J* = 7.2 Hz, 2H), 5.07 (s, 2H), 2.95 – 2.71 (m, 3H), 2.68 – 2.55 (m, 1H); ^{19}F NMR (376 MHz, $CDCl_3$) δ -66.44 (s, 3F); ^{13}C NMR (125 MHz, $CDCl_3$) δ 139.23 (s), 131.55 (s), 129.22 (s), 128.91 (s), 128.56 (s), 128.43 (s), 128.13 (d, *J* = 2.5 Hz), 126.70 (s), 125.93 (q, *J* = 282.5 Hz), 77.75 (s), 59.31 (q, *J* = 27.5 Hz), 34.84 (s), 32.13 (s). HPLC (AD-H, 5% EtOH in hexanes, 1 mL/min, 210 nm): t_{major} = 8.48 min, t_{minor} = 7.81 min, 92% ee; $^{25}[\alpha]_D$ = -11.7 ° (c = 1.0 in $CHCl_3$); HRMS (ESI+) Calcd for $C_{17}H_{16}F_3NO_2SNa^+$ $(M+Na)^+$: 378.0752, Found: 378.0806.

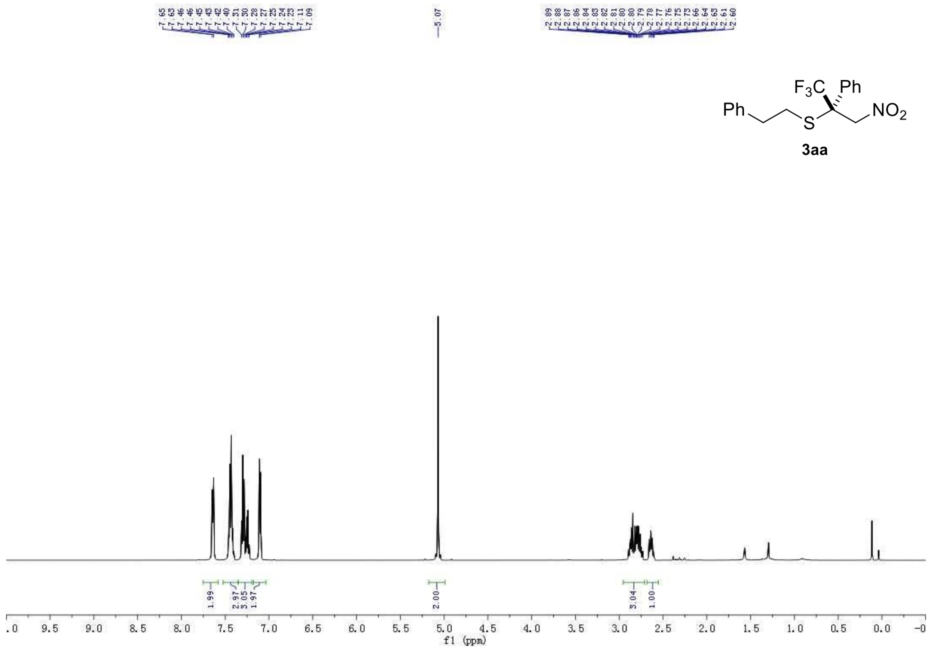

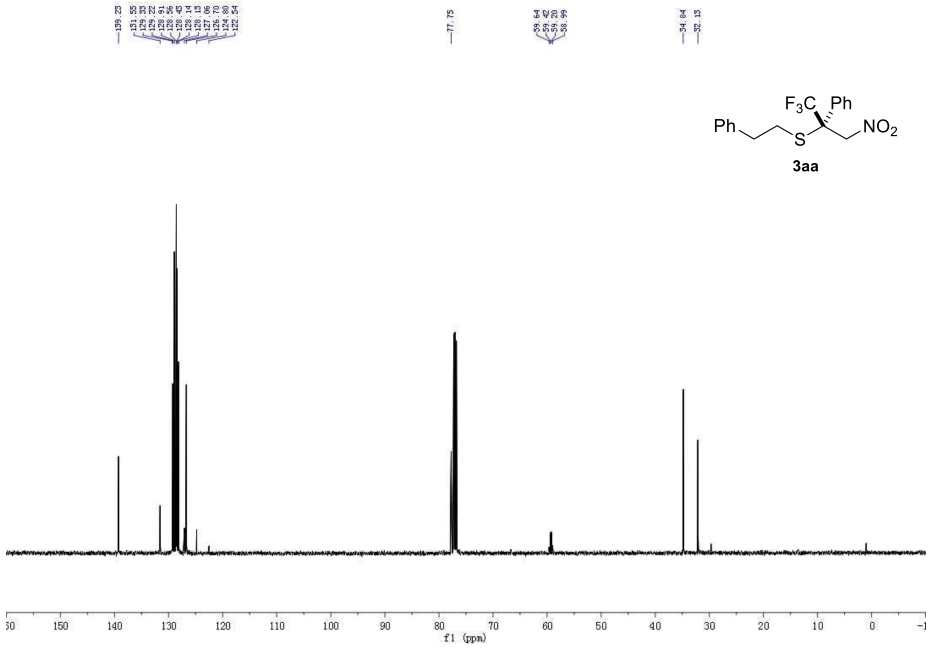

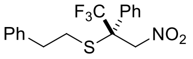

3aa

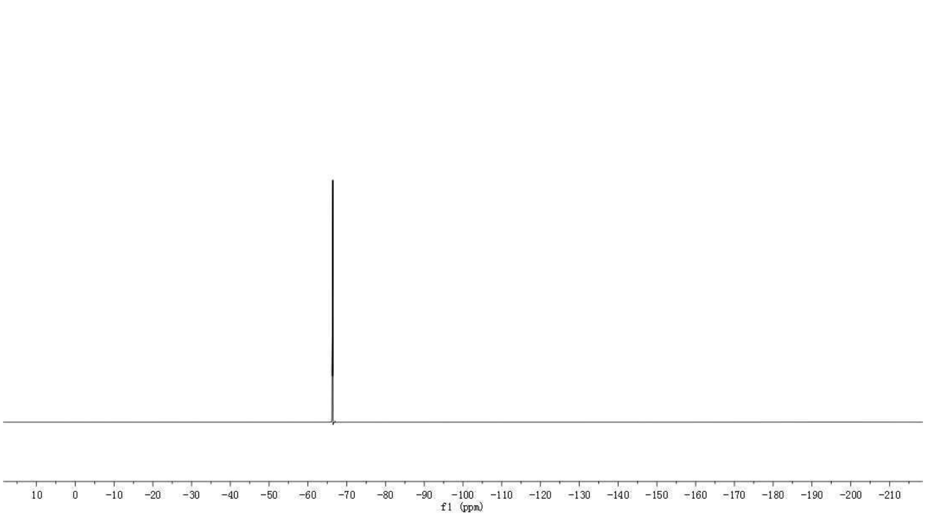

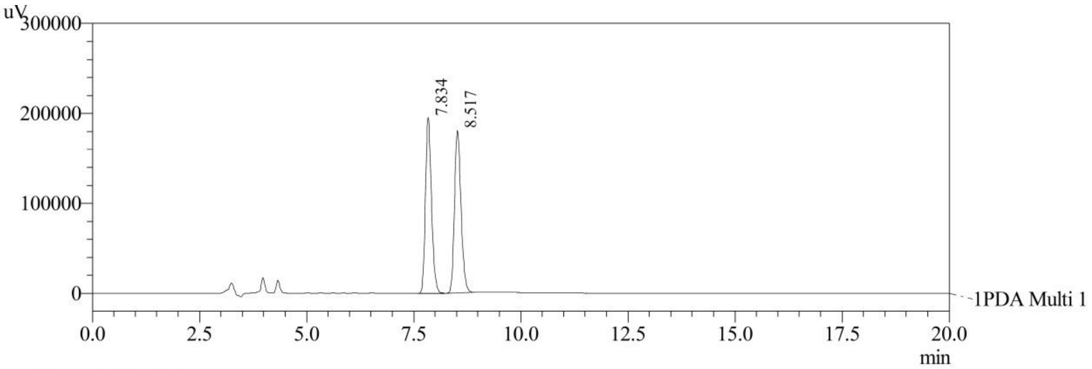

1 PDA Multi 1 / 210nm 4nm

PeakTable

PDA Ch1 210nm 4nm

Peak#	Ret. Time	Area	Height	Area %	Height %
1	7.834	1913065	195492	49.942	51.958
2	8.517	1917505	180761	50.058	48.042
Total		3830569	376253	100.000	100.000

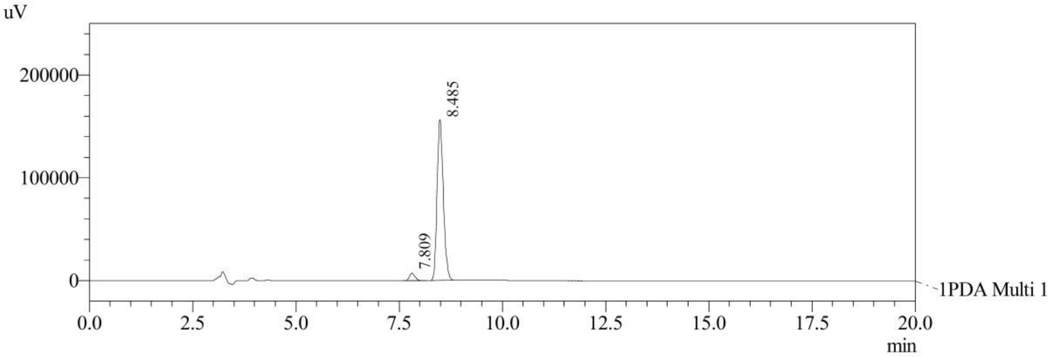

1 PDA Multi 1 / 210nm 4nm

PeakTable

PDA Ch1 210nm 4nm

Peak#	Ret. Time	Area	Height	Area %	Height %
1	7.809	73420	7472	4.250	4.552
2	8.485	1653961	156679	95.750	95.448
Total		1727381	164152	100.000	100.000

(R)-isopentyl(1,1,1-trifluoro-3-nitro-2-phenylpropan-2-yl)sulfane (3ba)

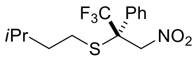

3ba

The title compound was prepared according to the general procedure A and purified by flash column chromatography (50:1 hexanes : EtOAc) to afford **3ba** (31 mg, 98%) as a colorless oil. Analytical data: IR (KBr, cm^{-1}) 2959, 1564, 1449, 1369, 1231, 1150, 1014, 761, 695; ^{1}H NMR (400 MHz, $CDCl_3$) δ 7.66 (d, J = 8.2 Hz, 2H), 7.52 – 7.34 (m, 3H), 5.09 (s, 2H), 2.68 – 2.51 (m, 1H), 2.45 – 2.27 (m, 1H), 1.60 (dp, J = 13.3, 6.7 Hz, 1H), 1.47 – 1.31 (m, 2H), 0.83 (dd, J = 6.6, 1.7 Hz, 6H); ^{19}F NMR (376 MHz, $CDCl_3$) δ -66.49 (s, 3F); ^{13}C NMR (100 MHz, $CDCl_3$) δ 131.62 (s), 129.12 (s), 128.83 (s), 128.04 (d, J = 2.0 Hz), 125.94 (q, J = 283.0 Hz), 124.54 (s), 121.71 (s), 77.64 (s), 58.96 (q, J = 27.4 Hz), 37.03 (s), 28.79 (s), 28.78 (s), 27.27 (s), 22.06 (s). HPLC (AD-H, 5% EtOH in hexanes, 1 mL/min, 210 nm): t_{major} = 5.6 min, t_{minor} = 7.3 min, 90% ee; $^{25}[\alpha]_D$ = -11.5 ^{o} (c = 1.0 in $CHCl_3$); HRMS (ESI+) Calcd for $C_{14}H_{18}F_3NO_2SNa^+$ $(M+Na)^+$: 344.0908, Found: 344.0900.

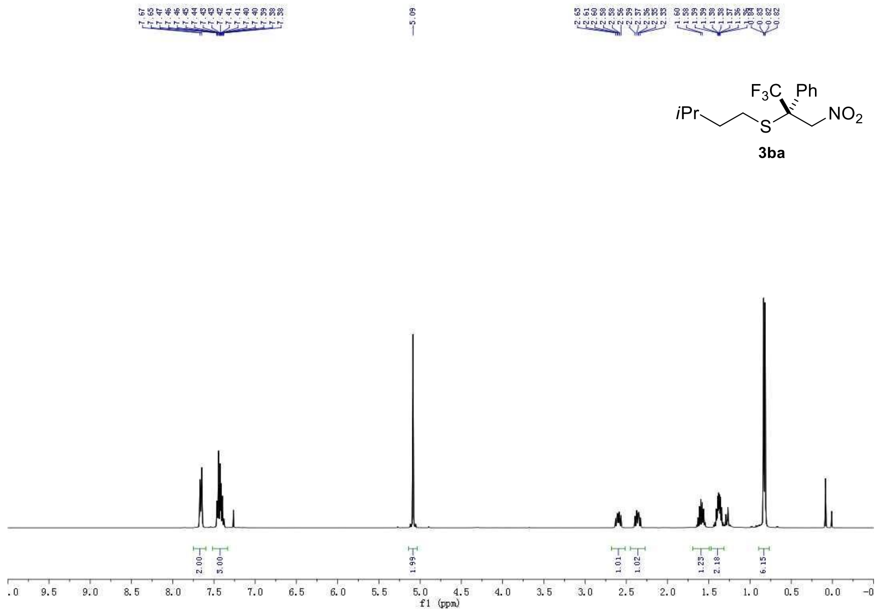

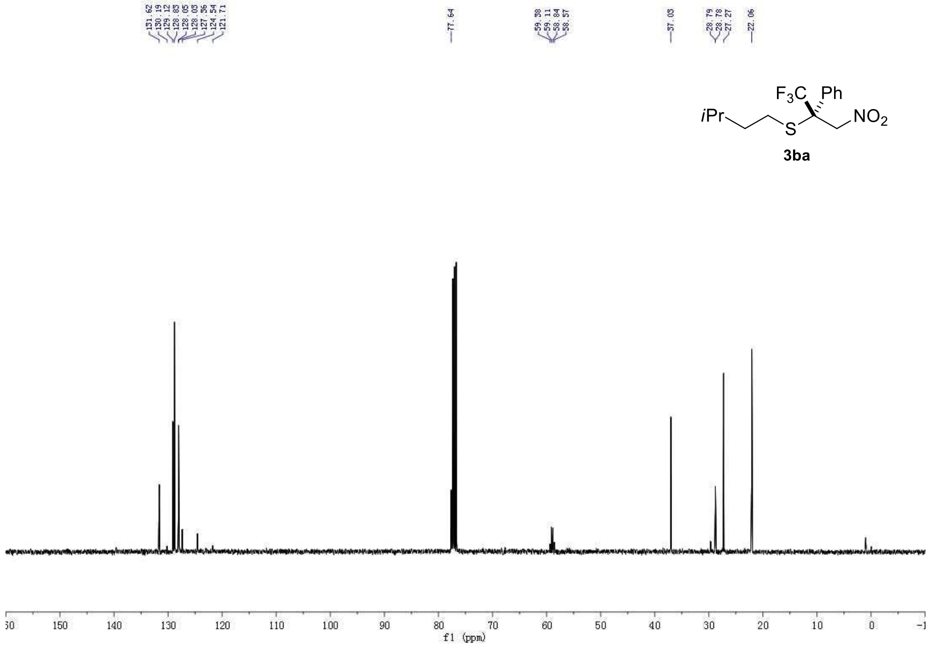

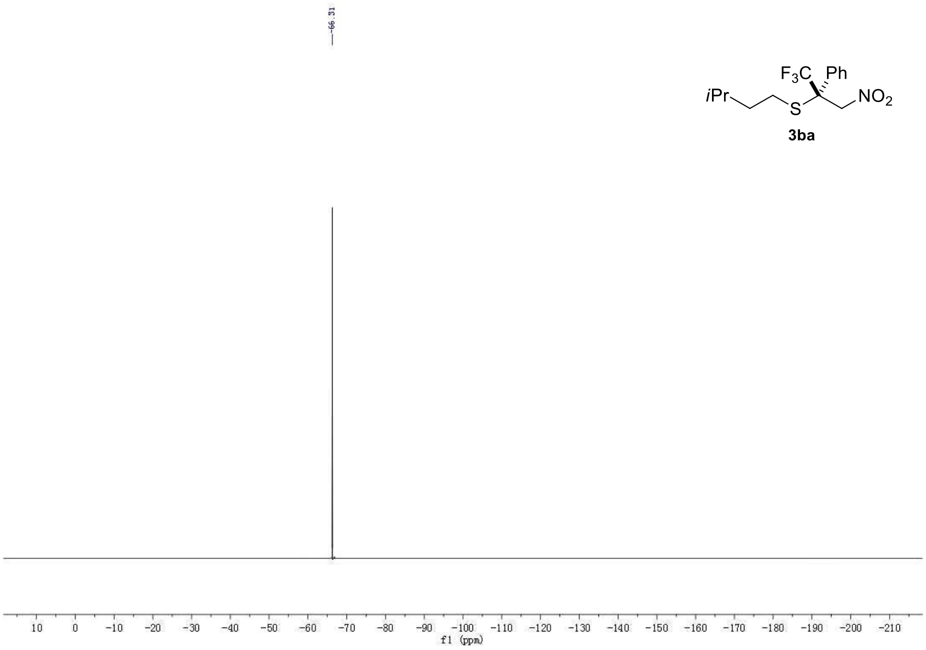

3ba

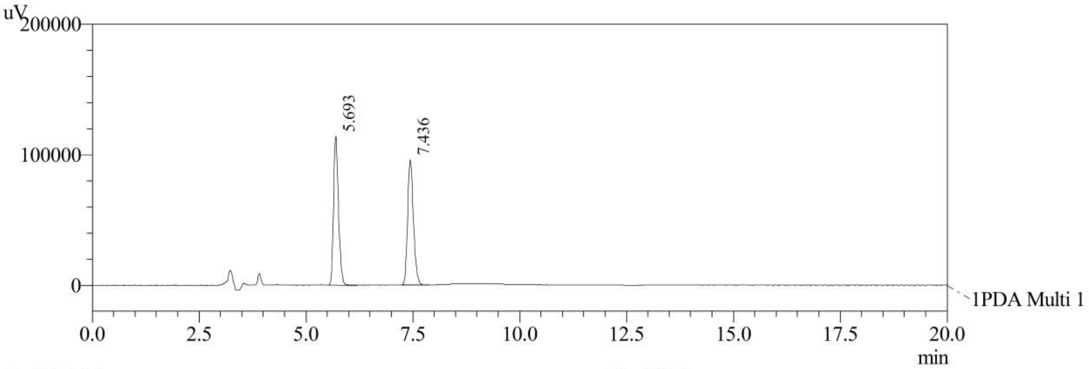

1 PDA Mu

PeakTable

PDA Ch1 210nm 4nm

Peak#	Ret. Time	Area	Height	Area %	Height %
1	5.693	919963	114132	50.111	54.172
2	7.436	915891	96552	49.889	45.828
Total		1835855	210684	100.000	100.000

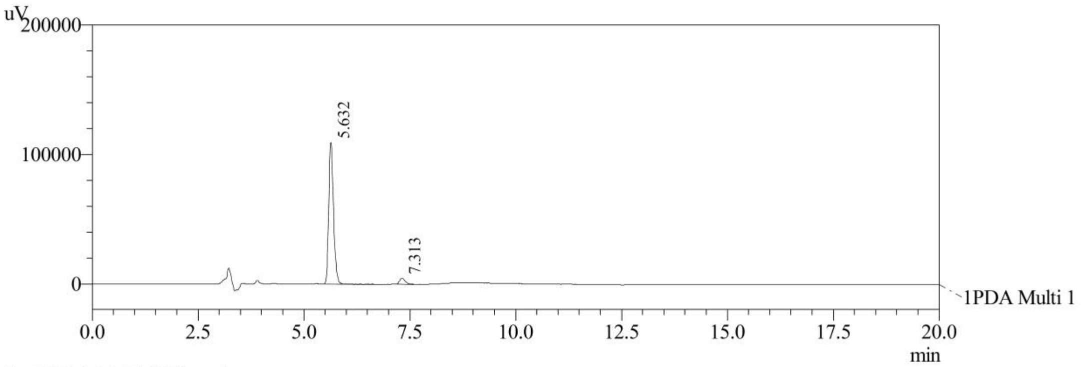

1 PDA Multi 1 / 210nm 4nm

PeakTable

PDA Ch1 210nm 4nm

Peak#	Ret. Time	Area	Height	Area %	Height %
1	5.632	852574	109287	95.198	95.896
2	7.313	43002	4677	4.802	4.104
Total		895576	113964	100.000	100.000

(R)-propyl(1,1,1-trifluoro-3-nitro-2-phenylpropan-2-yl)sulfane (3ca)

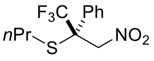

3ca

The title compound was prepared according to the general procedure A and purified by flash column chromatography (50:1 hexanes : EtOAc) to afford **3ca** (27 mg, 92%) as a colorless oil. Analytical data: IR (KBr, cm^{-1}) 2966, 2367, 1560, 1369, 1147, 695; ^{1}H NMR (400 MHz, $CDCl_3$) δ 7.66 (dd, *J* = 7.4, 0.9 Hz, 2H), 7.52 – 7.33 (m, 3H), 5.08 (s, 2H), 2.68 – 2.48 (m, 1H), 2.35 (m, 1H), 1.65 – 1.42 (m, 2H), 0.94 (t, *J* = 7.4 Hz, 3H); ^{19}F NMR (376 MHz, $CDCl_3$) δ -66.52 (s, 3F); ^{13}C NMR (100 MHz, $CDCl_3$) δ 131.65 (s), 129.12 (s), 128.83 (s), 128.07 (d, *J* = 1.0 Hz), 125.93 (q, *J* = 282.0 Hz), 77.68 (s), 58.92(q, *J* = 27.0 Hz), 32.66 (d, *J* = 1.3 Hz), 21.72 (s), 13.37 (s). HPLC (IA-H, 10% EtOH in hexanes, 1 mL/min, 210 nm): t_{major} = 11.7 min, t_{minor} = 22.7 min, 99% ee; $^{25}[\alpha]_D$ = -10.6 ° (c = 1.0 in $CHCl_3$); HRMS (ESI+) Calcd for $C_{12}H_{14}F_3NO_2SNa^+$ $(M+Na)^+$: 316.0595, Found: 316.0589.

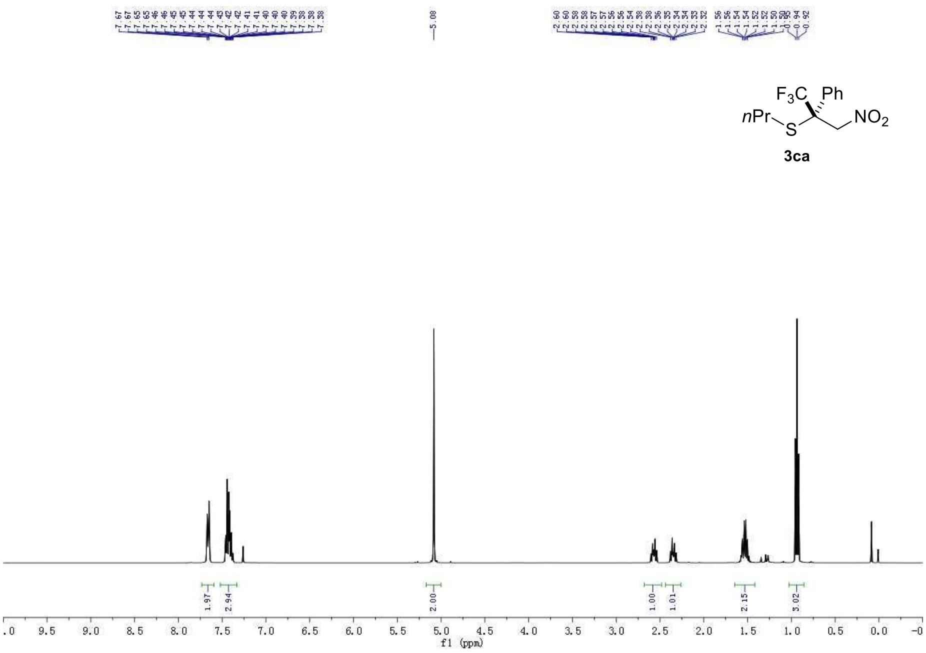

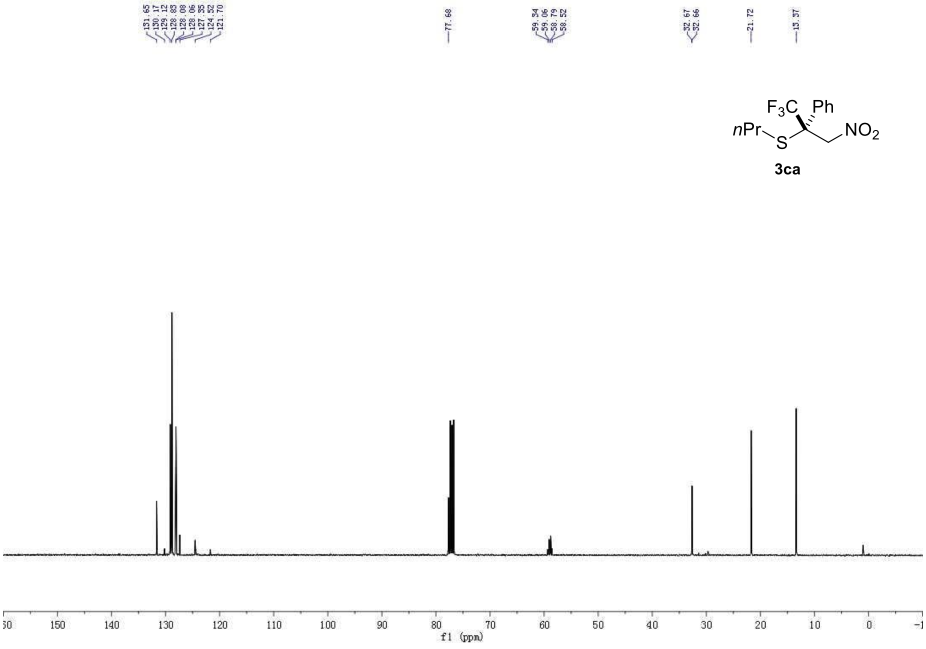

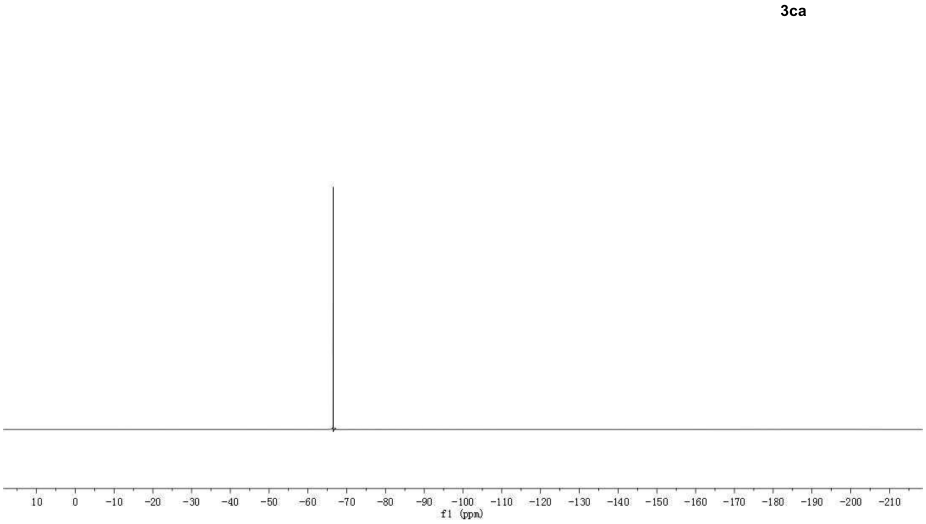

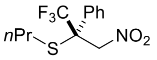

3ca

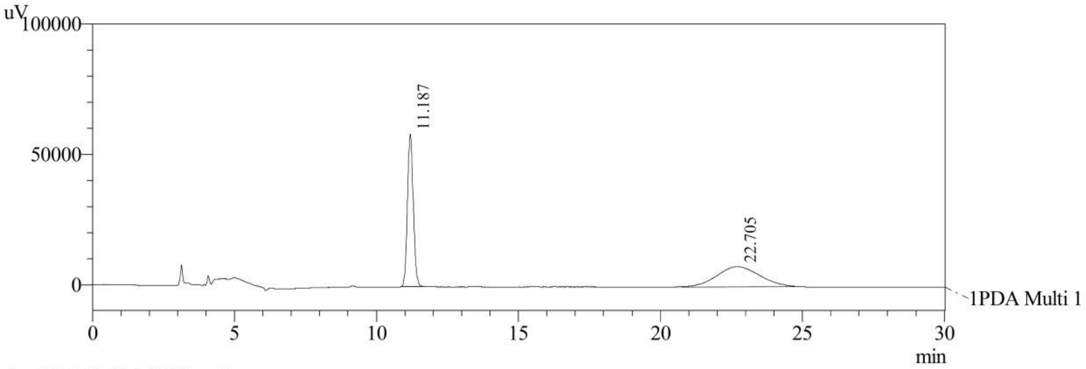

1 PDA Multi 1 / 210nm 4nm

PeakTable

PDA Ch1 210nm 4nm

Peak#	Ret. Time	Area	Height	Area %	Height %
1	11.187	834328	58563	50.464	88.347
2	22.705	818974	7725	49.536	11.653
Total		1653302	66288	100.000	100.000

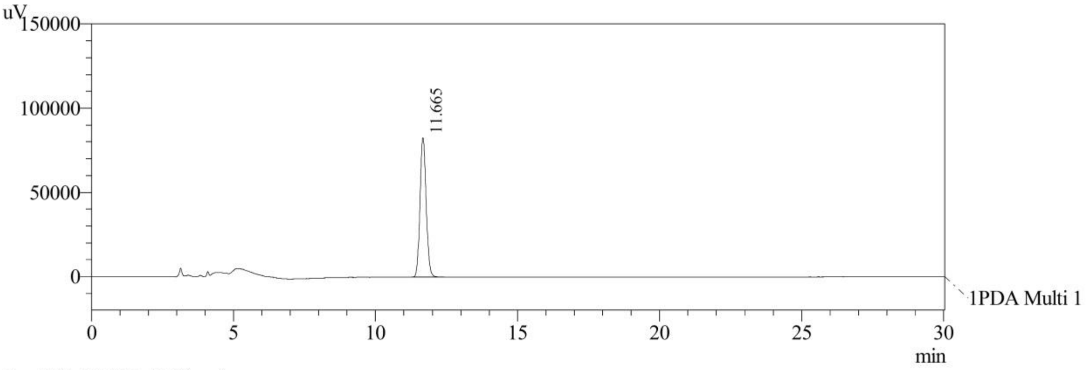

1 PDA Multi 1 / 210nm 4nm

PeakTable

PDA Ch1 210nm 4nm

Peak#	Ret. Time	Area	Height	Area %	Height %
1	11.665	1251542	82724	100.000	100.000
Total		1251542	82724	100.000	100.000

(R)-hexyl(1,1,1-trifluoro-3-nitro-2-phenylpropan-2-yl)sulfane (3da)

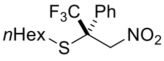

3da

The title compound was prepared according to the general procedure A and purified by flash column chromatography (50:1 hexanes : EtOAc) to afford **3da** (33 mg, 98%) as a colorless oil. Analytical data: IR (KBr, cm^{-1}) 2957, 1565, 1449, 1369, 1219, 1149, 1015, 695; ^{1}H NMR (400 MHz, $CDCl_3$) δ 7.66 (d, J = 8.1 Hz, 2H), 7.50 – 7.34 (m, 3H), 5.08 (s, 2H), 2.59 (m, 1H), 2.36 (m, 1H), 1.56 – 1.42 (m, 2H), 1.35 – 1.16 (m, 6H), 0.87 (t, J = 7.1 Hz, 3H); ^{19}F NMR (376 MHz, $CDCl_3$) δ -66.50 (s, 3F); ^{13}C NMR (100 MHz, $CDCl_3$) δ 131.66 (s), 130.24 (s), 129.10 (s), 128.06 (d, J = 1.0 Hz), 125.94 (q, J = 282.0 Hz), 77.67 (s), 58.90 (q, J = 27.0 Hz), 31.20 (s), 30.71 (s), 28.36 (s), 28.18 (s), 22.36 (s), 13.92 (s). HPLC (OJ-H, 5% EtOH in hexanes, 1 mL/min, 210 nm): t_{major} = 7.8 min, t_{minor} = 22.5 min, 90% ee; $^{25}[\alpha]_D$ = -9.4 ° (c = 1.0 in $CHCl_3$); HRMS (ESI+) Calcd for $C_{15}H_{20}F_3NO_2SNa^+$ $(M+Na)^+$: 358.1065, Found: 358.1054.

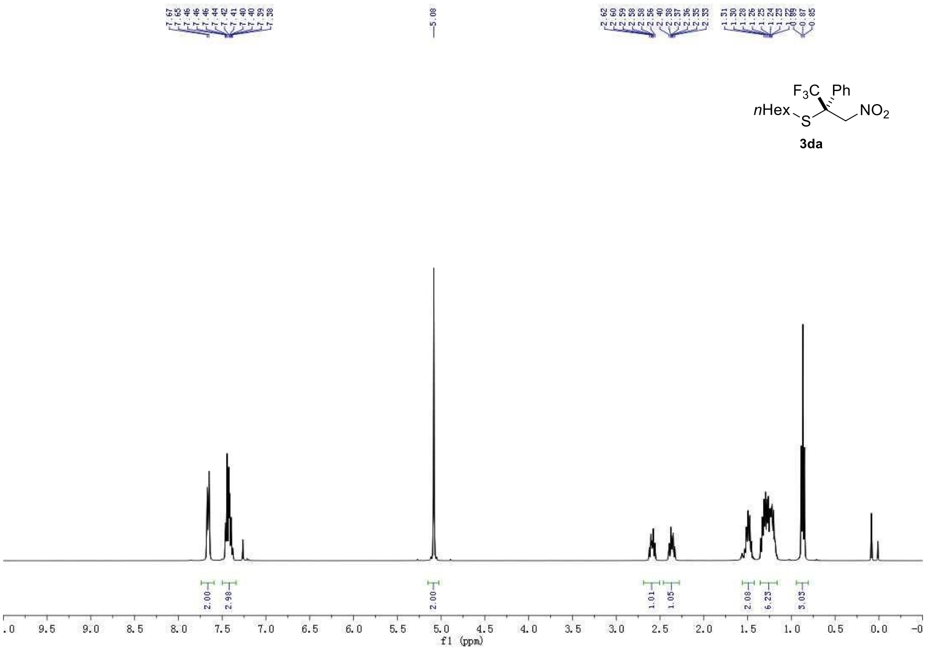

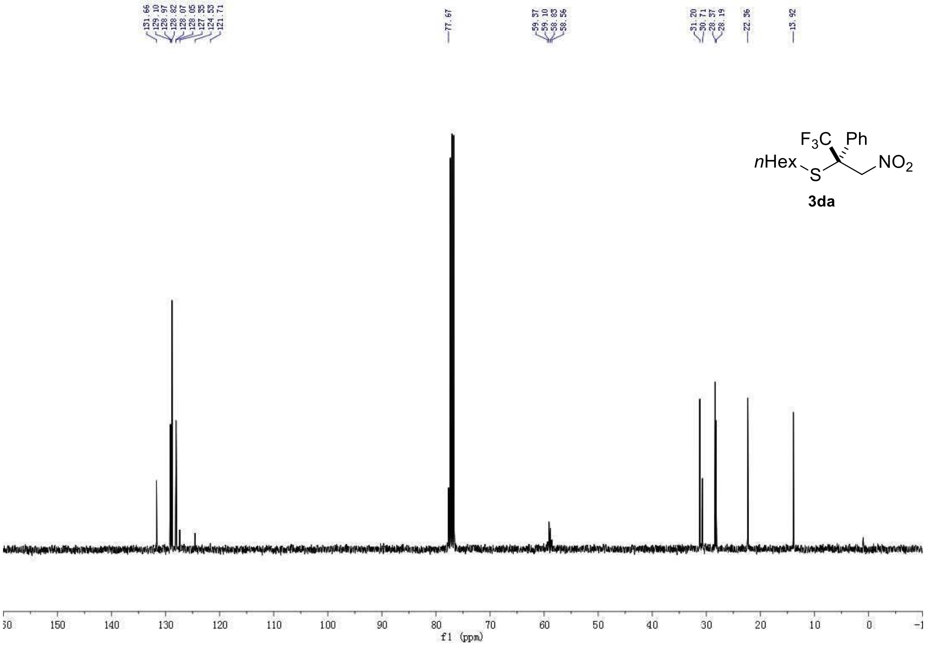

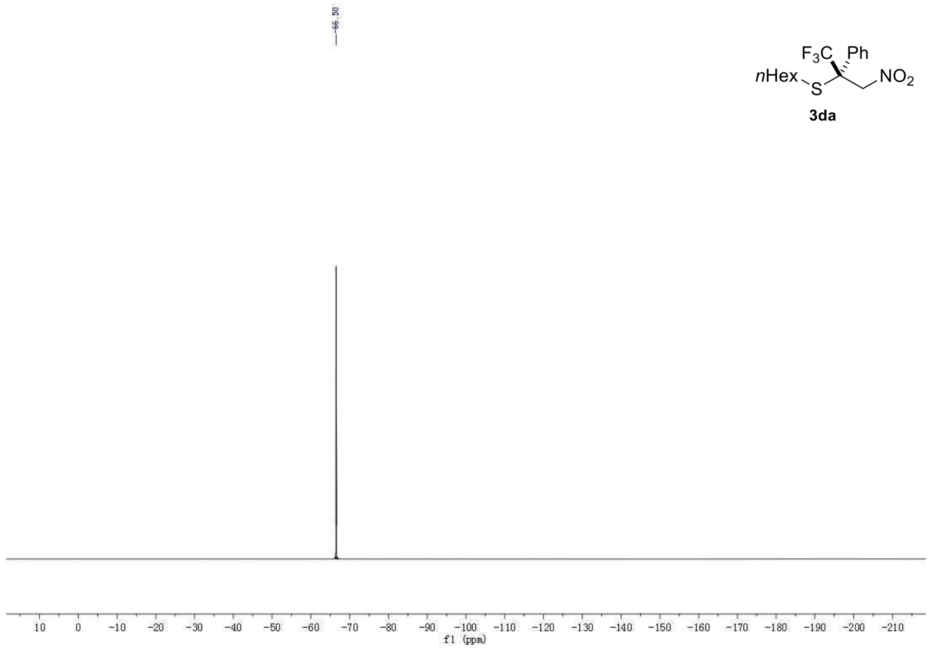

3da

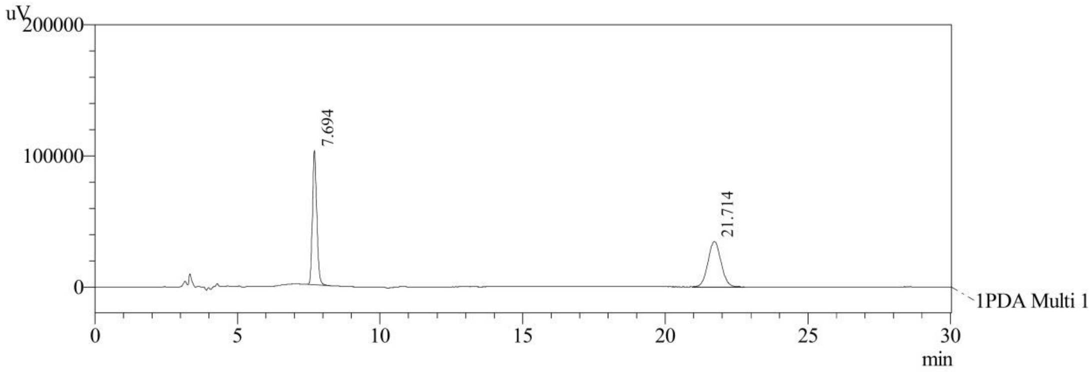

1 PDA Mu

PeakTable

PDA Ch1 210nm 4nm

Peak#	Ret. Time	Area	Height	Area %	Height %
1	7.694	1091627	103050	49.793	74.852
2	21.714	1100693	34621	50.207	25.148
Total		2192320	137671	100.000	100.000

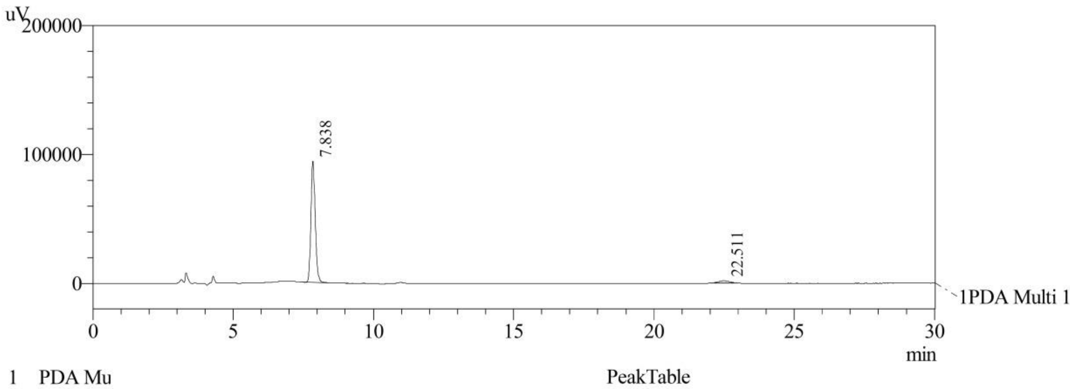

1 PDA Mu

PeakTable

PDA Ch1 210nm 4nm

Peak#	Ret. Time	Area	Height	Area %	Height %
1	7.838	1006852	94186	95.062	98.157
2	22.511	52304	1769	4.938	1.843
Total		1059156	95954	100.000	100.000

(R)-decyl(1,1,1-trifluoro-3-nitro-2-phenylpropan-2-yl)sulfane (3ea)

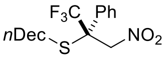

3ea

The title compound was prepared according to the general procedure A and purified by flash column chromatography (50:1 hexanes : EtOAc) to afford **3ea** (38 mg, 97%) as a colorless oil. Analytical data: IR (KBr, cm^{-1}) 2917, 2854, 2377, 1560, 1368, 1218, 1149, 649; ^{1}H NMR (400 MHz, $CDCl_3$) δ 7.70 – 7.61 (m, 2H), 7.48 – 7.36 (m, 3H), 5.08 (s, 2H), 2.58 (dt, *J* = 10.8, 7.3 Hz, 1H), 2.35 (dt, *J* = 10.9, 7.4 Hz, 1H), 1.53 – 1.44 (m, 2H), 1.33 – 1.18 (m, 14H), 0.89 (t, *J* = 6.9 Hz, 3H); ^{19}F NMR (376 MHz, $CDCl_3$) δ -66.50 (s, 3F); ^{13}C NMR (100 MHz, $CDCl_3$) δ 131.65 (s), 129.10 (s), 128.82 (s), 128.06 (d, *J* = 1.5 Hz), 125.94 (q, *J* = 282.0 Hz), 77.67 (s), 58.90 (q, *J* = 27.0 Hz), 31.85 (s), 30.71 (s),29.46 (s), 29.33 (s), 29.25 (s), 29.03 (s), 28.70 (s), 28.21 (s), 22.66 (s), 14.09 (s). HPLC (OJ-H, 5% EtOH in hexanes, 1 mL/min, 210 nm): t_{major} = 6.5 min, t_{minor} = 24.2 min, 98% ee; $^{25}[\alpha]_D$ = -5.3 ° (c = 1.0 in $CHCl_3$); HRMS (ESI+) Calcd for $C_{19}H_{28}F_3NO_2SNa^+$ $(M+Na)^+$: 414.1691, Found: 414.1687.

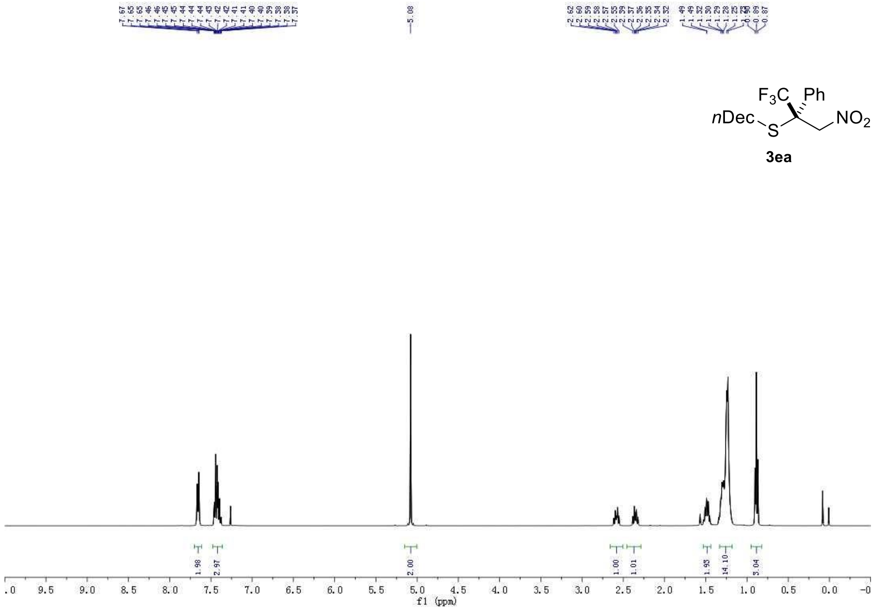

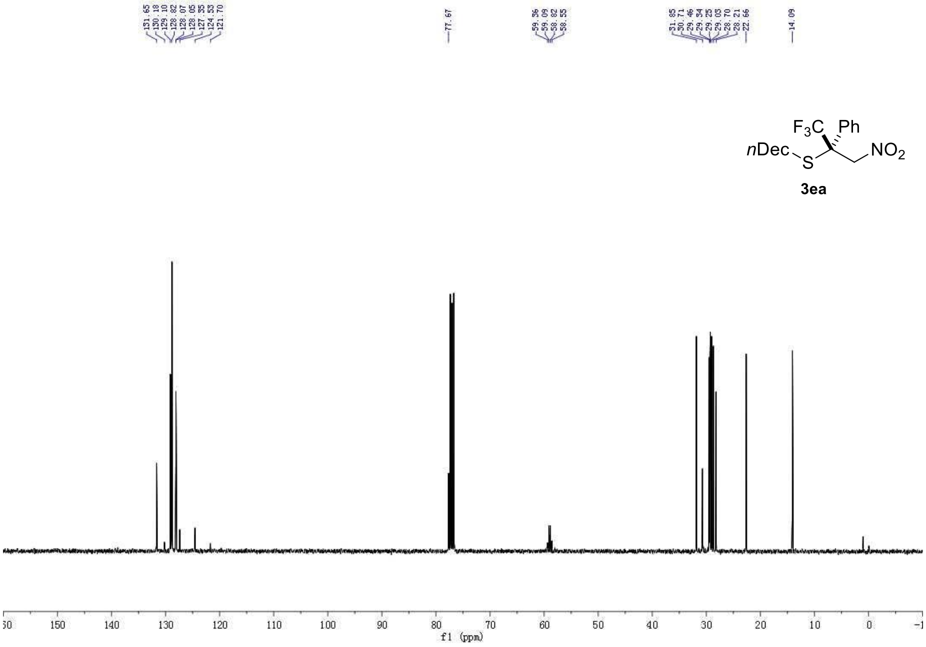

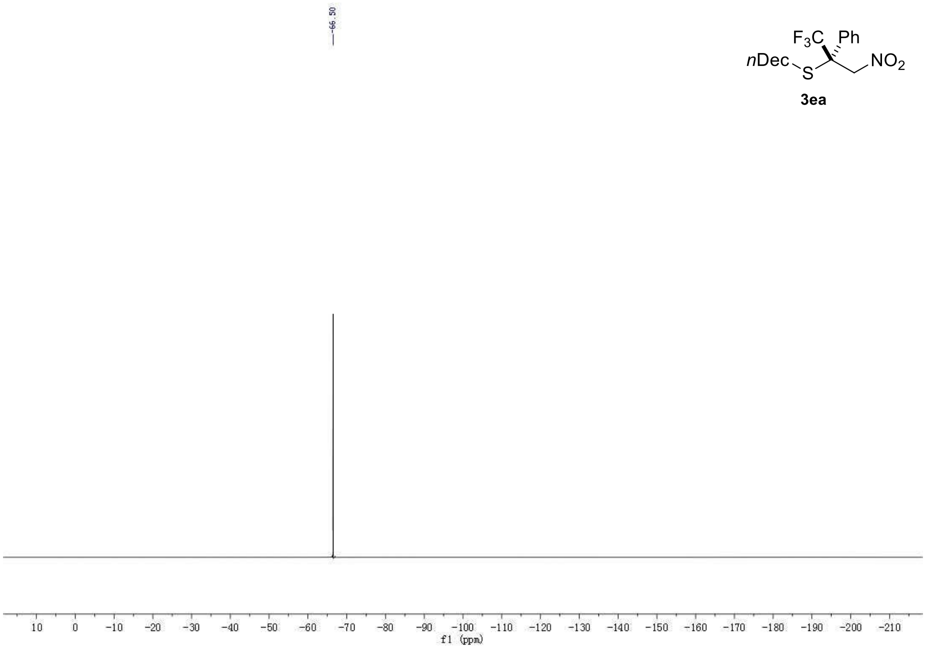

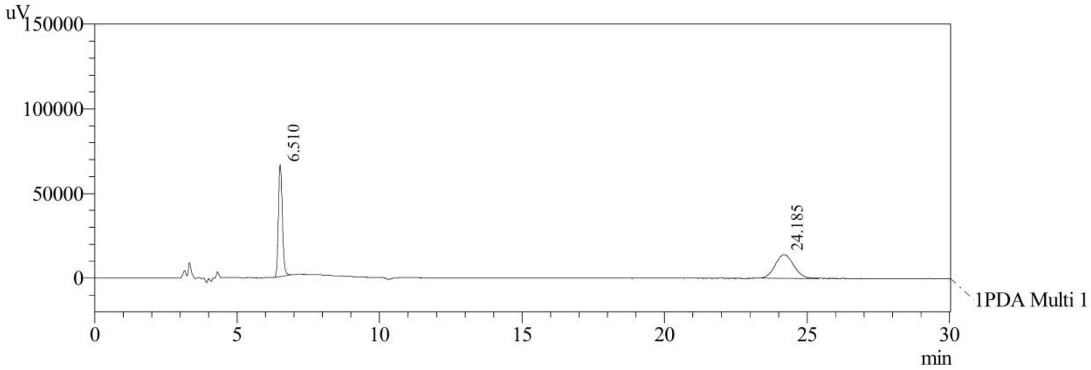

1 PDA Multi 1 / 210nm 4nm

PeakTable

PDA Ch1 210nm 4nm

Peak#	Ret. Time	Area	Height	Area %	Height %
1	6.510	638190	66113	49.999	82.505
2	24.185	638225	14020	50.001	17.495
Total		1276415	80132	100.000	100.000

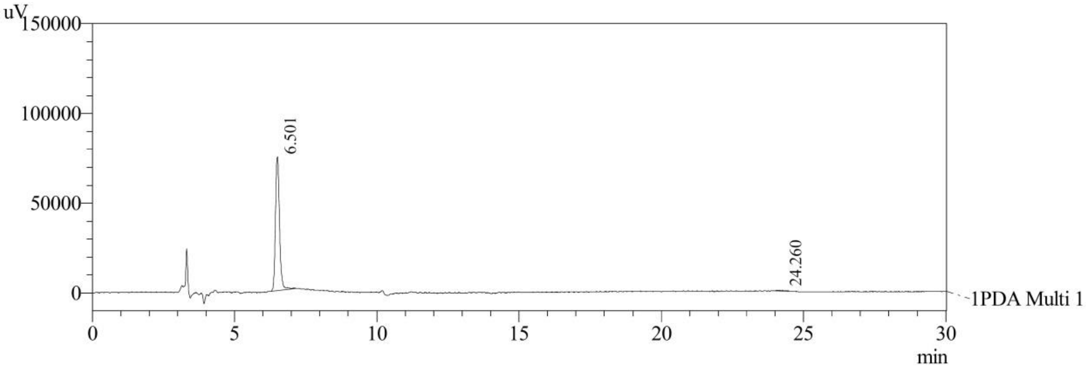

1 PDA Multi 1 / 210nm 4nm

PeakTable

PDA Ch1 210nm 4nm

Peak#	Ret. Time	Area	Height	Area %	Height %
1	6.501	733745	74574	98.996	99.543
2	24.260	7441	343	1.004	0.457
Total		741186	74917	100.000	100.000

(R)-isobutyl(1,1,1-trifluoro-3-nitro-2-phenylpropan-2-yl)sulfane (3fa)

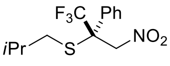

3fa

The title compound was prepared according to the general procedure A and purified by flash column chromatography (50:1 hexanes : EtOAc) to afford **3fa** (29 mg, 96%) as a colorless oil. Analytical data: IR (KBr, cm^{-1}) 3064, 2962, 2375, 1564, 1369, 1218, 1149, 1015, 695; ^{1}H NMR (400 MHz, $CDCl_3$) δ 7.66 (d, *J* = 7.9 Hz, 2H), 7.50 – 7.35 (m, 3H), 5.08 (s, 2H), 2.48 (dd, *J* = 11.0, 6.7 Hz, 1H), 2.24 (dd, *J* = 11.0, 6.9 Hz, 1H), 1.75 (dt, *J* = 13.4, 6.7 Hz, 1H), 0.93 (dd, *J* = 6.6, 4.8 Hz, 6H); ^{19}F NMR (376 MHz, $CDCl_3$) δ -66.40 (s, 3F); ^{13}C NMR (100 MHz, $CDCl_3$) δ 131.68 (s), 129.12 (s), 128.81 (s), 128.12 (d, *J* = 1.9 Hz), 125.94 (q, *J* = 282.0 Hz), 77.74 (s), 58.80 (q, *J* = 27.0 Hz), 39.14 (s), 28.15 (s), 22.01 (s), 21.95 (s). HPLC (OJ-H, 5% EtOH in hexanes, 1 mL/min, 210 nm): t_{major} = 8.9 min, t_{minor} = 25.2 min, 92% ee; $^{25}[\alpha]_D$ = -12.2 ^{o} (c = 1.0 in $CHCl_3$); HRMS (ESI+) Calcd for $C_{13}H_{16}F_3NO_2SNa^+$ $(M+Na)^+$: 330.0752, Found: 330.0745.

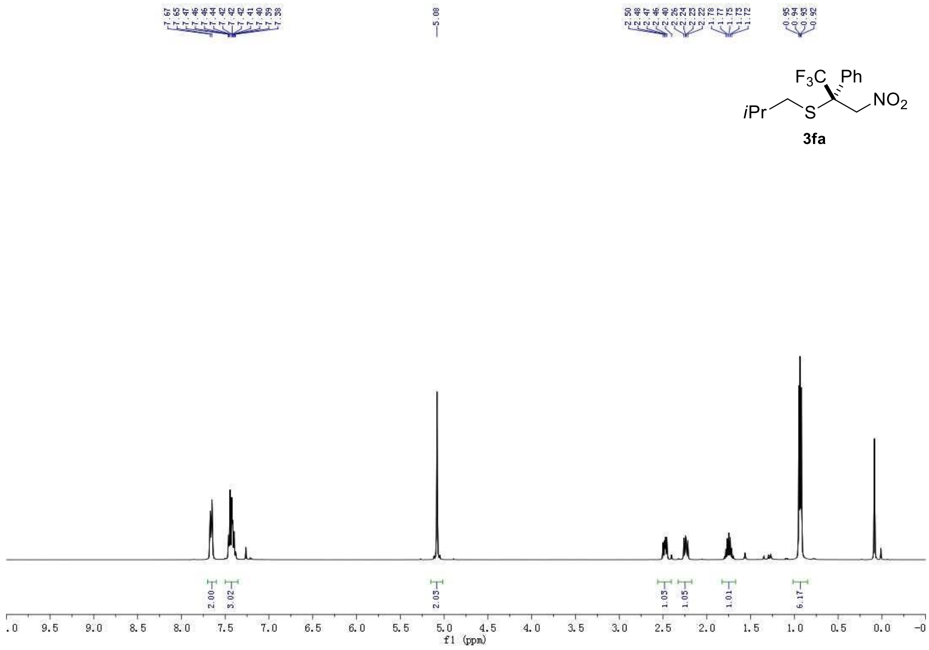

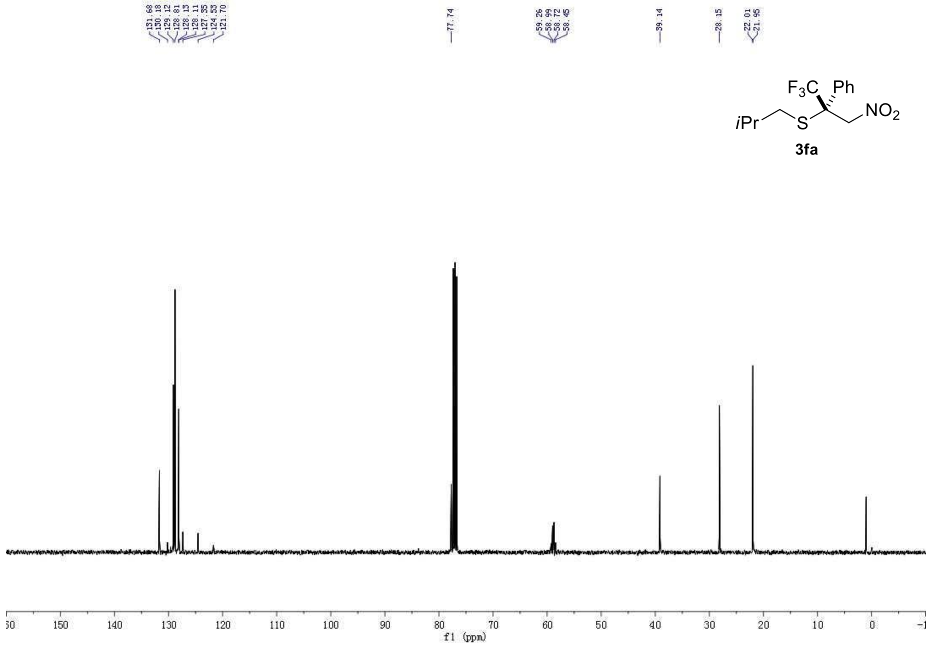

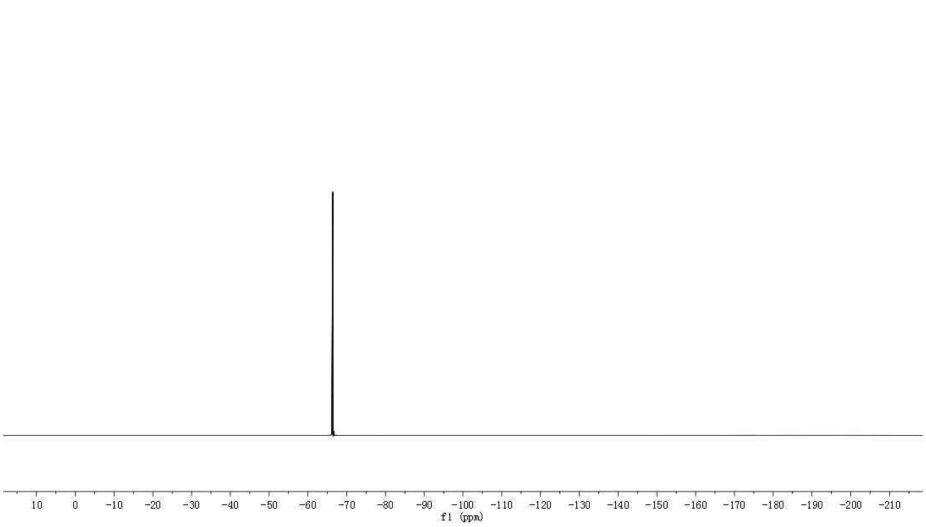

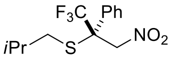

3fa

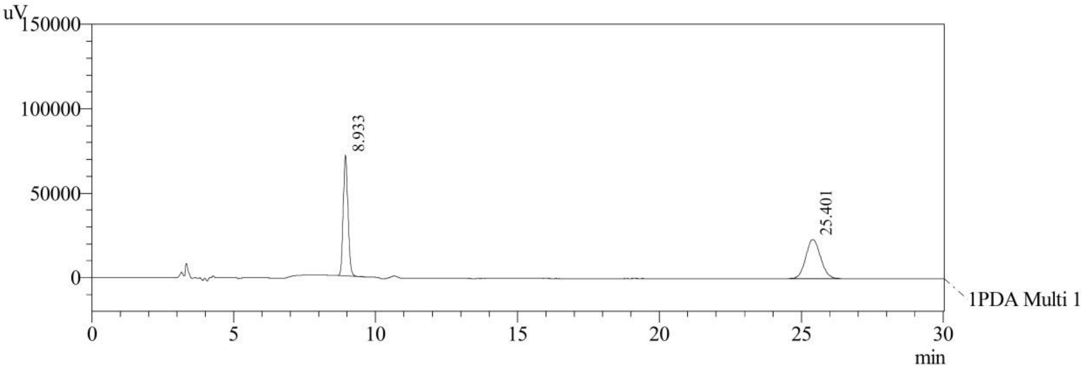

1 PDA Multi 1 / 210nm 4nm

PeakTable

PDA Ch1 210nm 4nm

Peak#	Ret. Time	Area	Height	Area %	Height %
1	8.933	832586	71608	49.908	75.543
2	25.401	835650	23183	50.092	24.457
Total		1668236	94790	100.000	100.000

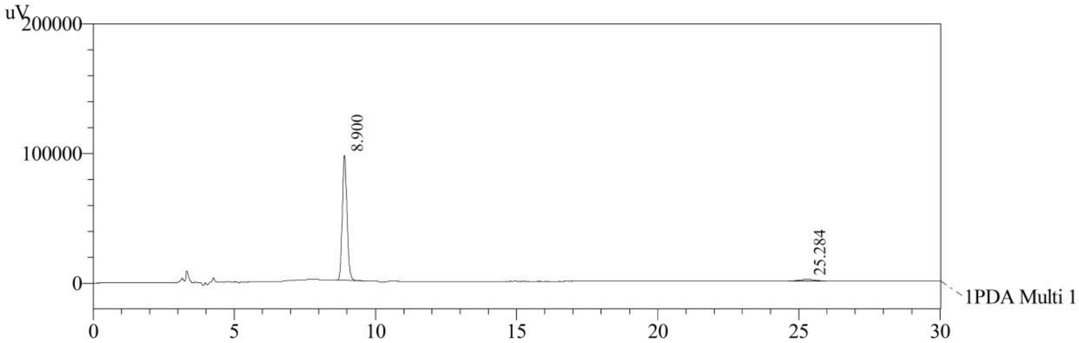

1 PDA M

PeakTable

PDA Ch1 210nm 4nm

Peak#	Ret. Time	Area	Height	Area %	Height %
1	8.900	1125505	96877	96.054	98.635
2	25.284	46239	1340	3.946	1.365
Total		1171744	98217	100.000	100.000

(R)-cyclopentyl(1,1,1-trifluoro-3-nitro-2-phenylpropan-2-yl)sulfane (3ga)

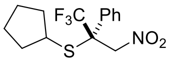

3ga

The title compound was prepared according to the general procedure A and purified by flash column chromatography (50:1 hexanes : EtOAc) to afford **3ga** (29 mg, 90%) as a colorless oil. Analytical data: IR (KBr, cm^{-1}) 2961, 1564, 1450, 1368, 1219, 1149, 1014, 695; ^1H NMR (400 MHz, $CDCl_3$) δ 7.66 (d, *J* = 8.2 Hz, 2H), 7.49 – 7.34 (m, 3H), 5.18 – 5.01 (m, 2H), 2.91 (m, 1H), 1.96 (ddd, *J* = 10.7, 7.8, 2.7 Hz, 1H), 1.82 – 1.70 (m, 1H), 1.70 – 1.31 (m, 6H); ^{19}F NMR (376 MHz, $CDCl_3$) δ -65.91 (s, 3F); ^{13}C NMR (100 MHz, $CDCl_3$) δ 132.04 (s), 129.07 (s), 128.75 (s), 128.09 (d, *J* = 2.0 Hz), 125.70 (q, *J* = 283.0 Hz), 77.98 (s), 59.50 (q, *J* = 27.0 Hz), 44.02 (s), 34.99 (s), 34.44 (s), 24.75 (s), 24.68 (s). HPLC (AD-H, 5% EtOH in hexanes, 1 mL/min, 210 nm): t_{major} = 6.5 min, t_{minor} = 8.5 min, 992% ee; $^{25}[\alpha]_D$ = -14.3 ° (c = 1.0 in $CHCl_3$). HRMS (ESI+) Calcd for $C_{14}H_{16}F_3NO_2SNa^+$ (M+Na)$^+$: 342.0752, Found: 342.0746.

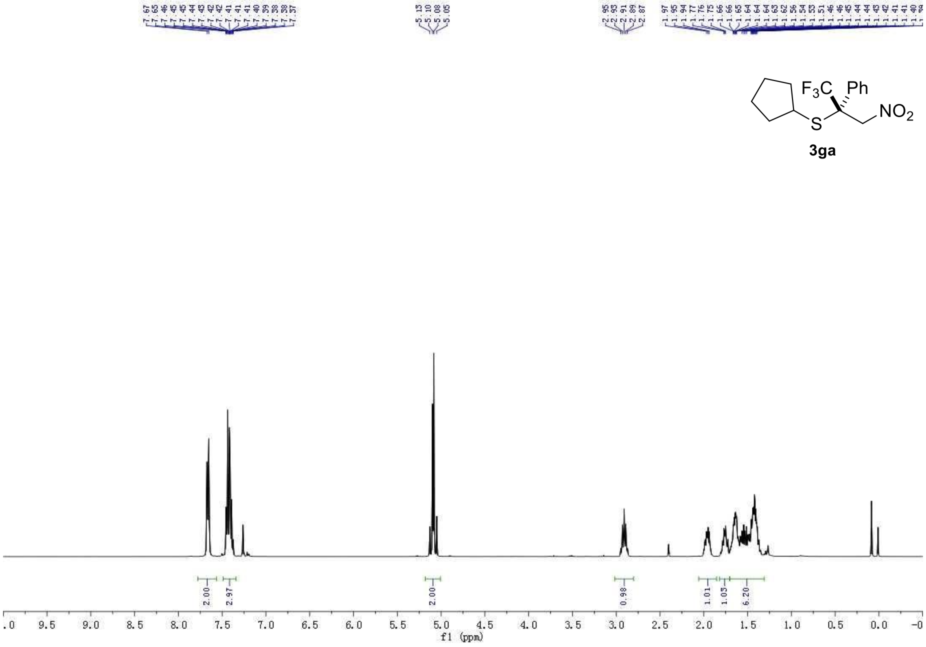

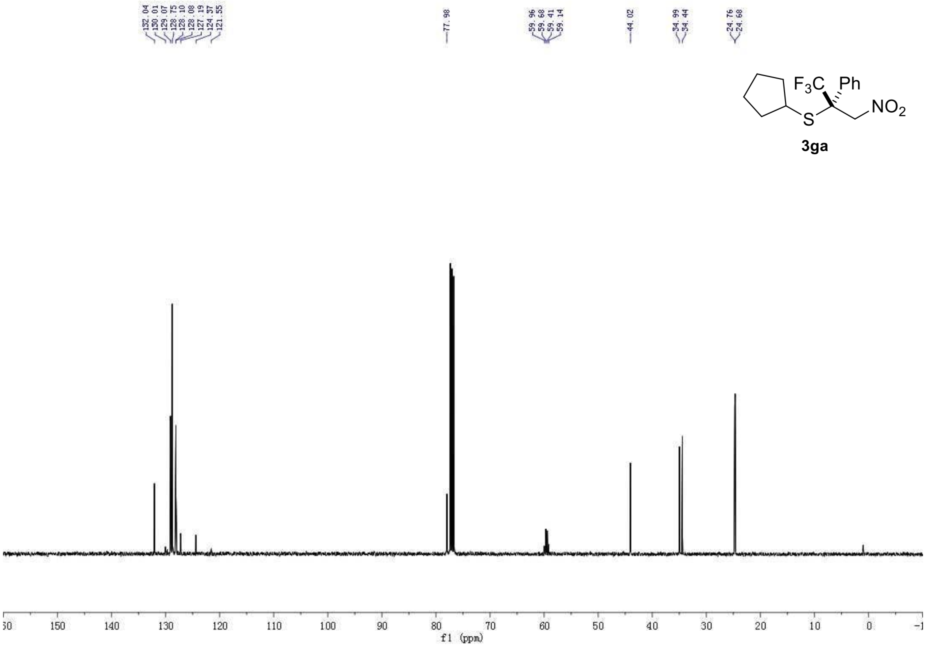

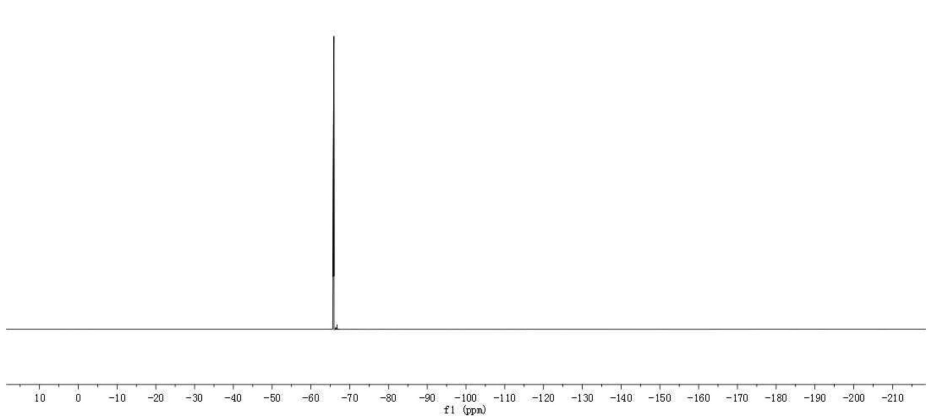

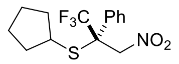

3ga

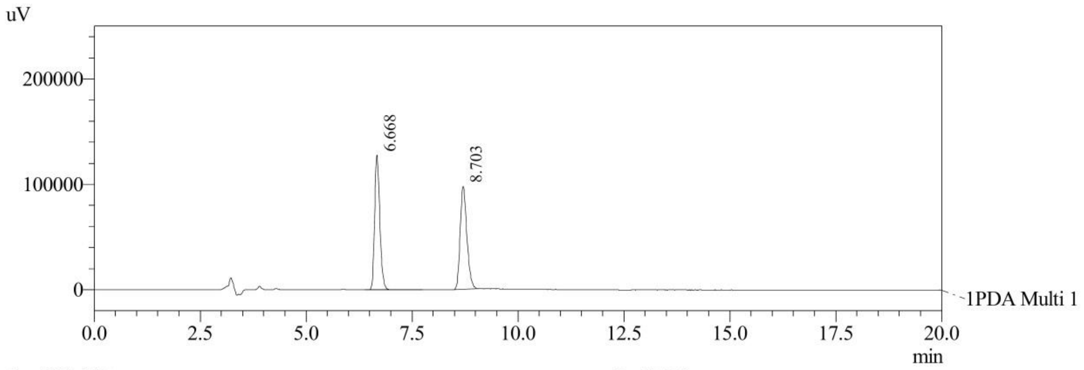

1 PDA Mu

PeakTable

PDA Ch1 210nm 4nm

Peak#	Ret. Time	Area	Height	Area %	Height %
1	6.668	1088269	128056	50.115	56.788
2	8.703	1083275	97444	49.885	43.212
Total		2171544	225500	100.000	100.000

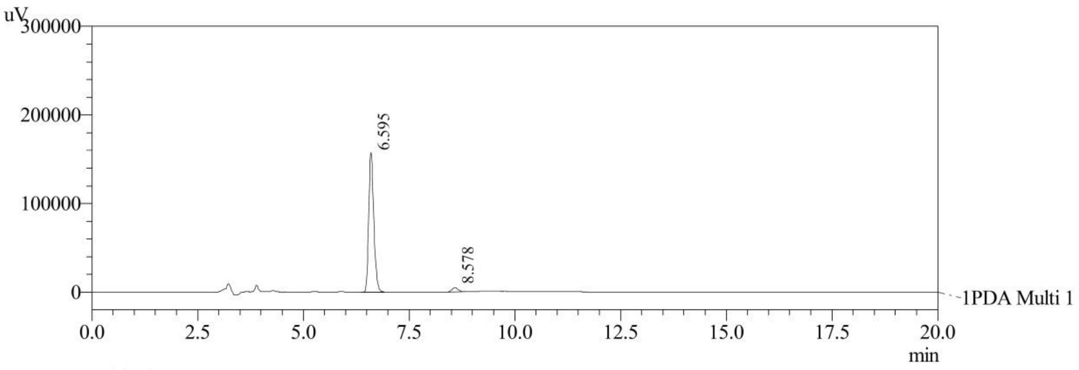

1 PDA Multi 1 / 210nm 4nm

PeakTable

PDA Ch1 210nm 4nm

Peak#	Ret. Time	Area	Height	Area %	Height %
1	6.595	1313706	157892	96.189	96.920
2	8.578	52056	5018	3.811	3.080
Total		1365761	162910	100.000	100.000

(R)-cyclohexyl(1,1,1-trifluoro-3-nitro-2-phenylpropan-2-yl)sulfane (3ha)

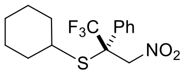

3ha

The title compound was prepared according to the general procedure A and purified by flash column chromatography (50:1 hexanes : EtOAc) to afford **3ha** (29 mg, 89%) as a colorless oil. Analytical data: IR (KBr, cm^{-1}t) 2932, 2371, 1560, 1448, 1368, 1219, 1148, 695; ^{1}H NMR (400 MHz, $CDCl_3$) δ 7.71 – 7.59 (m, 2H), 7.50 – 7.33 (m, 3H), 5.06 (q, *J* = 12.8 Hz, 2H), 2.63 – 2.45 (m, 1H), 1.88 (dd, *J* = 9.3, 3.8 Hz, 1H), 1.71 – 1.57 (m, 3H), 1.49 – 1.36 (m, 2H), 1.33 – 1.09 (m, 4H); ^{19}F NMR (376 MHz, $CDCl_3$) δ -66.01 (s, 3F); ^{13}C NMR (100 MHz, $CDCl_3$) δ 131.96 (s), 129.04 (s), 128.66 (s), 128.06 (d, *J* = 2.1 Hz), 125.80 (q, *J* = 283.0 Hz), 78.11 (s), 59.50 (q, *J* = 27.0 Hz), 44.46 (s), 34.90 (s), 34.67 (s), 26.07 (s), 25.15 (s). HPLC (AD-H, 5% EtOH in hexanes, 1 mL/min, 210 nm): t_{major} = 6.1 min, t_{minor} = 14.0 min, 96% ee; $^{25}[\alpha]_D$ = -23.8 ^{o} (c = 1.0 in $CHCl_3$); HRMS (ESI+) Calcd for $C_{15}H_{18}F_3NO_2SNa^+$ $(M+Na)^+$: 356.0908, Found: 356.0903.

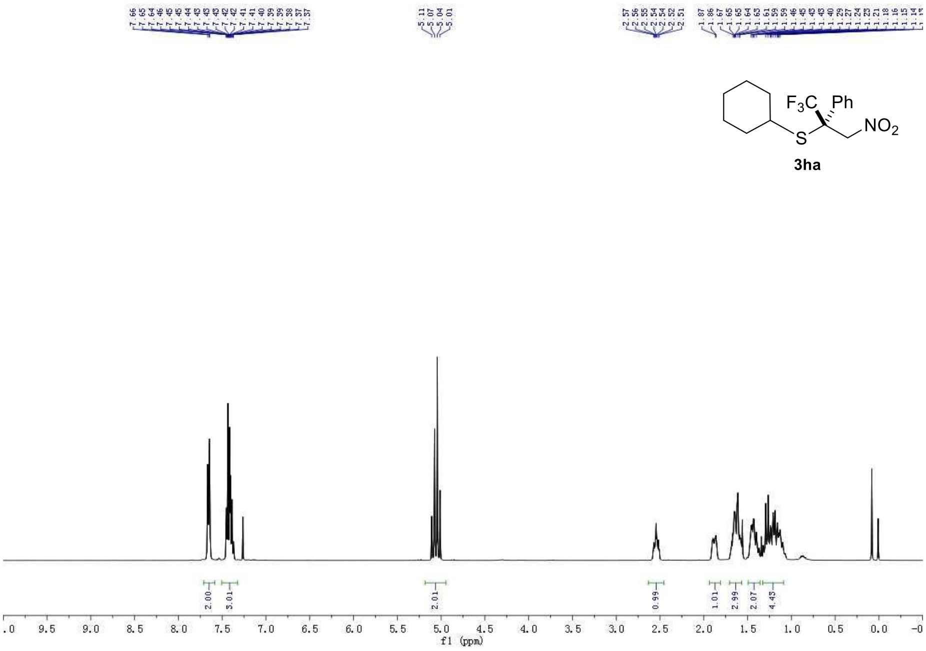

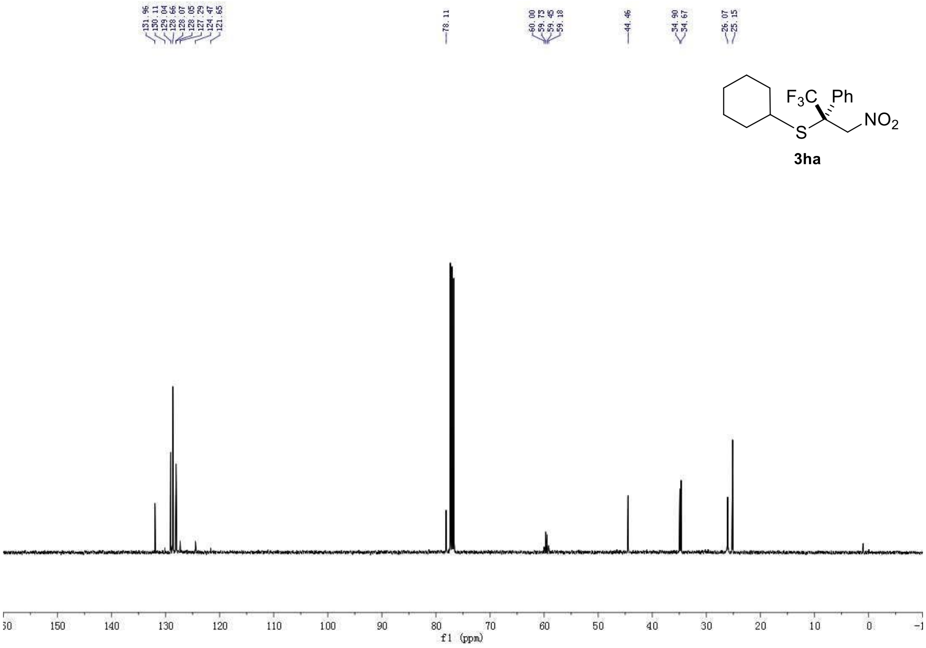

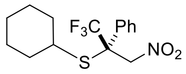

3ha

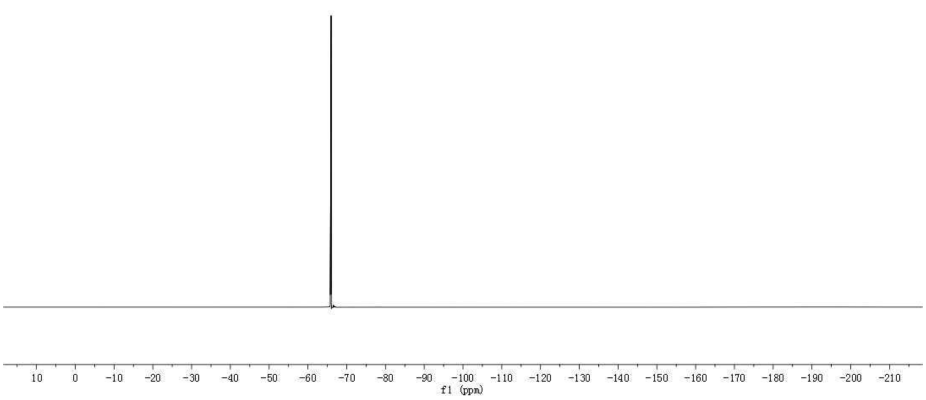

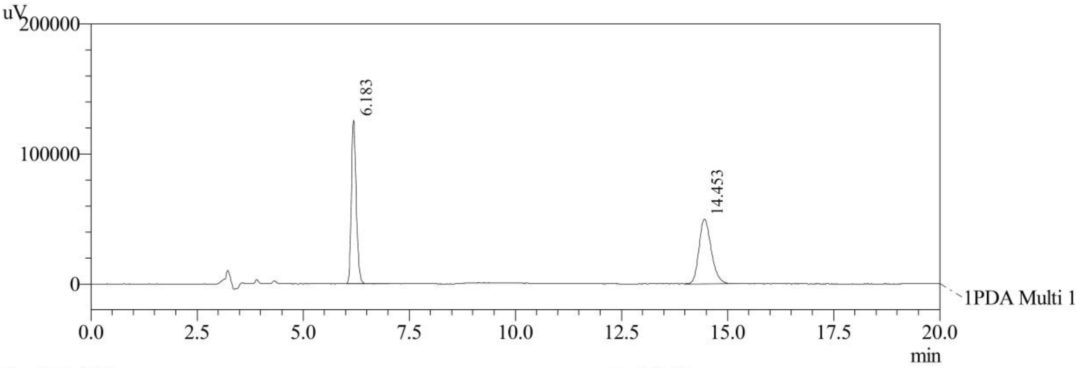

1 PDA Mu

PeakTable

PDA Ch1 210nm 4nm

Peak#	Ret. Time	Area	Height	Area %	Height %
1	6.183	1000224	125971	49.798	71.621
2	14.453	1008320	49915	50.202	28.379
Total		2008544	175886	100.000	100.000

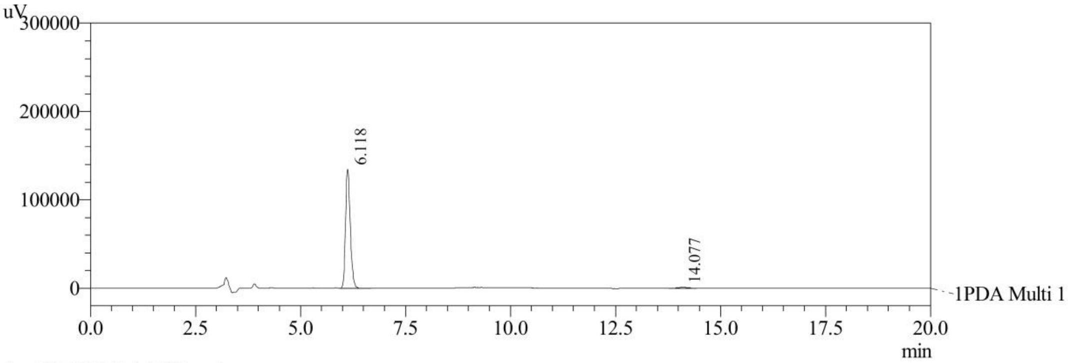

1 PDA Multi 1 / 210nm 4nm

PeakTable

PDA Ch1 210nm 4nm

Peak#	Ret. Time	Area	Height	Area %	Height %
1	6.118	1059493	134745	97.839	99.065
2	14.077	23404	1272	2.161	0.935
Total		1082897	136017	100.000	100.000

(R)-isopropyl(1,1,1-trifluoro-3-nitro-2-phenylpropan-2-yl)sulfane (3ia)

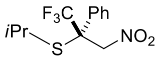

3ia

The title compound was prepared according to the general procedure A and purified by flash column chromatography (50:1 hexanes : EtOAc) to afford **3ia** (24 mg, 80%) as a colorless oil. Analytical data: IR (KBr, cm^{-1}) 2970, 2308, 1564, 1449, 1369, 1265, 1219, 1150, 697; ^{1}H NMR (400 MHz, $CDCl_3$) δ 7.66 (dd, *J* = 7.4, 1.0 Hz, 2H), 7.52 – 7.35 (m, 3H), 5.14 – 4.95 (m, 2H), 2.79 (m, 1H), 1.25 (d, *J* = 7.2 Hz, 3H), 1.09 (d, *J* = 7.2 Hz, 3H); ^{19}F NMR (376 MHz, $CDCl_3$) δ -65.96 (s, 3F); ^{13}C NMR (100 MHz, $CDCl_3$) δ 131.85 (s), 129.09 (s), 128.75 (s), 128.08 (d, *J* = 3.0 Hz), 125.85 (q, *J* = 282.0 Hz), 78.19 (s), 59.70 (q, *J* = 27.0 Hz), 36.68 (s), 24.93 (s), 24.55 (s). HPLC (OJ-H, 5% EtOH in hexanes, 1 mL/min, 210 nm): t_{major} = 9.8 min, t_{minor} = 22.4 min, 88% ee; $^{25}[\alpha]_D$ = -14.7 º (c = 1.0 in $CHCl_3$); HRMS (ESI+) Calcd for $C_{12}H_{14}F_3NO_2SNa^+$ $(M+Na)^+$: 316.0595, Found: 316.0593.

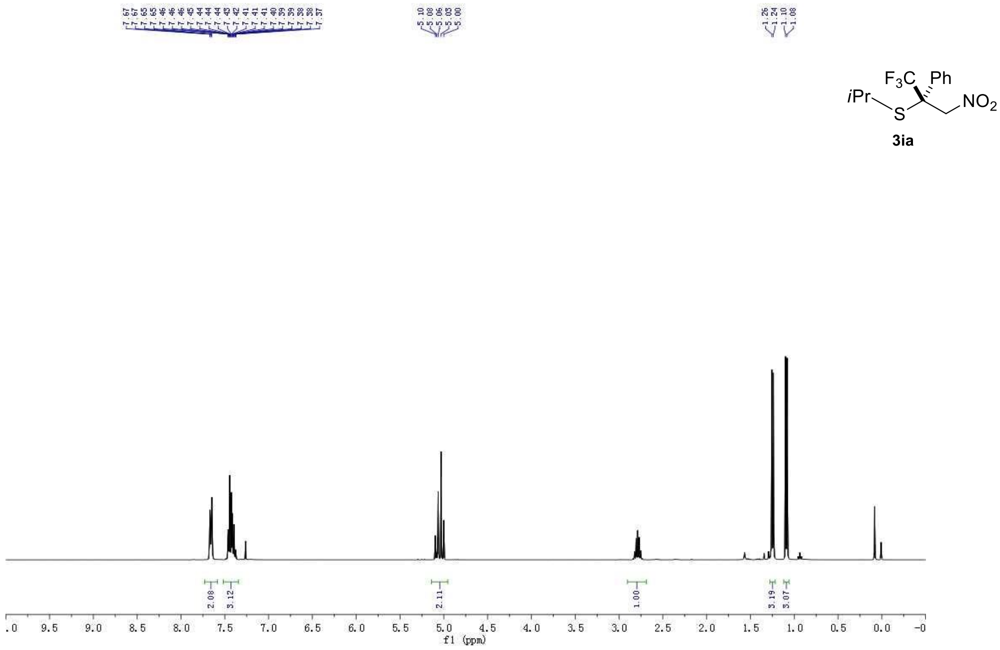

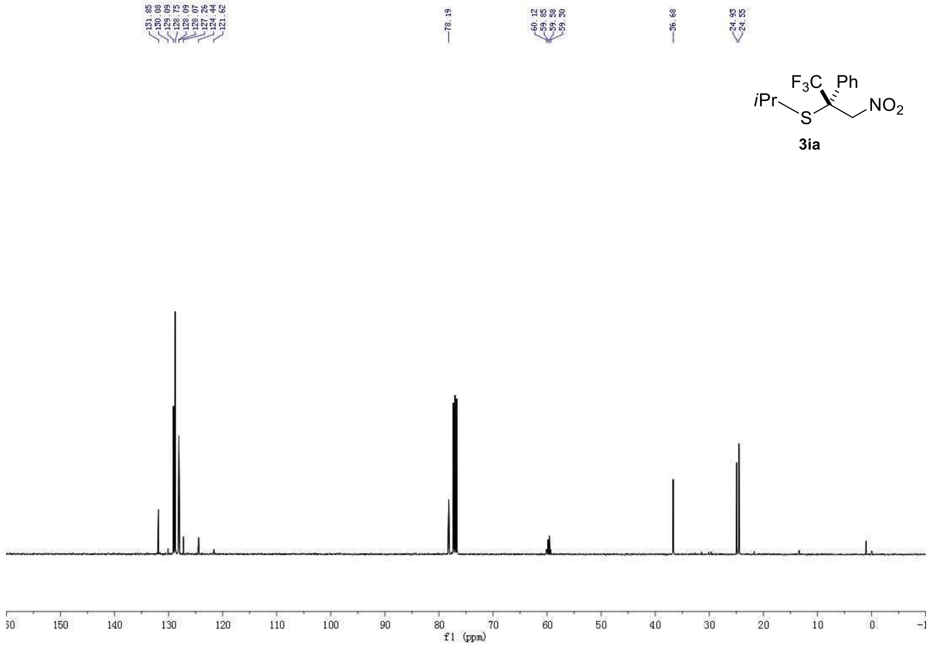

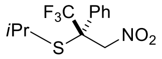

3ia

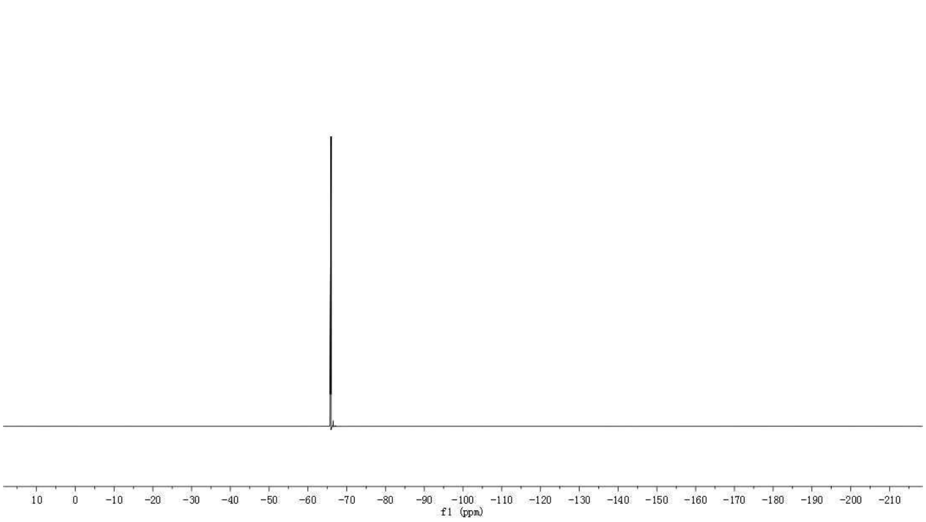

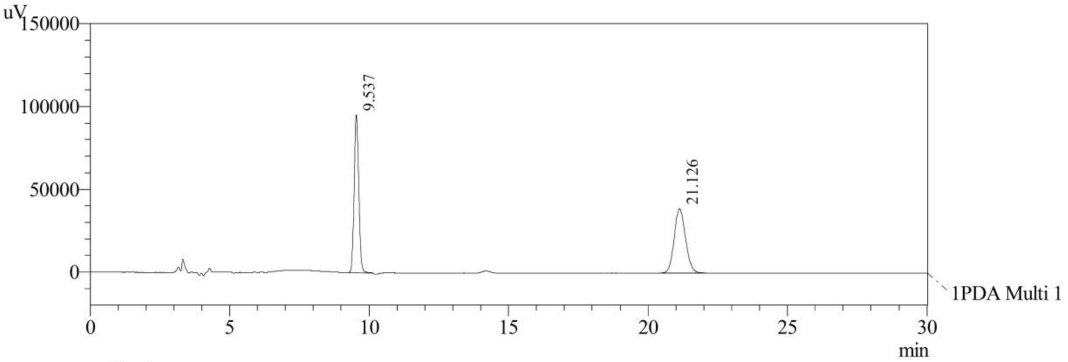

1 PDA Multi 1 / 210nm 4nm

PeakTable

PDA Ch1 210nm 4nm

Peak#	Ret. Time	Area	Height	Area %	Height %
1	9.537	1095538	95437	50.011	71.039
2	21.126	1095044	38908	49.989	28.961
Total		2190582	134345	100.000	100.000

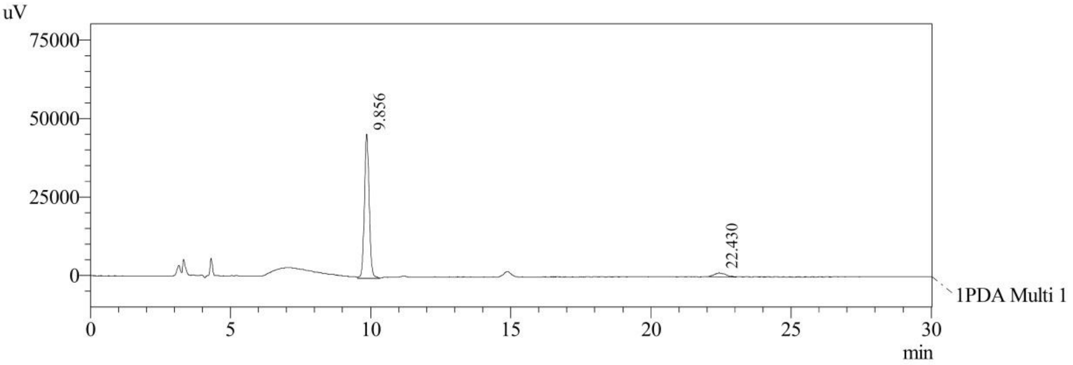

1 PDA Multi 1 / 210nm 4nm

PeakTable

PDA Ch1 210nm 4nm

Peak#	Ret. Time	Area	Height	Area %	Height %
1	9.856	541235	45685	93.811	97.324
2	22.430	35706	1256	6.189	2.676
Total		576941	46942	100.000	100.000

(R)-2-(((1,1,1-trifluoro-3-nitro-2-phenylpropan-2-yl)thio)methyl)furan (3ka)

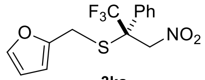

3ka

The title compound was prepared according to the general procedure A and purified by flash column chromatography (50:1 hexanes : EtOAc) to afford **3ka** (31 mg, 95%) as a colorless oil. Analytical data: IR (KBr, cm^{-1}) 2921, 2308, 1559, 1369, 1216, 1149, 1012, 743, 696; ^{1}H NMR (400 MHz, $CDCl_3$) δ 7.69 (d, J = 8.0 Hz, 2H), 7.52 – 7.39 (m, 3H), 7.39 – 7.31 (m, 1H), 6.29 (dd, J = 3.1, 1.9 Hz, 1H), 6.13 (d, J = 3.1 Hz, 1H), 5.16 – 5.02 (m, 2H), 3.88 (d, J = 13.4 Hz, 1H), 3.59 (d, J = 13.4 Hz, 1H); ^{19}F NMR (376 MHz, $CDCl_3$) δ -66.14 (s, 3F); ^{13}C NMR (100 MHz, $CDCl_3$) δ 148.36 (s), 142.79 (s), 131.00 (s), 129.39 (s), 129.07 (s), 128.07 (d, J = 1.6 Hz), 125.87 (q, J = 283.0 Hz), 110.74 (s), 109.02 (s), 77.64 (s), 59.55 (q, J = 28.0 Hz), 28.07 (s), 28.05 (s). HPLC (AD-H, 5% EtOH in hexanes, 1 mL/min, 210 nm): t_{major} = 14.2 min, t_{minor} = 16.0 min, 80% ee; $^{25}[\alpha]_D$ = +18.6 ° (c = 1.0 in $CHCl_3$); HRMS (ESI+) Calcd for $C_{14}H_{12}F_3NO_3SNa^+$ $(M+Na)^+$: 354.0388, Found: 354.0383.

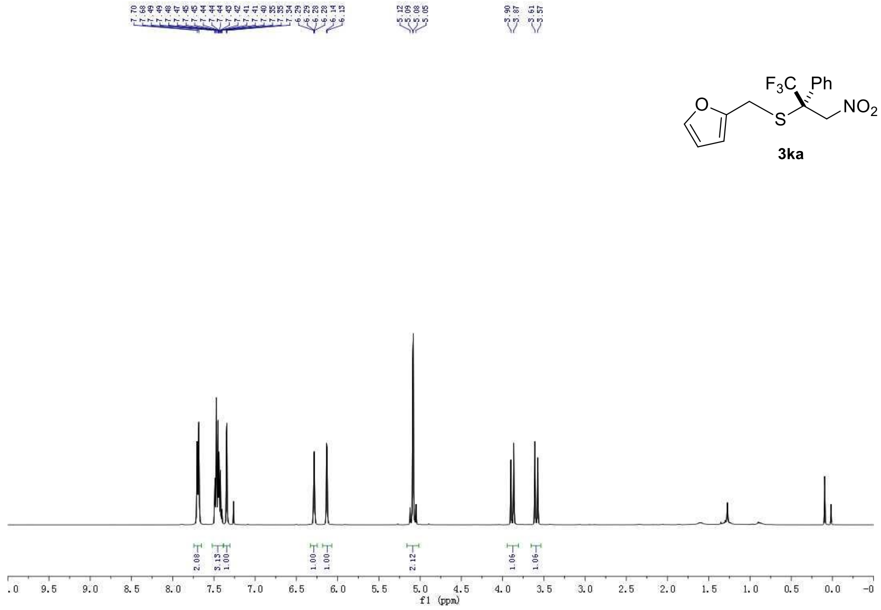

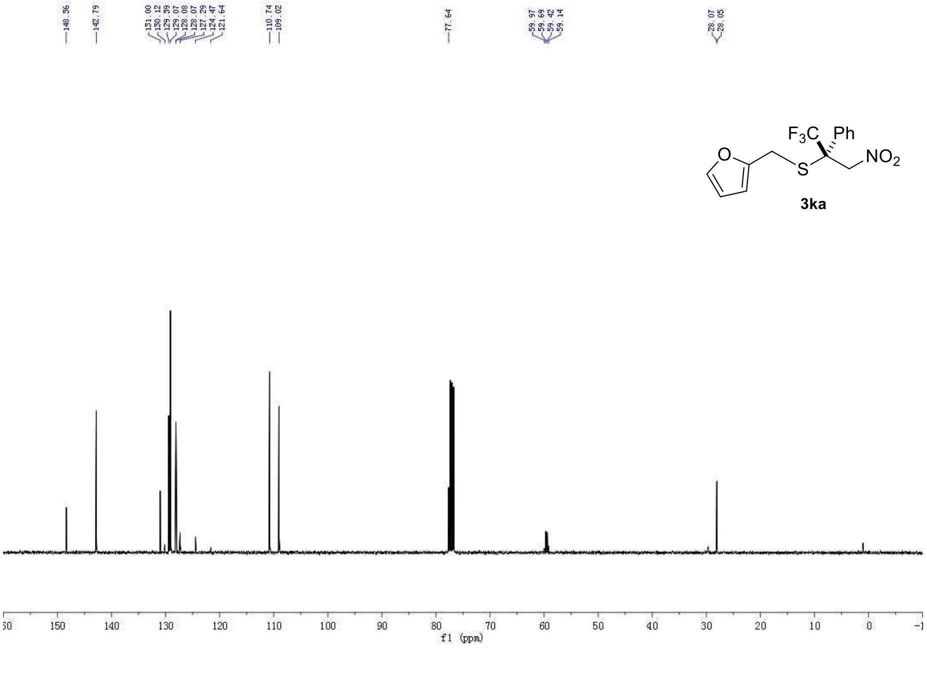

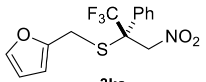

3ka

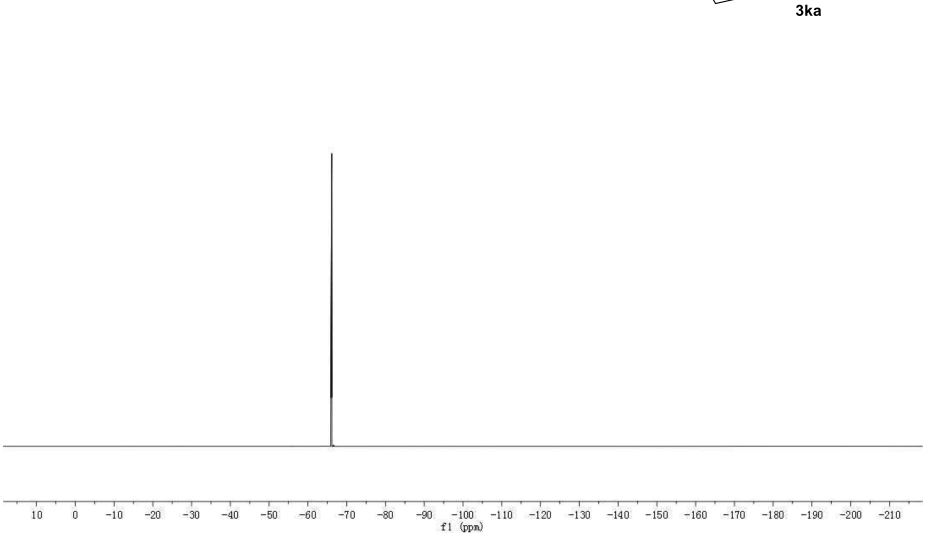

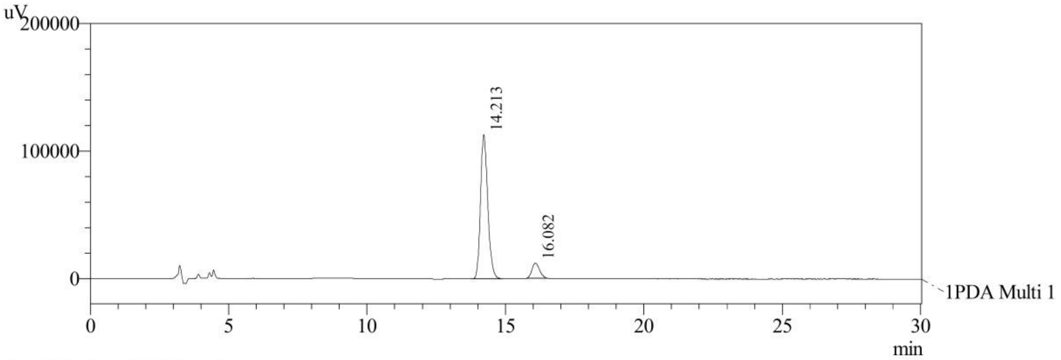

1 PDA Multi 1 / 210nm 4nm

PeakTable

PDA Ch1 210nm 4nm

Peak#	Ret. Time	Area	Height	Area %	Height %
1	14.213	2056566	113205	89.955	90.385
2	16.082	229643	12043	10.045	9.615
Total		2286208	125248	100.000	100.000

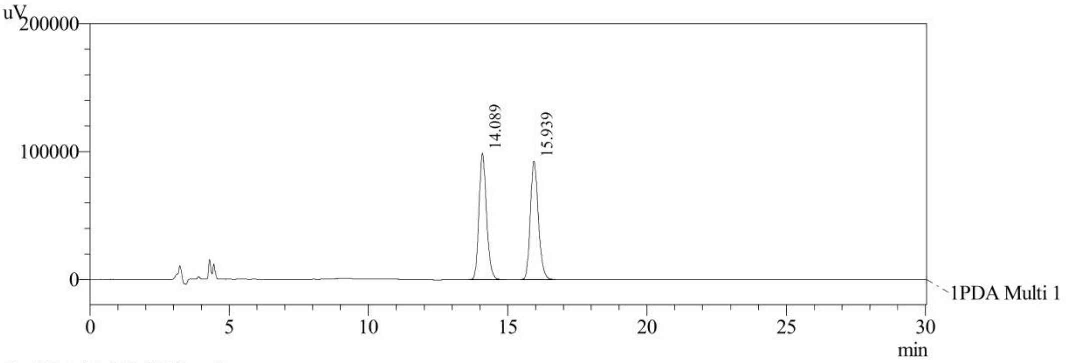

1 PDA Multi 1 / 210nm 4nm

PeakTable

PDA Ch1 210nm 4nm

Peak#	Ret. Time	Area	Height	Area %	Height %
1	14.089	1857634	99007	50.013	51.596
2	15.939	1856671	92881	49.987	48.404
Total		3714305	191888	100.000	100.000

(R)-benzyl(1,1,1-trifluoro-3-nitro-2-phenylpropan-2-yl)sulfane (3la)

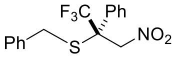

3la

The title compound was prepared according to the general procedure A and purified by flash column chromatography (50:1 hexanes : EtOAc) to afford **3la** (32 mg, 93%) as a colorless oil. Analytical data: IR (KBr, cm^{-1}) 3032, 2310, 1559, 1495, 1369, 1218, 1151, 695; ^1H NMR (400 MHz, $CDCl_3$) δ 7.73 (d, *J* = 8.3 Hz, 2H), 7.53 – 7.39 (m, 3H), 7.36 – 7.15 (m, 5H), 5.13 (s, 2H), 3.87 (d, *J* = 11.0 Hz, 1H), 3.56 (d, *J* = 11.0 Hz, 1H); ^{19}F NMR (376 MHz, $CDCl_3$) δ -66.31 (s, 3F); ^{13}C NMR (100 MHz, $CDCl_3$) δ 134.73 (s), 131.35 (s), 129.38 (s), 129.34 (s), 129.00 (s), 128.78 (s), 128.07 (d, *J* = 1.0 Hz), 127.91 (s), 125.95 (q, *J* = 283.0 Hz), 77.45 (s), 59.66 (q, *J* = 28.0 Hz), 35.71 (s). HPLC (AD-H, 5% EtOH in hexanes, 1 mL/min, 210 nm): t_{major} = 12.2 min, t_{minor} = 9.1 min, 85% ee; $^{25}[\alpha]_D$ = +33.6 ° (c = 0.5 in $CHCl_3$); HRMS (ESI+) Calcd for $C_{16}H_{14}F_3NO_2SNa^+$ $(M+Na)^+$: 364.0595, Found: 364.0589.

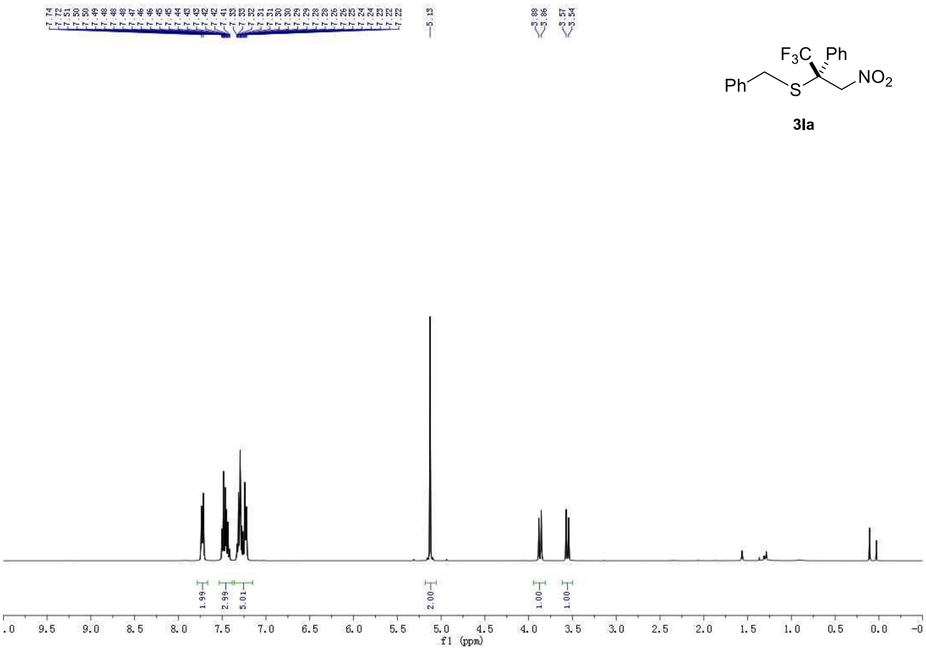

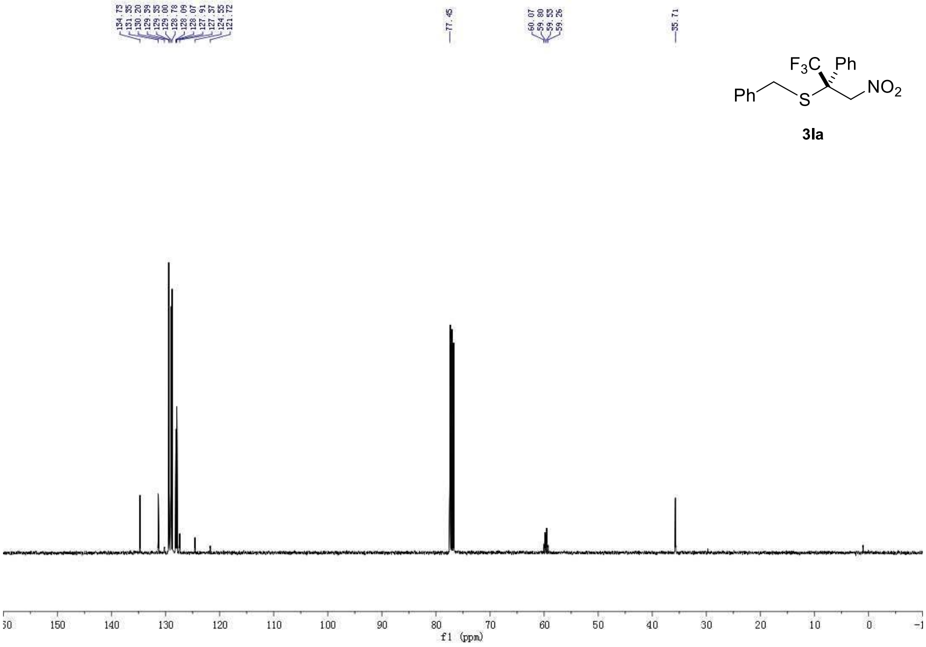

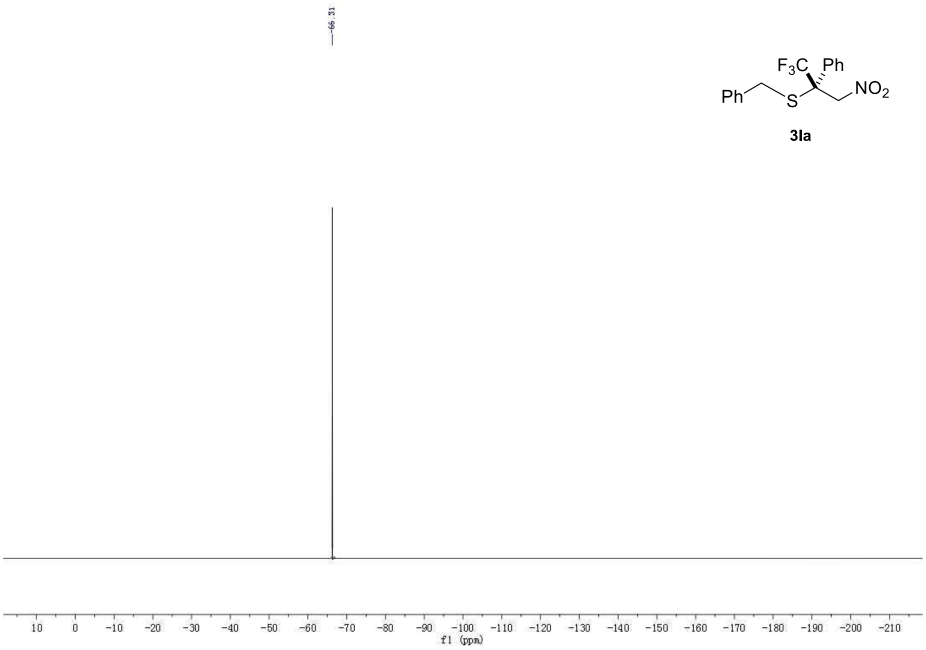

3la

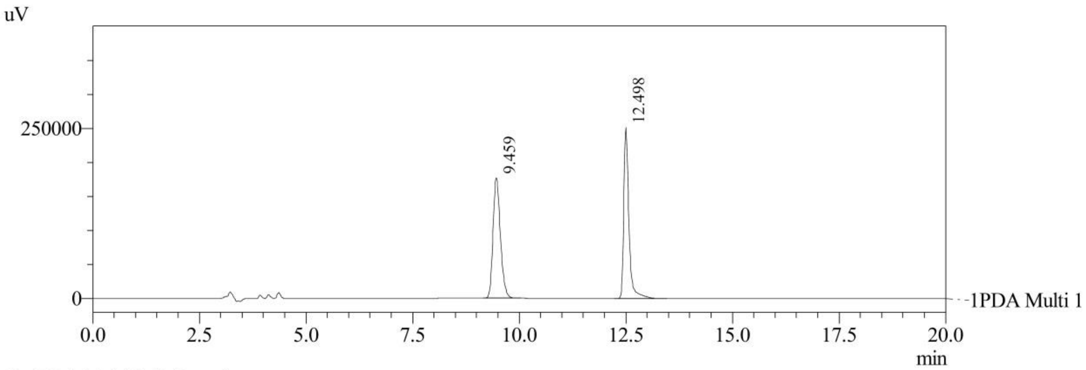

1 PDA Multi 1 / 210nm 4nm

PeakTable

PDA Ch1 210nm 4nm

Peak#	Ret. Time	Area	Height	Area %	Height %
1	9.459	2129716	176557	50.123	41.257
2	12.498	2119272	251386	49.877	58.743
Total		4248987	427943	100.000	100.000

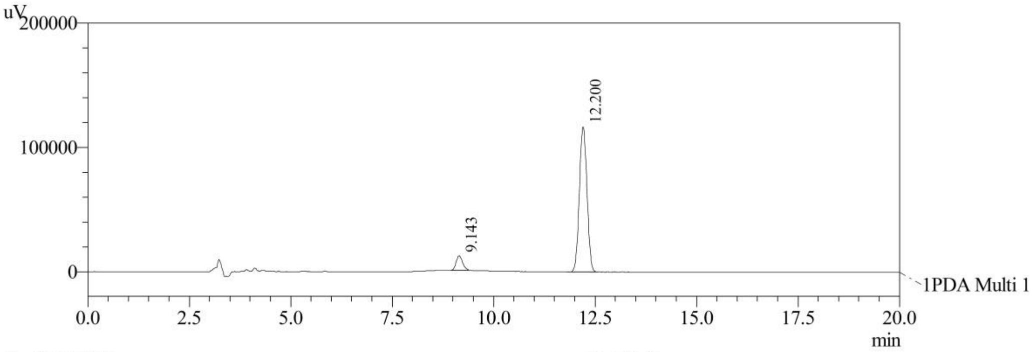

1 PDA Mul

PeakTable

PDA Ch1 210nm 4nm

Peak#	Ret. Time	Area	Height	Area %	Height %
1	9.143	126477	11504	7.612	8.957
2	12.200	1535098	116936	92.388	91.043
Total		1661574	128440	100.000	100.000

(R)-(3-fluorobenzyl)(1,1,1-trifluoro-3-nitro-2-phenylpropan-2-yl)sulfane (3ma)

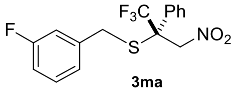

3ma

The title compound was prepared according to the general procedure A and purified by column chromatography (50:1 hexanes : EtOAc) to afford **3ma** (34 mg, 95%) as a colorless oil. Analytical data: IR (KBr, cm^{-1}) 2921, 2321, 1560, 1490, 1448, 1369, 1218, 1152, 946, 742, 696; ^1H NMR (400 MHz, $CDCl_3$) δ 7.76 – 7.65 (m, 2H), 7.54 – 7.38 (m, 3H), 7.34 – 7.19 (m, 1H), 7.06 – 6.87 (m, 3H), 5.12 (s, 2H), 3.83 (d, *J* = 11.3 Hz, 1H), 3.52 (d, *J* = 11.3 Hz, 1H); ^{19}F NMR (376 MHz, $CDCl_3$) δ -66.38 (s, 3F), -112.49 (s, 1F); ^{13}C NMR (100 MHz, $CDCl_3$) δ 162.75 (d, *J* = 245.0 Hz), 137.26 (d, *J* = 7.7 Hz), 131.13 (s),130.18 (d, *J* = 8.0 Hz), 129.46 (s), 129.06 (s), 128.05 (d, *J* = 1.9 Hz), 125.88 (q, *J* = 283.0 Hz), 125.02 (d, *J* = 3.0 Hz), 116.27 (d, *J* = 21.0 Hz), 114.89 (d, *J* = 20.0 Hz), 77.45 (s), 59.71 (q, *J* = 28.0 Hz), 35.20 (s). HPLC (AD-H, 5% EtOH in hexanes, 1 mL/min, 210 nm): t_{major} = 15.2 min, t_{minor} = 13.1 min, 92% ee; $^{25}[\alpha]_D$ = +25.5 ° (c = 1.0 in $CHCl_3$); HRMS (ESI+) Calcd for $C_{16}H_{13}F_4NO_2SNa^+$ $(M+Na)^+$: 382.0501, Found: 382.0496.

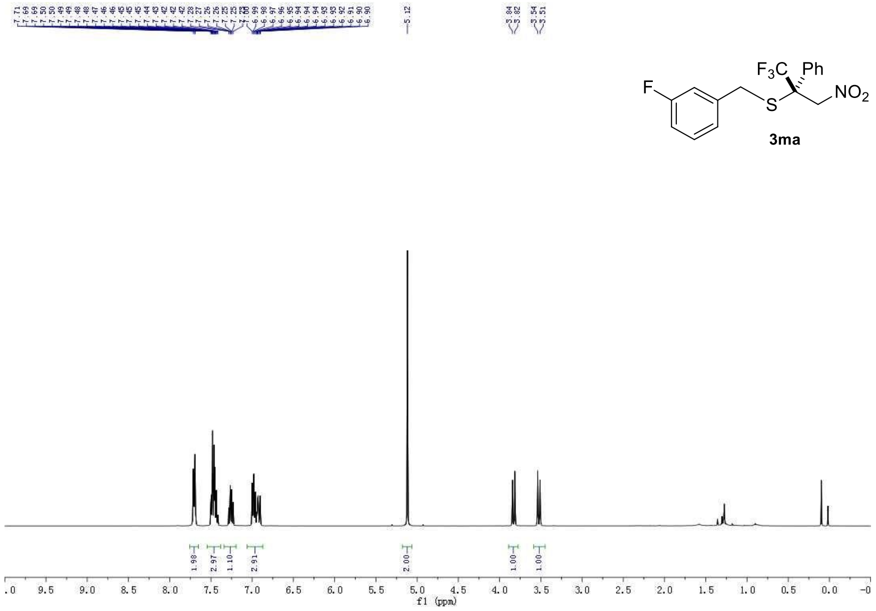

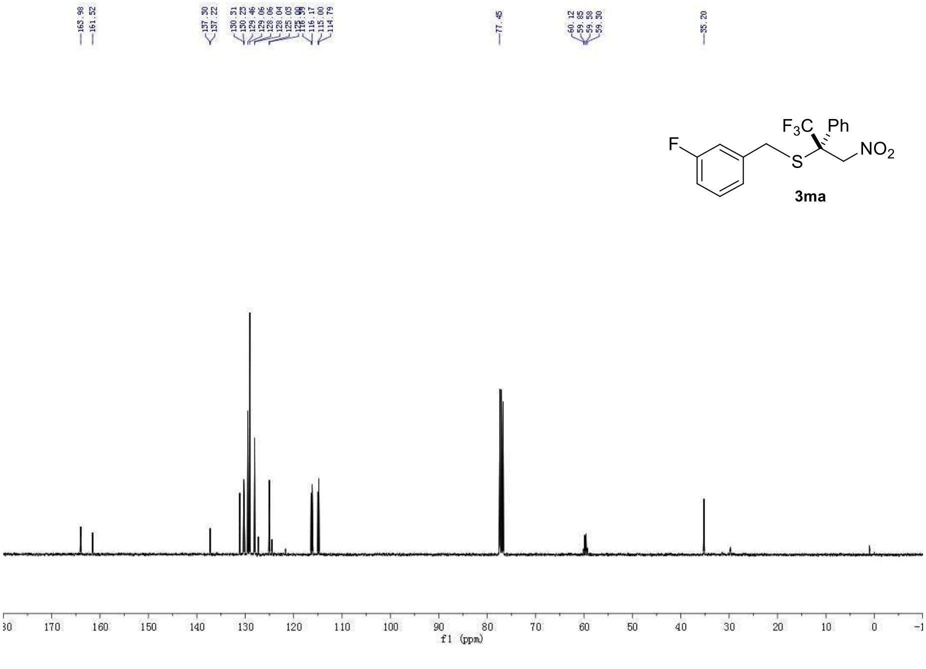

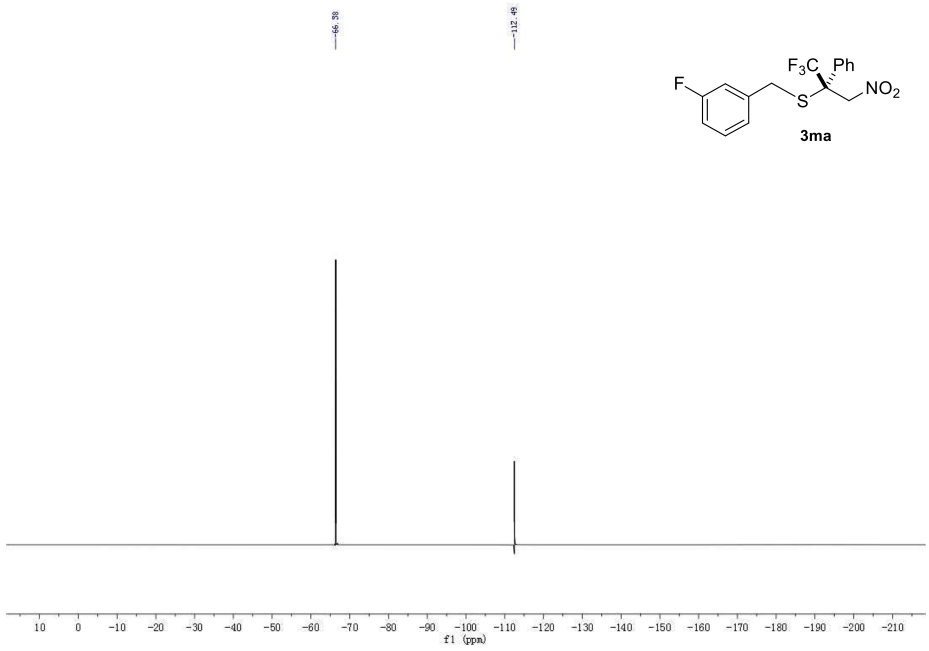

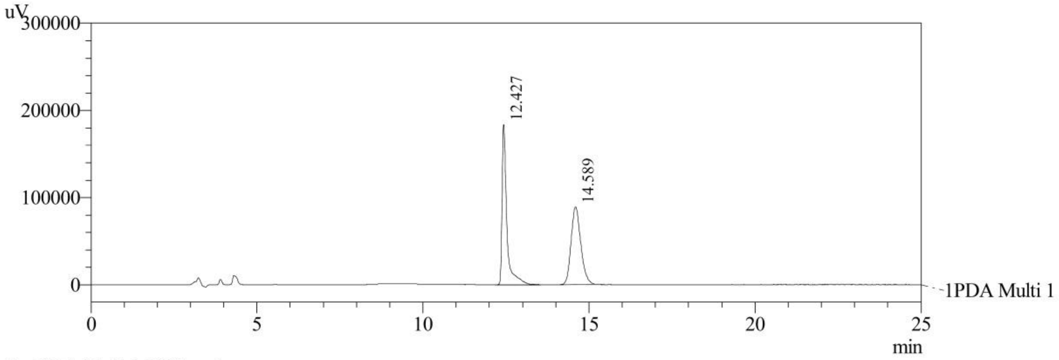

1 PDA Multi 1 / 210nm 4nm

PeakTable

PDA Ch1 210nm 4nm

Peak#	Ret. Time	Area	Height	Area %	Height %
1	12.427	1823520	183811	49.932	67.324
2	14.589	1828462	89214	50.068	32.676
Total		3651982	273024	100.000	100.000

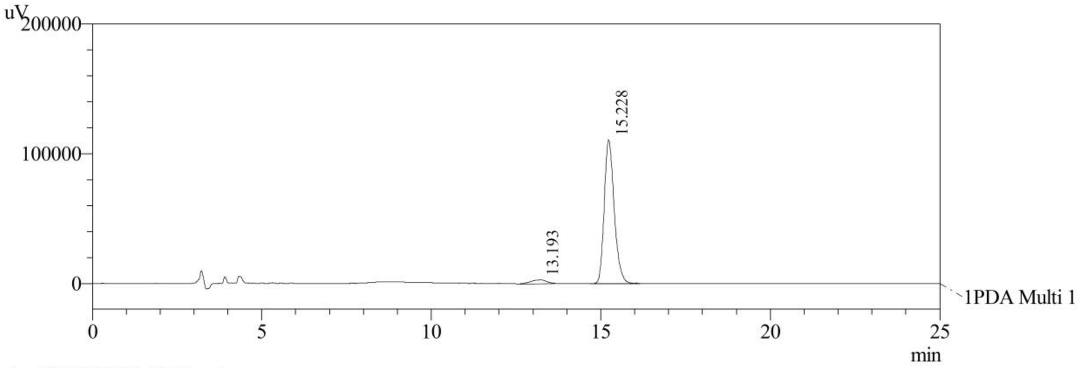

1 PDA Multi 1 / 210nm 4nm

PeakTable

PDA Ch1 210nm 4nm

Peak#	Ret. Time	Area	Height	Area %	Height %
1	13.193	95153	2969	4.016	2.601
2	15.228	2274186	111175	95.984	97.399
Total		2369339	114145	100.000	100.000

(R)-(4-methylbenzyl)(1,1,1-trifluoro-3-nitro-2-phenylpropan-2-yl)sulfane (3na)

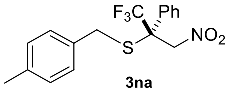

3na

The title compound was prepared according to the general procedure A and purified by flash column chromatography (50:1 hexanes : EtOAc) to afford **3na** (34 mg, 96%) as a colorless oil. Analytical data: IR (KBr, cm^{-1}) 2923, 2308, 1564, 1513, 1448, 1368, 1217, 1152, 695; ^{1}H NMR (400 MHz, $CDCl_3$) δ 7.73 (d, J = 8.3 Hz, 2H), 7.56 – 7.37 (m, 3H), 7.13 (s, 4H), 5.12 (s, 2H), 3.85 (d, J = 11.2 Hz, 1H), 3.54 (d, J = 10.9 Hz, 1H), 2.34 (s, 3H); ^{19}F NMR (376 MHz, $CDCl_3$) δ -66.31 (s, 3F); ^{13}C NMR (100 MHz, $CDCl_3$) δ 137.76 (s), 131.57 (s), 131.42 (s), 129.49 (s), 129.30 (s), 128.98 (s), 128.09 (s), 128.07 (s), 125.98 (q, J = 283.0 Hz), 77.43 (s), 59.63 (q, J = 27.0 Hz), 35.46 (s), 21.14 (s). HPLC (AD-H, 5% EtOH in hexanes, 1 mL/min, 210 nm): t_{major} = 11.4 min, t_{minor} = 9.0 min, 85% ee; $^{25}[\alpha]_D$ = +27.5 ° (c = 1.0 in $CHCl_3$); HRMS (ESI+) Calcd for $C_{17}H_{16}F_3NO_2SNa^+$ $(M+Na)^+$: 378.0752, Found: 378.0746.

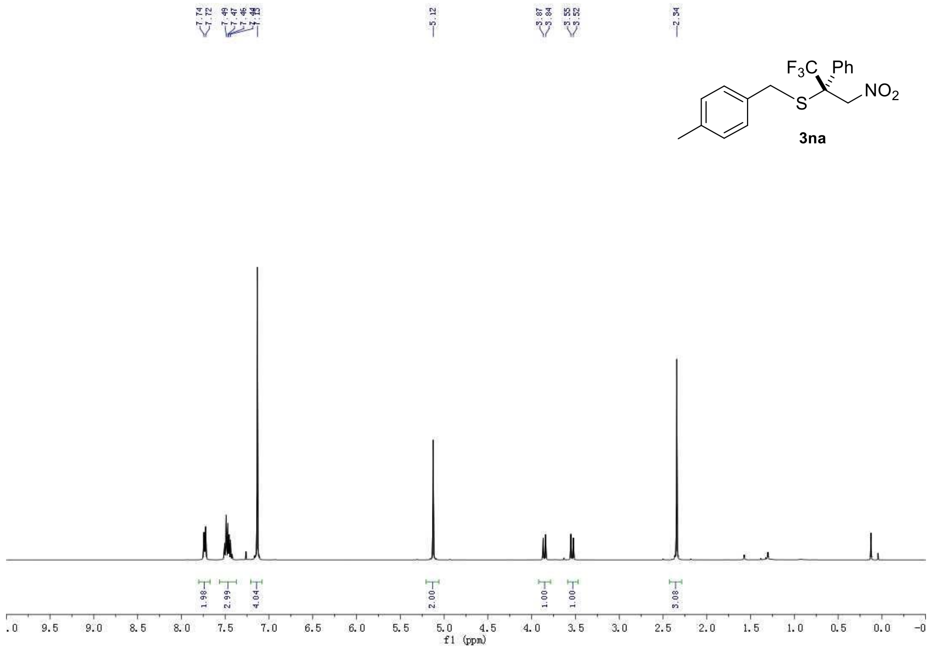

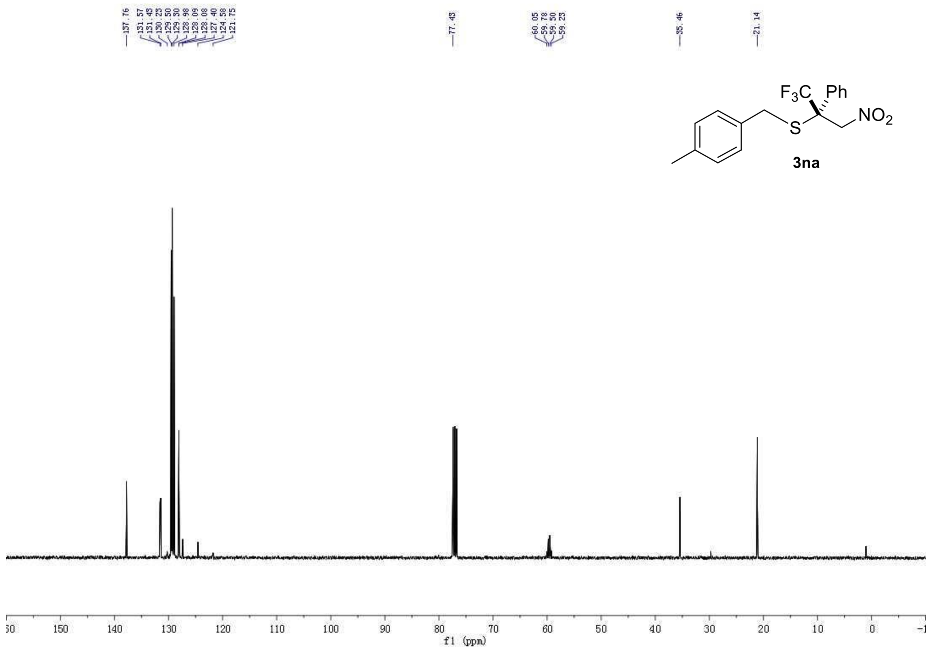

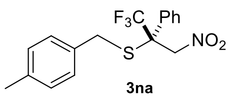

3na

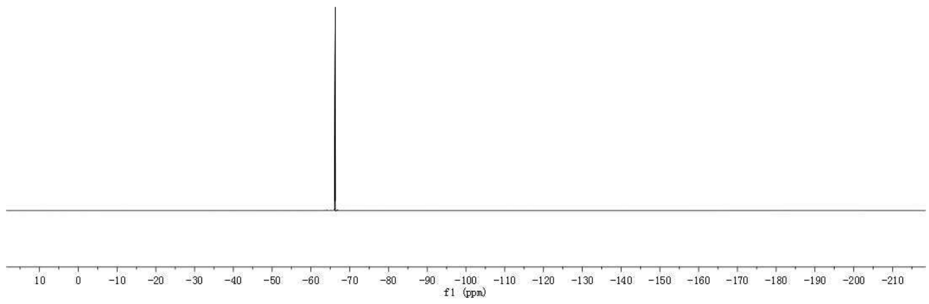

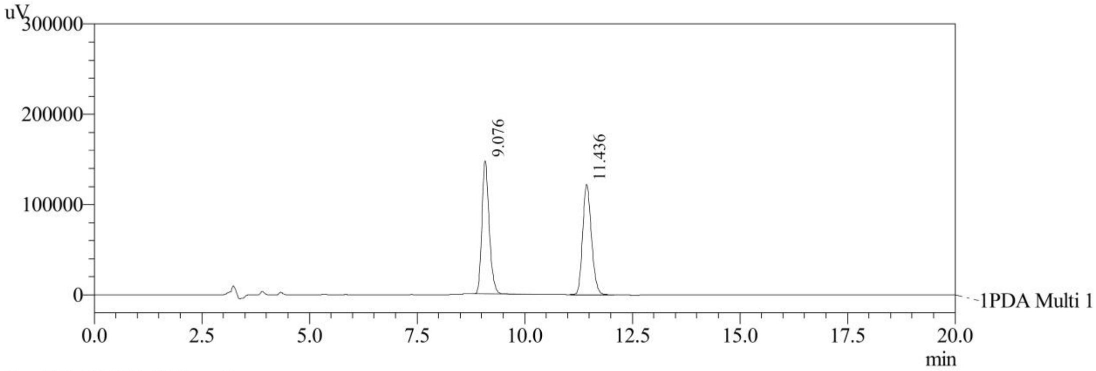

1 PDA Multi 1 / 210nm 4nm

PeakTable

PDA Ch1 210nm 4nm

Peak#	Ret. Time	Area	Height	Area %	Height %
1	9.076	1736329	147453	50.030	54.715
2	11.436	1734226	122039	49.970	45.285
Total		3470556	269491	100.000	100.000

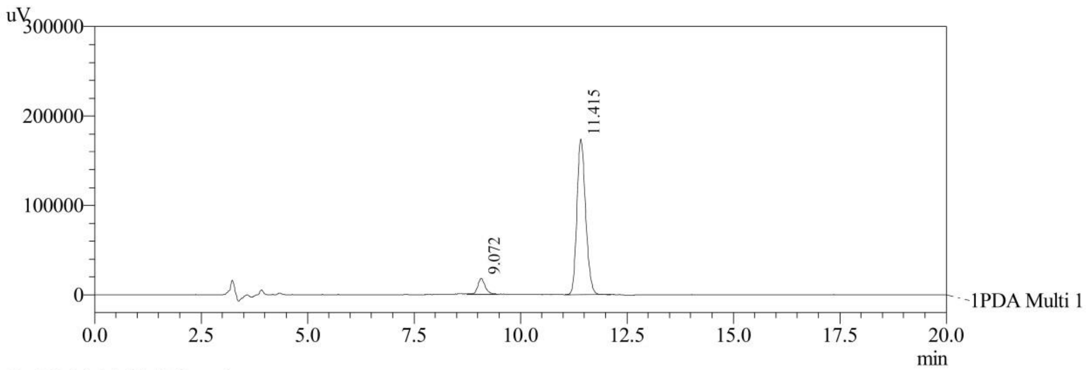

1 PDA Multi 1 / 210nm 4nm

PeakTable

PDA Ch1 210nm 4nm

Peak#	Ret. Time	Area	Height	Area %	Height %
1	9.072	205515	17536	7.657	9.153
2	11.415	2478516	174058	92.343	90.847
Total		2684031	191594	100.000	100.000

(R)-(4-chlorobenzyl)(1,1,1-trifluoro-3-nitro-2-phenylpropan-2-yl)sulfane (3oa)

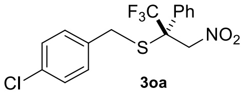

The title compound was prepared according to the general procedure A and purified by flash column chromatography (50:1 hexanes : EtOAc) to afford **3oa** (36 mg, 96%) as a slightly yellow oil. Analytical data: IR (KBr, cm^{-1}) 2920,2321, 1559, 1490, 1368, 1217, 1151, 1094, 1015, 695; ^{1}H NMR (400 MHz, $CDCl_3$) δ 7.70 (d, *J* = 7.8 Hz, 2H), 7.53 – 7.39 (m, 3H), 7.26 (d, *J* = 8.4 Hz, 2H), 7.14 (d, *J* = 8.4 Hz, 2H), 5.12 (s, 2H), 3.82 (d, *J* = 11.6 Hz, 1H), 3.51 (d, *J* = 11.2 Hz, 1H); ^{19}F NMR (376 MHz, $CDCl_3$) δ -66.43 (s, 3F); ^{13}C NMR (100 MHz, $CDCl_3$) δ 133.80 (s), 133.29 (s), 131.15 (s), 130.72 (s), 129.44 (s), 129.05 (s), 128.93 (s), 128.04 (d, *J* = 2.0 Hz), 125.88 (q, *J* = 283.0 Hz), 77.39 (s), 59.68 (q, *J* = 27.0 Hz), 35.02 (s). HPLC (AD-H, 5% EtOH in hexanes, 1 mL/min, 210 nm): t_{major} = 14.6 min, t_{minor} = 11.0 min, 84% ee; $^{25}[\alpha]_D$ = +27.5 ° (c = 1.0 in $CHCl_3$); HRMS (ESI+) Calcd for $C_{16}H_{13}F_3ClNO_2SNa^+$ $(M+Na)^+$: 398.0205, Found: 398.0202.

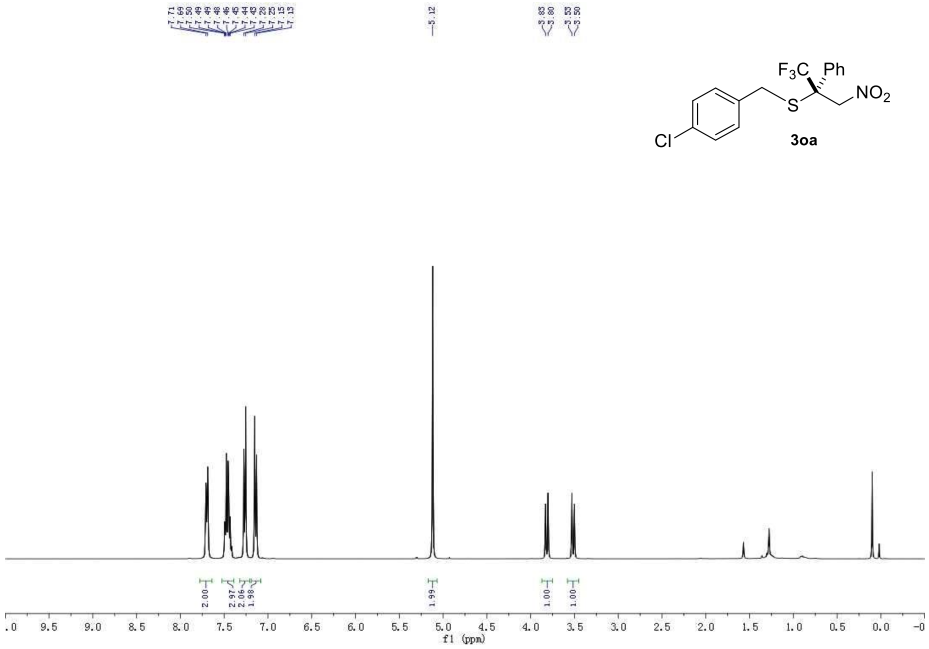

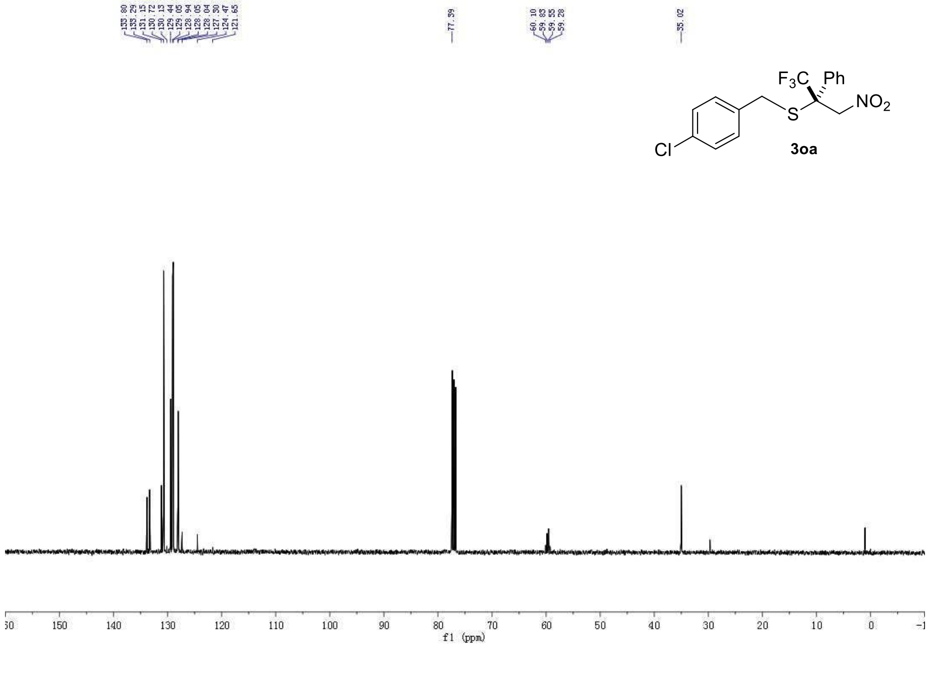

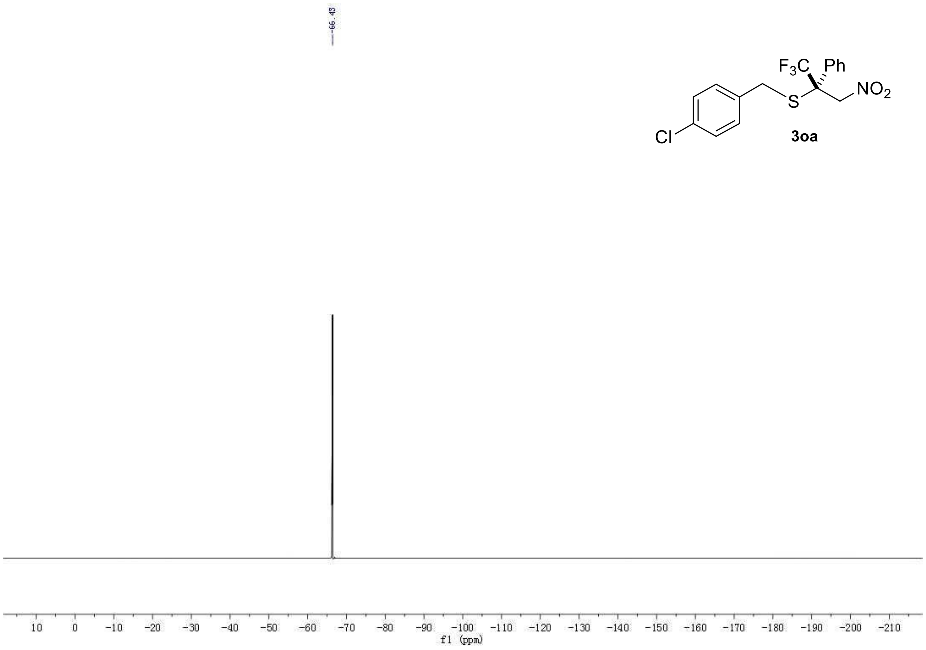

3oa

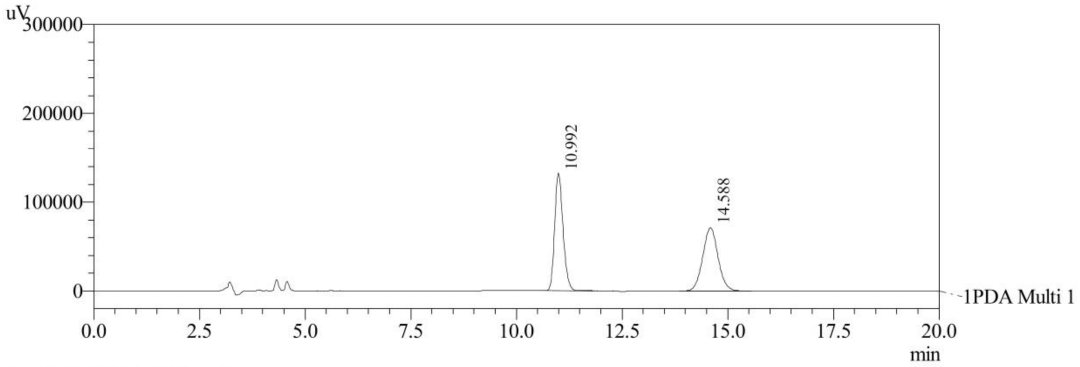

1 PDA Multi 1 / 210nm 4nm

PeakTable

PDA Ch1 210nm 4nm

Peak#	Ret. Time	Area	Height	Area %	Height %
1	10.992	1807013	132469	50.049	65.021
2	14.588	1803486	71263	49.951	34.979
Total		3610500	203731	100.000	100.000

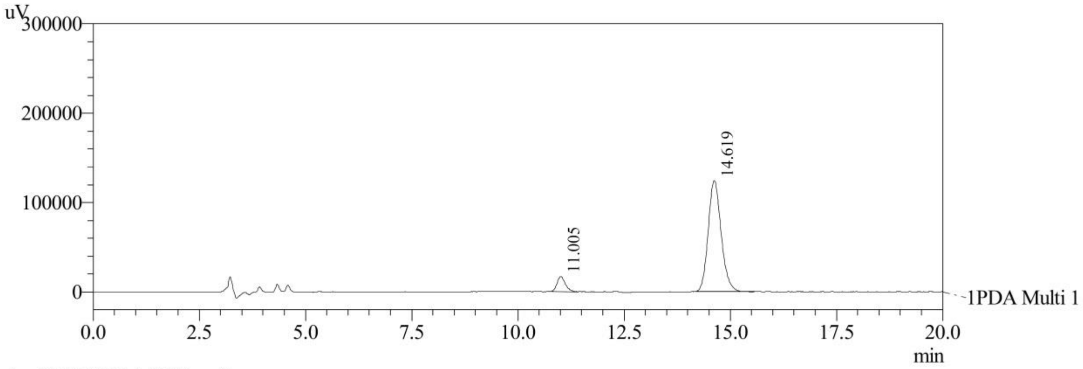

1 PDA Multi 1 / 210nm 4nm

PeakTable

PDA Ch1 210nm 4nm

Peak#	Ret. Time	Area	Height	Area %	Height %
1	11.005	229081	16750	7.946	11.864
2	14.619	2654064	124432	92.054	88.136
Total		2883145	141182	100.000	100.000

(R)-(2-(4-chlorophenyl)-1,1,1-trifluoro-3-nitropropan-2-yl)(propyl)sulfane (3cb)

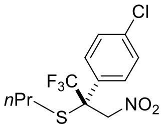

3cb

The title compound was prepared according to the general procedure A and purified by flash column chromatography (50:1 hexanes : EtOAc) to afford **3cb** (30 mg, 91%) as a colorless oil. Analytical data: IR (KBr, cm^{-1}) 2966, 2933, 2308, 1565, 1496, 1369, 1218, 1168, 1099, 1010, 813, 666; ^{1}H NMR (400 MHz, $CDCl_3$) δ 7.60 (d, *J* = 8.2 Hz, 2H), 7.48 – 7.34 (m, 2H), 5.17 – 4.94 (m, 2H), 2.59 (dt, *J* = 10.7, 7.2 Hz, 1H), 2.37 (dt, *J* = 10.9, 7.4 Hz, 1H), 1.64 – 1.45 (m, 2H), 0.95 (t, *J* = 7.4 Hz, 3H); ^{19}F NMR (376 MHz, $CDCl_3$) δ -66.74 (s, 3F); ^{13}C NMR (100 MHz, $CDCl_3$) δ 135.33 (s), 130.23 (s), 129.56 (d, *J* = 1.0 Hz), 129.04 (s), 125.72 (q, *J* = 283.0 Hz), 77.38 (s), 58.49 (q, *J* = 27.0 Hz), 32.71 (s), 21.70 (s), 13.37 (s). HPLC (OJ-H, 5% EtOH in hexanes, 1 mL/min, 210 nm): t_{major} = 10.0 min, t_{minor} = 17.5 min, 92% ee; $^{25}[\alpha]_D$ = -8.3 ^{o} (c = 1.0 in $CHCl_3$); HRMS (ESI-) Calcd for $C_{12}H_{12}F_3ClNO_2S^-$ $(M-H)^-$: 326.0235, Found: 326.0232.

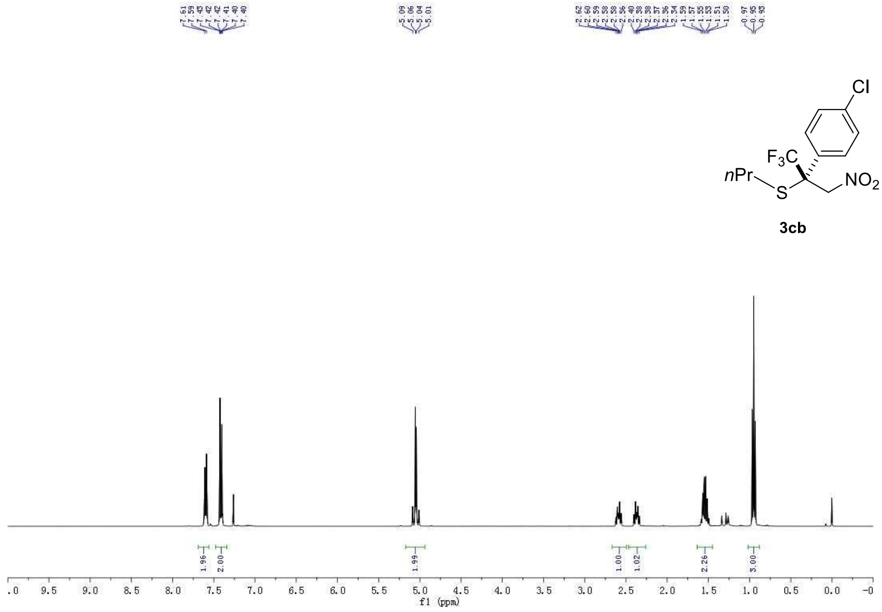

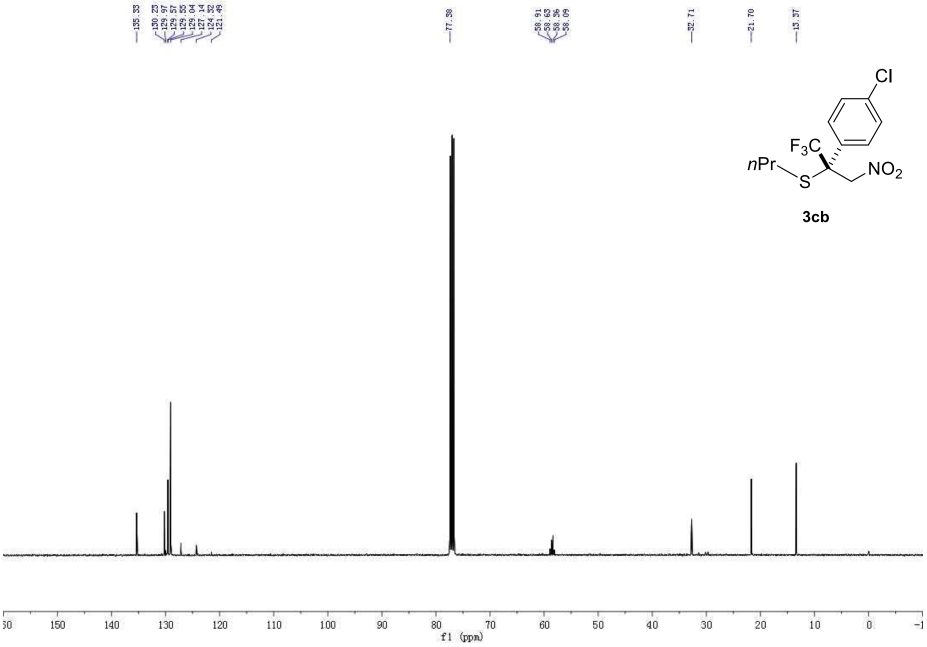

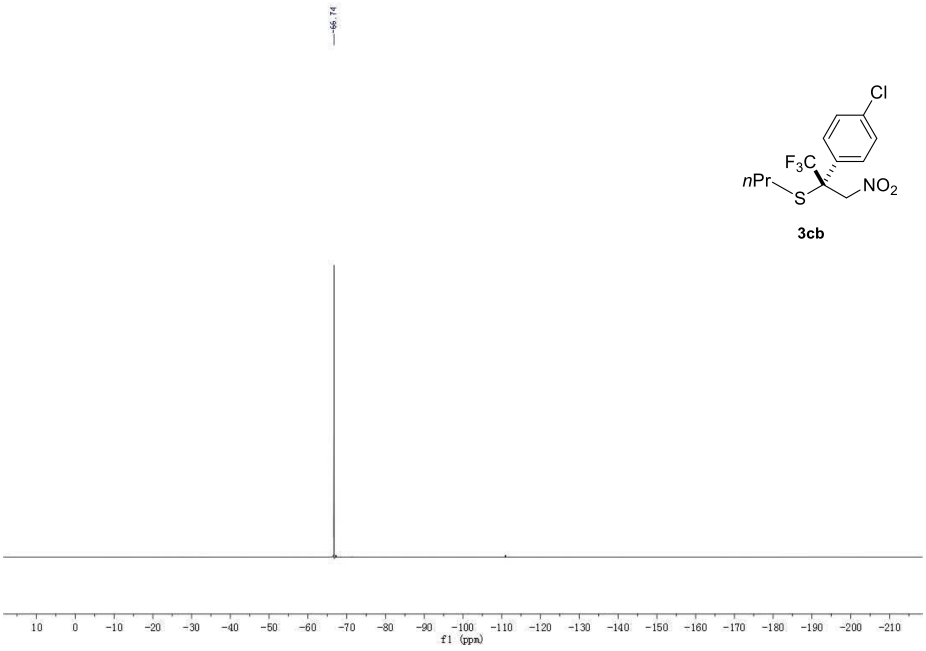

3cb

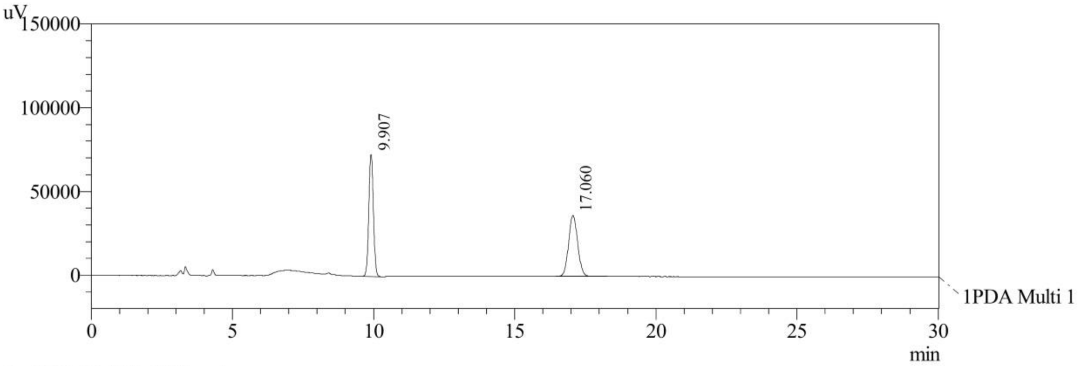

1 PDA Multi 1 / 210nm 4nm

PeakTable

PDA Ch1 210nm 4nm

Peak#	Ret. Time	Area	Height	Area %	Height %
1	9.907	823456	73004	50.053	66.622
2	17.060	821699	36575	49.947	33.378
Total		1645155	109579	100.000	100.000

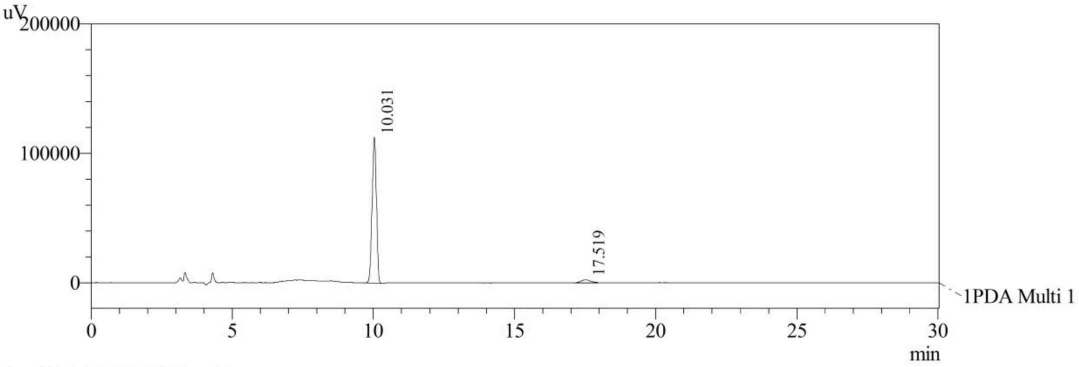

1 PDA Multi 1 / 210nm 4nm

PeakTable

PDA Ch1 210nm 4nm

Peak#	Ret. Time	Area	Height	Area %	Height %
1	10.031	1215474	113181	95.940	98.008
2	17.519	51443	2301	4.060	1.992
Total		1266917	115482	100.000	100.000

(R)-cyclohexyl(1,1,1-trifluoro-3-nitro-2-(p-tolyl)propan-2-yl)sulfane (3hc)

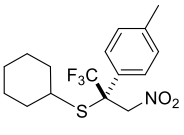

3hc

The title compound was prepared according to the general procedure A and purified by column chromatography (50:1 hexanes : EtOAc) to afford **3hc** (29 mg, 85%) as a colorless oil. Analytical data: IR (KBr, cm^{-1}) 2931, 2854, 2310, 1560, 1448, 1369, 1219, 1149, 804, 668; ^{1}H NMR (400 MHz, $CDCl_3$) δ 7.53 (d, *J* = 7.9 Hz, 2H), 7.23 (d, *J* = 8.2 Hz, 2H), 5.04 (q, *J* = 12.6 Hz, 2H), 2.64 – 2.50 (m, 1H), 2.38 (s, 3H), 1.88 (dd, *J* = 9.2, 3.8 Hz, 1H), 1.75 – 1.52 (m, 3H), 1.42 (ddd, *J* = 13.4, 10.6, 4.0 Hz, 2H), 1.33 – 1.09 (m, 4H); ^{19}F NMR (376 MHz, $CDCl_3$) δ -66.14 (s, 3F); ^{13}C NMR (100 MHz, $CDCl_3$) δ 139.06 (s), 129.63 (s), 128.83 (s), 127.94 (d, *J* = 2.0 Hz), 125.91 (q, *J* = 282.0 Hz), 78.16 (s), 59.49 (q, *J* = 28.0 Hz), 44.35 (s), 34.93 (s), 34.70 (s), 26.04 (s), 25.18 (s), 21.01 (s). HPLC (AD-H, 5% EtOH in hexanes, 1 mL/min, 210 nm): t_{major} = 5.6 min, t_{minor} = 16.6 min, 91% ee; $^{25}[\alpha]_D$ = -21.5 ^{o} (c = 1.0 in $CHCl_3$); HRMS (ESI+) Calcd for $C_{16}H_{20}F_3NO_2SNa^+$ $(M+Na)^+$: 370.1065, Found: 370.1058.

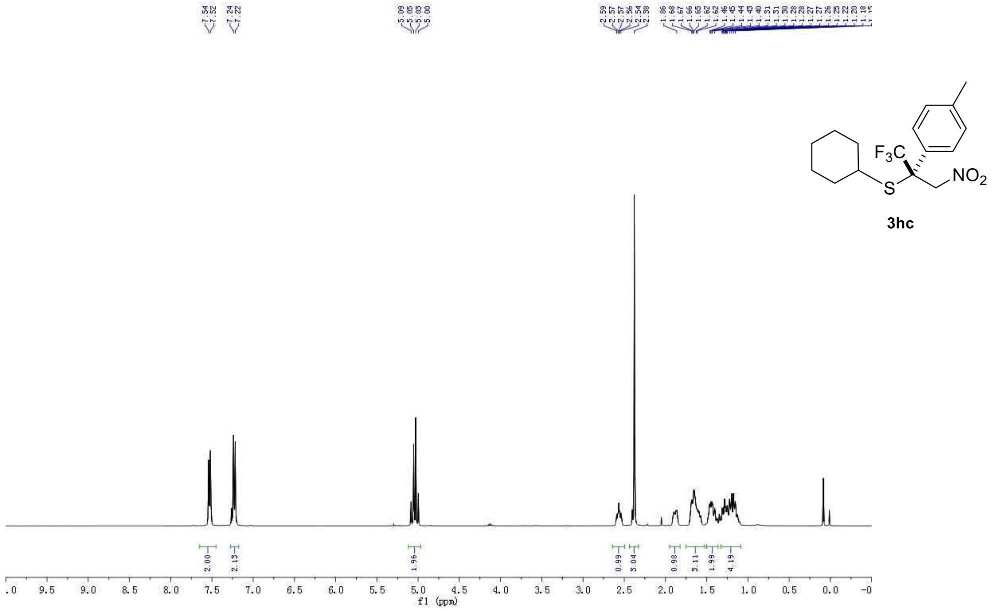

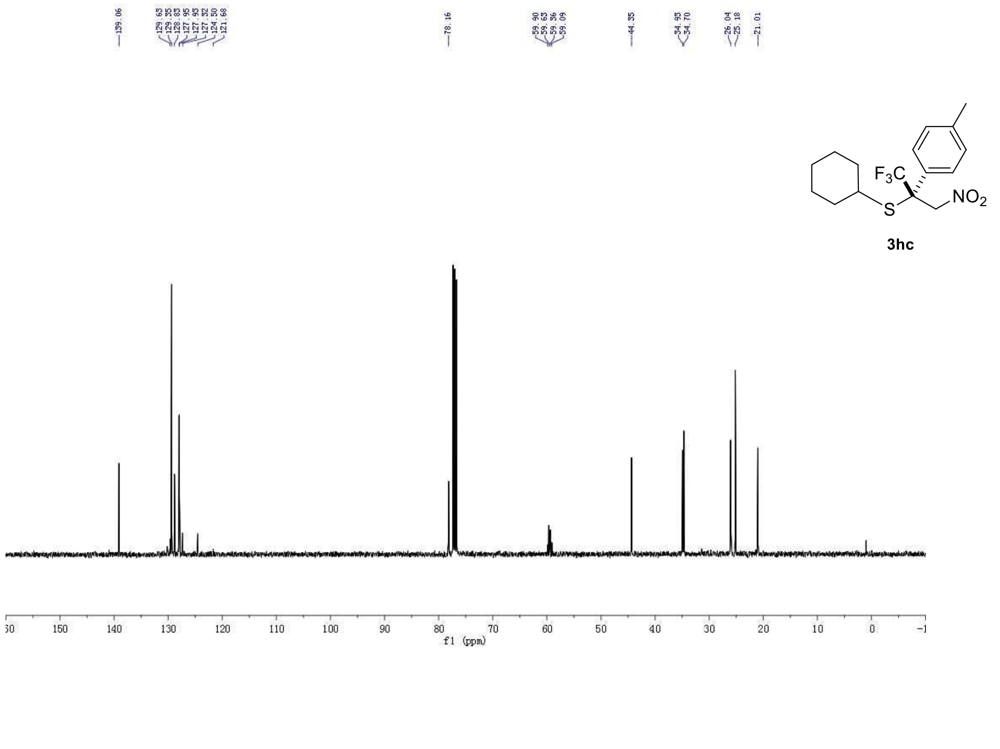

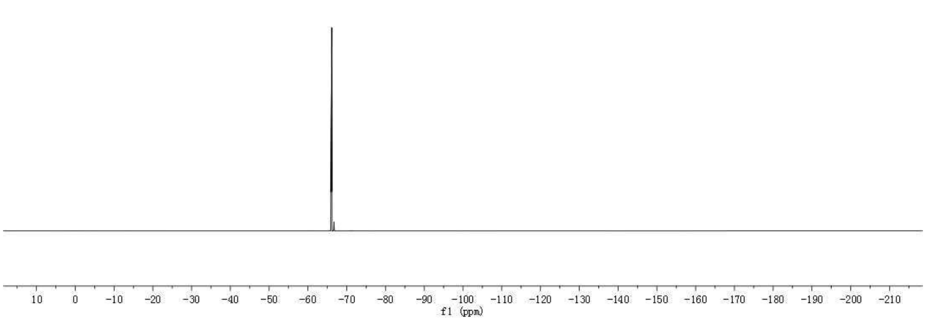

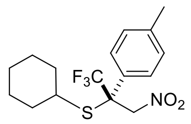

3hc

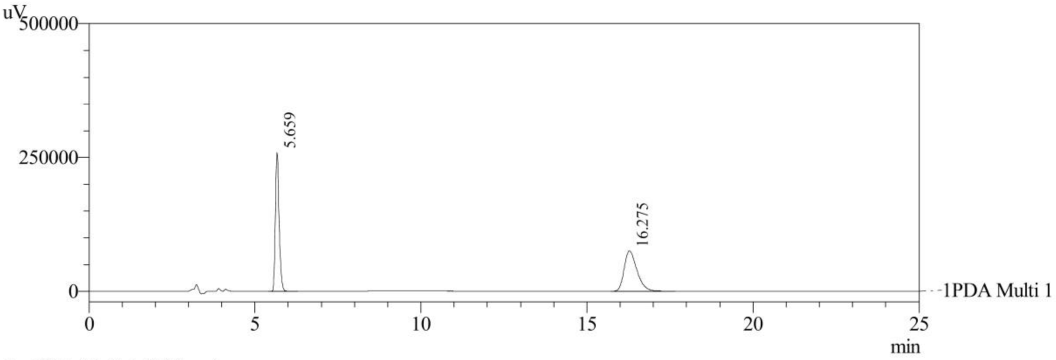

1 PDA Multi 1 / 210nm 4nm

PeakTable

PDA Ch1 210nm 4nm

Peak#	Ret. Time	Area	Height	Area %	Height %
1	5.659	2020906	259522	50.083	77.532
2	16.275	2014207	75208	49.917	22.468
Total		4035113	334730	100.000	100.000

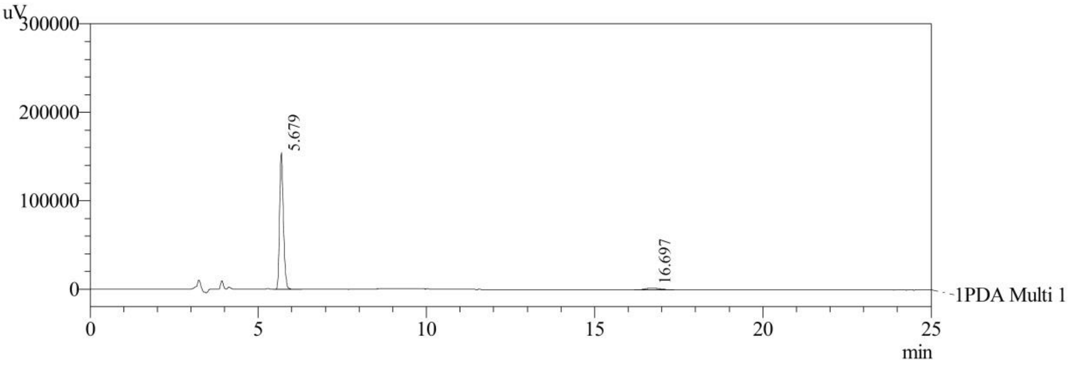

1 PDA Multi 1 / 210nm 4nm

PeakTable

PDA Ch1 210nm 4nm

Peak#	Ret. Time	Area	Height	Area %	Height %
1	5.679	1203341	154726	95.202	98.559
2	16.697	60641	2262	4.798	1.441
Total		1263982	156989	100.000	100.000

(R)-cyclohexyl(1,1,1-trifluoro-2-(4-methoxyphenyl)-3-nitropropan-2-yl)sulfane (3hd)

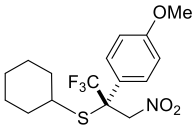

3hd

The title compound was prepared according to the general procedure A and purified by flash column chromatography (50:1 hexanes : EtOAc) to afford **3hd** (31 mg, 86%) as a colorless oil. Analytical data: IR (KBr, cm^{-1}) 2933, 2853, 2320, 1560, 1517, 1368, 1260, 1166, 1148, 1035, 822; ^{1}H NMR (400 MHz, $CDCl_3$) δ 7.57 (d, *J* = 8.5 Hz, 2H), 6.93 (d, *J* = 9.0 Hz, 2H), 5.12 – 4.93 (m, 2H), 3.84 (s, 3H), 2.65 – 2.49 (m, 1H), 1.88 (d, *J* = 13.0 Hz, 1H), 1.72 – 1.57 (m, 3H), 1.41 (ddd, *J* = 13.4, 11.9, 4.5 Hz, 2H), 1.33 – 1.12 (m, 4H); ^{19}F NMR (376 MHz, $CDCl_3$) δ -66.33 (s, 3F); ^{13}C NMR (100 MHz, $CDCl_3$) δ 159.72 (s), 129.49 (d, *J* = 1.0 Hz),125.92 (q, *J* = 282.0 Hz), 123.52 (s), 113.93 (s), 78.19 (s), 59.36 (q, *J* = 28.0 Hz), 55.28 (s), 44.35 (s), 34.91 (s), 34.73 (s), 26.07 (s), 25.18 (s). HPLC (AD-H, 5% EtOH in hexanes, 1 mL/min, 210 nm): t_{major} = 7.6 min, t_{minor} = 20.0 min, 96% ee; $^{25}[\alpha]_D$ = -23.3 º (c = 1.0 in $CHCl_3$); HRMS (ESI+) Calcd for $C_{16}H_{20}F_3NO_3SNa^+$ $(M+Na)^+$: 386.1014, Found: 386.1005.

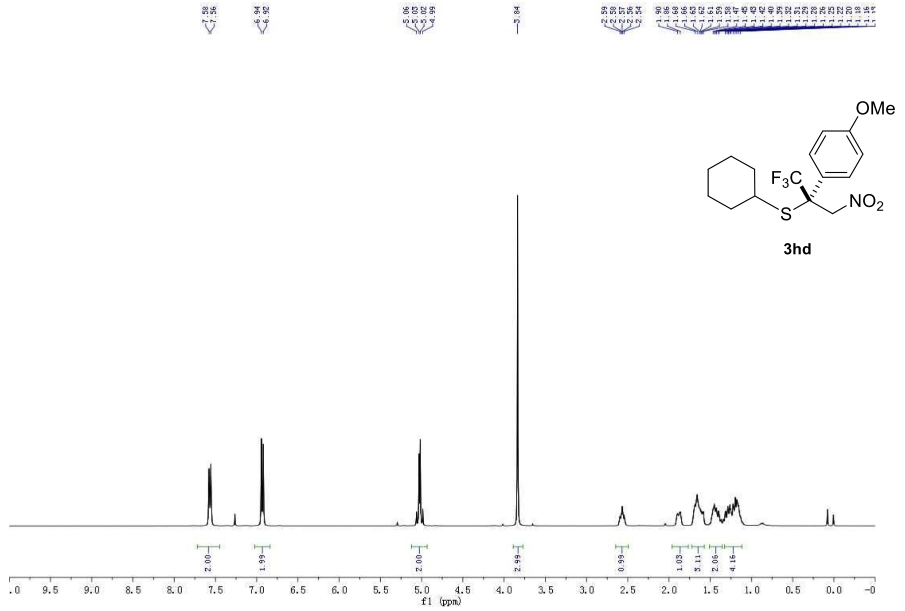

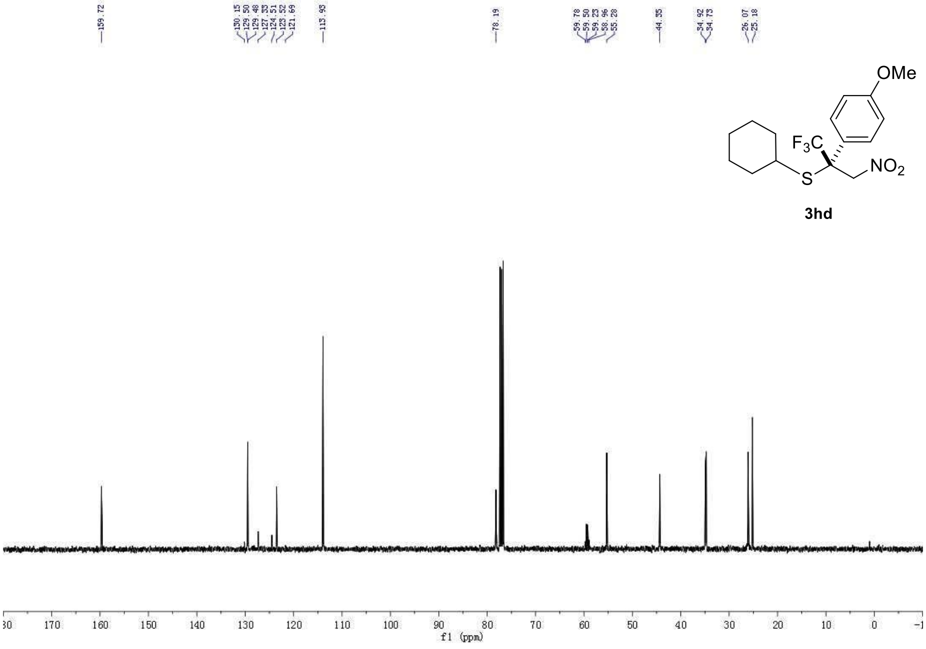

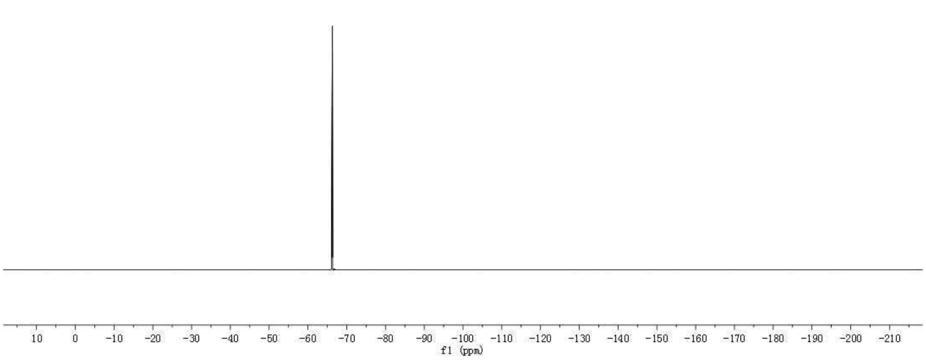

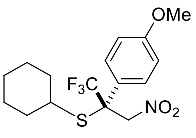

3hd

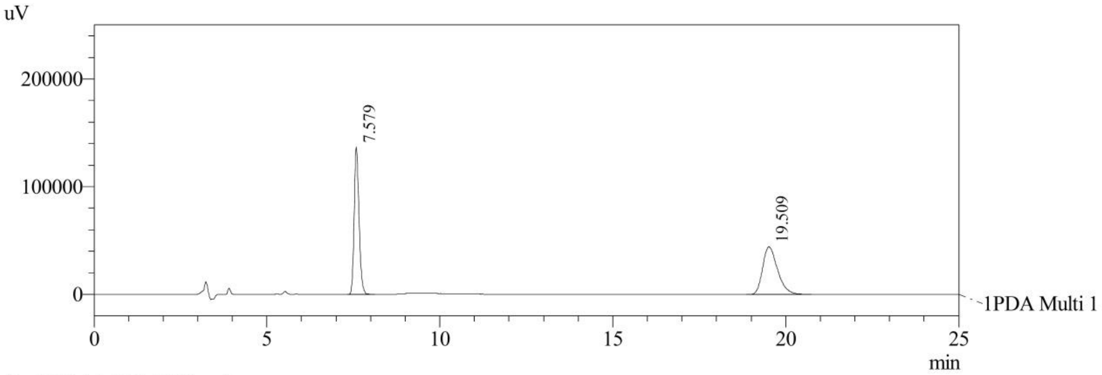

1 PDA Multi 1 / 210nm 4nm

PeakTable

PDA Ch1 210nm 4nm

Peak#	Ret. Time	Area	Height	Area %	Height %
1	7.579	1332509	136865	50.165	75.589
2	19.509	1323753	44199	49.835	24.411
Total		2656262	181064	100.000	100.000

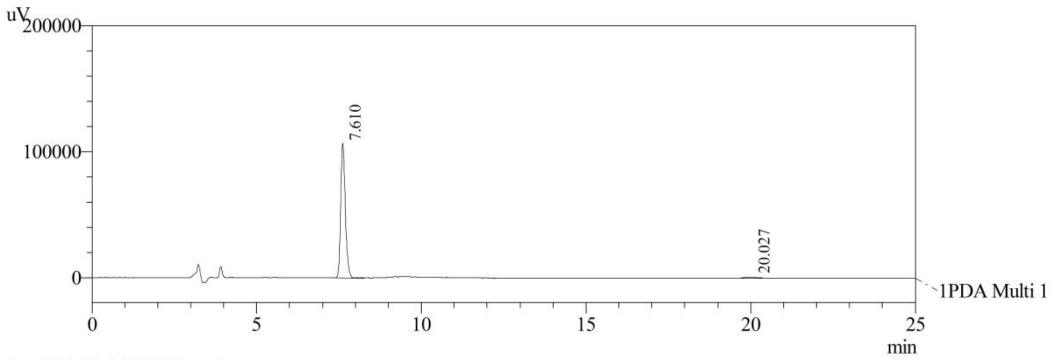

1 PDA Multi 1 / 210nm 4nm

PeakTable

PDA Ch1 210nm 4nm

Peak#	Ret. Time	Area	Height	Area %	Height %
1	7.610	1054172	107381	97.901	99.158
2	20.027	22602	912	2.099	0.842
Total		1076774	108293	100.000	100.000

(R)-phenethyl(1,1,1-trifluoro-3-nitro-2-(4-(trifluoromethyl)phenyl)propan-2-yl)sulfane (3ae)

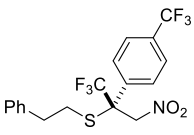

3ae

The title compound was prepared according to the general procedure A and purified by flash column chromatography (50:1 hexanes : EtOAc) to afford **3ae** (40 mg, 95%) as a colorless oil. Analytical data: IR (KBr, cm^{-1}) 1565, 1369, 1326, 1169, 1127, 1075, 1012, 749; ^{1}H NMR (400 MHz, $CDCl_3$) δ 7.74 (d, *J* = 8.5 Hz, 2H), 7.67 (d, *J* = 8.6 Hz, 2H), 7.36 – 7.21 (m, 3H), 7.15 – 7.04 (m, 2H), 5.15 – 4.97 (m, 2H), 2.96 – 2.71 (m, 3H), 2.67 – 2.57 (m, 1H); ^{19}F NMR (376 MHz, $CDCl_3$) δ -63.01 (s, 3F), -66.49 (s, 3F); ^{13}C NMR (100 MHz, $CDCl_3$) δ 138.81 (s), 135.60 (s), 131.33 (dd, *J* = 66.2, 33.1 Hz), 128.68 (d, *J* = 2.0 Hz), 128.62 (s), 128.46 (s), 126.85 (s), 125.82 (dd, *J* = 7.2, 3.7 Hz), 125.61 (q, *J* = 283.0 Hz), 123.52 (q, *J* = 270.0 Hz), 77.25 (s), 58.84 (q, *J* = 28.0 Hz), 34.60 (s), 32.23 (d, *J* = 1.0 Hz). HPLC (OJ-H, 5% EtOH in hexanes, 1 mL/min, 210 nm): t_{major} = 18.6 min, t_{minor} = 20.5 min, 86% ee; $^{25}[\alpha]_D$ = -8.5 ° (c = 1.0 in $CHCl_3$); HRMS (ESI+) Calcd for $C_{18}H_{15}F_6NO_2SNa^+$ $(M+Na)^+$: 446.0625, Found: 446.0620.

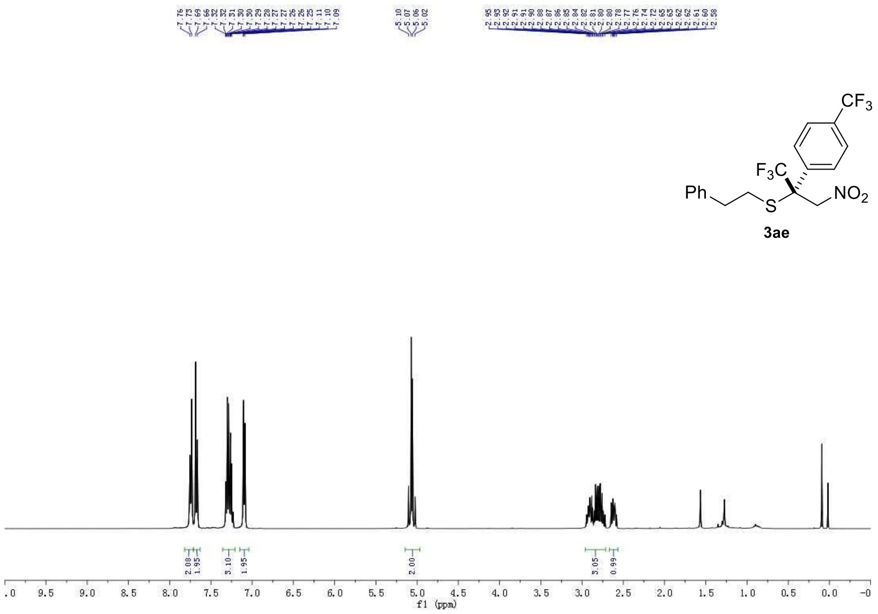

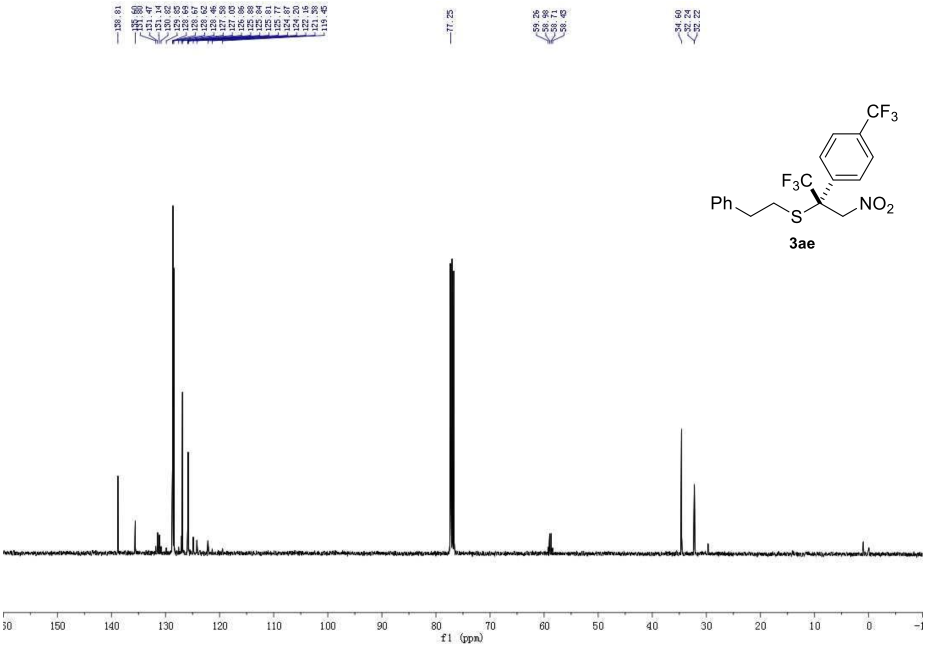

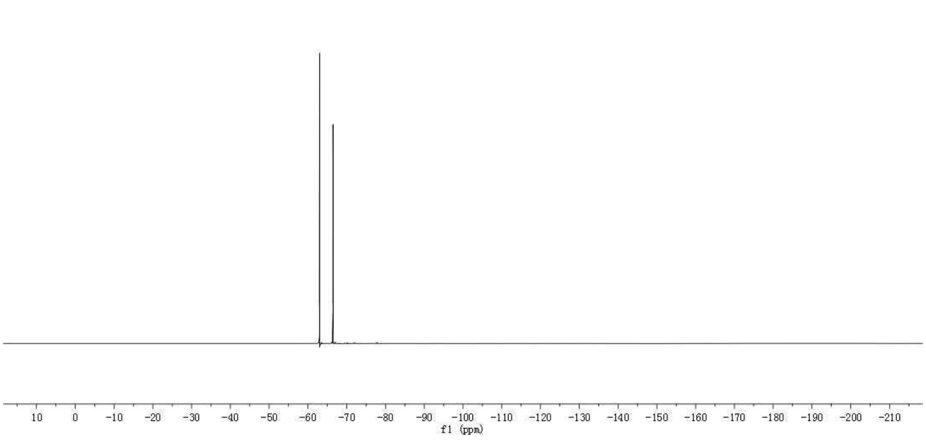

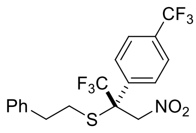

3ae

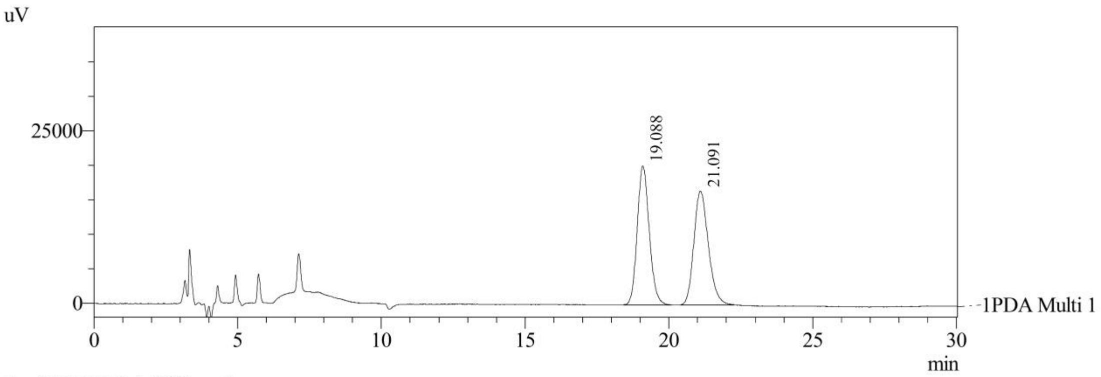

1 PDA Multi 1 / 210nm 4nm

PeakTable

PDA Ch1 210nm 4nm

Peak#	Ret. Time	Area	Height	Area %	Height %
1	19.088	586929	20133	50.359	54.977
2	21.091	578561	16488	49.641	45.023
Total		1165490	36621	100.000	100.000

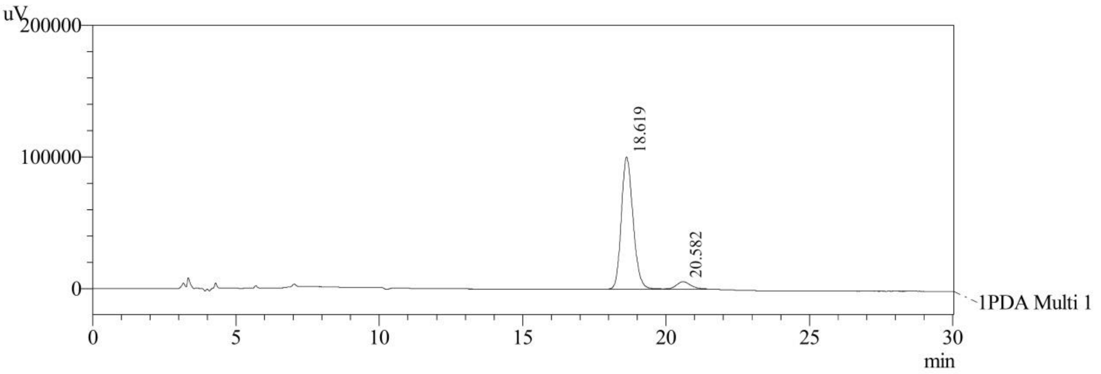

1 PDA Multi 1 / 210nm 4nm

PeakTable

PDA Ch1 210nm 4nm

Peak#	Ret. Time	Area	Height	Area %	Height %
1	18.619	2872100	100622	92.917	94.543
2	20.582	218938	5808	7.083	5.457
Total		3091038	106431	100.000	100.000

(R)-(2-(4-chlorophenyl)-1,1,1-trifluoro-3-nitropropan-2-yl)(phenethyl)sulfane (3ab)

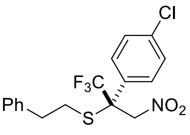

3ab

The title compound was prepared according to the general procedure A and purified by flash column chromatography (50:1 hexanes : EtOAc) to afford **3ab** (37 mg, 95%) as a colorless oil. Analytical data: IR (KBr, cm^{-1}) 2918, 2310, 1559, 1507, 1368, 1229, 1148, 1099, 1010, 810, 750, 697; ^1H NMR (400 MHz, $CDCl_3$) δ 7.56 (d, *J* = 8.5 Hz, 2H), 7.40 (d, *J* = 8.8 Hz, 2H), 7.35 – 7.21 (m, 3H), 7.12 (d, *J* = 7.0 Hz, 2H), 5.12 – 4.94 (m, 2H), 2.95 – 2.71 (m, 3H), 2.71 – 2.57 (m, 1H); ^{19}F NMR (376 MHz, $CDCl_3$) δ -66.66 (s, 3F); ^{13}C NMR (100 MHz, $CDCl_3$) δ 138.98 (s), 135.43 (s), 130.01 (s), 127.59 (d, *J* = 1.0 Hz), 129.12 (s), 128.61 (s), 128.47 (s), 126.82 (s), 125.69 (q, *J* = 282.0 Hz), 77.39 (s), 58.78 (q, *J* = 27.0 Hz), 34.65 (s), 32.18 (s). HPLC (AD-H, 5% EtOH in hexanes, 1 mL/min, 210 nm): t_{major} = 9.9 min, t_{minor} = 9.5 min, 88% ee; $^{25}[\alpha]_D$ = -20.9 ^{o} (c = 1.0 in $CHCl_3$); HRMS (ESI+) Calcd for $C_{17}H_{15}F_3ClNO_2SNa^+$ $(M+Na)^+$: 412.0362, Found: 412.0356.

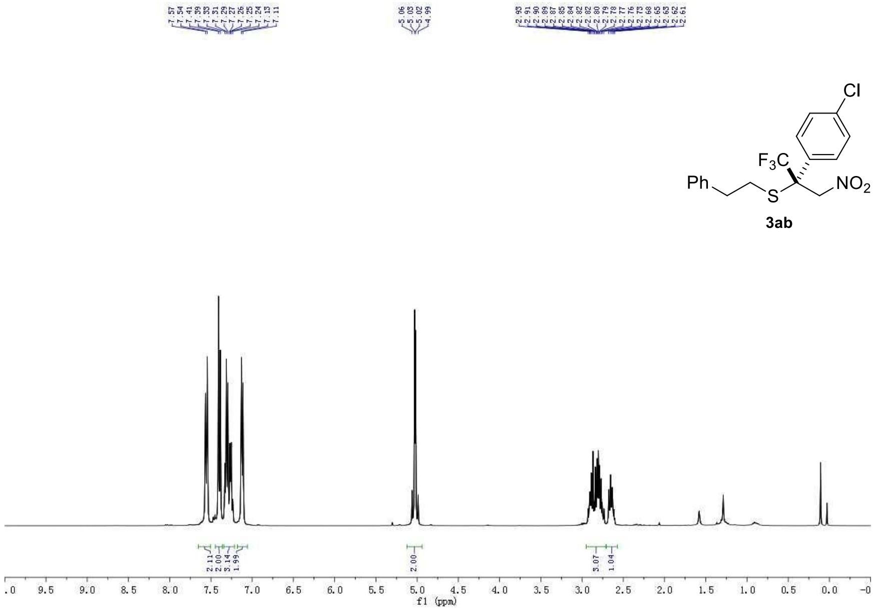

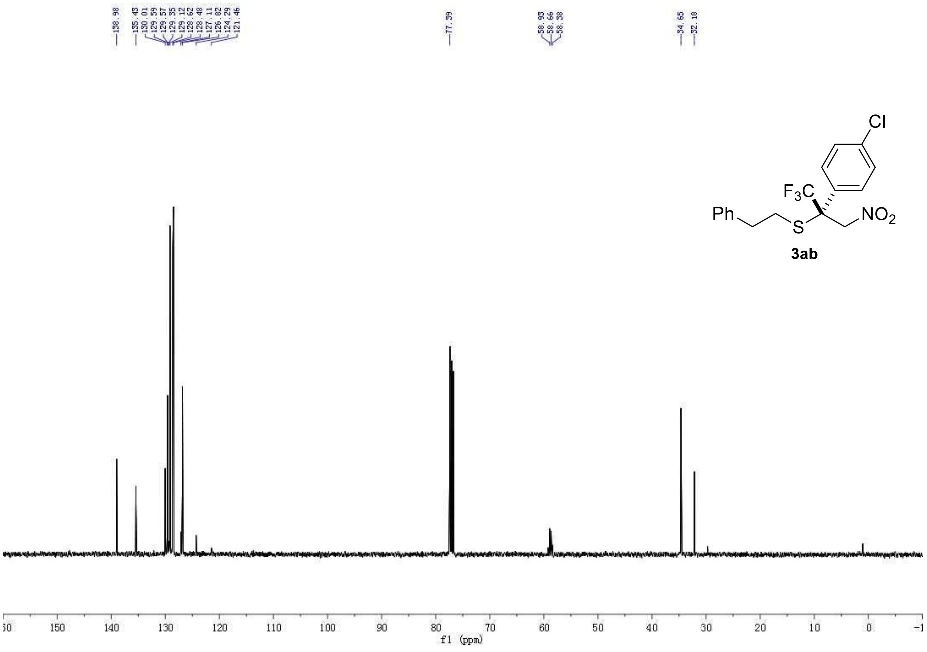

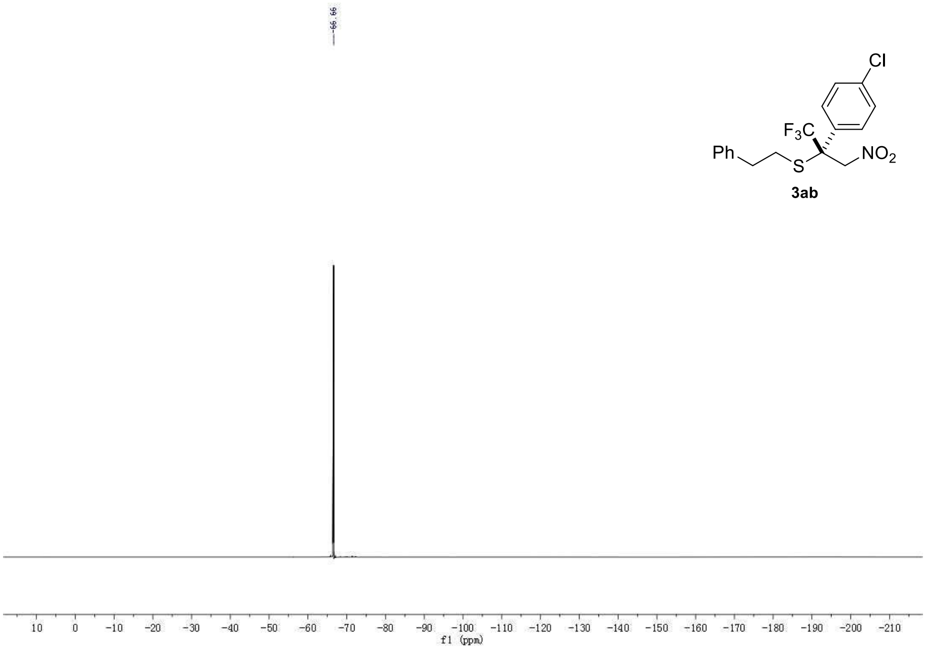

3ab

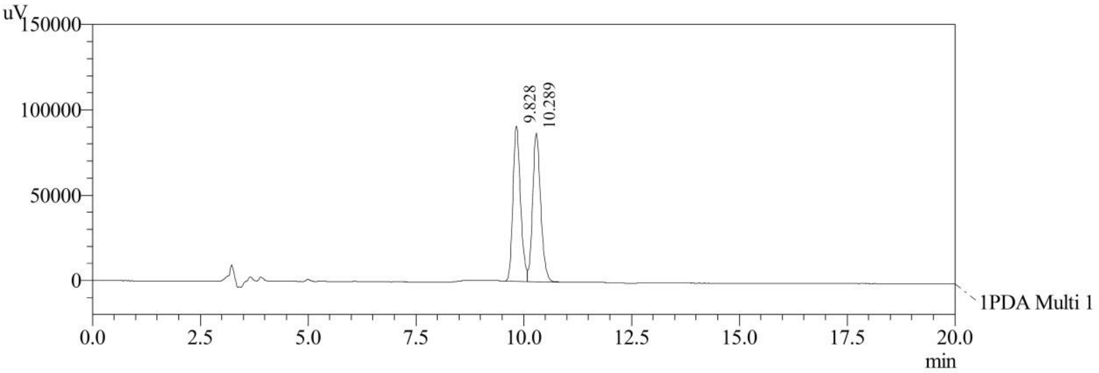

1 PDA Multi 1 / 210nm 4nm

PeakTable

PDA Ch1 210nm 4nm

Peak#	Ret. Time	Area	Height	Area %	Height %
1	9.828	1113978	90884	49.546	51.064
2	10.289	1134396	87095	50.454	48.936
Total		2248375	177979	100.000	100.000

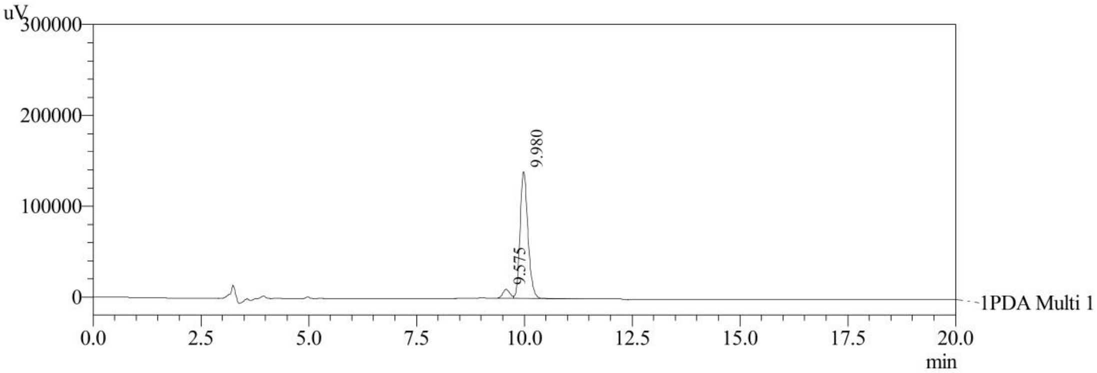

1 PDA Multi 1 / 210nm 4nm

PeakTable

PDA Ch1 210nm 4nm

Peak#	Ret. Time	Area	Height	Area %	Height %
1	9.575	113863	9936	6.080	6.635
2	9.980	1758848	139819	93.920	93.365
Total		1872711	149755	100.000	100.000

(R)-phenethyl(1,1,1-trifluoro-2-(4-fluorophenyl)-3-nitropropan-2-yl)sulfane (3af)

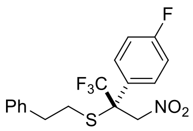

3af

The title compound was prepared according to the general procedure A and purified by flash column chromatography (50:1 hexanes : EtOAc) to afford **3af** (35 mg, 94%) as a colorless oil. Analytical data: IR (KBr, cm^{-1}) 2918, 2310, 1559, 1507, 1368, 1239, 1147, 826, 748, 697; ^{1}H NMR (400 MHz, $CDCl_3$) δ 7.62 (dd, *J* = 8.4, 5.0 Hz, 2H), 7.36 – 7.21 (m, 3H), 7.11 (dd, *J* = 12.0, 5.1 Hz, 4H), 5.10 – 4.97 (m, 2H), 2.96 – 2.72 (m, 3H), 2.72 – 2.59 (m, 1H); ^{19}F NMR (376 MHz, $CDCl_3$) δ -66.77 (s, 3F), -111.42 (s, 1F); ^{13}C NMR (100 MHz, $CDCl_3$) δ 162.70 (d, *J* = 274.0 Hz), 139.06 (s), 130.30 (d, *J* = 2.0 Hz), 130.21 (d, *J* = 2.0 Hz), 128.53 (d, *J* = 14.0 Hz), 127.23 (d, *J* = 4.0 Hz), 126.79 (s), 125.77 (q, *J* = 283.0 Hz), 115.95 (d, *J* = 22.0 Hz), 77.53 (s), 58.75 (q, *J* = 27.0 Hz), 34.67 (s), 32.18 (s). HPLC (AD-H, 5% EtOH in hexanes, 1 mL/min, 210 nm): t_{major} = 10.8 min, t_{minor} = 10.0 min, 91% ee; $^{25}[\alpha]_D$ = -10.8 ^{o} (c = 1.0 in $CHCl_3$); HRMS (ESI+) Calcd for $C_{17}H_{15}F_4NO_2SNa^+$ $(M+Na)^+$: 396.0657, Found: 396.0652.

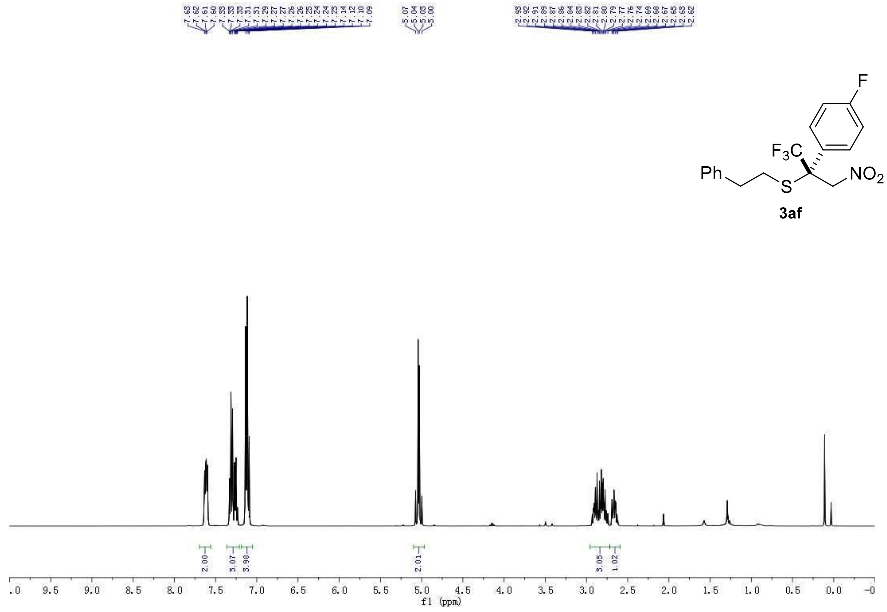

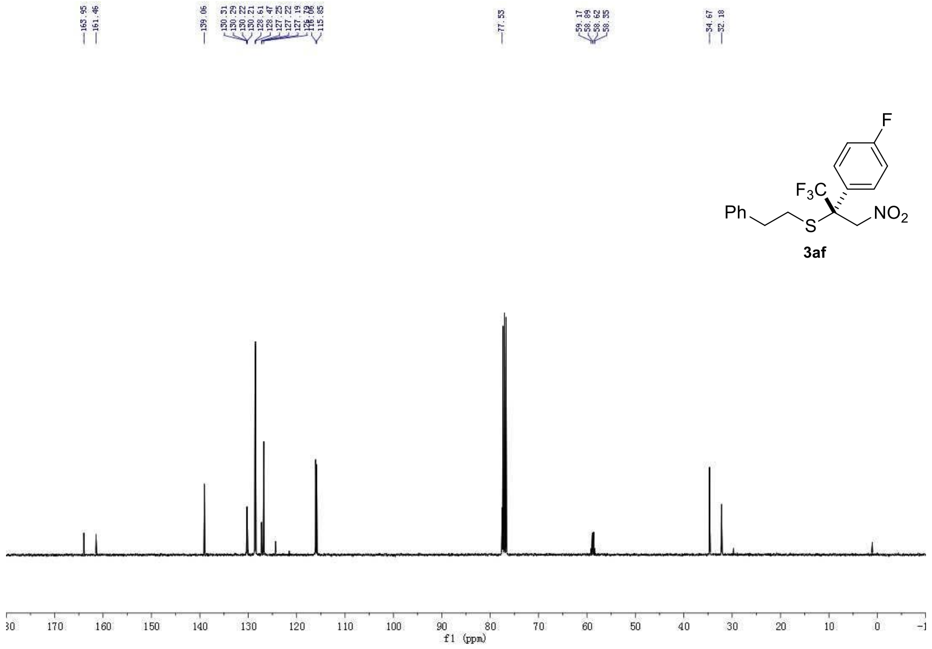

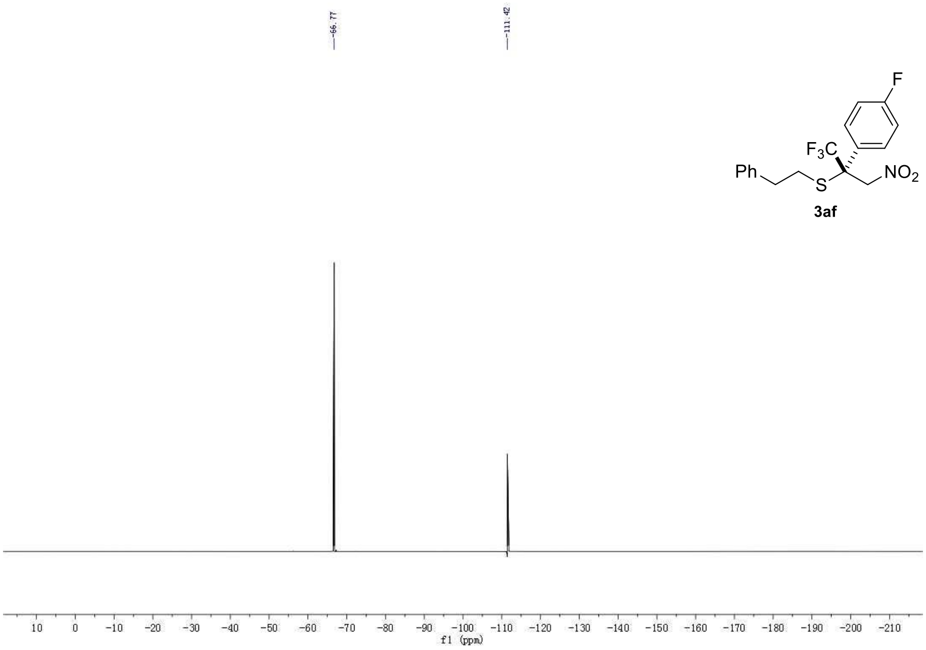

3af

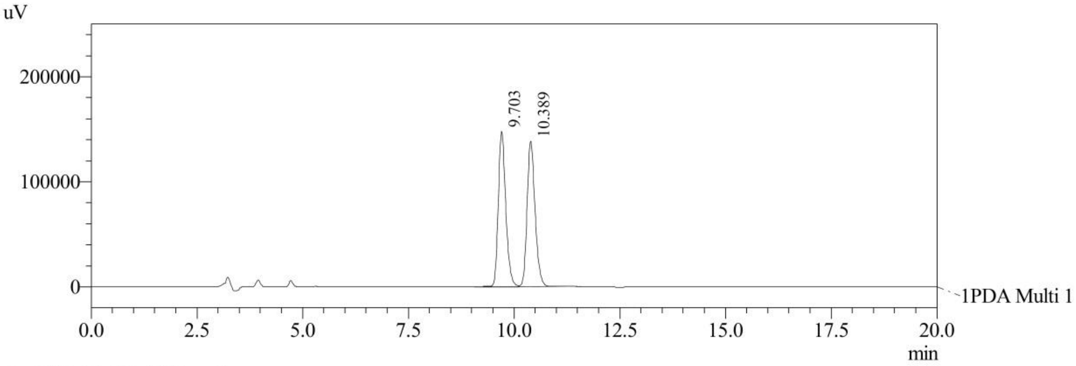

1 PDA Multi 1 / 210nm 4nm

PeakTable

PDA Ch1 210nm 4nm

Peak#	Ret. Time	Area	Height	Area %	Height %
1	9.703	1823071	147767	50.017	51.630
2	10.389	1821832	138437	49.983	48.370
Total		3644903	286204	100.000	100.000

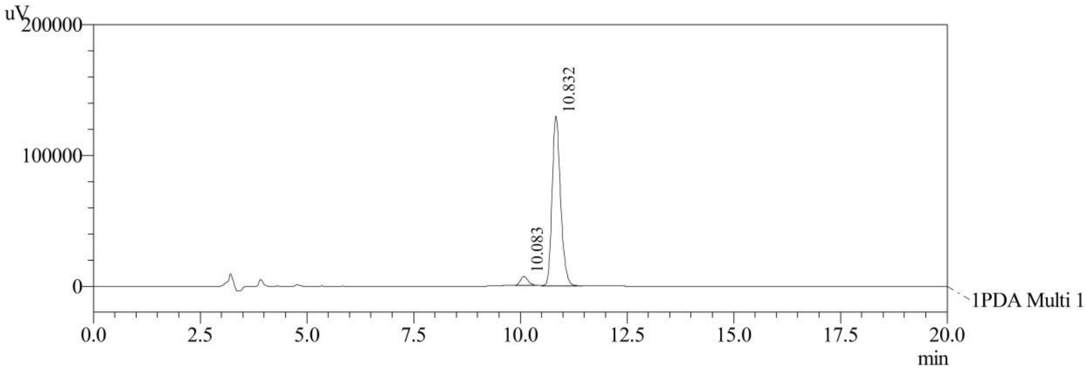

1 PDA Multi 1 / 210nm 4nm

PeakTable

PDA Ch1 210nm 4nm

Peak#	Ret. Time	Area	Height	Area %	Height %
1	10.083	85823	6876	4.585	5.022
2	10.832	1785884	130030	95.415	94.978
Total		1871707	136906	100.000	100.000

(R)-phenethyl(1,1,1-trifluoro-3-nitro-2-(p-tolyl)propan-2-yl)sulfane (3ac)

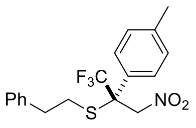

3ac

The title compound was prepared according to the general procedure A and purified by flash column chromatography (50:1 hexanes : EtOAc) to afford **3ac** (36 mg, 98%) as a colorless oil. Analytical data: IR (KBr, cm^{-1}) 2923, 2315, 1560, 1454, 1369, 1232, 1150, 1029, 804, 698; ^{1}H NMR (400 MHz, $CDCl_3$) δ 7.52 (d, *J* = 8.0 Hz, 2H), 7.37 – 7.19 (m, 5H), 7.12 (d, *J* = 7.0 Hz, 2H), 5.05 (s, 2H), 2.93 – 2.72 (m, 3H), 2.71 – 2.60 (m, 1H), 2.40 (s, 3H); ^{19}F NMR (376 MHz, $CDCl_3$) δ -66.62 (s, 3F); ^{13}C NMR (100 MHz, $CDCl_3$) δ 139.32 (s), 129.63 (s), 128.56 (s), 128.47 (s), 128.31 (s), 128.01 (s), 127.99 (s), 126.69 (s), 125.95 (q, *J* = 283.0 Hz), 77.73 (s), 59.11 (q, *J* = 27.0 Hz), 34.82 (s), 32.09 (s), 21.03 (s). HPLC (OJ-H, 5% EtOH in hexanes, 1 mL/min, 210 nm): t_{major} = 31.1 min, t_{minor} = 28.2 min, 92% ee; $^{25}[\alpha]_D$ = -16.1 ^{o} (c = 1.0 in $CHCl_3$); HRMS (ESI+) Calcd for $C_{18}H_{18}F_3NO_2SNa^+$ $(M+Na)^+$: 392.0908, Found: 392.0904.

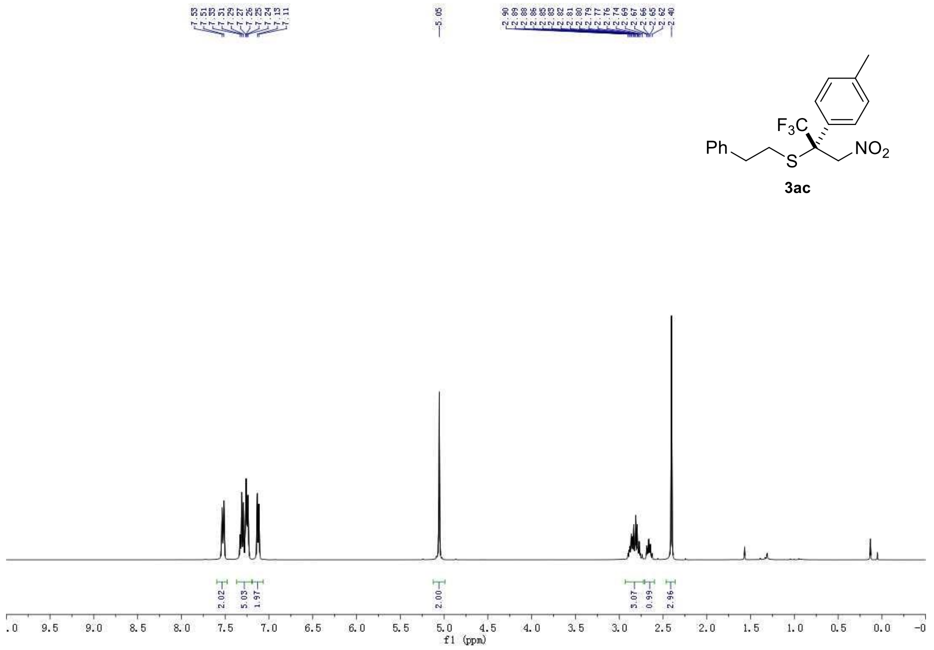

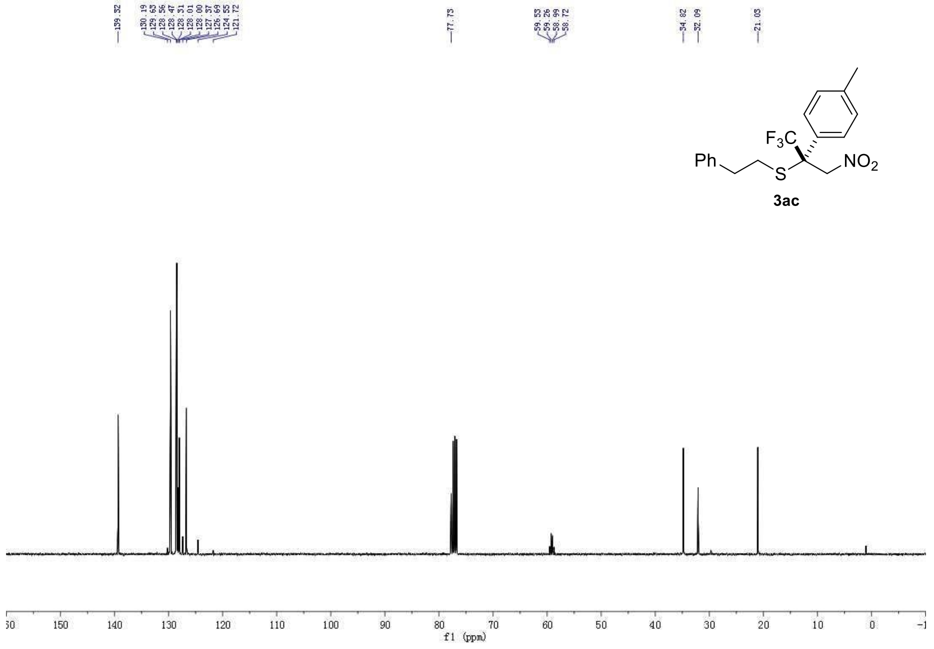

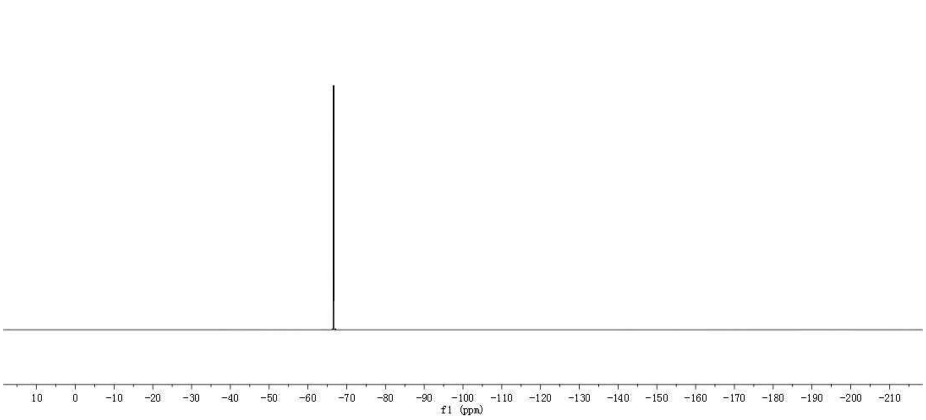

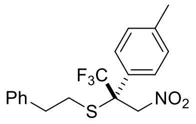

3ac

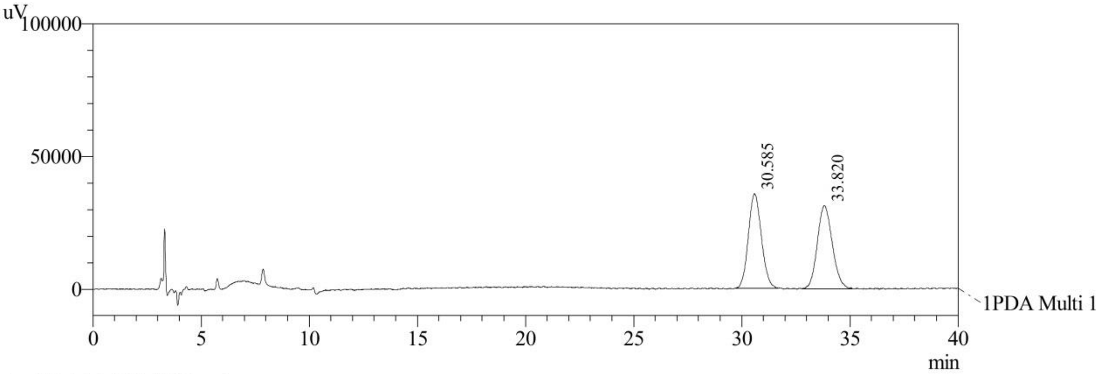

1 PDA Multi 1 / 210nm 4nm

PeakTable

PDA Ch1 210nm 4nm

Peak#	Ret. Time	Area	Height	Area %	Height %
1	30.585	1500889	35673	49.927	53.173
2	33.820	1505270	31415	50.073	46.827
Total		3006159	67088	100.000	100.000

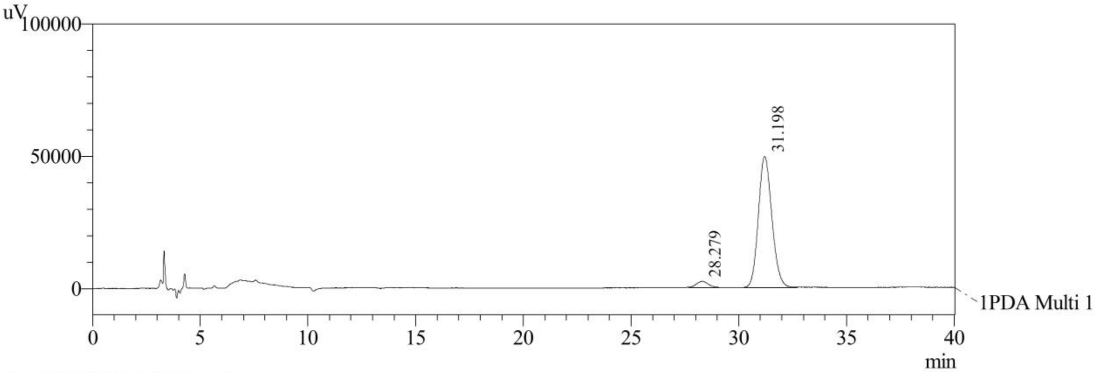

1 PDA Multi 1 / 210nm 4nm

PeakTable

PDA Ch1 210nm 4nm

Peak#	Ret. Time	Area	Height	Area %	Height %
1	28.279	88737	2325	3.922	4.473
2	31.198	2174054	49664	96.078	95.527
Total		2262791	51990	100.000	100.000

(R)-phenethyl(1,1,1-trifluoro-2-(4-methoxyphenyl)-3-nitropropan-2-yl)sulfane (3ad)

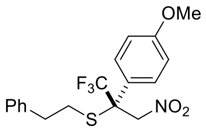

3ad

The title compound was prepared according to the general procedure A and purified by flash column chromatography (50:1 hexanes : EtOAc) to afford **3ad** (37 mg, 95%) as a colorless oil. Analytical data: IR (KBr, cm^{-1}) 2929, 2303, 1565, 1517, 1507, 1374, 1260, 1218, 1147, 1033, 823, 698; ^{1}H NMR (400 MHz, $CDCl_3$) δ 7.55 (d, *J* = 8.8 Hz, 2H), 7.41 – 7.21 (m, 3H), 7.21 – 7.07 (m, 2H), 7.07 – 6.88 (m, 2H), 5.03 (s, 2H), 3.84 (s, 3H), 2.84 (dtd, *J* = 16.0, 9.3, 5.6 Hz, 3H), 2.67 (dd, *J* = 10.2, 5.3 Hz, 1H); ^{19}F NMR (376 MHz, $CDCl_3$) δ -66.83 (s, 3F); ^{13}C NMR (100 MHz, $CDCl_3$) δ 159.88 (s), 139.31 (s), 129.54 (s), 128.56 (s), 128.47 (s), 126.69 (s), 125.96 (q, *J* = 282.0 Hz), 122.94 (s), 114.20 (s), 77.74 (s), 58.97(q, *J* = 28.0 Hz), 55.31 (s), 34.80 (s), 32.11 (s). HPLC (AD-H, 5% EtOH in hexanes, 1 mL/min, 210 nm): t_{major} = 10.0 min, t_{minor} = 11.7 min, 96% ee; $^{25}[\alpha]_D$ = -6.5 ^{o} (c = 1.0 in $CHCl_3$); HRMS (ESI+) Calcd for $C_{18}H_{18}F_3NO_3SNa^+$ $(M+Na)^+$: 408.0857, Found: 408.0850.

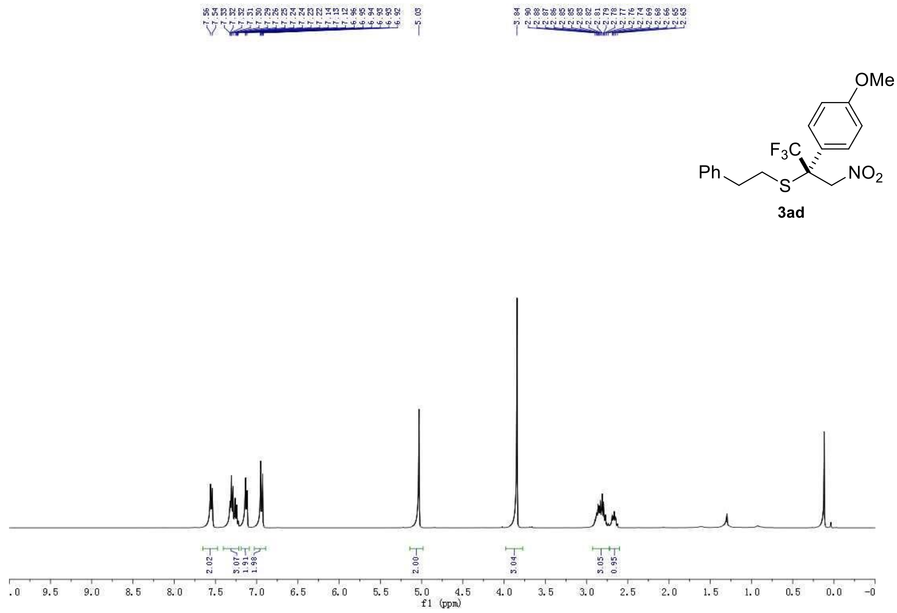

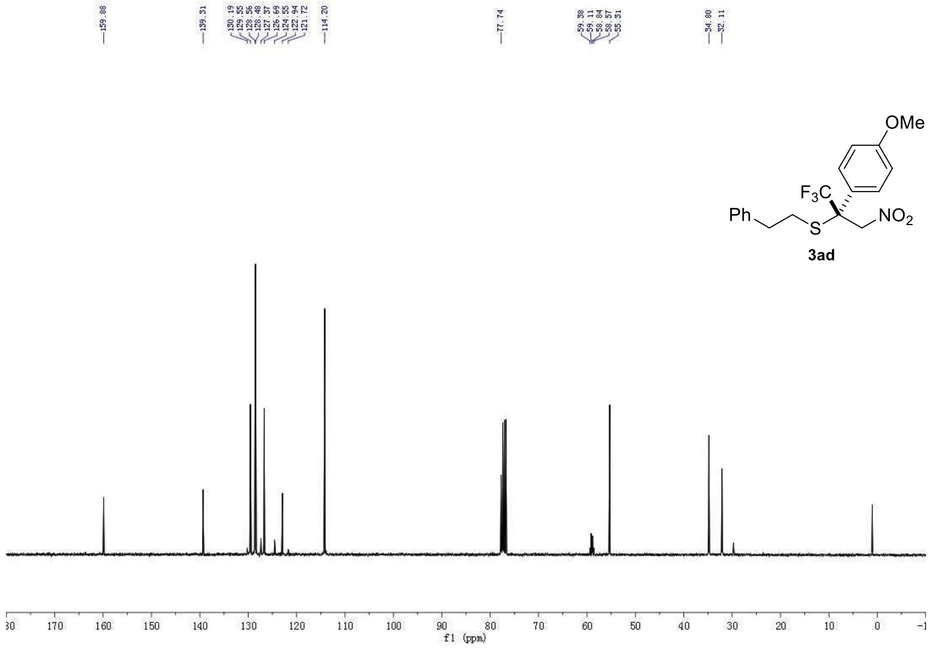

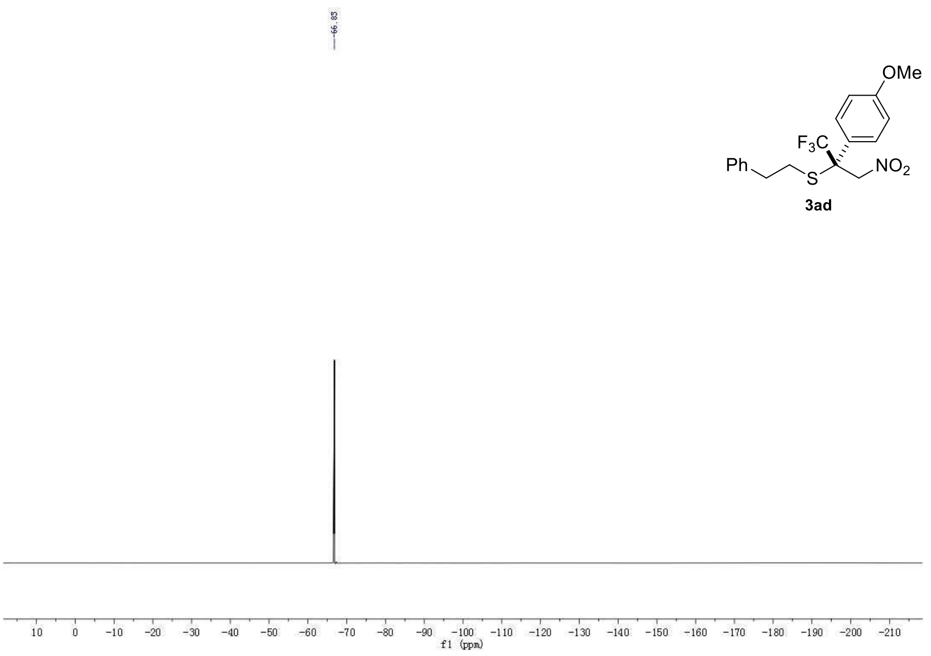

3ad

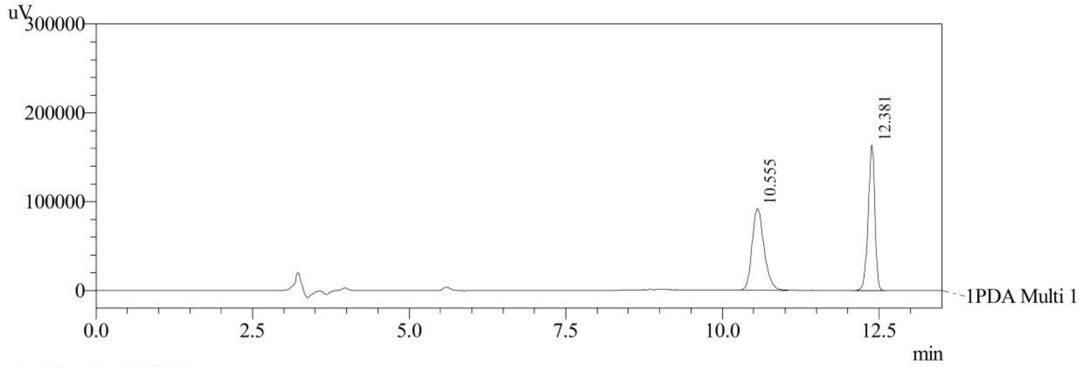

1 PDA Multi 1 / 210nm 4nm

PeakTable

PDA Ch1 210nm 4nm

Peak#	Ret. Time	Area	Height	Area %	Height %
1	10.555	1224412	92069	50.175	35.966
2	12.381	1215884	163916	49.825	64.034
Total		2440295	255985	100.000	100.000

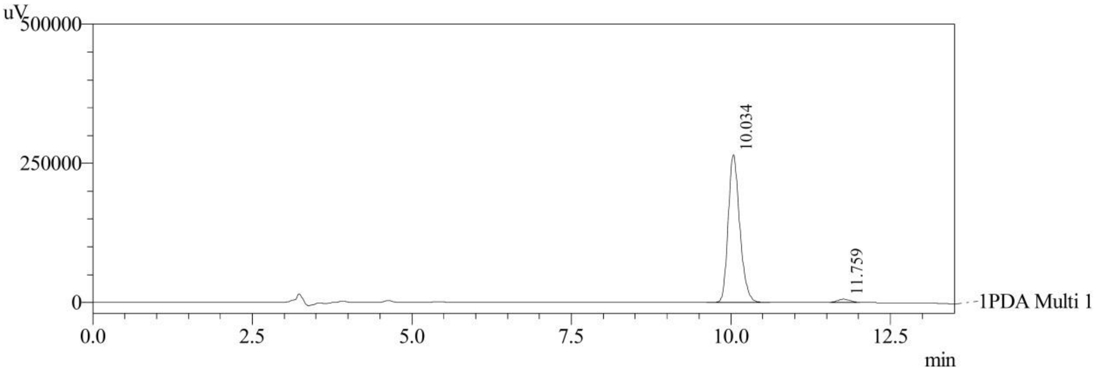

1 PDA Multi 1 / 210nm 4nm

PeakTable

PDA Ch1 210nm 4nm

Peak#	Ret. Time	Area	Height	Area %	Height %
1	10.034	3336751	265924	97.906	97.923
2	11.759	71366	5639	2.094	2.077
Total		3408117	271563	100.000	100.000

(R)-(2-(3,5-dimethylphenyl)-1,1,1-trifluoro-3-nitropropan-2-yl)(phenethyl)sulfane (3ag)

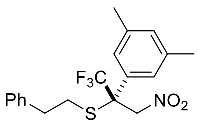

3ag

The title compound was prepared according to the general procedure A and purified by flash column chromatography (50:1 hexanes : EtOAc) to afford **3ag** (37 mg, 96%) as a colorless oil. Analytical data: IR (KBr, cm^{-1}) 2921, 2327, 1603, 1564, 1454, 1369, 1216, 1151, 1048, 841, 742, 699; ^{1}H NMR (400 MHz, $CDCl_3$) δ 7.36 – 7.29 (m, 2H), 7.28 – 7.22 (m, 3H), 7.16 – 7.09 (m, 2H), 7.06 (s, 1H), 5.14 – 4.99 (m, 2H), 2.92 – 2.74 (m, 3H), 2.73 – 2.60 (m, 1H), 2.37 (s, 6H); ^{19}F NMR (376 MHz, $CDCl_3$) δ -66.21 (s, 3F); ^{13}C NMR (100 MHz, $CDCl_3$) δ 139.37 (s), 138.48 (s), 131.27 (s), 130.93 (s), 128.57 (s), 128.44 (s), 126.68 (s), 125.96 (q, *J* = 283 Hz), 125.80 (d, *J* = 1.4 Hz), 77.81 (s), 59.20 (q, *J* = 27.0 Hz), 40.27 (s), 34.80 (s), 32.11 (s), 21.55 (s). HPLC (OJ-H, 5% EtOH in hexanes, 1 mL/min, 210 nm): t_{major} = 12.9 min, t_{minor} = 14.1 min, 92% ee; $^{25}[\alpha]_D$ = -15.5 º (c = 1.0 in $CHCl_3$); HRMS (ESI+) Calcd for $C_{19}H_{20}F_3NO_2SNa^+$ $(M+Na)^+$: 406.1065, Found: 406.1062.

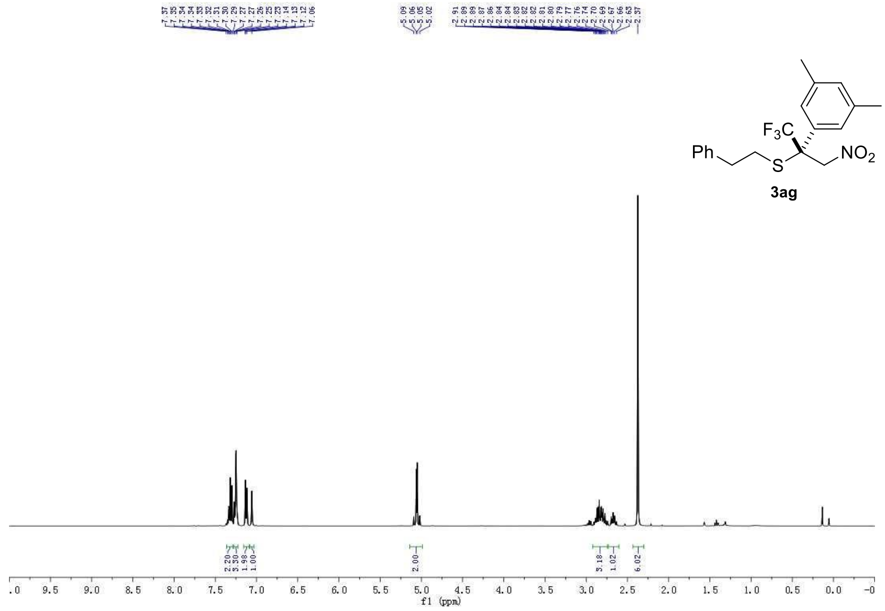

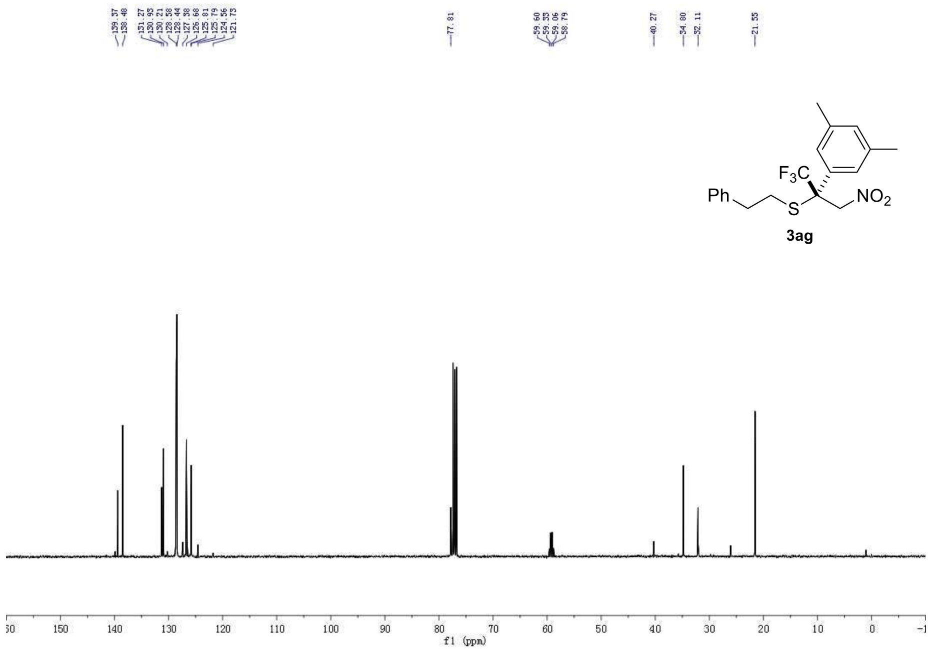

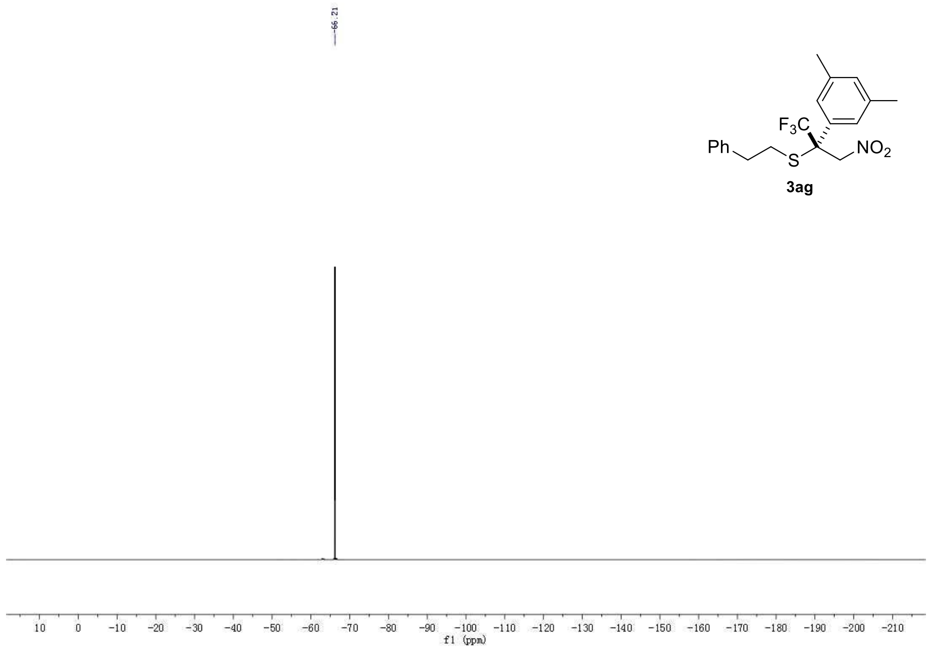

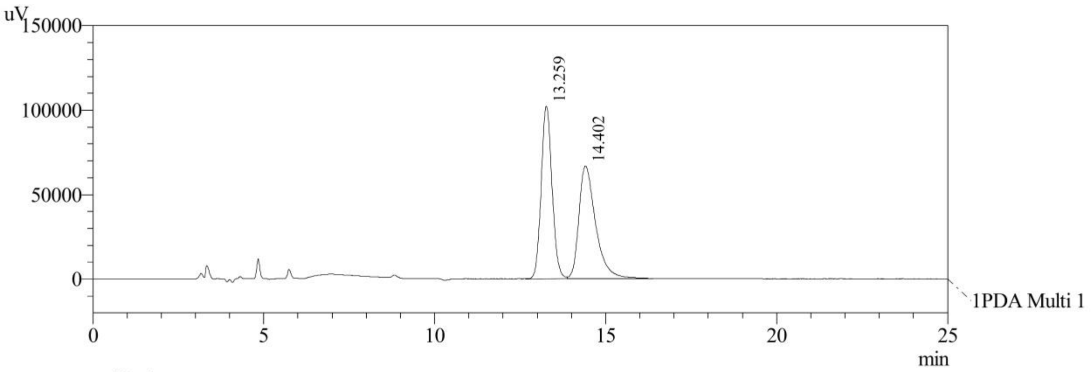

1 PDA Multi 1 / 210nm 4nm

PeakTable

PDA Ch1 210nm 4nm

Peak#	Ret. Time	Area	Height	Area %	Height %
1	13.259	2264406	102242	49.968	60.472
2	14.402	2267332	66831	50.032	39.528
Total		4531738	169073	100.000	100.000

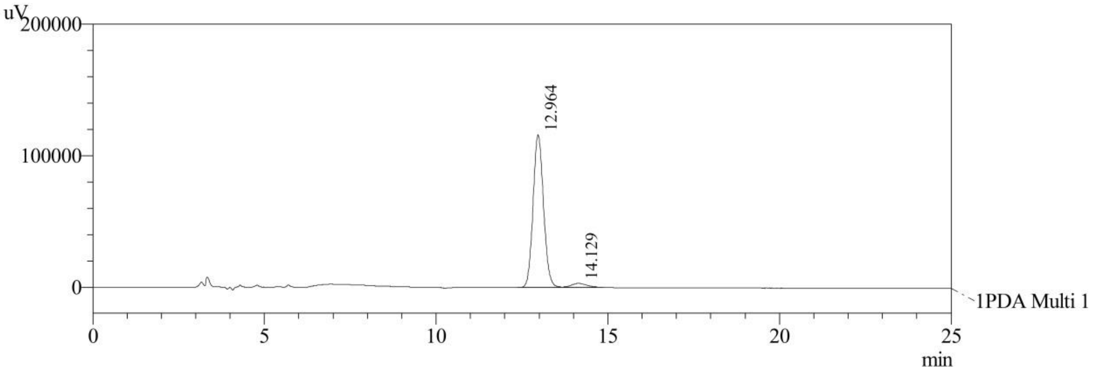

1 PDA Multi 1 / 210nm 4nm

PeakTable

PDA Ch1 210nm 4nm

Peak#	Ret. Time	Area	Height	Area %	Height %
1	12.964	2469054	116370	96.094	97.379
2	14.129	100354	3132	3.906	2.621
Total		2569407	119502	100.000	100.000

(R)-phenethyl(1,1,1-trifluoro-2-(3-methoxyphenyl)-3-nitropropan-2-yl)sulfane (3ah)

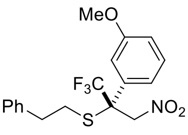

3ah

The title compound was prepared according to the general procedure A and purified by flash column chromatography (50:1 hexanes : EtOAc) to afford **3ah** (37 mg, 95%) as a colorless oil. Analytical data: IR (KBr, cm^{-1}) 2917, 2312, 1654, 1559, 1507, 1490, 1457, 1368, 1193, 1143, 695; ^{1}H NMR (400 MHz, $CDCl_3$) δ 7.40 – 7.34 (m, 1H), 7.34 – 7.27 (m, 2H), 7.27 – 7.20 (m, 3H), 7.17 – 7.08 (m, 2H), 6.95 (ddd, *J* = 8.3, 2.3, 0.7 Hz, 1H), 5.13 – 4.96 (m, 2H), 3.83 (s, 3H), 2.93 – 2.71 (m, 3H), 2.66 (td, *J* = 9.8, 6.3 Hz, 1H); ^{19}F NMR (376 MHz, $CDCl_3$) δ -66.28 (s, 3F); ^{13}C NMR (100 MHz, $CDCl_3$) δ 159.80 (s), 139.25 (s), 132.96 (s),129.93 (s), 128.58 (s), 128.44 (s), 126.71 (s), 125.87 (q, *J* = 283.0 Hz), 120.25 (s), 114.82 (d, *J* = 1.6 Hz), 114.06 (s), 77.81 (s), 59.20 (q, *J* = 27.0 Hz), 55.35 (s), 34.78 (s), 32.13 (s). HPLC (OJ-H, 20% EtOH in hexanes, 1 mL/min, 210 nm): t_{major} = 20.3 min, t_{minor} = 18.2 min, 86% ee; $^{25}[\alpha]_D$ = -15.1 ° (c = 1.0 in $CHCl_3$); HRMS (ESI+) Calcd for $C_{18}H_{18}F_3NO_3SNa^+$ $(M+Na)^+$: 408.0857, Found: 408.0851.

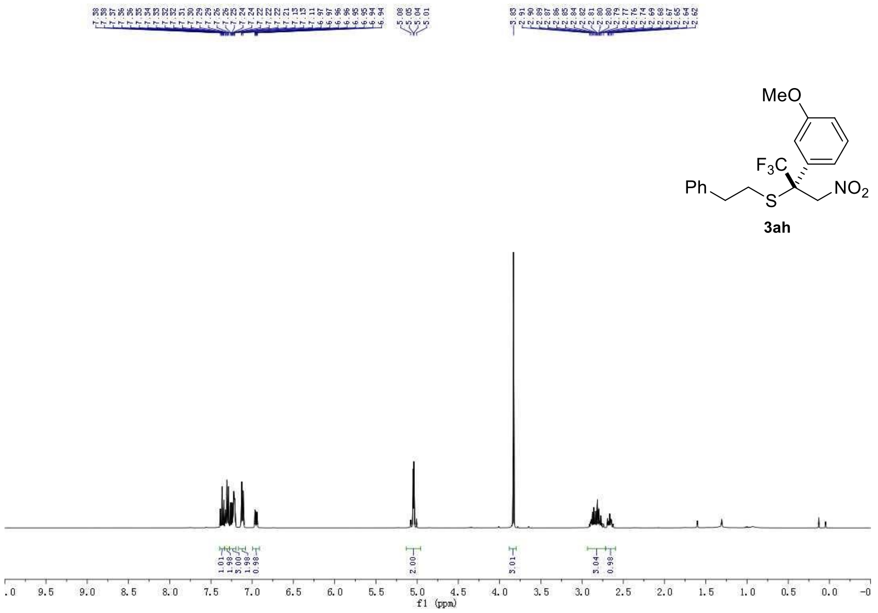

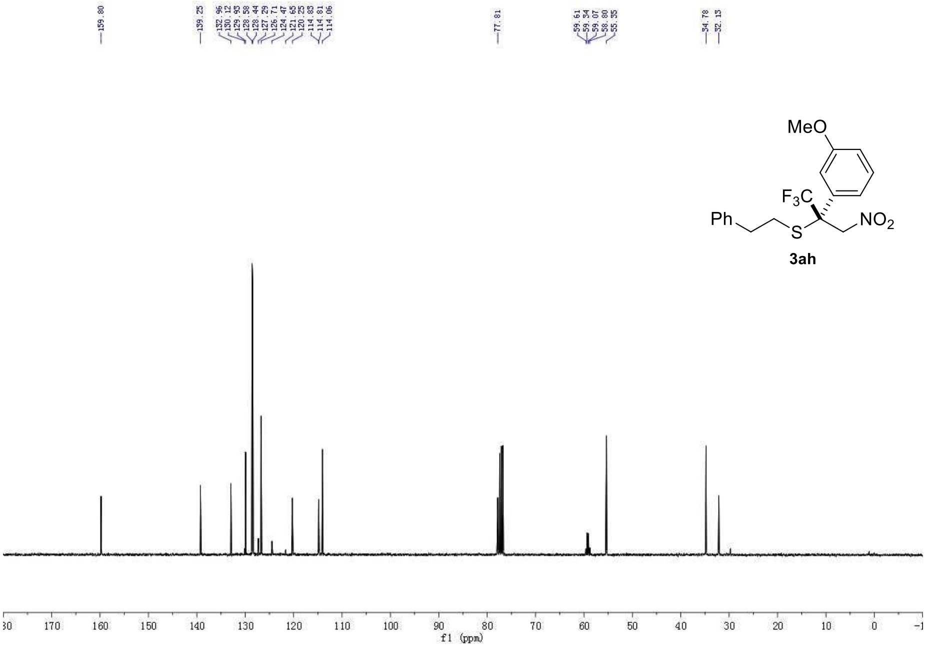

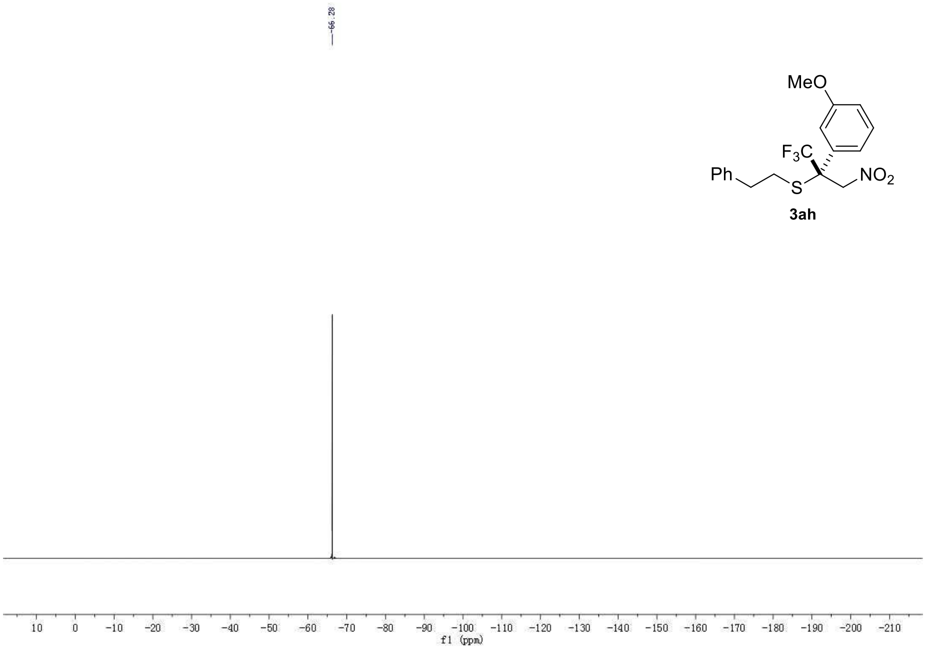

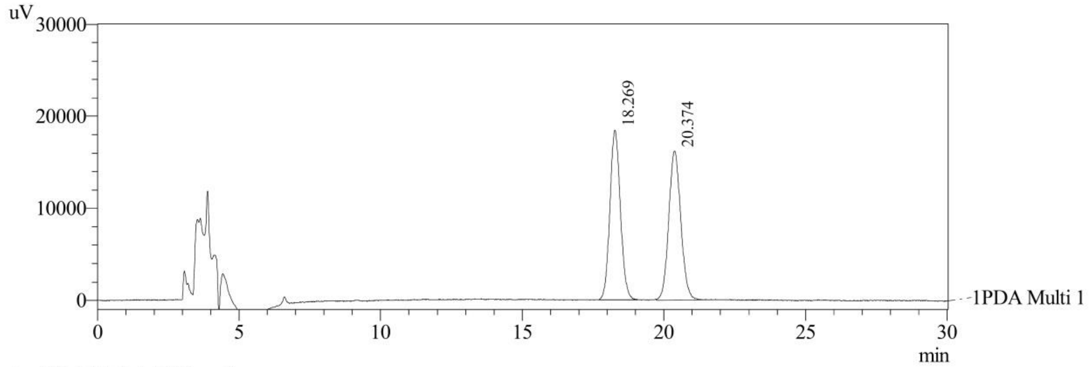

1 PDA Multi 1 / 210nm 4nm

PeakTable

PDA Ch1 210nm 4nm

Peak#	Ret. Time	Area	Height	Area %	Height %
1	18.269	477020	18458	49.951	53.325
2	20.374	477950	16156	50.049	46.675
Total		954970	34613	100.000	100.000

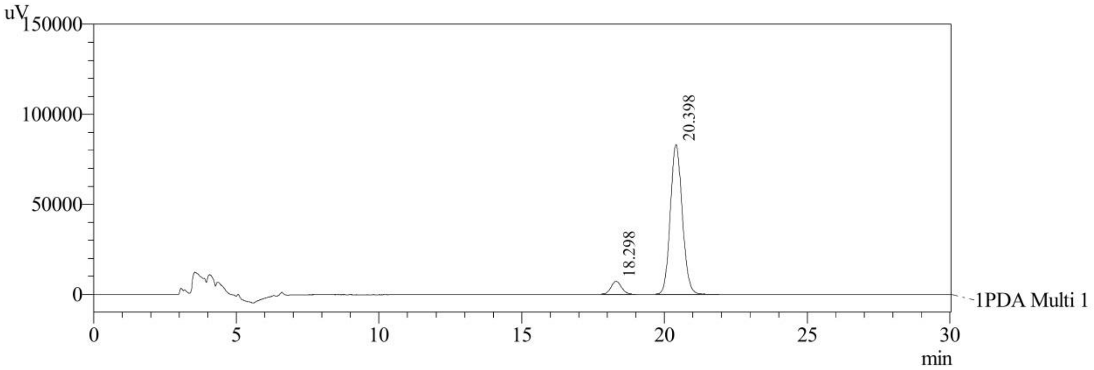

1 PDA Multi 1 / 210nm 4nm

PeakTable

PDA Ch1 210nm 4nm

Peak#	Ret. Time	Area	Height	Area %	Height %
1	18.298	182325	7207	6.980	7.973
2	20.398	2429923	83192	93.020	92.027
Total		2612248	90399	100.000	100.000

(R)-phenethyl(1,1,1-trifluoro-2-(naphthalen-2-yl)-3-nitropropan-2-yl)sulfane (3ai)

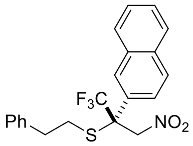

3ai

The title compound was prepared according to the general procedure A and purified by flash column chromatography (50:1 hexanes : EtOAc) to afford **3ai** (40 mg, 98%) as a colorless oil. Analytical data: IR (KBr, cm^{-1}) 3029, 2312, 1563, 1368, 1217, 1151, 813, 699; ^1H NMR (400 MHz, $CDCl_3$) δ 8.09 (s, 1H), 7.89 (ddd, *J* = 11.7, 10.3, 6.6 Hz, 3H), 7.74 (dd, *J* = 8.8, 1.8 Hz, 1H), 7.68 – 7.52 (m, 2H), 7.37 – 7.19 (m, 3H), 7.16 – 6.98 (m, 2H), 5.23 – 5.06 (m, 2H), 2.96 – 2.69 (m, 3H), 2.68 – 2.54 (m, 1H); ^{19}F NMR (376 MHz, $CDCl_3$) δ -66.14 (s, 3F); ^{13}C NMR (100 MHz, $CDCl_3$) δ 139.15 (s), 132.94 (s), 132.71 (s), 128.94 (s), 128.75 (s), 128.56 (s), 128.52 (s), 128.45 (s), 128.24 (d, *J* = 2.0 Hz), 127.59 (s), 127.51 (s), 126.97 (s), 126.72 (s), 126.07 (q, *J* = 282.0 Hz), 124.66 (s), 77.75 (s), 59.55 (q, *J* = 27.0 Hz), 34.74 (s), 32.16 (s). HPLC (AD-H, 5% EtOH in hexanes, 1 mL/min, 210 nm): t_{major} = 8.9 min, t_{minor} = 9.6 min, 94% ee; $^{25}[\alpha]_D$ = +35.5 ° (c = 1.0 in $CHCl_3$); HRMS (ESI+) Calcd for $C_{21}H_{18}F_3NO_2SNa^+$ $(M+Na)^+$: 428.0908, Found: 428.0903.

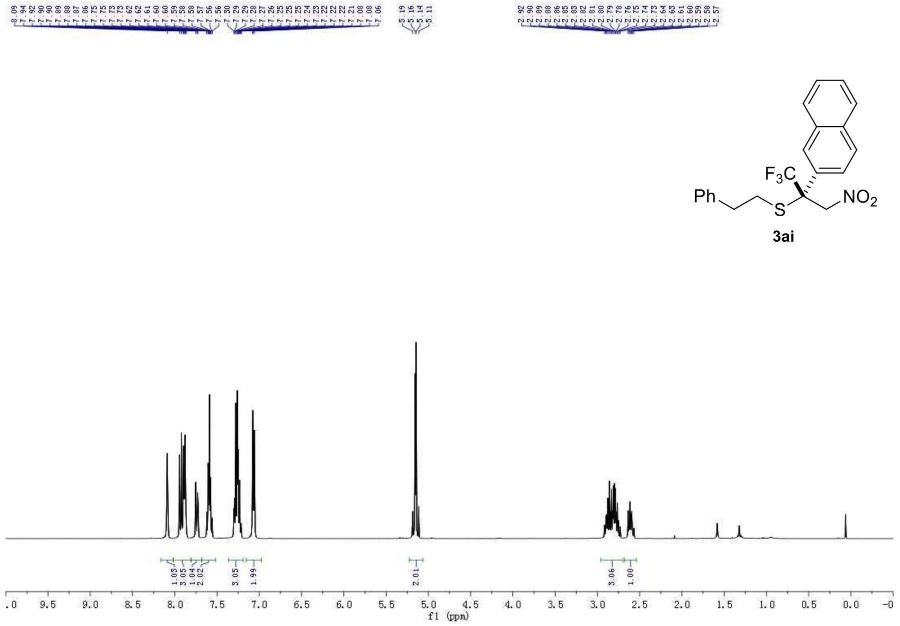

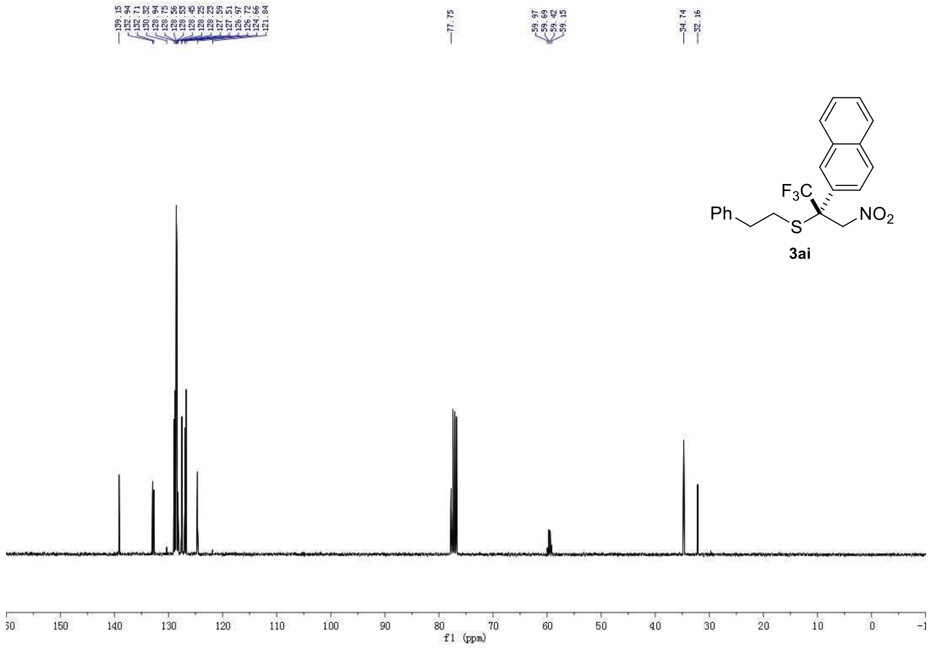

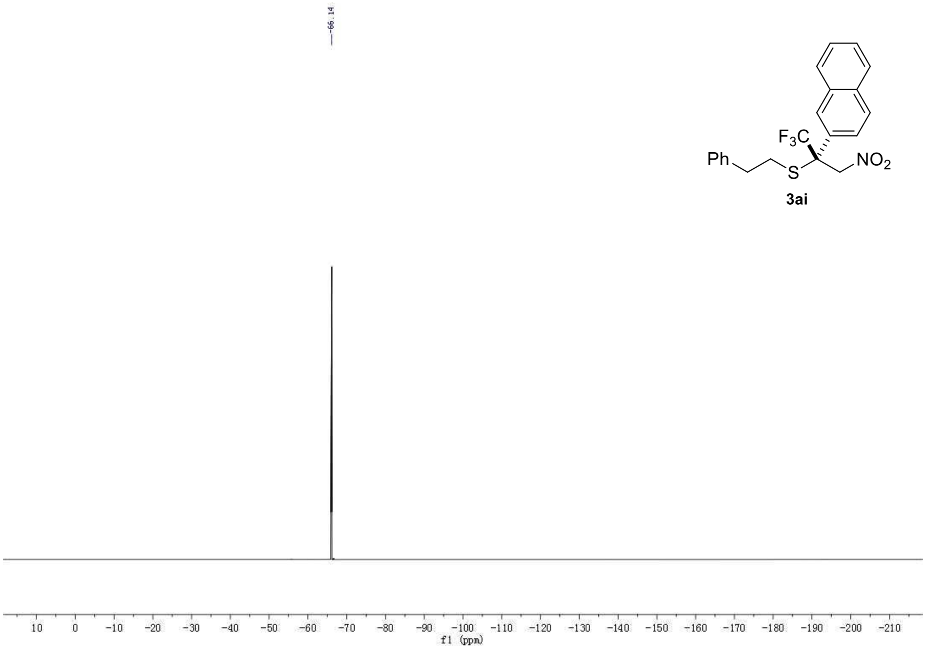

3ai

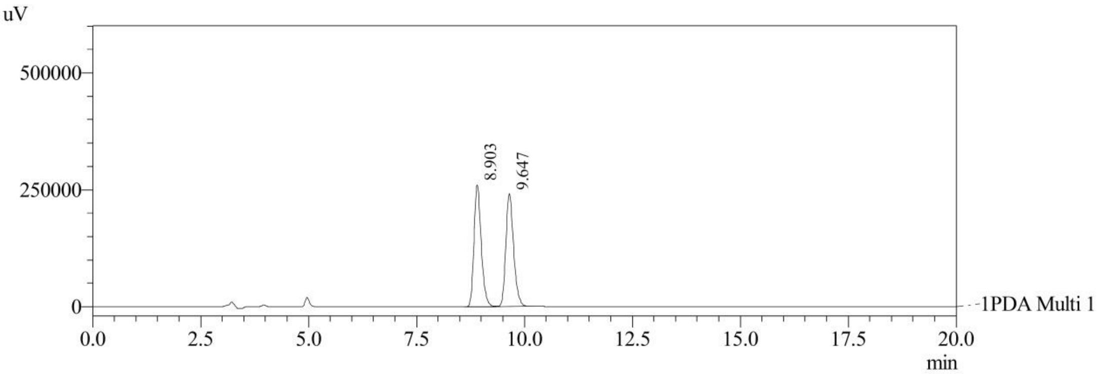

1 PDA Multi 1 / 210nm 4nm

PeakTable

PDA Ch1 210nm 4nm

Peak#	Ret. Time	Area	Height	Area %	Height %
1	8.903	3018439	260078	49.925	51.848
2	9.647	3027497	241537	50.075	48.152
Total		6045936	501615	100.000	100.000

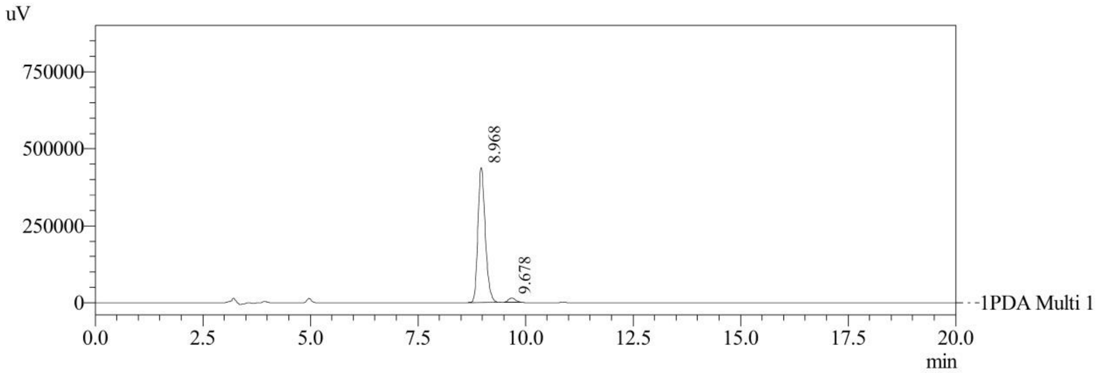

1 PDA Multi 1 / 210nm 4nm

PeakTable

PDA Ch1 210nm 4nm

Peak#	Ret. Time	Area	Height	Area %	Height %
1	8.968	5144763	438712	97.046	96.914
2	9.678	156588	13968	2.954	3.086
Total		5301351	452680	100.000	100.000

(R)-2-(1,1,1-trifluoro-3-nitro-2-(phenethylthio)propan-2-yl)thiophene (3aj)

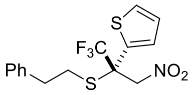

3aj

The title compound was prepared according to the general procedure A and purified by flash column chromatography (50:1 hexanes : EtOAc) to afford **3aj** (35 mg, 98%) as a colorless oil. Analytical data: IR (KBr, cm^{-1}) 3029, 2921, 1560, 1469, 1454, 1430, 1368, 1213, 1160, 740, 699; ^{1}H NMR (400 MHz, $CDCl_3$) δ 7.45 (dd, *J* = 5.2, 1.1 Hz, 1H), 7.37 – 7.22 (m, 4H), 7.20 – 7.12 (m, 2H), 7.04 (dd, *J* = 5.2, 3.8 Hz, 1H), 5.00 (s, 2H), 3.06 – 2.93 (m, 1H), 2.93 – 2.80 (m, 3H); ^{19}F NMR (376 MHz, $CDCl_3$) δ -68.40 (s, 3F); ^{13}C NMR (100 MHz, $CDCl_3$) δ 139.19 (s), 135.66 (s), 129.08 (d, *J* = 2.0 Hz), 128.62 (s), 128.47 (s), 127.95 (s), 127.21 (s), 126.77 (s), 125.26 (q, *J* = 283.0 Hz), 79.00 (s), 56.96 (q, *J* = 29.0 Hz), 34.78 (s), 32.57 (s). HPLC (AD-H, 5% EtOH in hexanes, 0.8 mL/min, 210 nm): t_{major} = 11.5 min, t_{minor} = 10.9 min, 80% ee; $^{25}[\alpha]_D$ = -25.7 ° (c = 1.0 in $CHCl_3$); HRMS (ESI+) Calcd for $C_{15}H_{14}F_3NO_2S_2Na^+$ $(M+Na)^+$: 384.0316, Found: 384.0308.

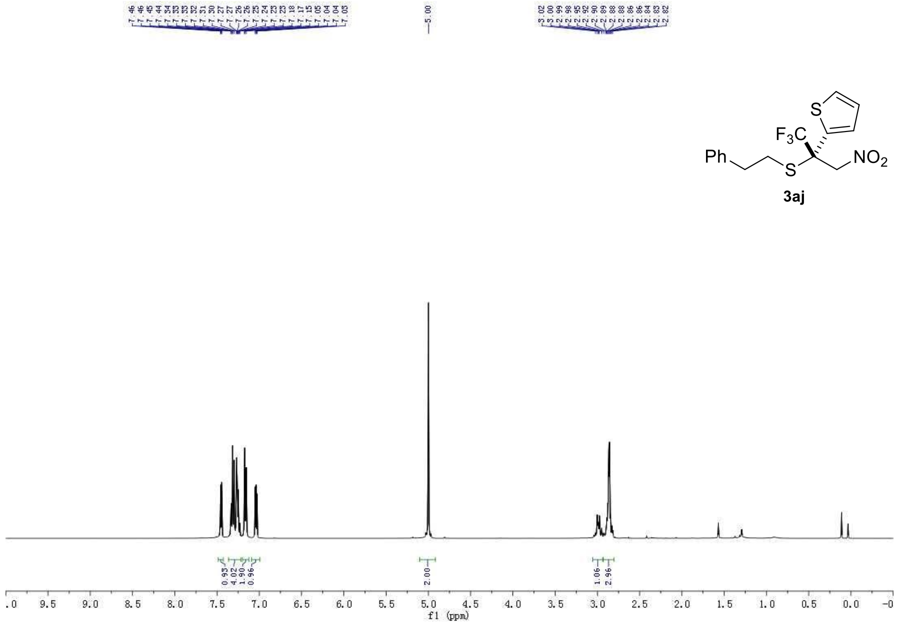

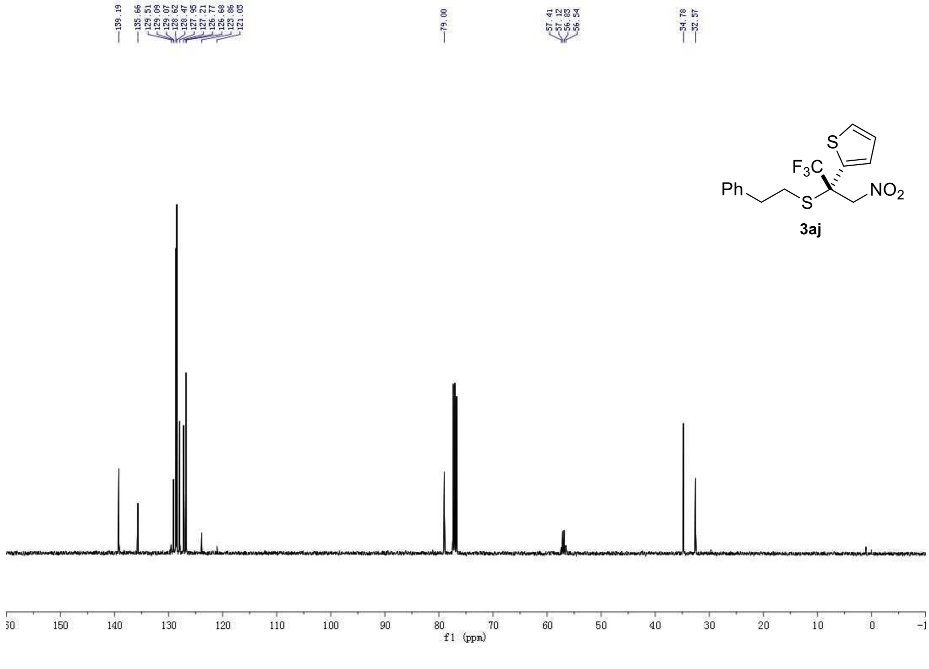

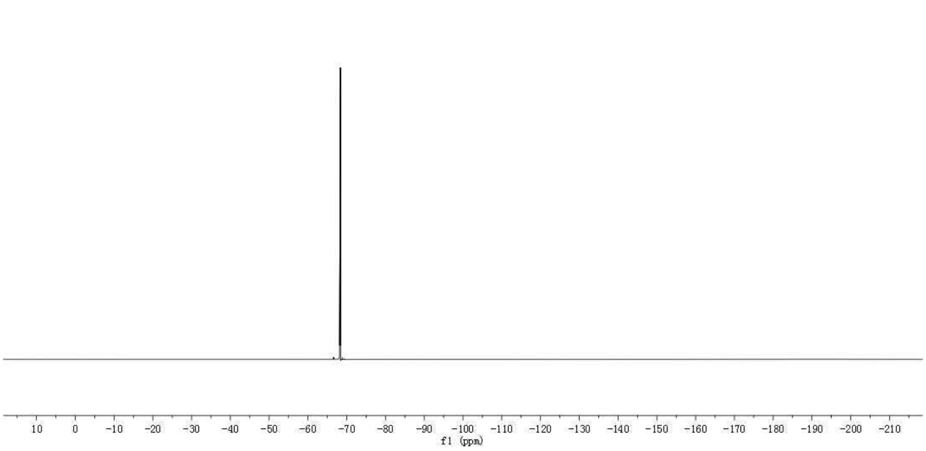

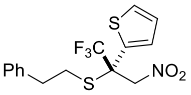

3aj

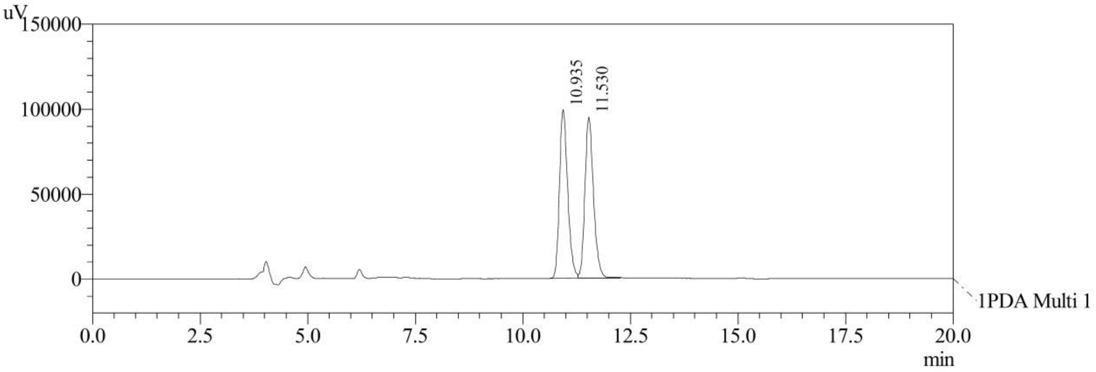

1 PDA Multi 1 / 210nm 4nm

PeakTable

PDA Ch1 210nm 4nm

Peak#	Ret. Time	Area	Height	Area %	Height %
1	10.935	1310859	99569	49.869	51.213
2	11.530	1317737	94853	50.131	48.787
Total		2628596	194422	100.000	100.000

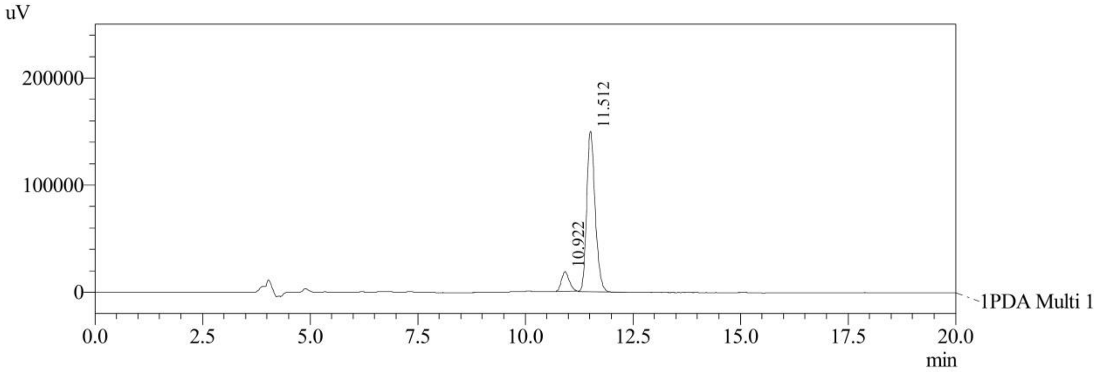

1 PDA Multi 1 / 210nm 4nm

PeakTable

PDA Ch1 210nm 4nm

Peak#	Ret. Time	Area	Height	Area %	Height %
1	10.922	226421	18145	9.903	10.790
2	11.512	2059869	150028	90.097	89.210
Total		2286290	168173	100.000	100.000

(R)-(2-nitro-1-phenylethyl)(phenethyl)sulfane (5ak)

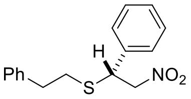

5ak

The title compound was prepared according to the general procedure A and purified by flash column chromatography (50:1 hexanes : EtOAc) to afford **5ak** (28 mg, 98%) as a colorless oil. Analytical data: IR (KBr, cm^{-1}) 3028, 2919, 1602, 1558, 1495, 1455, 1374, 1267, 1180, 1079, 1030, 749, 697; ^{1}H NMR (400 MHz, $CDCl_3$) δ 7.52 – 7.22 (m, 8H), 7.20 – 7.09 (m, 2H), 4.74 (dd, *J* = 7.5, 5.5 Hz, 2H), 4.57 (dd, *J* = 8.5, 7.1 Hz, 1H), 2.85 (dd, *J* = 11.4, 4.8 Hz, 2H), 2.79 – 2.65 (m, 2H); ^{13}C NMR (100 MHz, $CDCl_3$) δ 139.81 (s), 137.29 (s), 129.09 (s), 128.59 (s), 128.57 (s), 128.54 (s), 127.76 (s), 126.63 (s), 79.24 (s), 46.71 (s), 35.92 (s), 33.04 (s). HPLC (AD-H, 5% EtOH in hexanes, 1 mL/min, 210 nm): t_{major} = 10.7 min, t_{minor} = 12.9 min, 10% ee; $^{25}[\alpha]_D$ = +6.9 ^{o} (c = 1.0 in $CHCl_3$); HRMS (ESI+) Calcd for $C_{16}H_{17}NO_2NaS^+$ $(M+Na)^+$: 310.0878, Found: 310.0873.

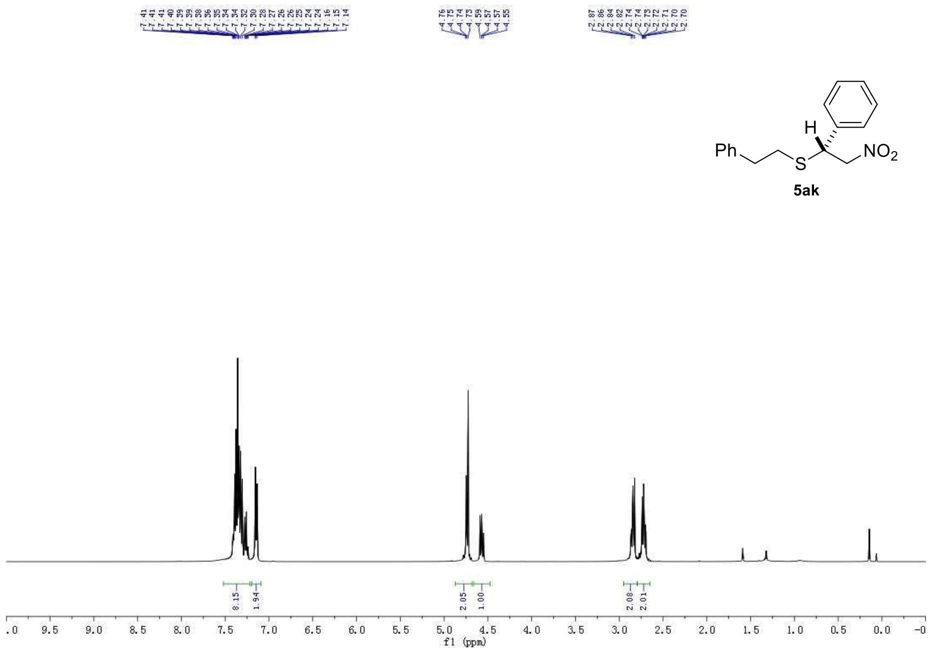

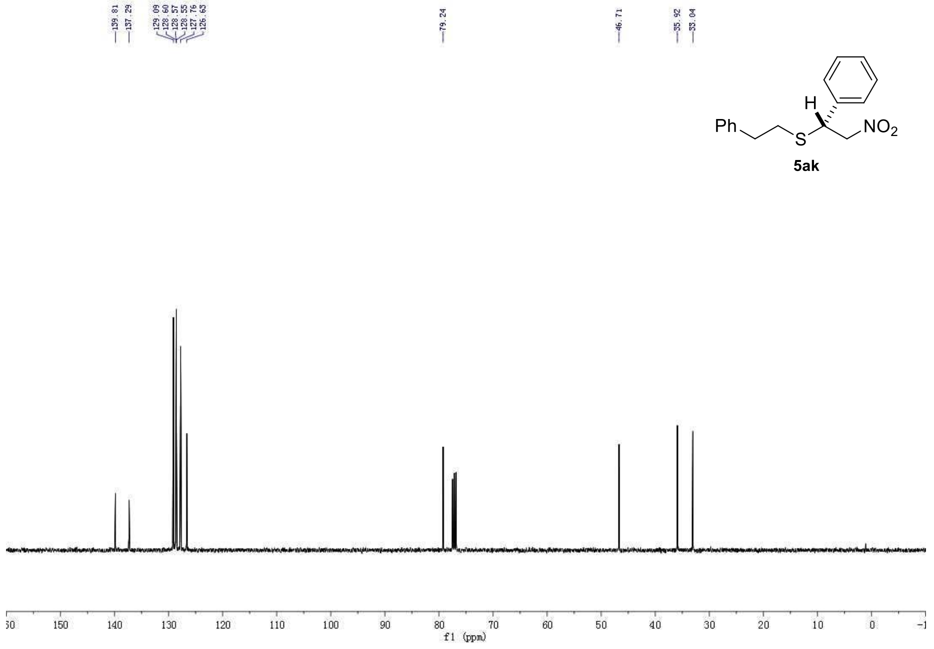

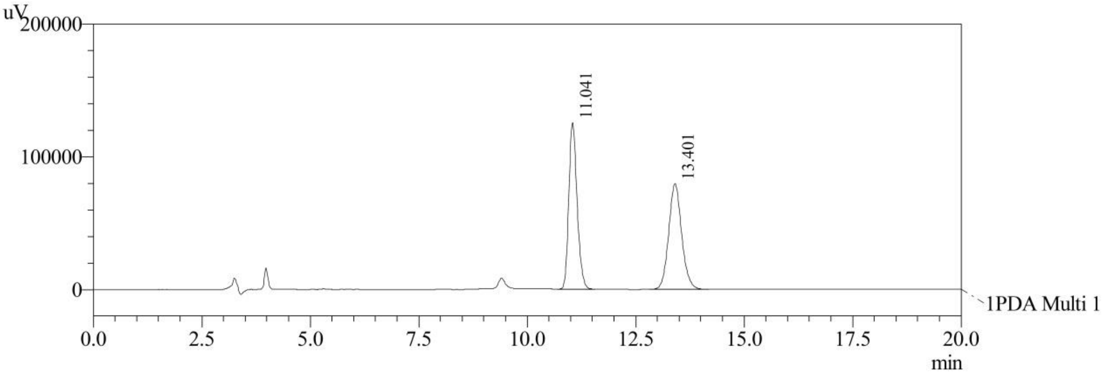

1 PDA Multi 1 / 210nm 4nm

PeakTable

PDA Ch1 210nm 4nm

Peak#	Ret. Time	Area	Height	Area %	Height %
1	11.041	1653445	125383	49.891	61.183
2	13.401	1660692	79549	50.109	38.817
Total		3314138	204931	100.000	100.000

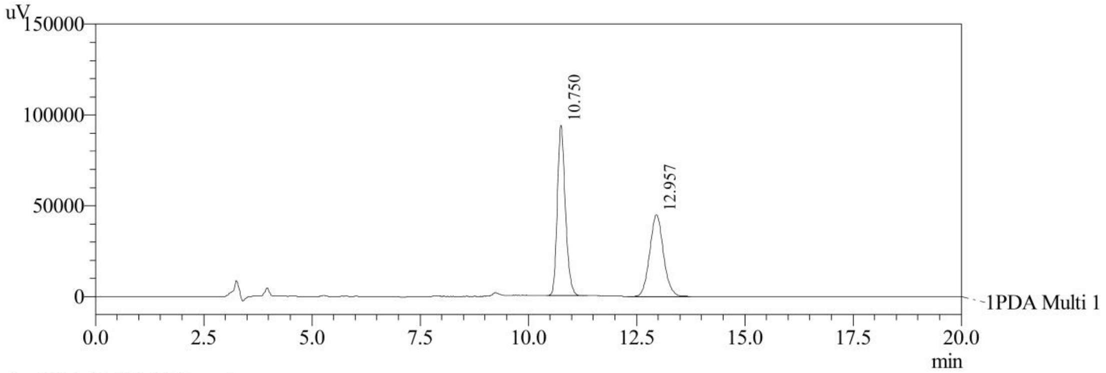

1 PDA Multi 1 / 210nm 4nm

PeakTable

PDA Ch1 210nm 4nm

Peak#	Ret. Time	Area	Height	Area %	Height %
1	10.750	1209333	93702	54.901	67.502
2	12.957	993433	45112	45.099	32.498
Total		2202766	138814	100.000	100.000

(R)-(1-nitro-2-phenylpropan-2-yl)(phenethyl)sulfane (5al)

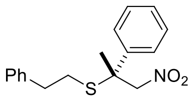

5al

The title compound was prepared according to the general procedure A and purified by flash column chromatography (50:1 hexanes : EtOAc) to afford **5al** (7 mg, 22%) as a colorless oil. Analytical data: IR (KBr, cm^{-1}) 3027, 2925, 1601, 1558, 1495, 1446, 1382, 1268, 1072, 1030, 748, 697; ^{1}H NMR (400 MHz, $CDCl_3$) δ 7.56 – 7.49 (m, 2H), 7.44 – 7.37 (m, 2H), 7.37 – 7.20 (m, 4H), 7.13 – 7.05 (m, 2H), 5.00 (d, *J* = 11.8 Hz, 1H), 4.76 (d, *J* = 11.8 Hz, 1H), 2.71 (dd, *J* = 11.5, 4.8 Hz, 2H), 2.59 (ddd, *J* = 8.4, 5.8, 1.1 Hz, 2H), 2.00 (s, 3H); ^{13}C NMR (101 MHz, $CDCl_3$) δ 140.34 (s), 139.94 (s), 128.72 (s), 128.51 (s), 128.42 (s), 127.89 (s), 126.62 (s), 126.54 (s), 84.07 (s), 50.07 (s), 35.33 (s), 30.92 (s), 25.40 (s). HPLC (AD-H, 5% EtOH in hexanes, 1 mL/min, 210 nm): t_{major} = 11.6 min, t_{minor} = 10.2 min, 16% ee; HRMS (ESI+) Calcd for $C_{16}H_{17}NO_2NaS^+$ $(M+Na)^+$: 324.1034, Found: 324.1027.

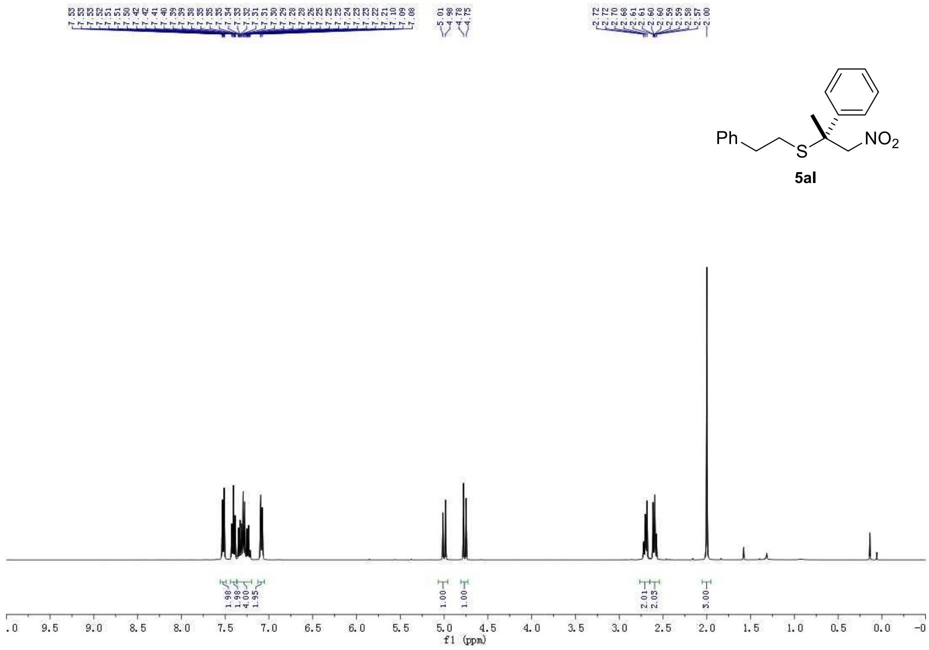

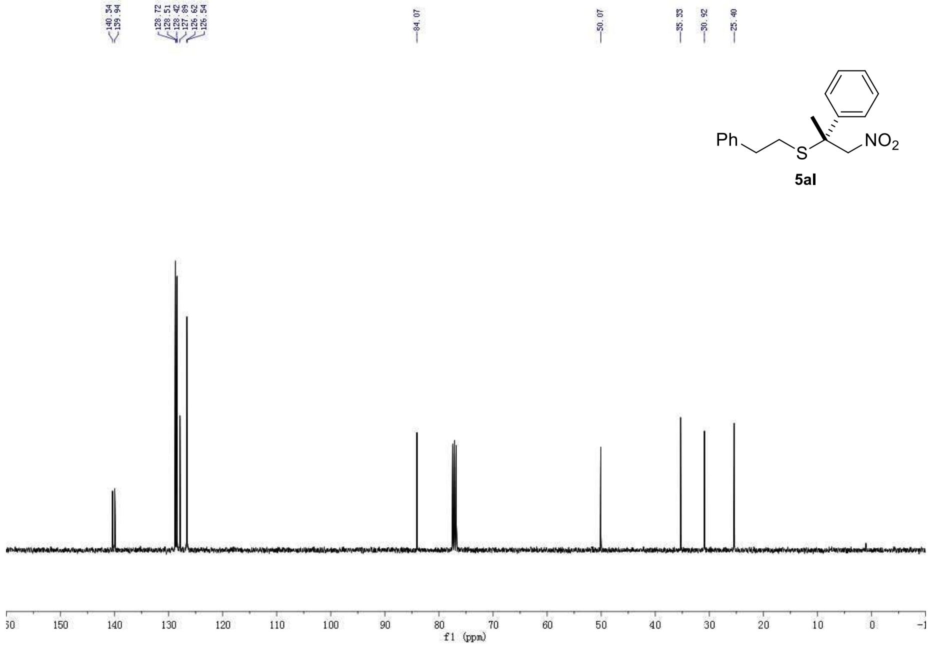

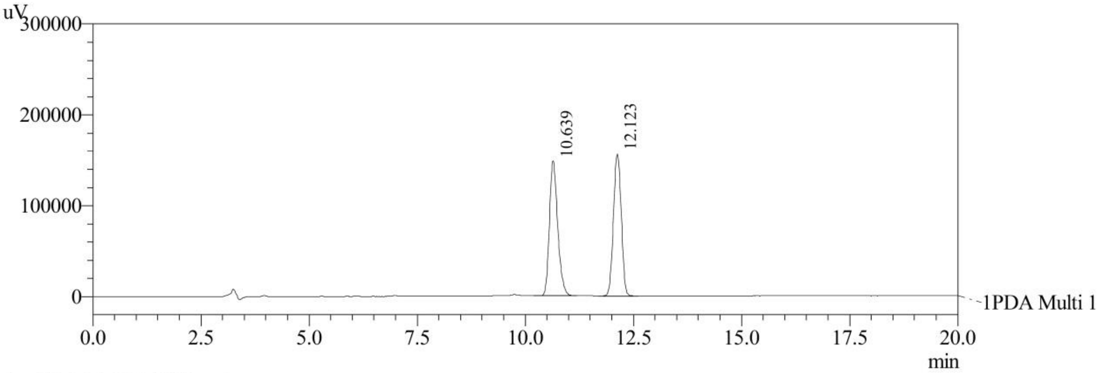

1 PDA Multi 1 / 210nm 4nm

PeakTable

PDA Ch1 210nm 4nm

Peak#	Ret. Time	Area	Height	Area %	Height %
1	10.639	1974618	148571	50.029	48.689
2	12.123	1972341	156572	49.971	51.311
Total		3946959	305143	100.000	100.000

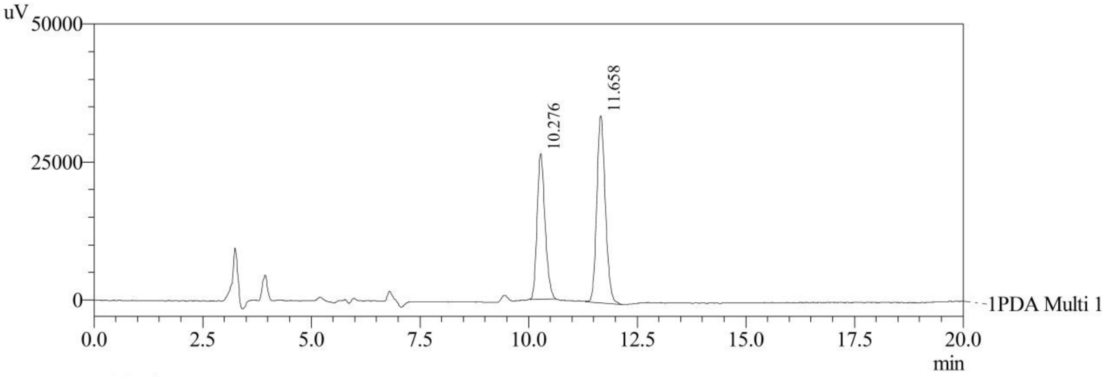

1 PDA Multi 1 / 210nm 4nm

PeakTable

PDA Ch1 210nm 4nm

Peak#	Ret. Time	Area	Height	Area %	Height %
1	10.276	337032	26417	41.854	43.765
2	11.658	468231	33945	58.146	56.235
Total		805263	60362	100.000	100.000

(R)-(1,1-difluoro-3-nitro-2-phenylpropan-2-yl)(phenethyl)sulfane (5am)

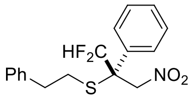

5am

The title compound was prepared according to the general procedure A and purified by flash column chromatography (50:1 hexanes : EtOAc) to afford **5am** (32 mg, 95%) as a colorless oil. Analytical data: IR (KBr, cm^{-1}) 3028, 2925, 1563, 1495, 1449, 1428, 1373, 1141, 1112, 1085, 1020, 967, 748, 696, 672, 556; ^{1}H NMR (400 MHz, $CDCl_3$) δ 7.56 (d, *J* = 8.0 Hz, 2H), 7.49 – 7.36 (m, 3H), 7.34 – 7.21 (m, 3H), 7.14 – 7.06 (m, 2H), 6.94 – 6.56 (m, 1H), 5.14 (dd, *J* = 13.6, 1.5 Hz, 1H), 4.98 (dd, *J* = 13.6, 1.0 Hz, 1H), 2.88 – 2.70 (m, 3H), 2.70 – 2.54 (m, 1H); ^{19}F NMR (376 MHz, $CDCl_3$) δ -118.53 (s), -119.27 (s), -124.10 (s), -124.84 (s); ^{13}C NMR (101 MHz, $CDCl_3$) δ 139.47 (s), 133.09 (d, *J* = 3.0 Hz), 129.16 (s), 128.97 (s), 128.54 (s), 128.48 (s), 127.77 (s), 126.65 (s), 115.96 (t, *J* = 247.0 Hz), 78.72 (t, *J* = 5.0 Hz), 56.85 (t, *J* = 21.0 Hz), 35.23 (s), 31.78 (s). HPLC (AD-H, 5% EtOH in hexanes, 0.8 mL/min, 210 nm): t_{major} = 12.7 min, t_{minor} = 11.3 min, 90% ee; $^{25}[\alpha]_D$ = -27.8 ° (c = 1.0 in $CHCl_3$); HRMS (ESI+) Calcd for $C_{17}H_{17}NO_2F_2NaS^+$ $(M+Na)^+$: 360.0846, Found: 360.0835.

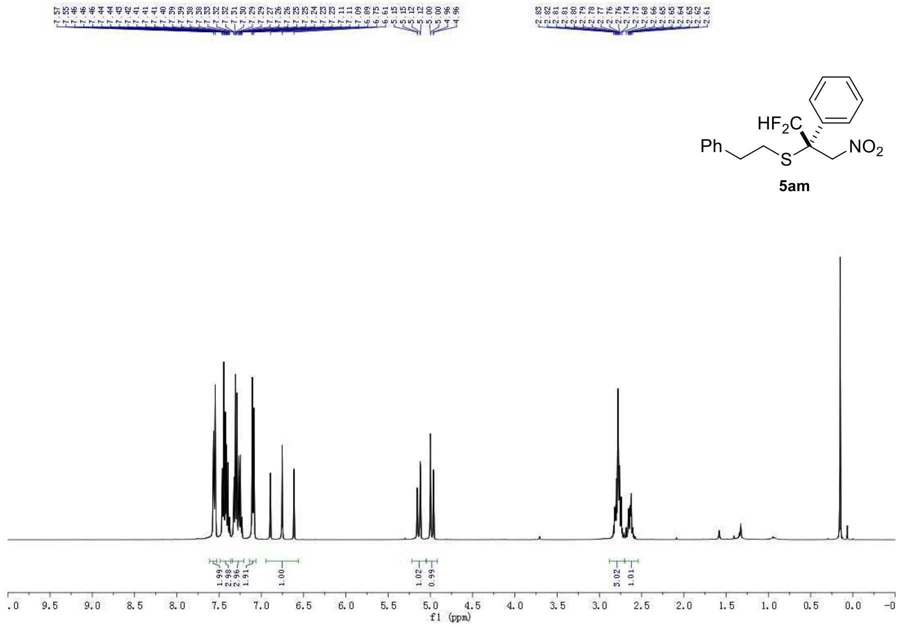

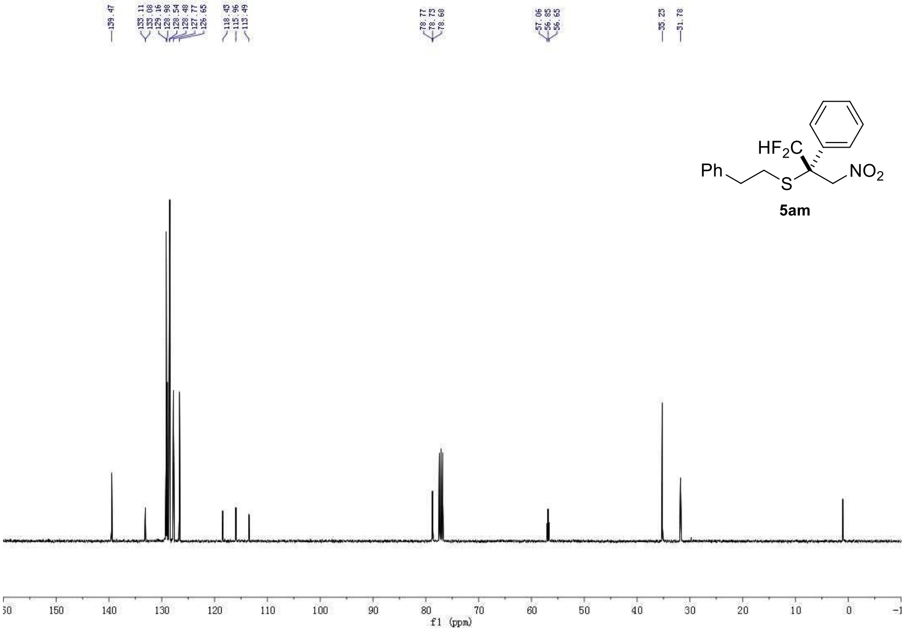

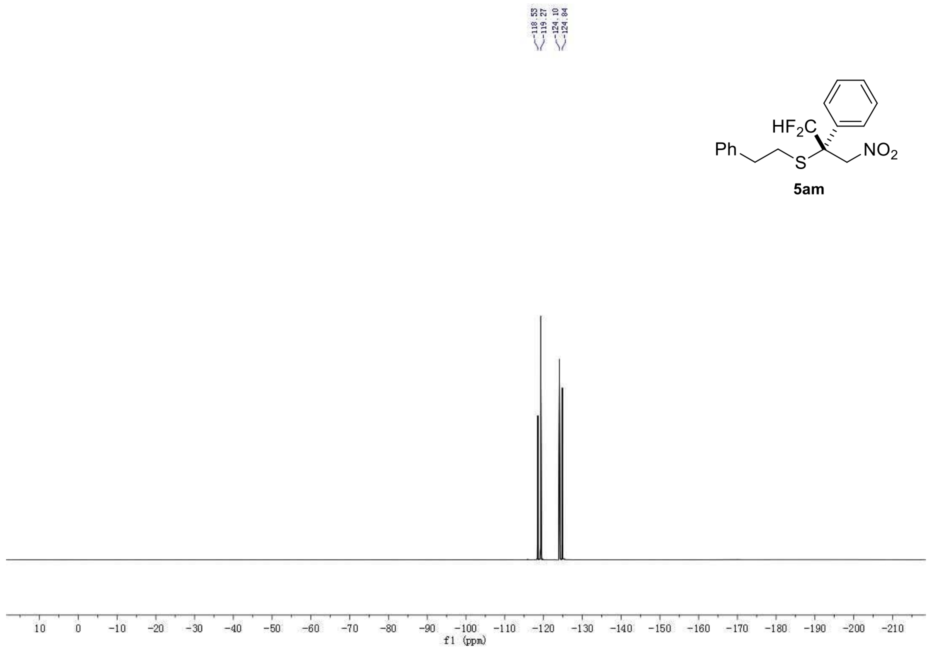

5am

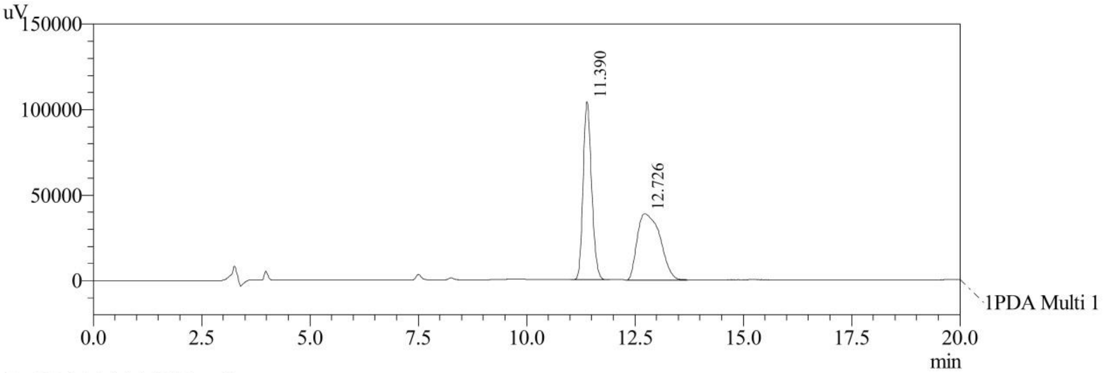

1 PDA Multi 1 / 210nm 4nm

PeakTable

PDA Ch1 210nm 4nm

Peak#	Ret. Time	Area	Height	Area %	Height %
1	11.390	1423648	104210	49.938	72.872
2	12.726	1427201	38795	50.062	27.128
Total		2850849	143004	100.000	100.000

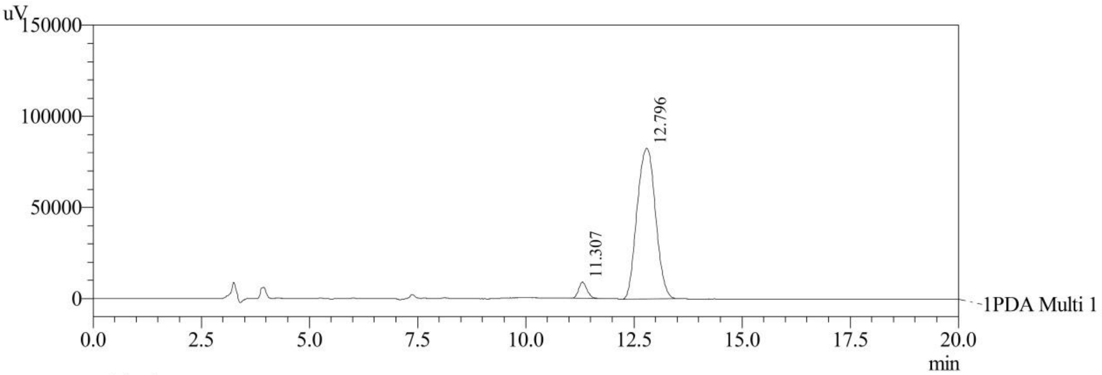

1 PDA Multi 1 / 210nm 4nm

PeakTable

PDA Ch1 210nm 4nm

Peak#	Ret. Time	Area	Height	Area %	Height %
1	11.307	121031	8948	4.670	9.751
2	12.796	2470581	82814	95.330	90.249
Total		2591612	91762	100.000	100.000

(S)-4-(benzylthio)-5,5,5-trifluoro-4-phenylpentan-2-one (6la)

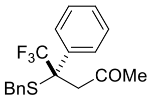

6la

The title compound was prepared according to the general procedure B and purified by flash column chromatography (20:1 hexanes : EtOAc) to afford **6la** (17 mg, 50%) as a colorless oil. Analytical data: IR (KBr, cm^{-1}) 2925, 2850, 1707, 1496, 1454, 1362, 1235, 1164, 1066, 752, 707; ^{1}H NMR (400 MHz, $CDCl_3$) δ 7.71 (d, *J* = 8.2 Hz, 2H), 7.50 – 7.42 (m, 2H), 7.40 (dt, *J* = 9.5, 4.2 Hz, 1H), 7.33 – 7.21 (m, 5H), 3.87 (d, *J* = 11.2 Hz, 1H), 3.56 (d, *J* = 11.2 Hz, 1H), 3.36 (q, *J* = 15.6 Hz, 2H), 1.95 (s, 3H); ^{19}F NMR (376 MHz, $CDCl_3$) δ -67.82 (s, 3F); ^{13}C NMR (100 MHz, $CDCl_3$) δ 202.36 (s), 135.77 (s), 134.63 (s), 129.31 (s), 129.03 (s), 128.66 (s),128.63 (s), 128.51 (s), 128.04 (d, J = 1.4 Hz), 127.05 (q, *J* = 282.0 Hz), 127.51 (s), 58.04 (q, *J* = 26.0 Hz), 48.20 (s), 35.52 (d, J = 2.1 Hz), 31.23 (s). HPLC (AD-H, 1% EtOH in hexanes, 1.0 mL/min, 210 nm): t_{major} = 11.8 min, t_{minor} = 9.5 min, 90% ee; $^{25}[\alpha]_D$ = 10.5 ° (c = 1.0 in $CHCl_3$); HRMS (ESI+) Calcd for $C_{18}H_{17}OF_3NaS^+$ $(M+Na)^+$: 361.0850, Found: 361.0847.

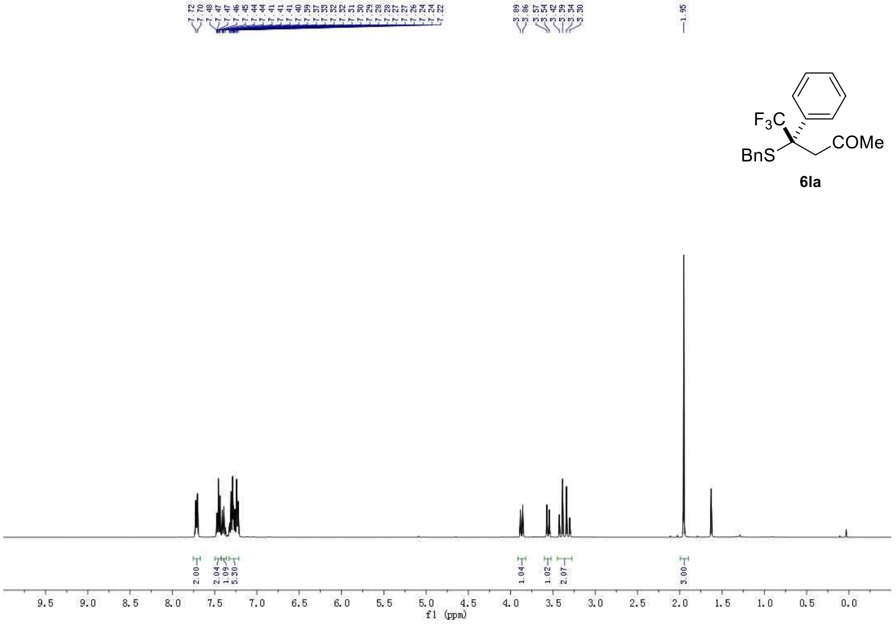

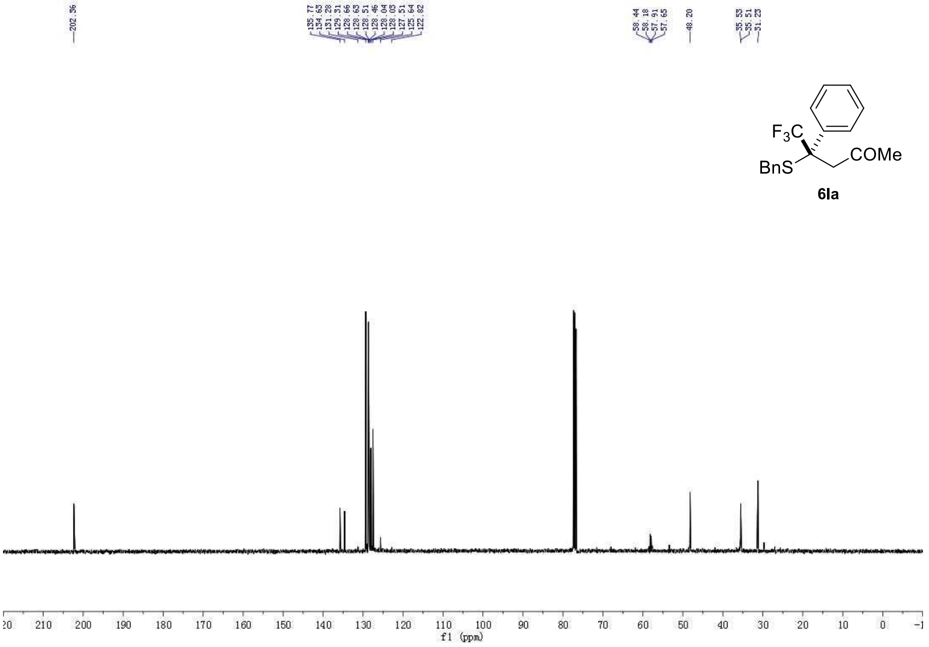

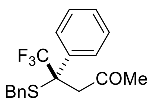

6la

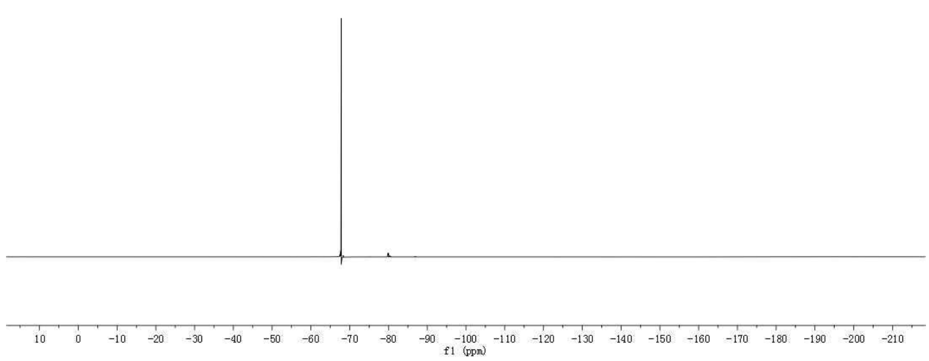

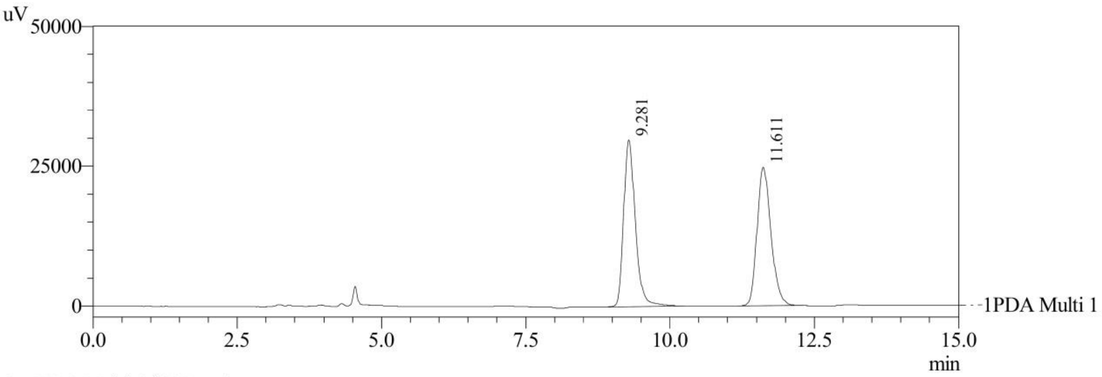

1 PDA Multi 1 / 225nm 4nm

PeakTable

PDA Ch1 225nm 4nm

Peak#	Ret. Time	Area	Height	Area %	Height %
1	9.281	423583	29808	50.354	54.636
2	11.611	417624	24750	49.646	45.364
Total		841207	54558	100.000	100.000

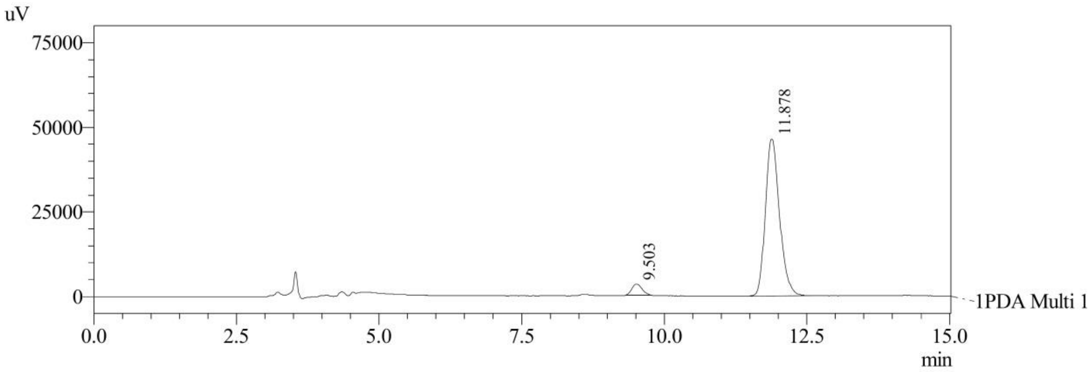

1 PDA Multi 1 / 210nm 4nm

PeakTable

PDA Ch1 210nm 4nm

Peak#	Ret. Time	Area	Height	Area %	Height %
1	9.503	41623	3265	4.941	6.566
2	11.878	800828	46455	95.059	93.434
Total		842451	49719	100.000	100.000

(S)-4-(benzylthio)-4-(4-chlorophenyl)-5,5,5-trifluoropentan-2-one (6lb)

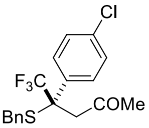

6lb

The title compound was prepared according to the general procedure B and purified by flash column chromatography (20:1 hexanes : EtOAc) to afford **6lb** (31 mg, 83%) as a colorless oil. Analytical data: IR (KBr, cm^{-1}) 2916, 2906, 1734, 1496, 1234, 1165, 1097, 1012, 706; ^{1}H NMR (400 MHz, $CDCl_3$) δ 7.62 (d, *J* = 8.3 Hz, 2H), 7.44 – 7.38 (m, 2H), 7.34 – 7.20 (m, 5H), 3.86 (d, *J* = 11.4 Hz, 1H), 3.58 (d, *J* = 11.4 Hz, 1H), 3.34 (dd, J = 41.4, 16.0 Hz, 2H), 2.01 (s, 3H); ^{19}F NMR (376 MHz, $CDCl_3$) δ -67.98 (s, 3F); ^{13}C NMR (100 MHz, $CDCl_3$) δ 201.74 (s), 135.54 (s), 134.52 (s), 133.25 (s), 129.50 (s), 129.27 (s), 128.76 (s), 128.68 (s), 127.61 (s), 126.83 (q, *J* = 282.0 Hz), 57.60 (q, *J* = 27 Hz), 47.65 (s), 35.58 (d, *J* = 2.1 Hz), 31.35 (s). HPLC (AD-H, 2.5% EtOH in hexanes, 1.0 mL/min, 210 nm): t_{major} = 12.5 min, t_{minor} = 9.8 min, 93% ee; $^{25}[\alpha]_D$ = 27.3 ° (c = 1.0 in $CHCl_3$); HRMS (ESI+) Calcd for $C_{18}H_{16}OF_3NaSCl^+$ $(M+Na)^+$: 395.0460, Found: 395.0453.

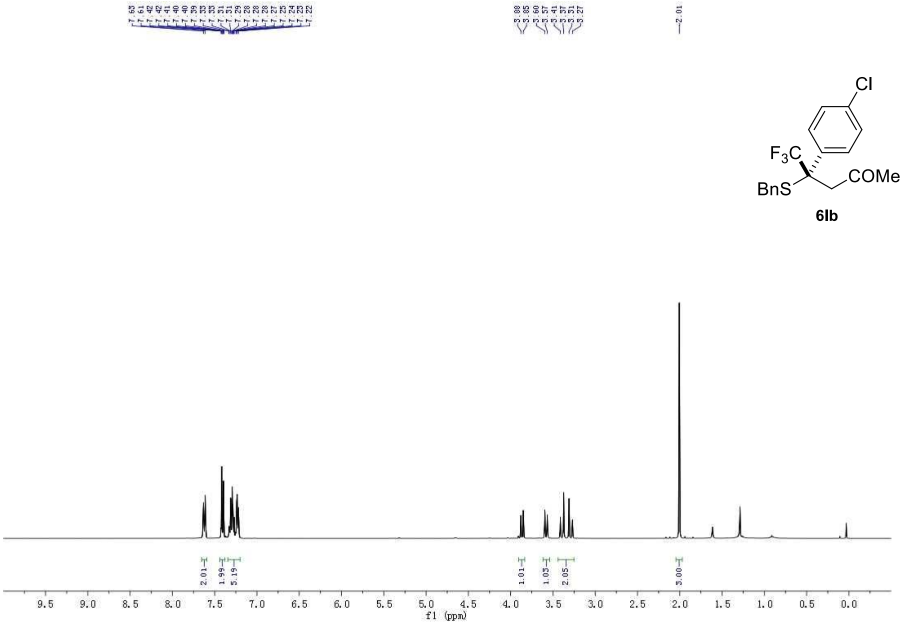

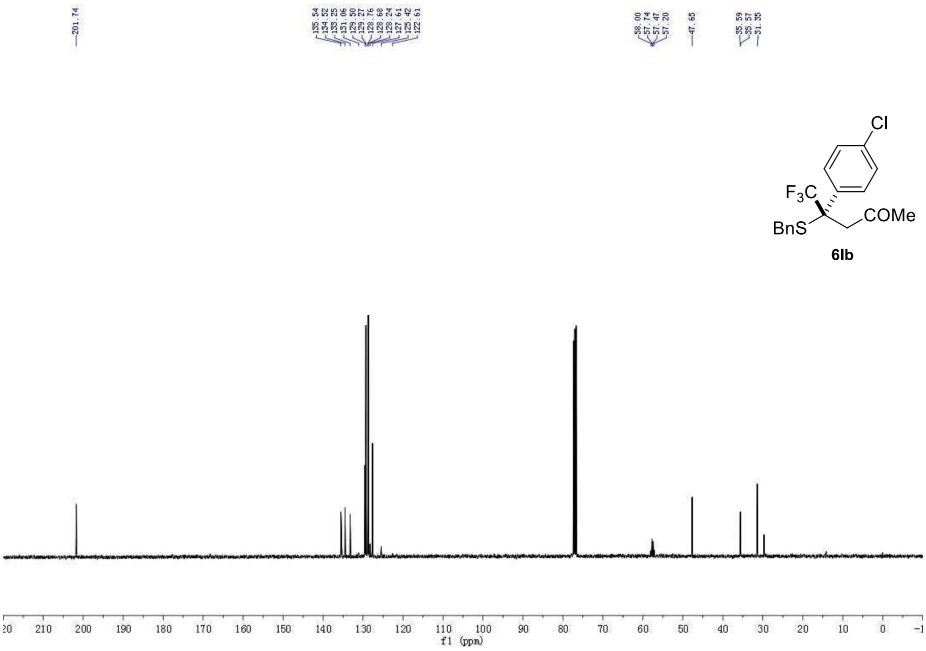

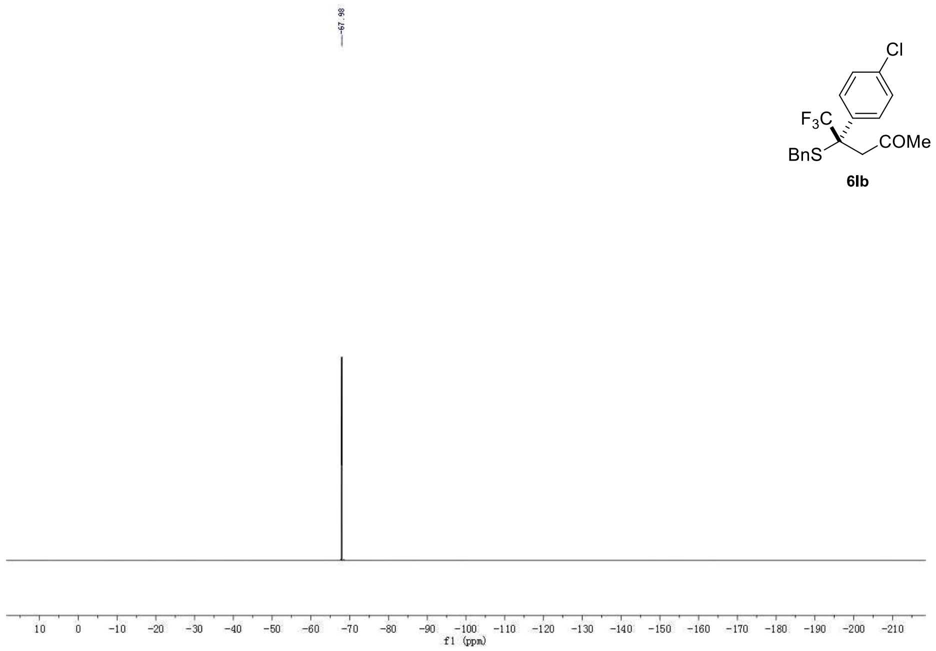

6lb

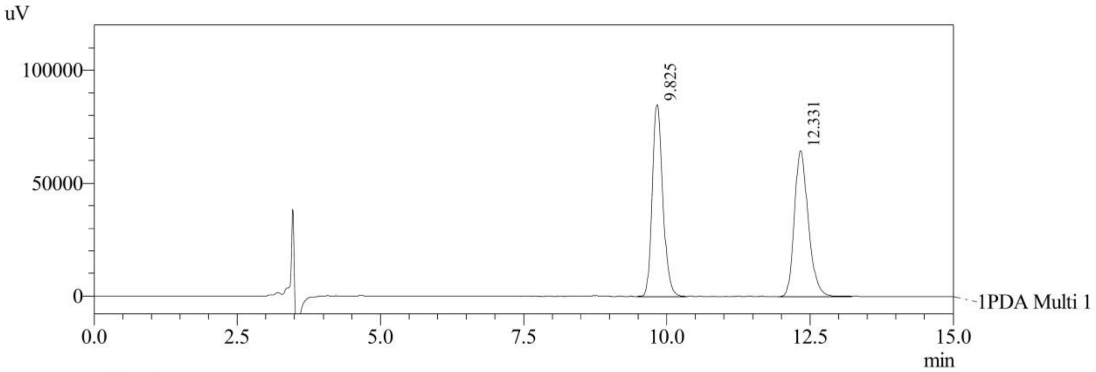

1 PDA Multi 1 / 210nm 4nm

PeakTable

PDA Ch1 210nm 4nm

Peak#	Ret. Time	Area	Height	Area %	Height %
1	9.825	1094603	85055	50.018	56.820
2	12.331	1093805	64636	49.982	43.180
Total		2188408	149691	100.000	100.000

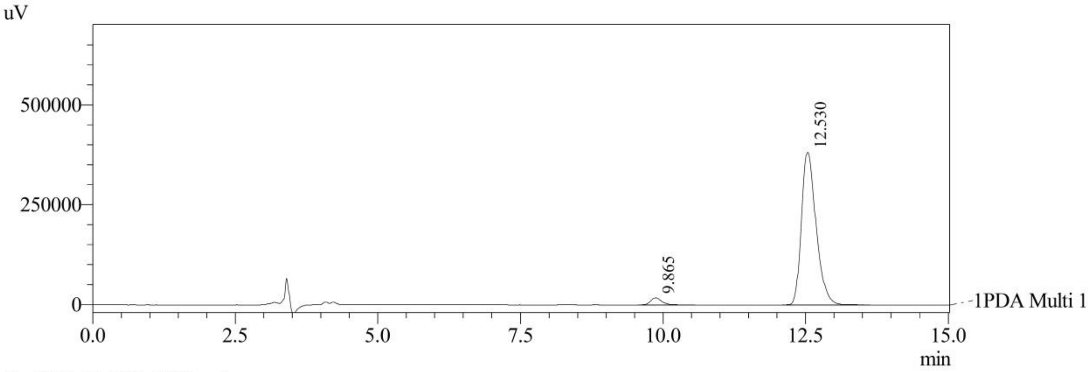

1 PDA Multi 1 / 210nm 4nm

PeakTable

PDA Ch1 210nm 4nm

Peak#	Ret. Time	Area	Height	Area %	Height %
1	9.865	239598	17438	3.398	4.368
2	12.530	6811533	381783	96.602	95.632
Total		7051131	399222	100.000	100.000

(S)-4-(benzylthio)-5,5,5-trifluoro-4-(4-fluorophenyl)pentan-2-one (6lc)

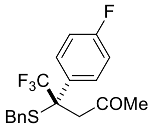

6lc

The title compound was prepared according to the general procedure B and purified by flash column chromatography (20:1 hexanes : EtOAc) to afford **6lc** (28mg, 80%) as a colorless oil. Analytical data: IR (KBr, cm^{-1}) 2925, 2858, 1710, 1515, 1454, 1360, 1236, 1164, 1043, 812, 776, 708; ^{1}H NMR (400 MHz, $CDCl_3$) δ 7.68 (dd, *J* = 8.3, 5.1 Hz, 2H), 7.35 – 7.22 (m, 5H), 7.18 – 7.08 (m, 2H), 3.97 – 3.82 (m, 1H), 3.60 (d, *J* = 11.4 Hz, 1H), 3.41 (d, *J* = 15.9 Hz, 1H), 3.30 (d, *J* = 15.9 Hz, 1H), 2.00 (s, 3H); ^{19}F NMR (376 MHz, $CDCl_3$) δ -68.23 (s, 3F), δ -112.89 (s, 1F); ^{13}C NMR (100 MHz, $CDCl_3$) δ 201.96 (s), 162.31 (d, *J* = 148.0 Hz), 135.61 (s), 130.42 (d, *J* = 3.6 Hz), 130.08 (d, *J* = 6.7 Hz), 129.28 (s), 128.68 (s), 127.60 (s), 126.90 (q, *J* = 282.0 Hz), 115.57 (d, *J* = 21.0 Hz) , 57.48 (q, J = 27.0 Hz), 47.71 (s), 35.57 (s), 31.34 (s). HPLC (AD-H, 5% EtOH in hexanes, 1.0 mL/min, 210 nm): t_{major} = 9.3 min, t_{minor} = 6.5 min, 86% ee; $^{25}[\alpha]_D$ = 18.8 ^{o} (c = 1.0 in $CHCl_3$); HRMS (ESI+) Calcd for $C_{18}H_{16}OF_4NaS^+$ $(M+Na)^+$: 379.0756, Found: 379.0751.

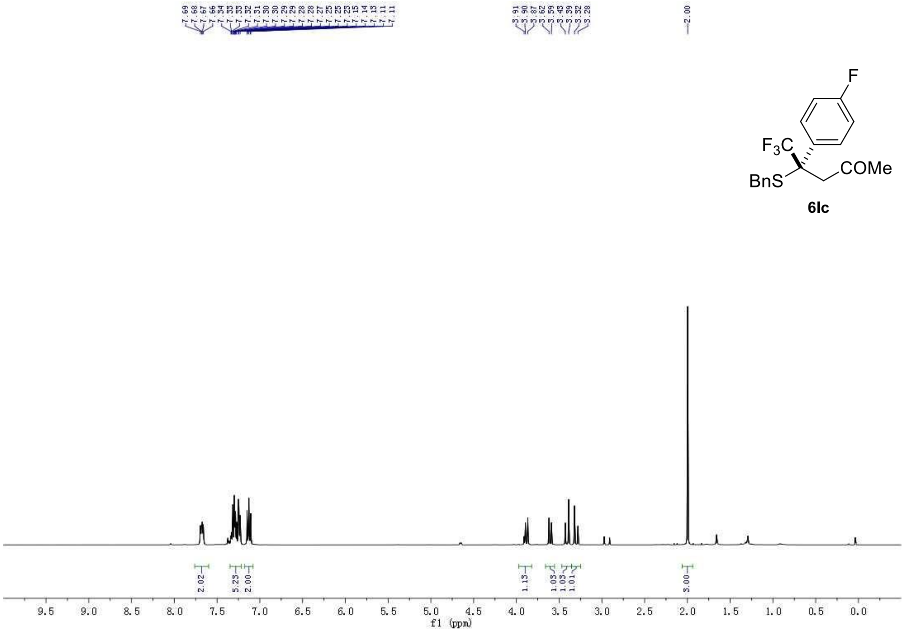

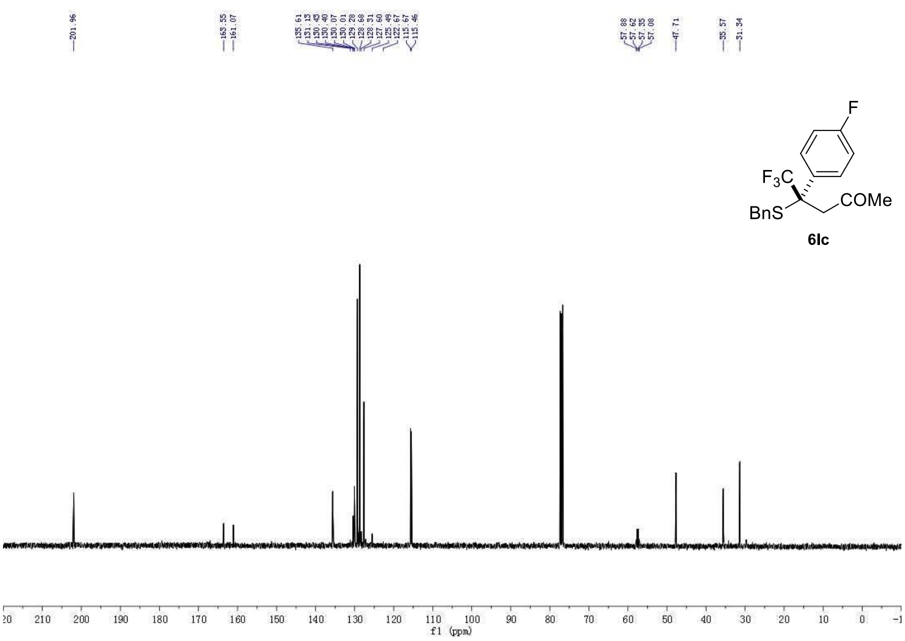

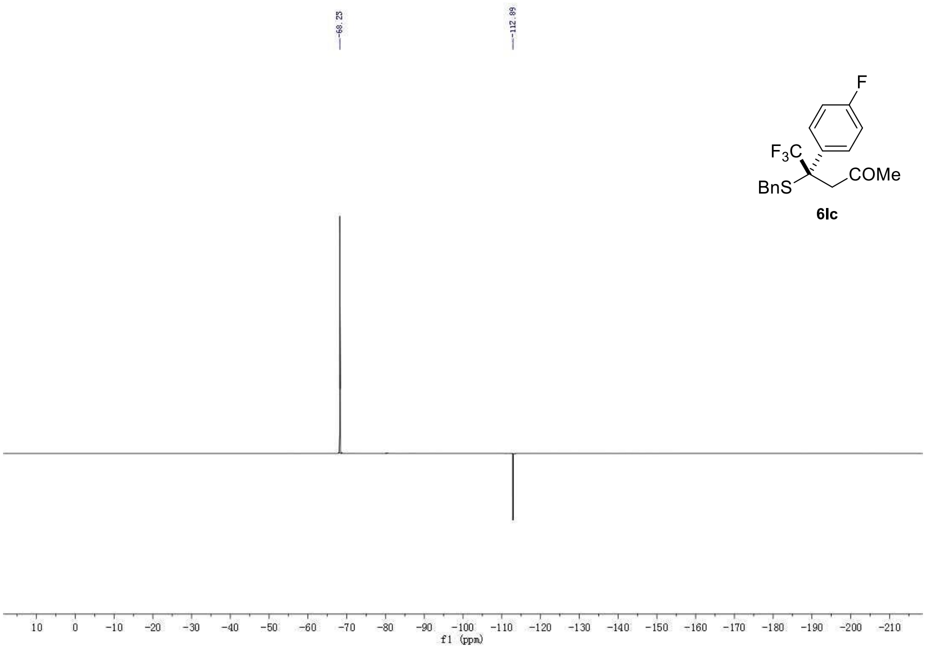

6lc

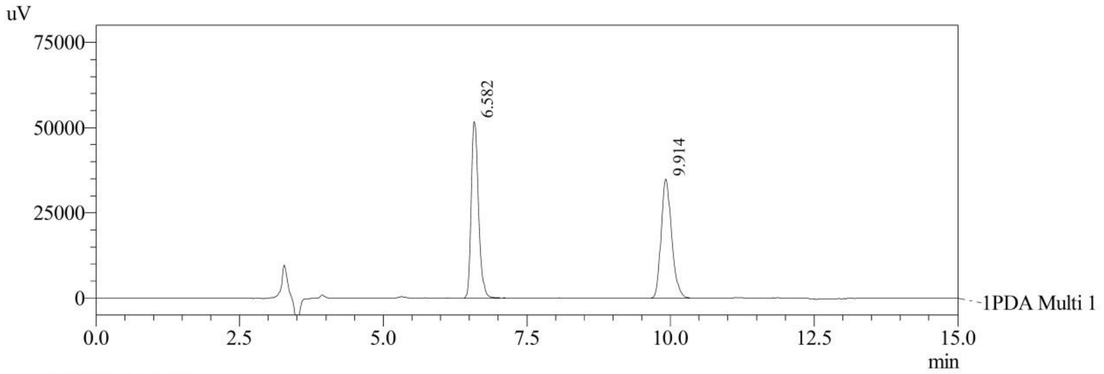

1 PDA Multi 1 / 210nm 4nm

PeakTable

PDA Ch1 210nm 4nm

Peak#	Ret. Time	Area	Height	Area %	Height %
1	6.582	457029	51782	50.341	59.621
2	9.914	450843	35070	49.659	40.379
Total		907871	86852	100.000	100.000

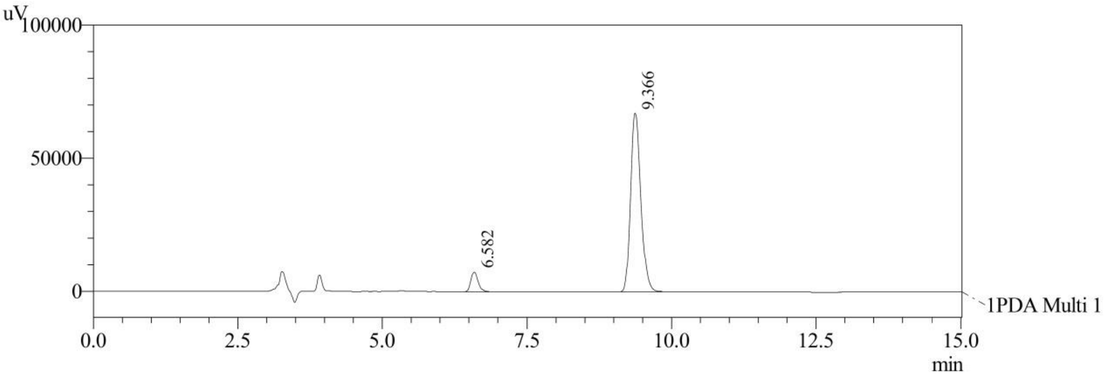

1 PDA Multi 1 / 210nm 4nm

PeakTable

PDA Ch1 210nm 4nm

Peak#	Ret. Time	Area	Height	Area %	Height %
1	6.582	64606	7454	7.248	9.986
2	9.366	826735	67191	92.752	90.014
Total		891341	74645	100.000	100.000

(S)-4-(benzylthio)-5,5,5-trifluoro-4-(p-tolyl)pentan-2-one (6ld)

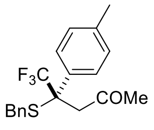

6ld

The title compound was prepared according to the general procedure B and purified by flash column chromatography (20:1 hexanes : EtOAc) to afford **6ld** (20 mg, 56%) as a colorless oil. Analytical data: IR (KBr, cm^{-1}) 2936, 2840, 1707, 1611, 1515, 1455, 1360, 1258, 1164, 1029, 829, 709; ^{1}H NMR (400 MHz, $CDCl_3$) δ 7.60 (d, J = 8.0 Hz, 2H), 7.39 – 7.18 (m, 7H), 3.88 (d, J = 11.2 Hz, 1H), 3.59 (d, J = 11.2 Hz, 1H), 3.35 (dd, J = 34.8, 15.5 Hz, 2H), 2.40 (s, 3H), 1.96 (s, 3H); ^{19}F NMR (376 MHz, $CDCl_3$) δ -68.07 (s, 3F); ^{13}C NMR (100 MHz, $CDCl_3$) δ 202.58 (s), 138.44 (s), 135.87 (s), 131.48 (s), 129.36 (s), 129.34 (s), 128.61 (s), 127.94 (s), 127.48 (s), 127.10 (q, J = 282.0 Hz), 57.83 (q, J = 26.0 Hz), 48.18 (s), 35.50 (d, J = 2.0 Hz), 31.28 (s), 21.00 (s). HPLC (AD-H, 2.5% EtOH in hexanes, 1.0 mL/min, 210 nm): t_{major} = 8.2 min, t_{minor} = 7.0 min, 90% ee; $^{25}[\alpha]_D$ = 18.4 ° (c = 1.0 in $CHCl_3$); HRMS (ESI+) Calcd for $C_{19}H_{19}OF_3NaS^+$ $(M+Na)^+$: 375.1006, Found: 375.0998.

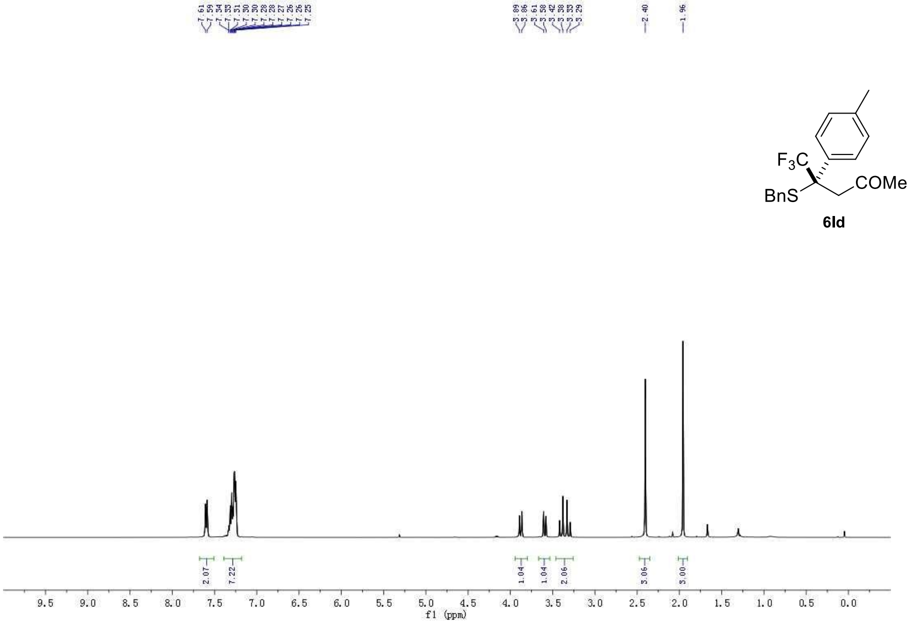

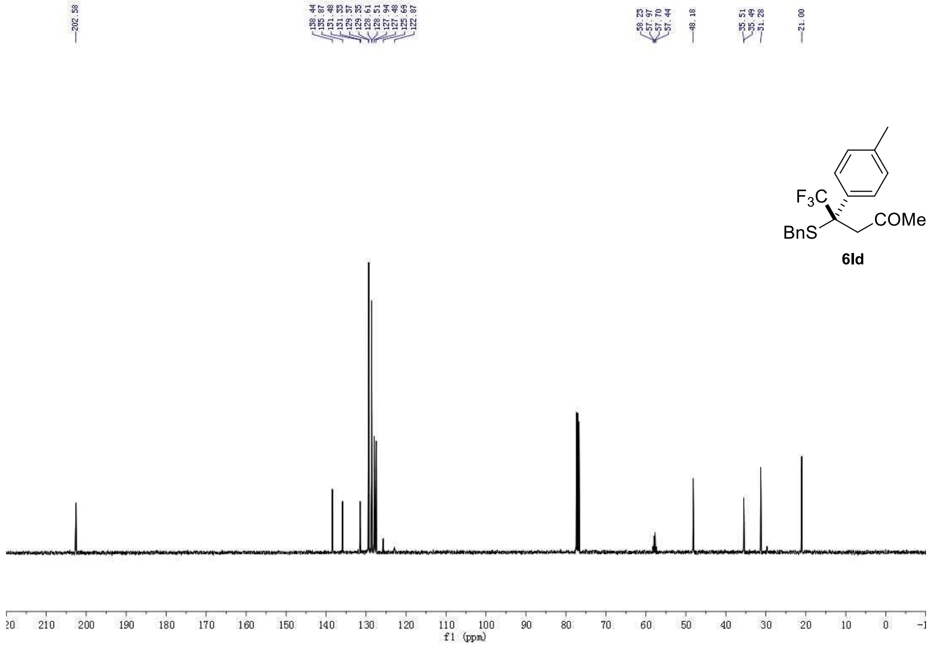

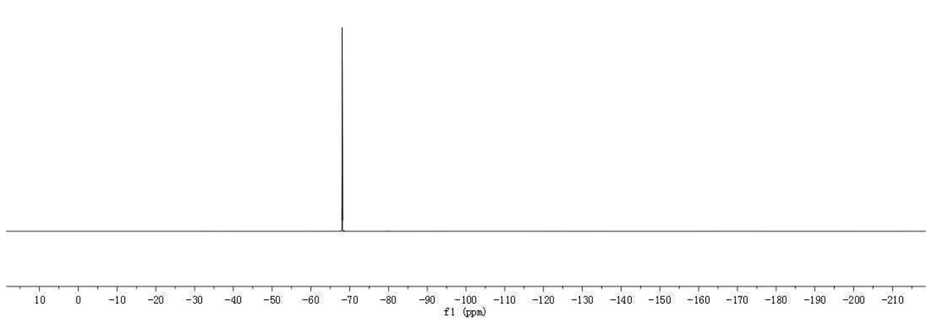

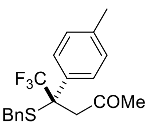

6ld

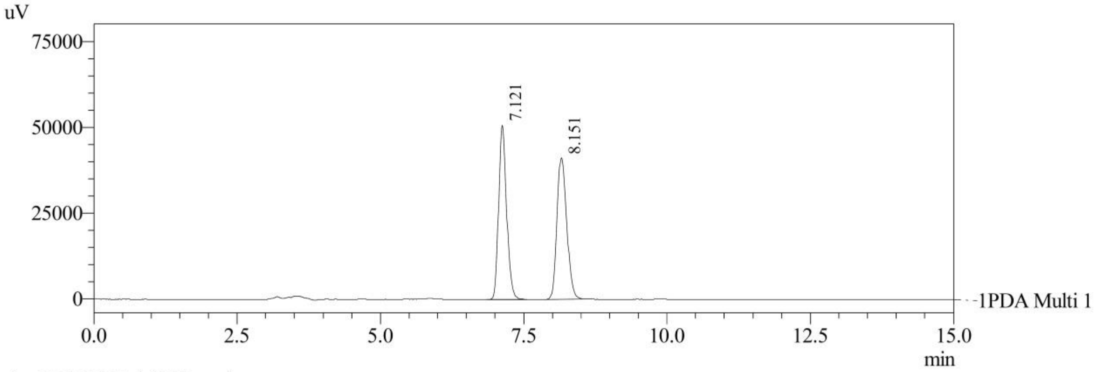

1 PDA Multi 1 / 220nm 4nm

PeakTable

PDA Ch1 220nm 4nm

Peak#	Ret. Time	Area	Height	Area %	Height %
1	7.121	491843	50694	49.993	55.156
2	8.151	491979	41216	50.007	44.844
Total		983822	91910	100.000	100.000

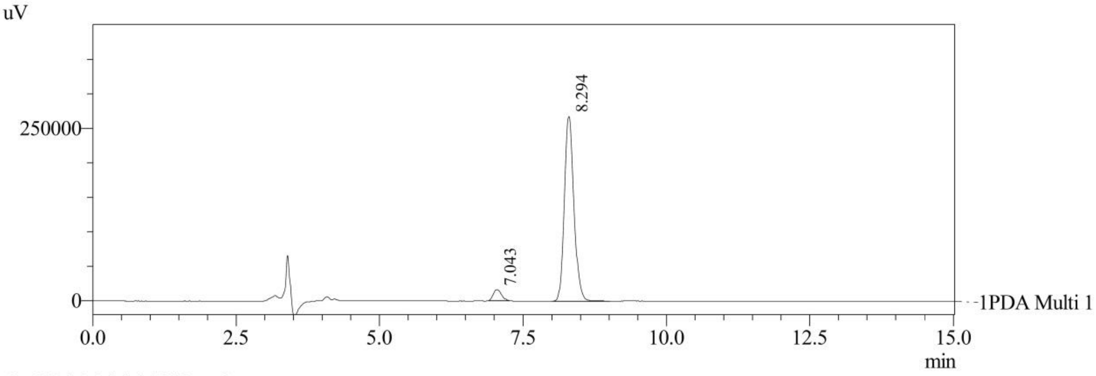

1 PDA Multi 1 / 210nm 4nm

PeakTable

PDA Ch1 210nm 4nm

Peak#	Ret. Time	Area	Height	Area %	Height %
1	7.043	160850	16188	4.950	5.709
2	8.294	3088499	267375	95.050	94.291
Total		3249349	283563	100.000	100.000

(S)-4-(benzylthio)-5,5,5-trifluoro-4-(4-methoxyphenyl)pentan-2-one (6le)

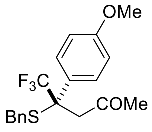

6le

The title compound was prepared according to the general procedure B and purified by flash column chromatography (20:1 hexanes : EtOAc) to afford **6le** (33 mg, 90%) as a colorless oil. Analytical data: IR (KBr, cm^{-1}) 2923, 2850, 1707, 1496, 1454, 1361, 1235, 1160, 1039, 815, 748, 715; ^{1}H NMR (400 MHz, $CDCl_3$) δ 7.63 (d, J = 8.6 Hz, 2H), 7.28 (ddd, J = 14.4, 13.4, 7.4 Hz, 5H), 6.96 (d, J = 9.0 Hz, 2H), 4.00 – 3.78 (m, 4H), 3.73 – 3.52 (m, 1H), 3.34 (dd, J = 36.2, 15.4 Hz, 2H), 1.96 (s, 3H); ^{13}C NMR (100 MHz, $CDCl_3$) δ 202.66 (s), 159.38 (s), 135.87 (s), 129.39 (s), 129.33 (s), 128.63 (s), 127.49 (s), 127.08 (q, J = 282.0 Hz), 126.22 (s), 113.93 (s), 57.58 (q, J = 27.0 Hz), 55.26 (s), 48.10 (s), 35.49 (s), 31.32 (s). HPLC (AD-H, 5% EtOH in hexanes, 1.0 mL/min, 210 nm): t_{major} = 9.3 min, t_{minor} = 8.5 min, 93% ee; $^{25}[\alpha]_D$ = 47.8 ^{o} (c = 1.0 in $CHCl_3$); HRMS (ESI+) Calcd for $C_{19}H_{19}O_2F_3NaS^+$ $(M+Na)^+$: 391.0956, Found: 391.0949.

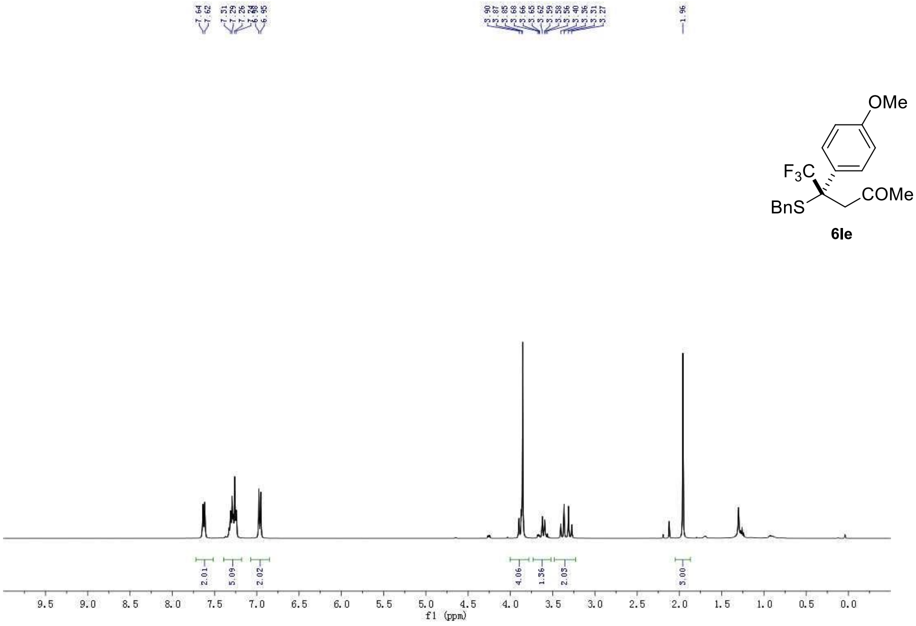

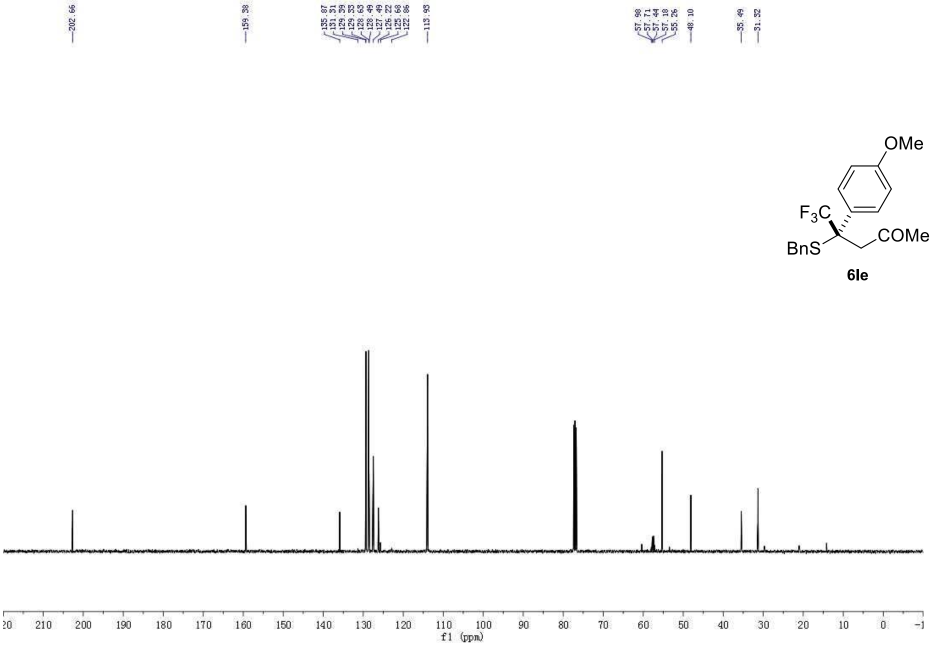

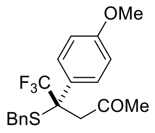

6le

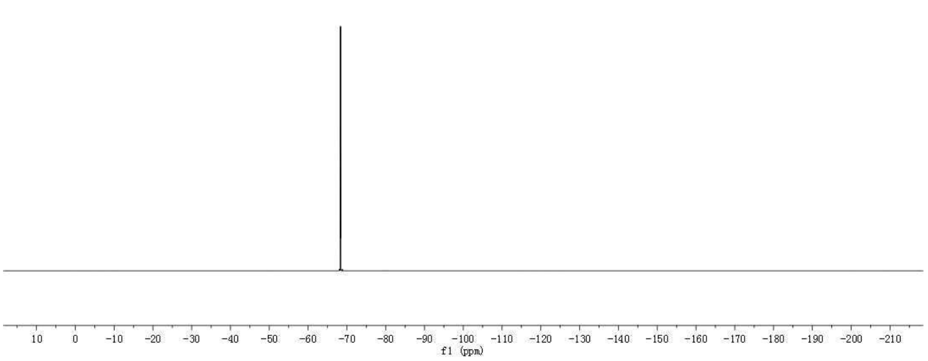

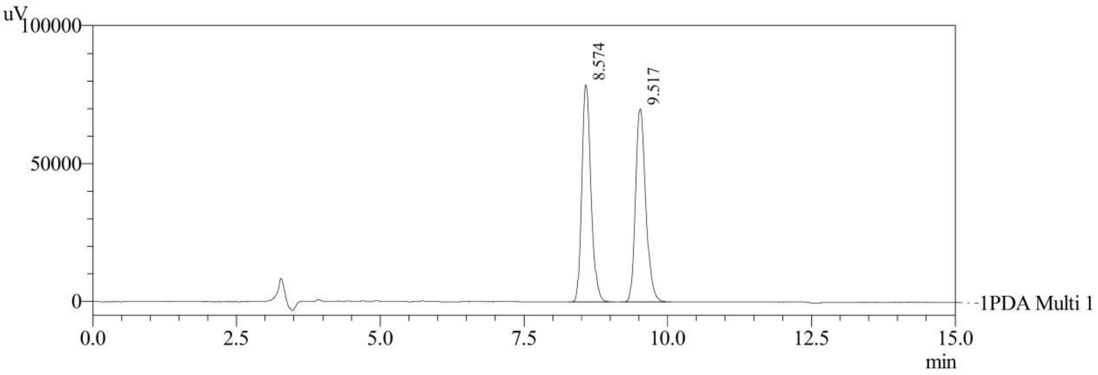

1 PDA Multi 1 / 210nm 4nm

PeakTable

PDA Ch1 210nm 4nm

Peak#	Ret. Time	Area	Height	Area %	Height %
1	8.574	869718	78867	50.063	52.954
2	9.517	867535	70069	49.937	47.046
Total		1737253	148935	100.000	100.000

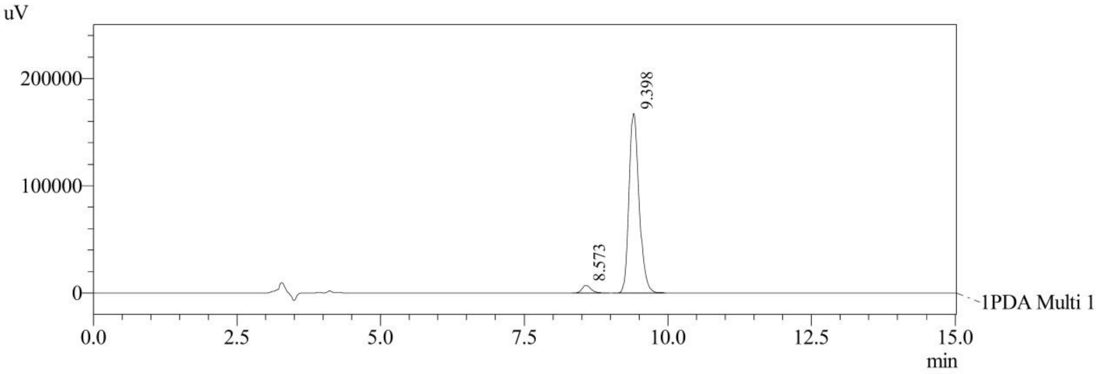

1 PDA Multi 1 / 210nm 4nm

PeakTable

PDA Ch1 210nm 4nm

Peak#	Ret. Time	Area	Height	Area %	Height %
1	8.573	77897	7051	3.658	4.047
2	9.398	2051438	167184	96.342	95.953
Total		2129335	174234	100.000	100.000

(S)-5,5,5-trifluoro-4-(4-methoxyphenyl)-4-(phenethylthio)pentan-2-one (6ae)

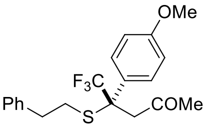

6ae

The title compound was prepared according to the general procedure B and purified by flash column chromatography (20:1 hexanes : EtOAc) to afford **6ae** (32 mg, 84%) as a colorless oil. Analytical data: IR (KBr, cm^{-1}) 2923, 2850, 1707, 1495, 1454, 1369, 1218, 1167, 1039, 815, 749, 696; ^{1}H NMR (400 MHz, $CDCl_3$) δ 7.56 (d, J = 8.6 Hz, 2H), 7.27 (dt, J = 27.2, 7.2 Hz, 3H), 7.14 (d, J = 7.1 Hz, 2H), 6.93 (d, J = 8.9 Hz, 2H), 3.85 (s, 3H), 3.28 (dd, J = 40.8, 15.3 Hz, 2H), 2.91 – 2.73 (m, 3H), 2.73 – 2.62 (m, 1H), 1.95 (s, 3H); ^{19}F NMR (376 MHz, $CDCl_3$) δ -68.38 (s, 3F); ^{13}C NMR (100 MHz, $CDCl_3$) δ 202.74 (s), 159.28 (s), 139.79 (s), 129.42 (s), 129.41 (s), 128.46 (s), 127.03 (q, J = 282.0 Hz), 126.48 (s), 126.32 (s), 113.86 (s), 57.86 – 56.89 (m), 57.04 (s), 57.04 (s), 56.77 (s), 55.25 (s), 48.42 (s), 34.98 (s), 31.93 (s), 31.37 (s). HPLC (AD-H, 5% EtOH in hexanes, 1.0 mL/min, 210 nm): t_{major} = 6.9 min, t_{minor} = 8.9 min, 87% ee; $^{25}[\alpha]_D$ = 4.2 ° (c = 1.0 in $CHCl_3$); HRMS (ESI+) Calcd for $C_{20}H_{21}O_2F_3NaS^+$ $(M+Na)^+$: 405.1112, Found: 405.1107.

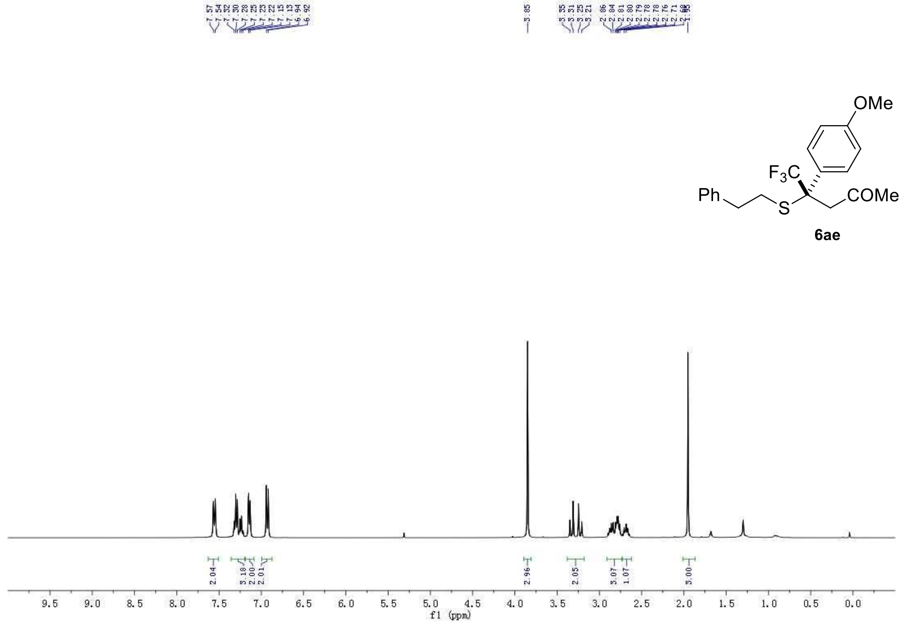

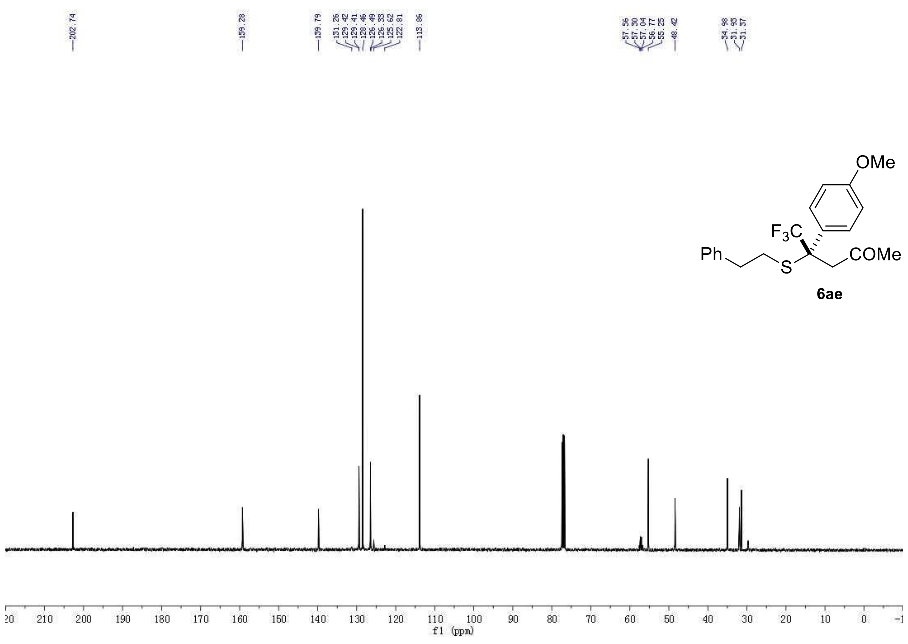

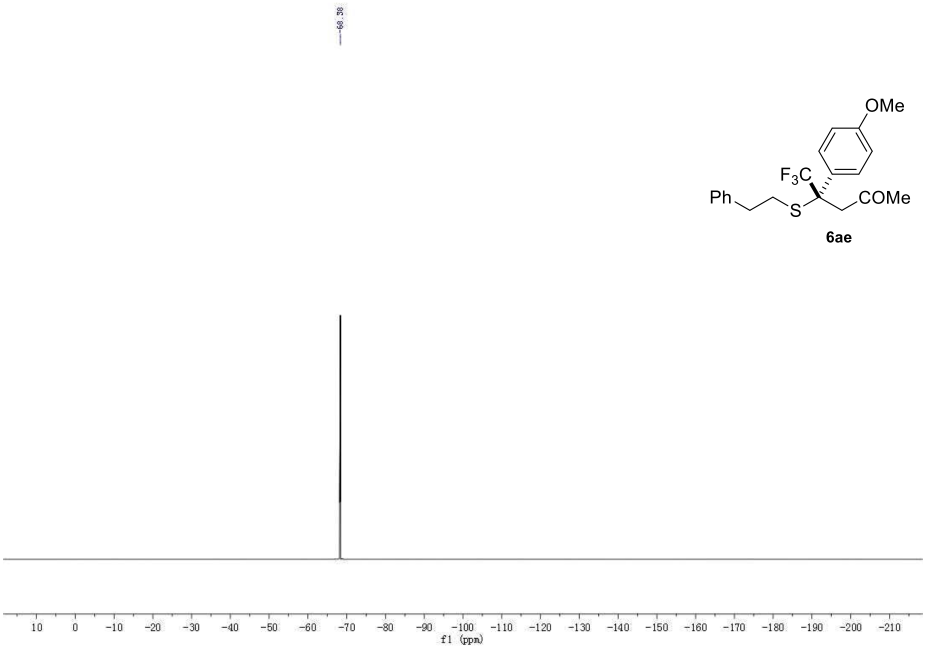

6ae

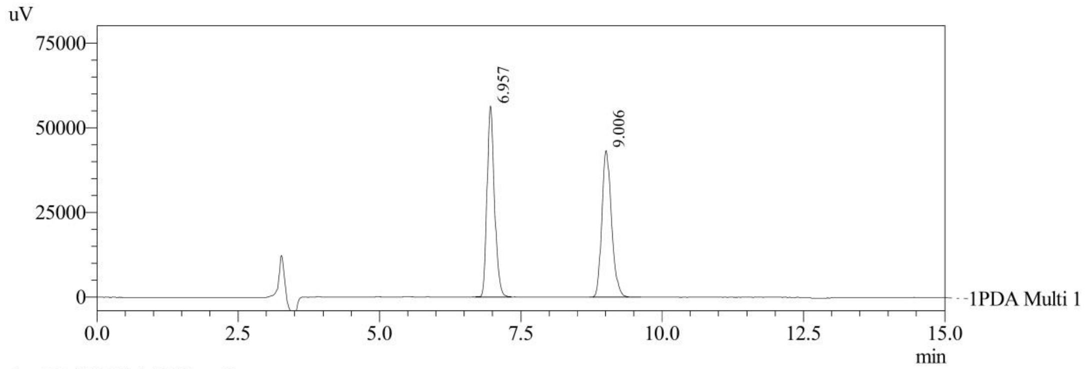

1 PDA Multi 1 / 210nm 4nm

PeakTable

PDA Ch1 210nm 4nm

Peak#	Ret. Time	Area	Height	Area %	Height %
1	6.957	516504	56317	49.915	56.584
2	9.006	518262	43212	50.085	43.416
Total		1034766	99529	100.000	100.000

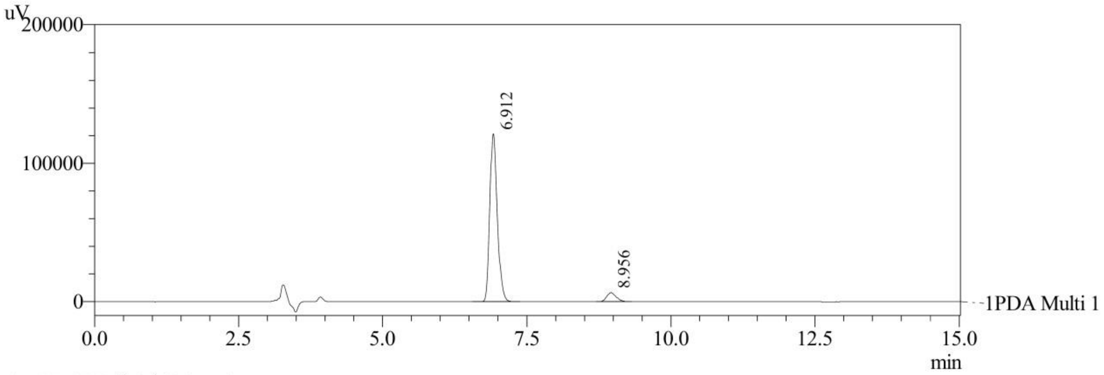

1 PDA Multi 1 / 210nm 4nm

PeakTable

PDA Ch1 210nm 4nm

Peak#	Ret. Time	Area	Height	Area %	Height %
1	6.912	1097341	120993	93.521	94.862
2	8.956	76017	6554	6.479	5.138
Total		1173358	127547	100.000	100.000

(S)-5,5,5-trifluoro-4-((furan-2-ylmethyl)thio)-4-(4-methoxyphenyl)pentan-2-one (6ke)

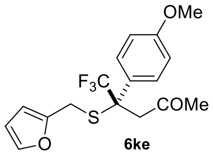

6ke

The title compound was prepared according to the general procedure B and purified by flash column chromatography (20:1 hexanes : EtOAc) to afford **6ke** (35 mg, 99%) as a colorless oil. Analytical data: IR (KBr, cm^{-1}) 2960, 2934, 1710, 1611, 1510, 1260, 1164, 1029, 935, 828, 747; ^{1}H NMR (400 MHz, $CDCl_3$) δ 7.59 (d, *J* = 8.4 Hz, 2H), 7.34 (dd, *J* = 1.8, 0.8 Hz, 1H), 7.00 – 6.90 (m, 2H), 6.29 (dd, *J* = 3.2, 1.9 Hz, 1H), 6.14 (dd, *J* = 3.2, 0.5 Hz, 1H), 3.89 (d, *J* = 13.5 Hz, 1H), 3.84 (s, 3H), 3.64 (d, *J* = 13.5 Hz, 1H), 3.31 (q, *J* = 15.5 Hz, 2H), 1.98 (s, 3H); ^{19}F NMR (376 MHz, $CDCl_3$) δ -68.31 (s, 3F); ^{13}C NMR (100 MHz, $CDCl_3$) δ 202.47 (s), 159.39 (s), 149.47 (s), 142.39 (s), 129.44 (d, *J* = 1.3 Hz), 126.98 (q, *J* = 281.0 Hz), 125.83 (s), 113.96 (s), 110.61 (s), 108.40 (s), 57.62 (q, *J* = 27.0 Hz), 55.24 (s), 48.16 (s), 31.32 (s), 27.80 (d, J = 2.2 Hz). HPLC (AD-H, 2.5% EtOH in hexanes, 1.0 mL/min, 210 nm): t_{major} = 14.2 min, t_{minor} = 15.4 min, 84% ee; $^{25}[\alpha]_D$ = 27.6 ° (c = 1.0 in $CHCl_3$); HRMS (ESI+) Calcd for $C_{17}H_{17}O_3F_3NaS^+$ $(M+Na)^+$: 381.0748, Found: 381.0743.

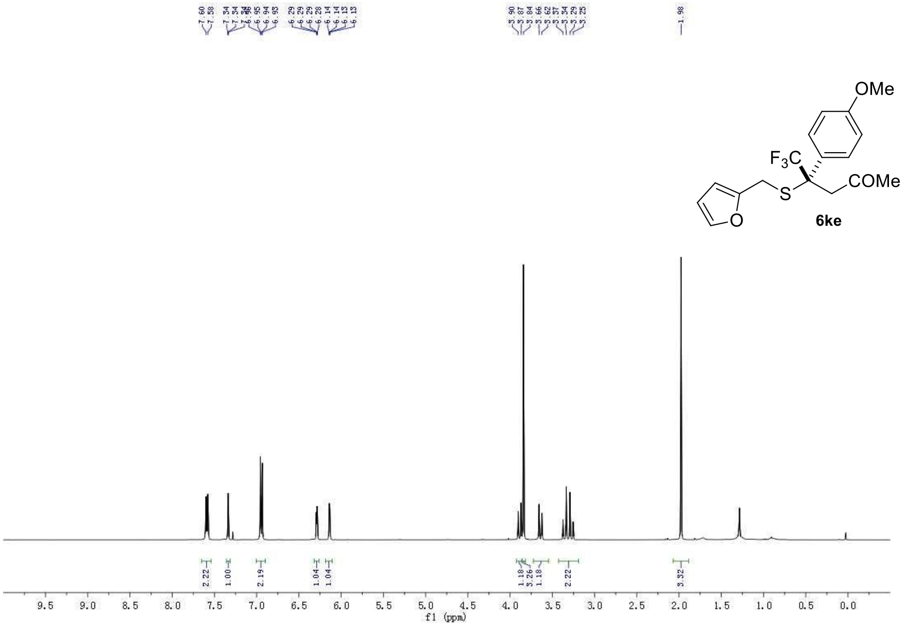

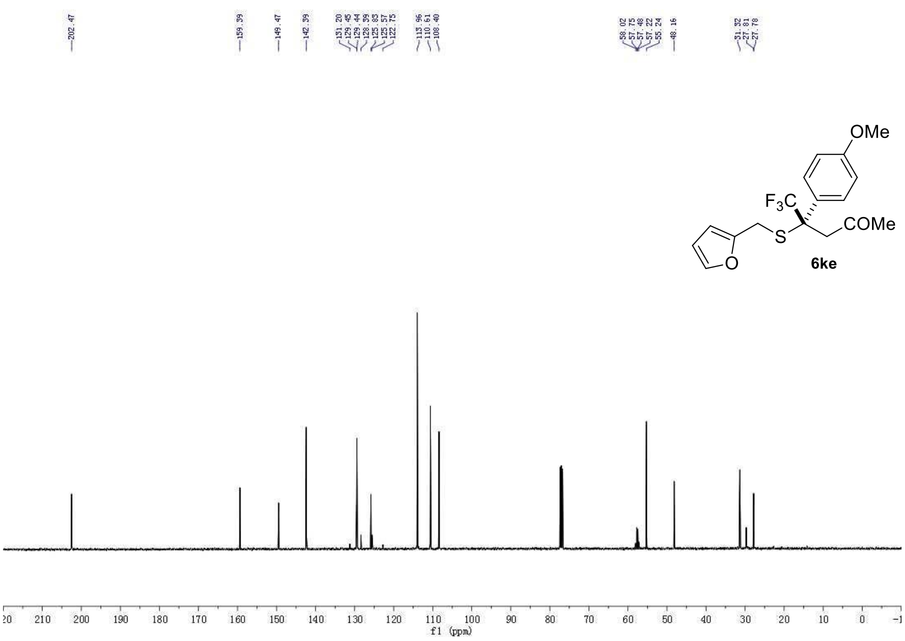

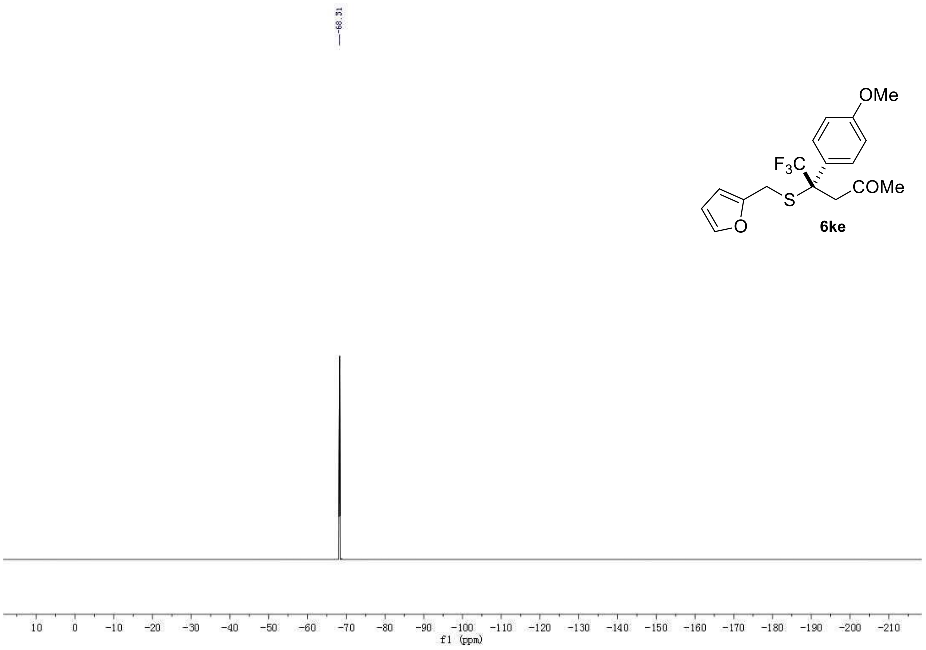

6ke

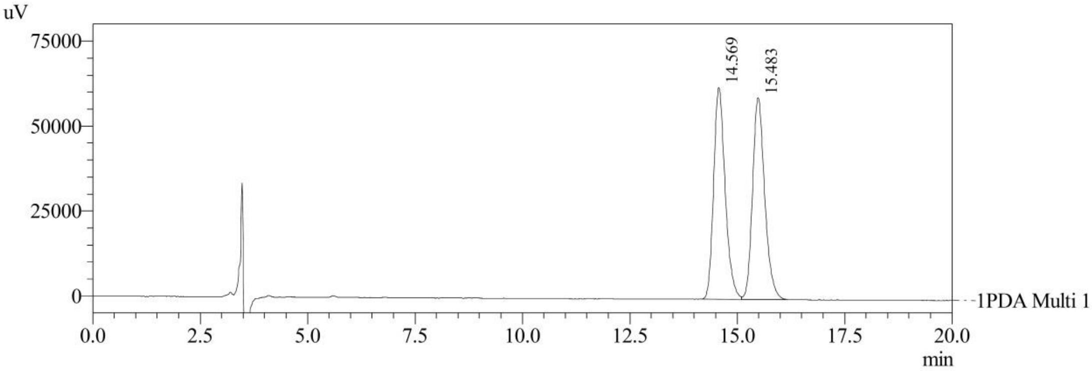

1 PDA Multi 1 / 210nm 4nm

PeakTable

PDA Ch1 210nm 4nm

Peak#	Ret. Time	Area	Height	Area %	Height %
1	14.569	1169205	62316	49.912	51.182
2	15.483	1173321	59438	50.088	48.818
Total		2342527	121755	100.000	100.000

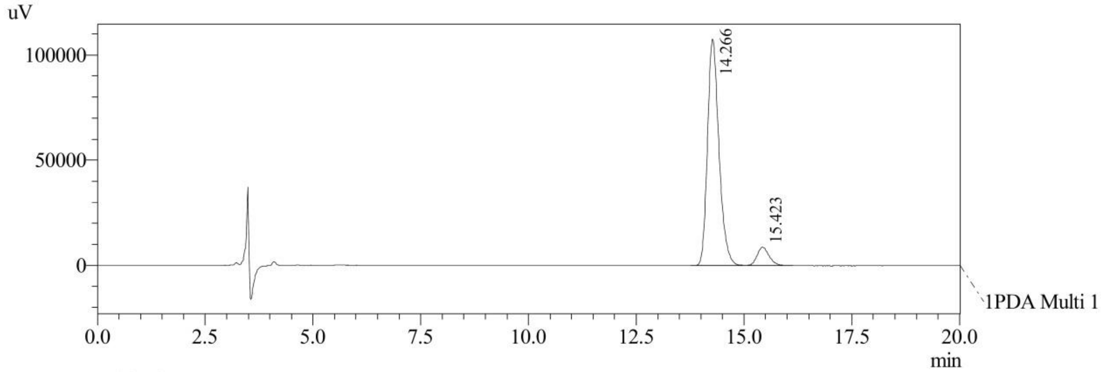

1 PDA Multi 1 / 210nm 4nm

PeakTable

PDA Ch1 210nm 4nm

Peak#	Ret. Time	Area	Height	Area %	Height %
1	14.266	2000209	107745	92.032	92.457
2	15.423	173176	8790	7.968	7.543
Total		2173385	116535	100.000	100.000

(S)-4-(benzylthio)-5,5,5-trifluoro-4-(naphthalen-2-yl)pentan-2-one (6lf)

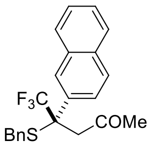

6lf

The title compound was prepared according to the general procedure B and purified by flash column chromatography (20:1 hexanes : EtOAc) to afford **6lf** (37 mg, 95%) as a colorless oil. Analytical data: IR (KBr, cm^{-1}) 2923, 2851, 1707, 1495, 1454, 1360, 1235, 1160, 1039, 815, 748, 715; ^{1}H NMR (400 MHz, $CDCl_3$) δ 8.17 (s, 1H), 8.03 – 7.80 (m, 4H), 7.68 – 7.53 (m, 2H), 7.35 – 7.16 (m, 5H), 3.90 (d, *J* = 11.3 Hz, 1H), 3.48 (dt, *J* = 25.3, 12.1 Hz, 3H), 1.97 (s, 3H); ^{19}F NMR (376 MHz, $CDCl_3$) δ -67.22 (s, 3F); ^{13}C NMR (100 MHz, $CDCl_3$) δ 202.37 (s), 135.73 (s), 132.75 (s), 132.70 (s), 131.79 (s), 129.35 (s), 128.66 (s), 128.65 (s), 127.81 (s), 127.79 (s), 127.54 (s), 127.48 (s), 127.34 (q, *J* = 282.0 Hz), 127.16 (s), 126.78 (s), 125.05 (s), 58.48 (q, J = 27.0 Hz), 48.35 (s), 35.62 (s), 31.34 (s). HPLC (AD-H, 2.5% EtOH in hexanes, 1.0 mL/min, 210 nm): t_{major} = 11.0 min, t_{minor} = 10.3 min, 83% ee; $^{25}[\alpha]_D$ = 72.8 ^{o} (c = 1.0 in $CHCl_3$); HRMS (ESI+) Calcd for $C_{22}H_{19}OF_3NaS^+$ $(M+Na)^+$: 411.1006, Found: 411.1000.

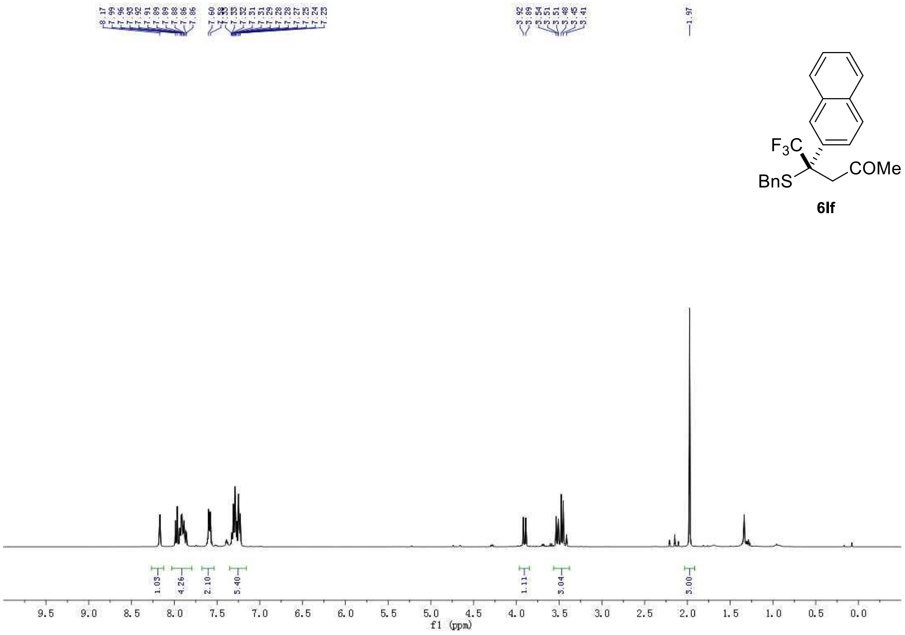

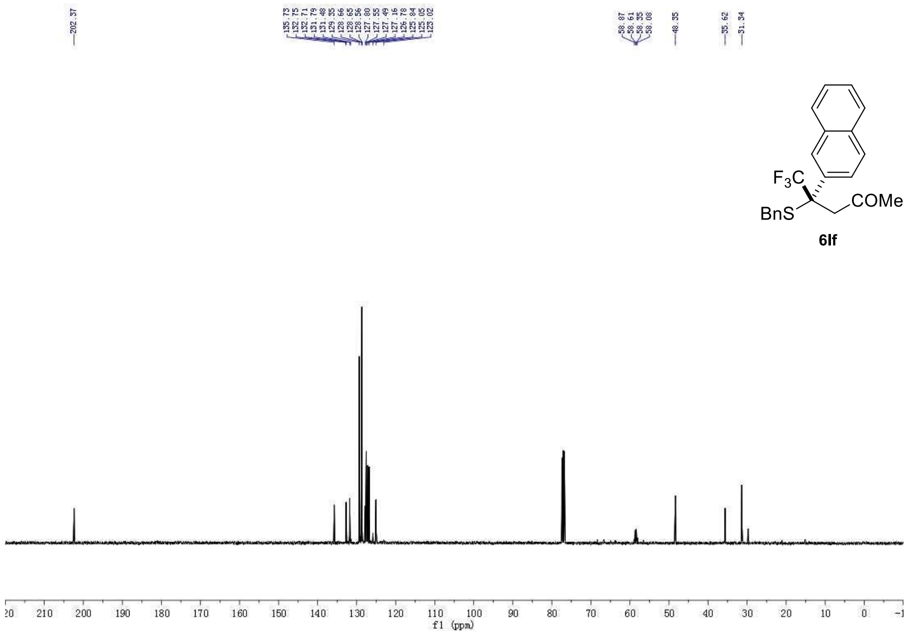

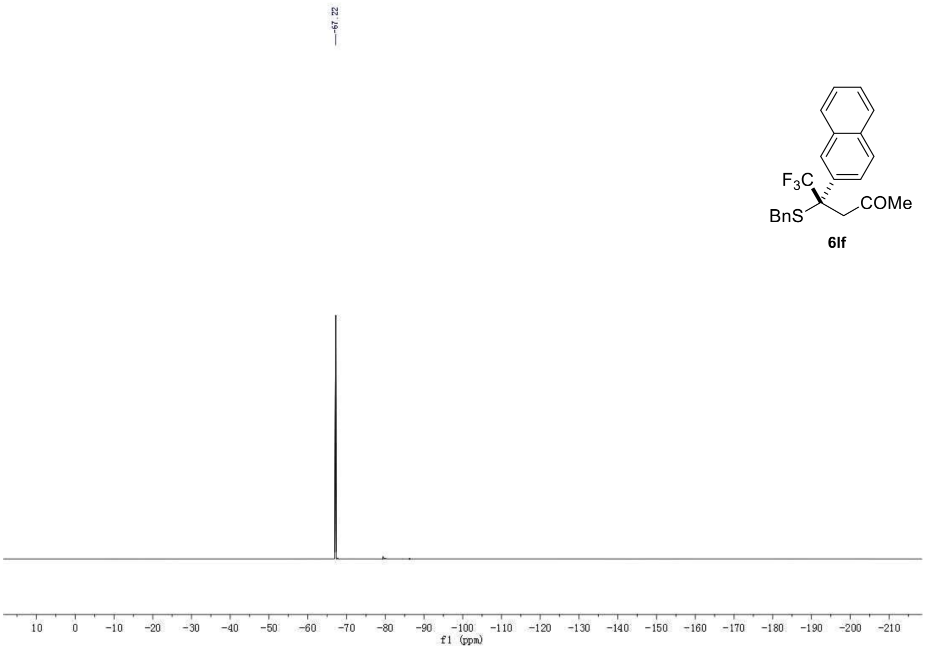

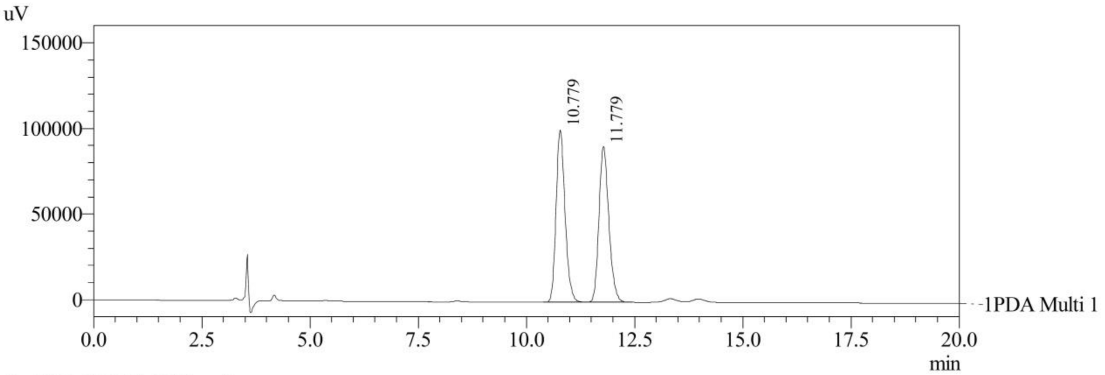

1 PDA Multi 1 / 210nm 4nm

PeakTable

PDA Ch1 210nm 4nm

Peak#	Ret. Time	Area	Height	Area %	Height %
1	10.779	1459814	100871	50.136	52.554
2	11.779	1451895	91066	49.864	47.446
Total		2911709	191936	100.000	100.000

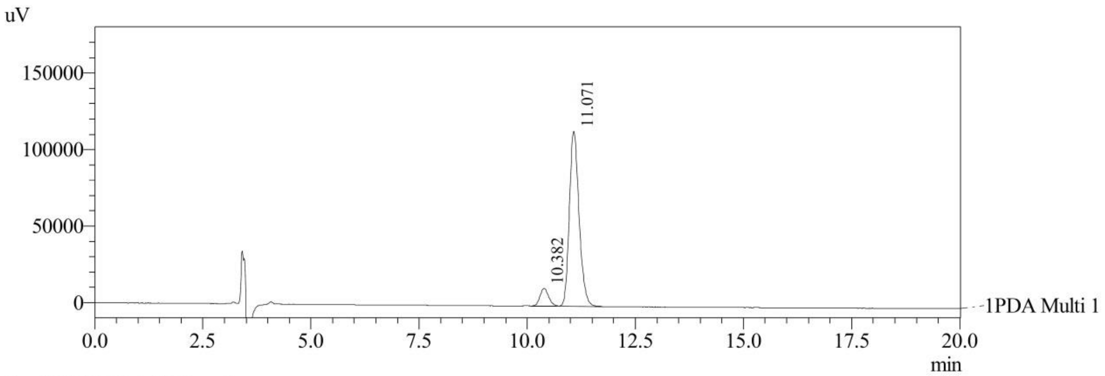

1 PDA Multi 1 / 210nm 4nm

PeakTable

PDA Ch1 210nm 4nm

Peak#	Ret. Time	Area	Height	Area %	Height %
1	10.382	162040	11615	8.320	9.199
2	11.071	1785630	114646	91.680	90.801
Total		1947670	126261	100.000	100.000

(R)-4-(benzylthio)-5,5,5-trifluoro-4-(thiophen-2-yl)pentan-2-one (6lg)

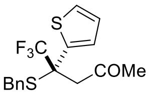

6lg

The title compound was prepared according to the general procedure B and purified by flash column chromatography (20:1 hexanes : EtOAc) to afford **6lg** (24 mg, 69%) as a colorless oil. Analytical data: IR (KBr, cm^{-1}) 2925, 2850, 1709, 1496, 1455, 1360, 1236, 1170, 1046, 711; ^{1}H NMR (400 MHz, $CDCl_3$) δ 7.43 (dd, J = 5.2, 1.1 Hz, 1H), 7.39 – 7.24 (m, 6H), 7.05 (dd, J = 5.2, 3.7 Hz, 1H), 3.98 (d, J = 11.0 Hz, 1H), 3.75 (d, J = 11.0 Hz, 1H), 3.30 (q, J = 14.7 Hz, 2H), 2.02 (s, 3H); ^{19}F NMR (376 MHz, $CDCl_3$) δ -69.55 (s, 3F); ^{13}C NMR (100 MHz, $CDCl_3$) δ 202.13 (s), 139.32 (s), 135.37 (s), 129.44 (s), 128.67 (s), 128.14 (s), 127.64 (s), 126.91 (s), 126.83 (s), 126.31 (q, J = 282.0 Hz), 55.88 (q, J = 28.0 Hz), 49.93 (s), 35.91 (d, J = 2.2 Hz), 31.35 (s). HPLC (AD-H, 2.5% EtOH in hexanes, 1.0 mL/min, 210 nm): t_{major} = 9.2 min, t_{minor} = 7.4 min, 88% ee; $^{25}[\alpha]_D$ = -0.7 ° (c = 1.0 in $CHCl_3$); HRMS (ESI+) Calcd for $C_{16}H_{15}OF_3NaS_2^+$ $(M+Na)^+$: 367.0414, Found: 367.0405.

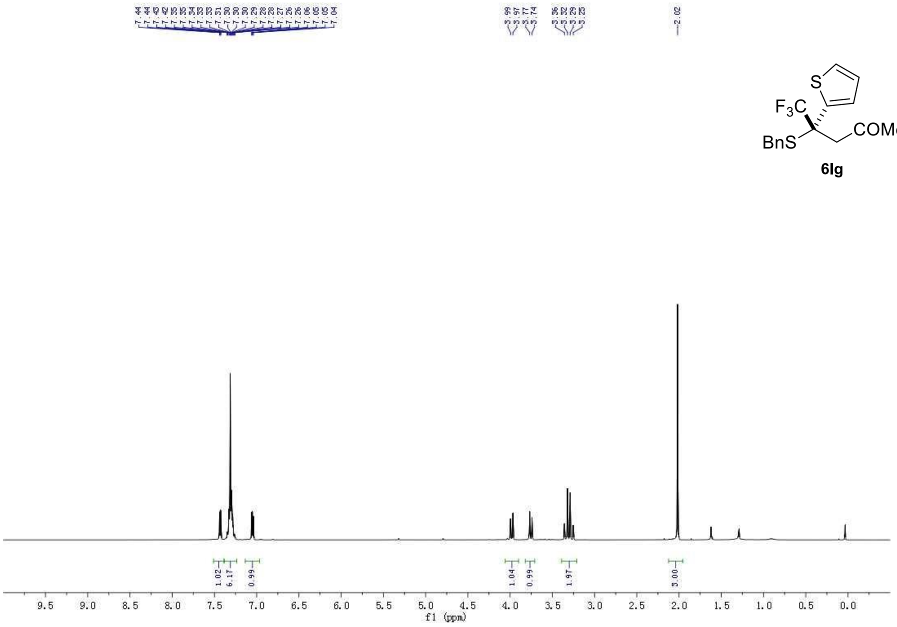

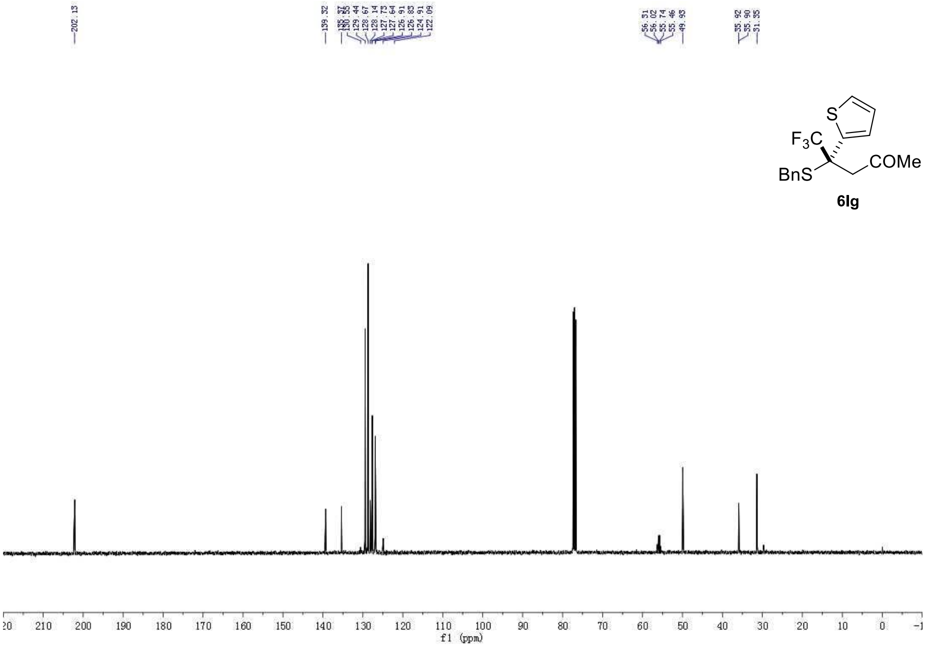

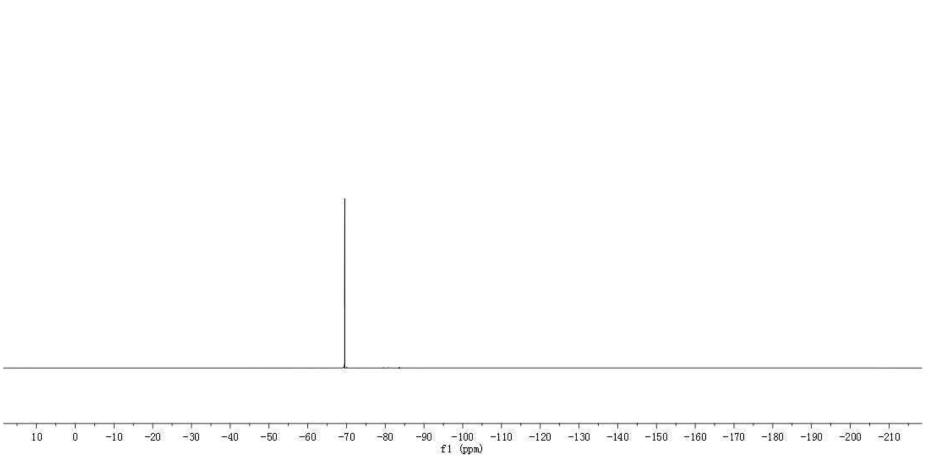

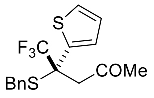

6lg

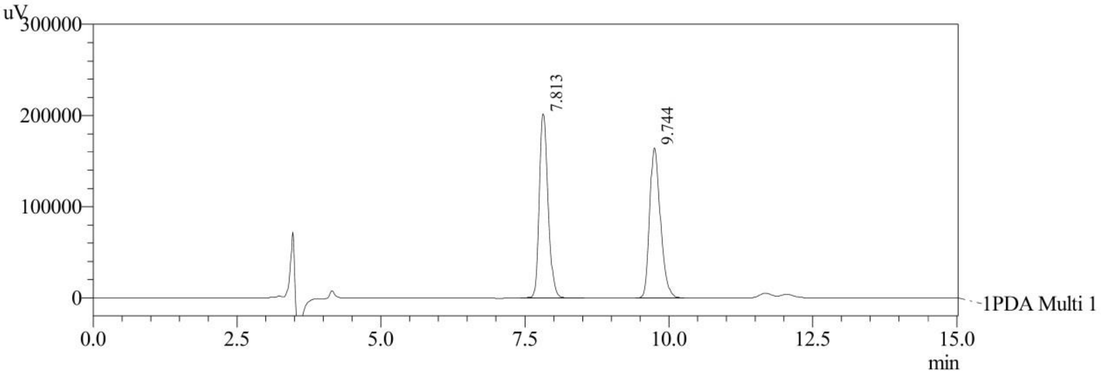

1 PDA Multi 1 / 210nm 4nm

PeakTable

PDA Ch1 210nm 4nm

Peak#	Ret. Time	Area	Height	Area %	Height %
1	7.813	2132297	202147	49.754	55.129
2	9.744	2153343	164535	50.246	44.871
Total		4285640	366682	100.000	100.000

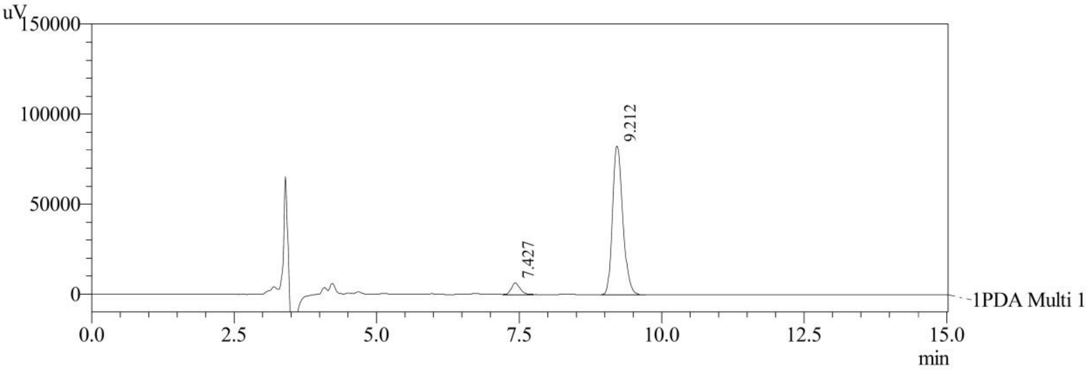

1 PDA Multi 1 / 210nm 4nm

PeakTable

PDA Ch1 210nm 4nm

Peak#	Ret. Time	Area	Height	Area %	Height %
1	7.427	66864	6462	6.058	7.246
2	9.212	1036933	82729	93.942	92.754
Total		1103798	89191	100.000	100.000

(S)-3-(benzylthio)-4,4,4-trifluoro-1,3-diphenylbutan-1-one (6lh)

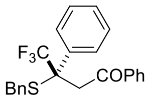

6lh

The title compound was prepared according to the general procedure B and purified by flash column chromatography (20:1 hexanes : EtOAc) to afford **6lh** (40 mg, 99%) as a colorless oil. Analytical data matched previously reported values.[3] ^{1}H NMR (400 MHz, $CDCl_3$) δ 7.94 – 7.86 (m, 2H), 7.71 (d, *J* = 7.9 Hz, 2H), 7.59 (t, *J* = 7.4 Hz, 1H), 7.50 – 7.34 (m, 5H), 7.31 – 7.19 (m, 5H), 4.07 (d, *J* = 16.9 Hz, 1H), 3.97 – 3.79 (m, 2H), 3.63 (d, *J* = 11.4 Hz, 1H); ^{19}F NMR (376 MHz, $CDCl_3$) δ -67.57 (s, 3F); ^{13}C NMR (100 MHz, $CDCl_3$) δ 193.35 (s), 137.01 (s), 135.97 (s), 134.95 (s), 133.34 (s), 129.29 (s), 128.62 (s), 128.58 (s), 128.42 (s), 128.19 (s), 128.09 (s), 128.04 (s), 127.42 (s), 127.27 (q, *J* = 282.0 Hz), 58.74 (q, J = 26.0 Hz), 41.86 (s), 35.86 (s). HPLC (AD-H, 2.5% EtOH in hexanes, 1.0 mL/min, 210 nm): t_{major} = 12.1 min, t_{minor} = 8.5 min, 70% ee; $^{25}[\alpha]_D$ = 21.7 º (c = 1.0 in $CHCl_3$); HRMS (ESI+) Calcd for $C_{23}H_{19}OF_3NaS^+$ $(M+Na)^+$: 423.1006, Found: 423.0999. The absolute stereochemistry was assigned as (S) by comparison to the sign of the specific rotation in the literature.[3]

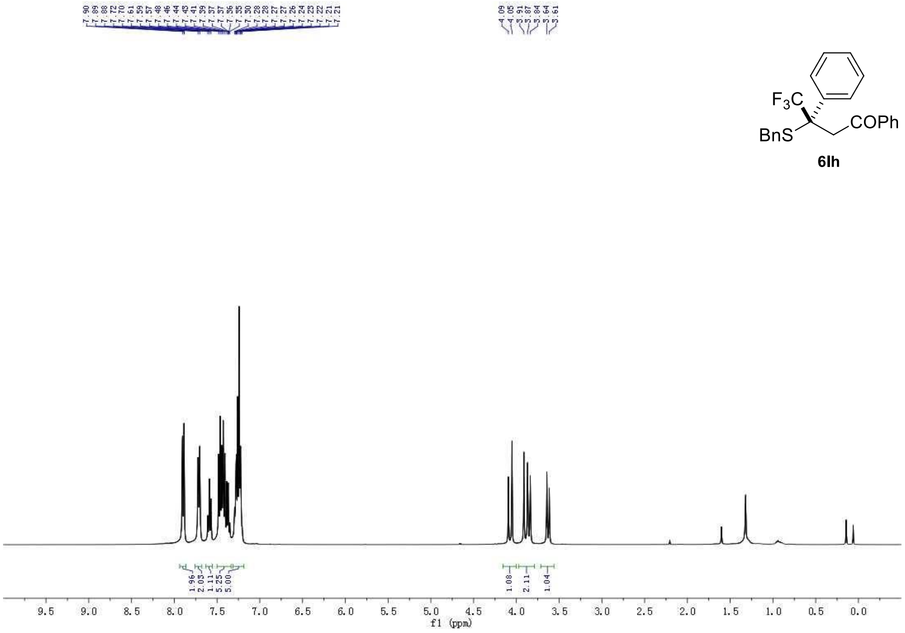

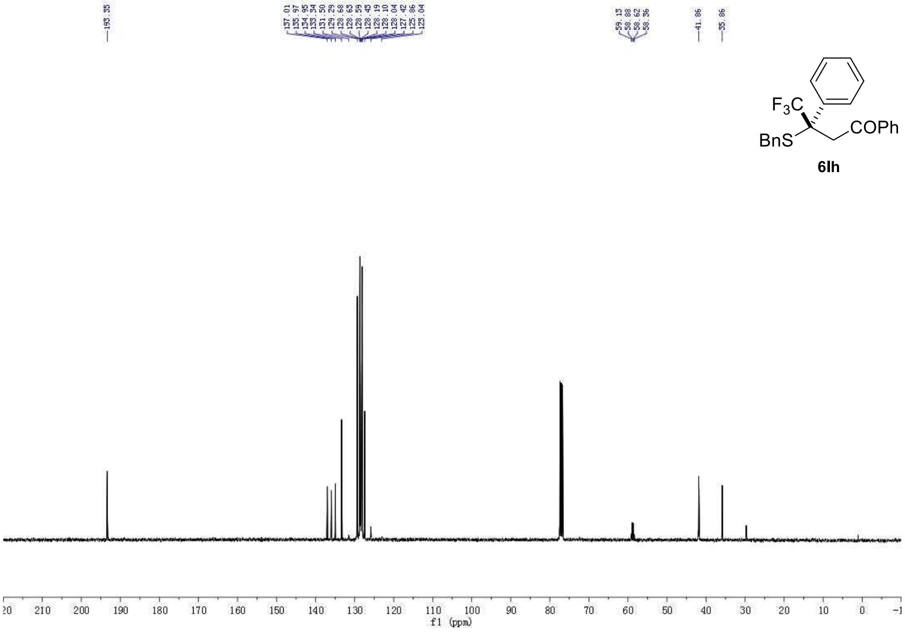

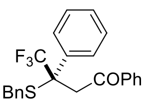

6lh

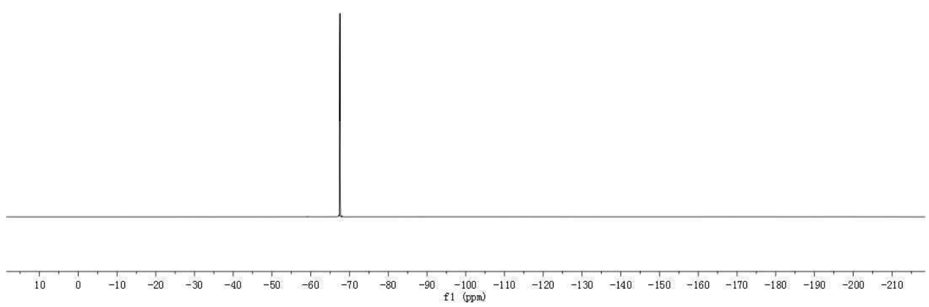

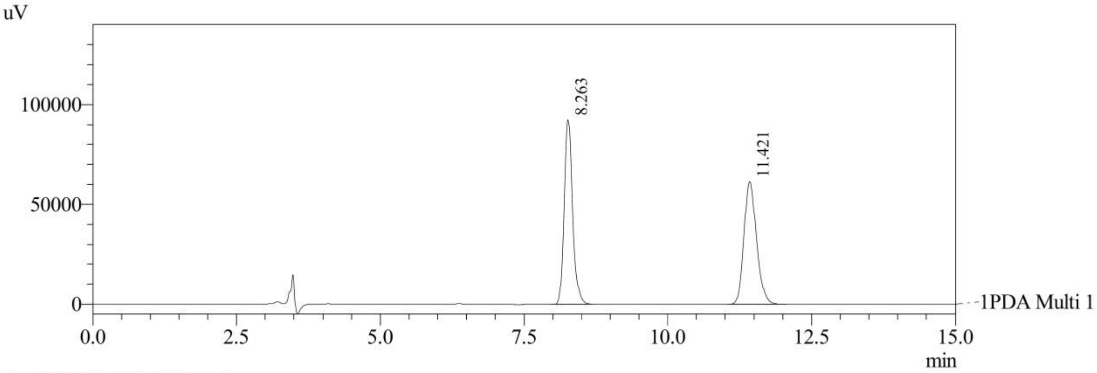

1 PDA Multi 1 / 210nm 4nm

PeakTable

PDA Ch1 210nm 4nm

Peak#	Ret. Time	Area	Height	Area %	Height %
1	8.263	937292	92634	50.015	60.130
2	11.421	936715	61422	49.985	39.870
Total		1874007	154057	100.000	100.000

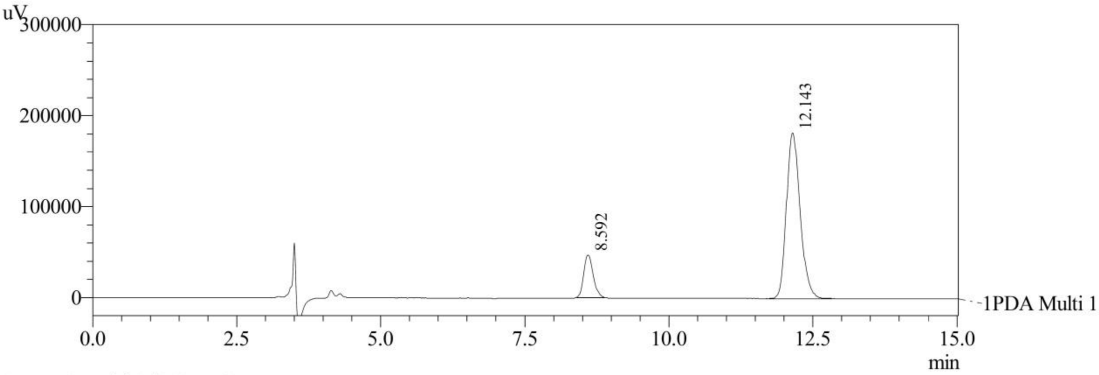

1 PDA Multi 1 / 210nm 4nm

PeakTable

PDA Ch1 210nm 4nm

Peak#	Ret. Time	Area	Height	Area %	Height %
1	8.592	535300	46878	15.025	20.436
2	12.143	3027396	182512	84.975	79.564
Total		3562696	229390	100.000	100.000

(S)-4-(benzylthio)pentan-2-one (8la)

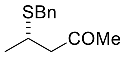

8la

The title compound was prepared according to the general procedure C and purified by flash column chromatography (20:1 hexanes : EtOAc) to afford **8la** (19 mg, 92%) as a colorless oil. Analytical data matched previously reported values.[4] ^{1}H NMR (300 MHz, $CDCl_3$) δ 7.39 – 7.18 (m, 5H), 3.76 (s, 2H), 3.27 – 3.04 (m, 1H), 2.68 (dd, *J* = 16.8, 5.9 Hz, 1H), 2.52 (dd, *J* = 16.8, 8.0 Hz, 1H), 2.08 (s, 3H), 1.27 (d, *J* = 6.7 Hz, 3H); ^{13}C NMR (75MHz, $CDCl_3$) δ 206.58 (s), 138.31 (s), 128.83 (s), 128.56 (s), 127.05 (s), 50.81 (s), 35.49 (s), 34.87 (s), 30.46 (s), 21.46 (s). HPLC (AD-H, 5% EtOH in hexanes, 1.0 mL/min, 210 nm): t_{major} = 7.1 min, t_{minor} = 6.5 min, 85% ee; $^{25}[\alpha]_D$ = 19.3 ^{o} (c = 1.0 in $CHCl_3$); HRMS (ESI+) Calcd for $C_{12}H_{16}ONaS^+$ $(M+Na)^+$: 231.0820, Found: 231.0814. The absolute stereochemistry was assigned as (S) by comparison to the sign of the specific rotation in the literature.[4]

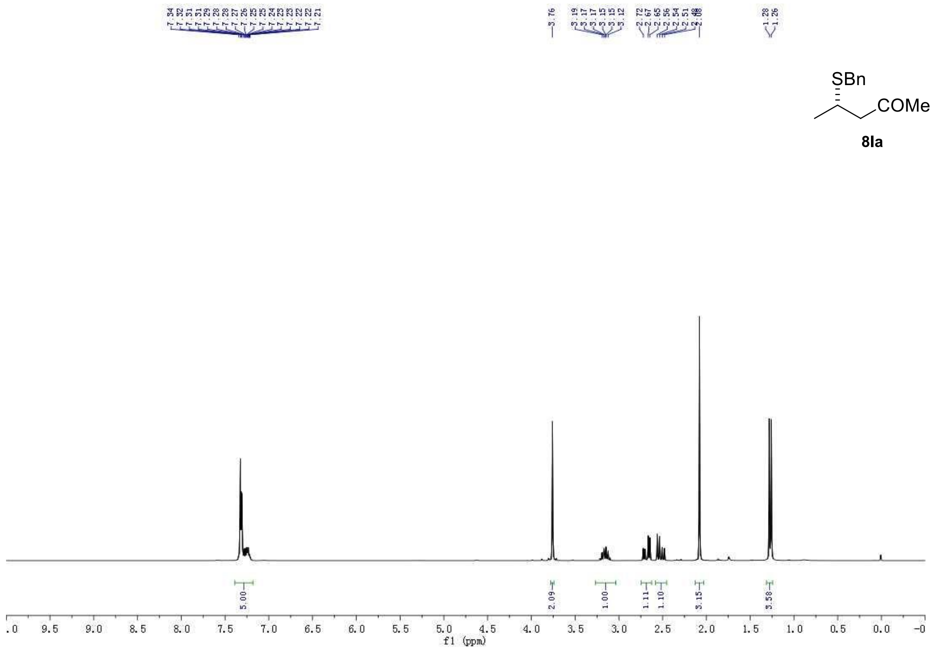

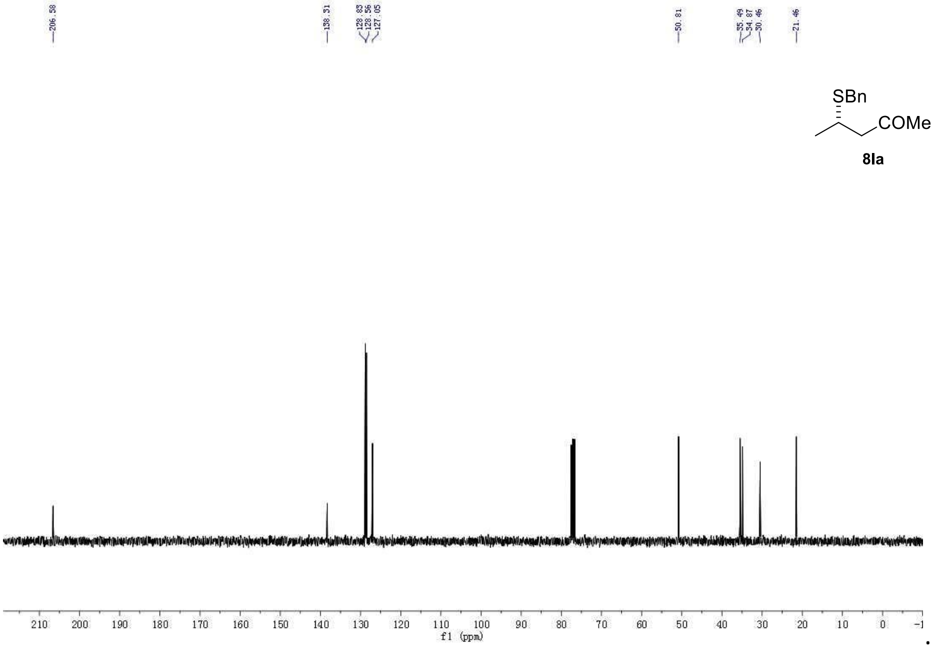

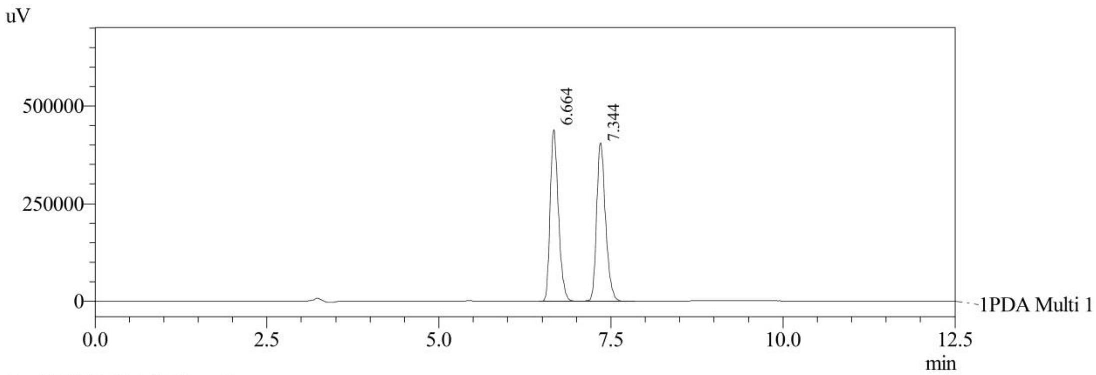

1 PDA Multi 1 / 210nm 4nm

PeakTable

PDA Ch1 210nm 4nm

Peak#	Ret. Time	Area	Height	Area %	Height %
1	6.664	3661468	439687	49.864	52.004
2	7.344	3681457	405794	50.136	47.996
Total		7342925	845482	100.000	100.000

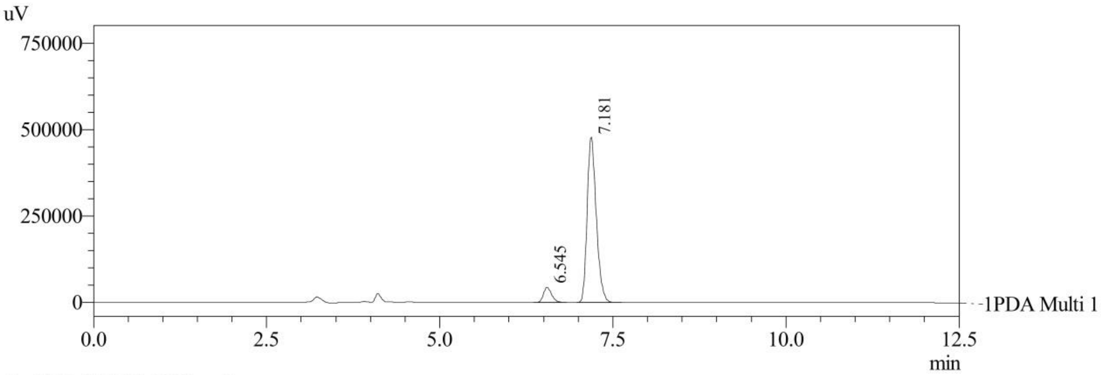

1 PDA Multi 1 / 210nm 4nm

PeakTable

PDA Ch1 210nm 4nm

Peak#	Ret. Time	Area	Height	Area %	Height %
1	6.545	373658	44285	7.857	8.494
2	7.181	4382241	477062	92.143	91.506
Total		4755899	521348	100.000	100.000

(S)-4-((furan-2-ylmethyl)thio)pentan-2-one (8ka)

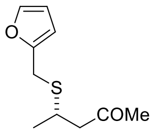

8ka

The title compound was prepared according to the general procedure C and purified by flash column chromatography (20:1 hexanes : EtOAc) to afford **8ka** (18 mg, 91%) as a colorless oil. Analytical data: IR (KBr, cm^{-1}) 2962, 2923, 1718, 1507, 1363, 1150, 1009, 740; ^1H NMR (400 MHz, $CDCl_3$) δ 7.36 (dd, *J* = 1.8, 0.8 Hz, 1H), 6.31 (dd, *J* = 3.1, 1.9 Hz, 1H), 6.19 (dd, *J* = 3.2, 0.6 Hz, 1H), 3.77 (s, 2H), 3.24 (ddd, *J* = 8.0, 6.8, 5.9 Hz, 1H), 2.70 (dd, *J* = 17.0, 5.8 Hz, 1H), 2.54 (dd, *J* = 16.9, 8.1 Hz, 1H), 2.12 (s, 3H), 1.27 (d, *J* = 6.8 Hz, 3H); ^{13}C NMR (100 MHz, $CDCl_3$) δ 206.42 (s), 151.63 (s), 142.00 (s), 110.48 (s), 107.38 (s), 50.70 (s), 35.09 (s), 30.42 (s), 27.51 (s), 21.28 (s). HPLC (OJ-H, 5% EtOH in hexanes, 1.0 mL/min, 210 nm): t_{major} = 14.4 min, t_{minor} = 11.4 min, 81% ee; $^{25}[\alpha]_D$ = 18.7 ° (c = 1.0 in $CHCl_3$); HRMS (ESI+) Calcd for $C_{10}H_{14}O_2NaS^+$ $(M+Na)^+$: 221.0612, Found: 221.0606.

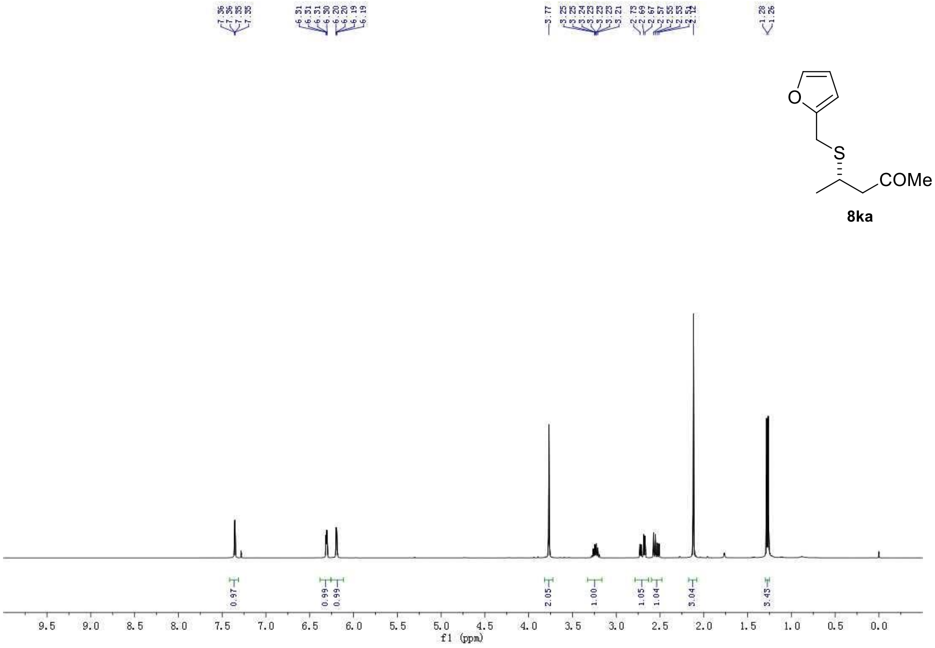

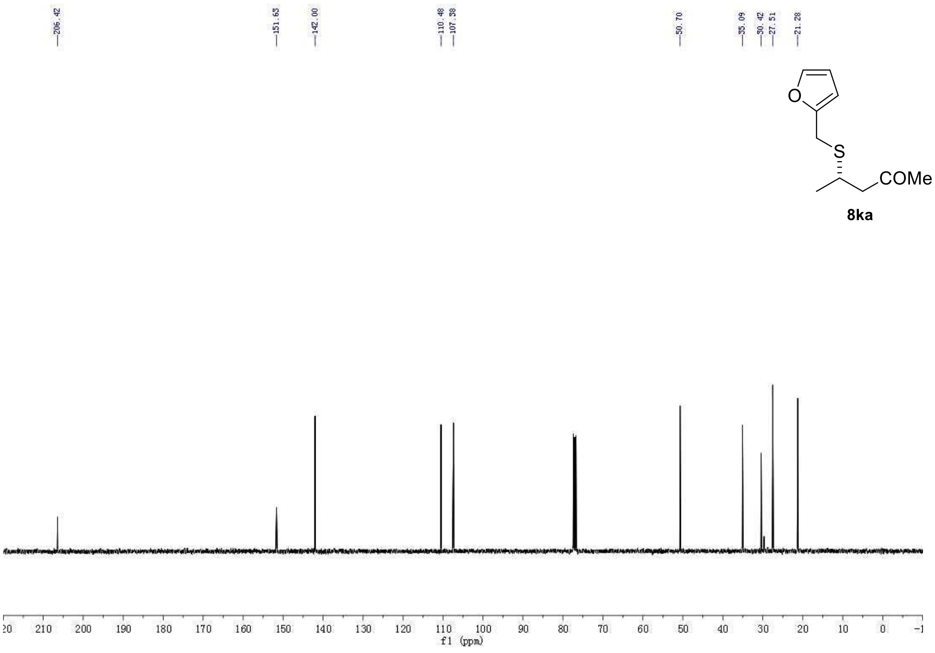

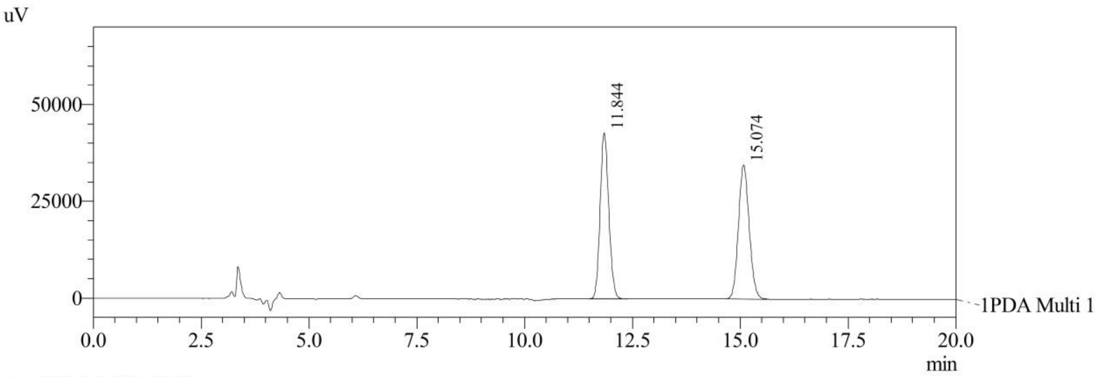

1 PDA Multi 1 / 210nm 4nm

PeakTable

PDA Ch1 210nm 4nm

Peak#	Ret. Time	Area	Height	Area %	Height %
1	11.844	598981	43024	49.998	55.417
2	15.074	599037	34613	50.002	44.583
Total		1198019	77638	100.000	100.000

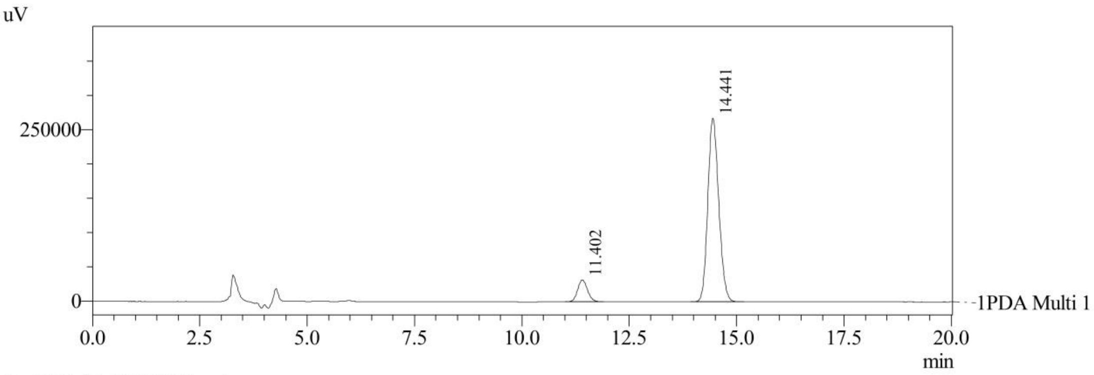

1 PDA Multi 1 / 210nm 4nm

PeakTable

PDA Ch1 210nm 4nm

Peak#	Ret. Time	Area	Height	Area %	Height %
1	11.402	505911	31743	9.638	10.620
2	14.441	4743038	267164	90.362	89.380
Total		5248949	298907	100.000	100.000

(S)-4-((3-fluorobenzyl)thio)pentan-2-one (8ma)

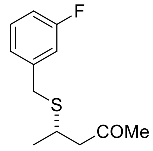

8ma

The title compound was prepared according to the general procedure C and purified by flash column chromatography (20:1 hexanes : EtOAc) to afford **8ma** (22 mg, 97%) as a colorless oil. Analytical data: IR (KBr, cm^{-1}) 2963, 2924, 1714, 1488, 1360, 1257, 1159, 1137, 944, 883, 787; ^{1}H NMR (300 MHz, $CDCl_3$) δ 7.26 (td, *J* = 8.3, 6.2 Hz, 1H), 7.07 (dd, *J* = 13.7, 4.7 Hz, 2H), 7.01 – 6.87 (m, 1H), 3.74 (s, 2H), 3.15 (dd, *J* = 14.0, 6.7 Hz, 1H), 2.69 (dd, *J* = 16.9, 6.0 Hz, 1H), 2.53 (dd, *J* = 16.9, 7.9 Hz, 1H), 2.10 (s, 3H), 1.26 (d, J = 6.7 Hz, 3H); ^{13}C NMR (75 MHz, $CDCl_3$) δ 206.43 (s), 162.86 (d, *J* = 245.3 Hz), 140.96 (d, *J* = 7.2 Hz), 129.97 (d, *J* = 8.3 Hz), 124.45 (d, *J* = 2.8 Hz), 115.69 (d, *J* = 21 Hz), 114.02 (d, *J* = 21 Hz), 50.75 (s), 35.08 (d, J = 2.25 Hz), 30.49 (s), 21.46 (s). HPLC (OJ-H, 2.5% EtOH in hexanes, 1.0 mL/min, 210 nm): t_{major} = 17.1 min, t_{minor} = 13.6 min, 78% ee; $^{25}[\alpha]_D$ = 14.0 ° (c = 1.0 in $CHCl_3$); HRMS (ESI+) Calcd for $C_{12}H_{15}OFNaS^+$ $(M+Na)^+$: 249.0725, Found: 249.0720.

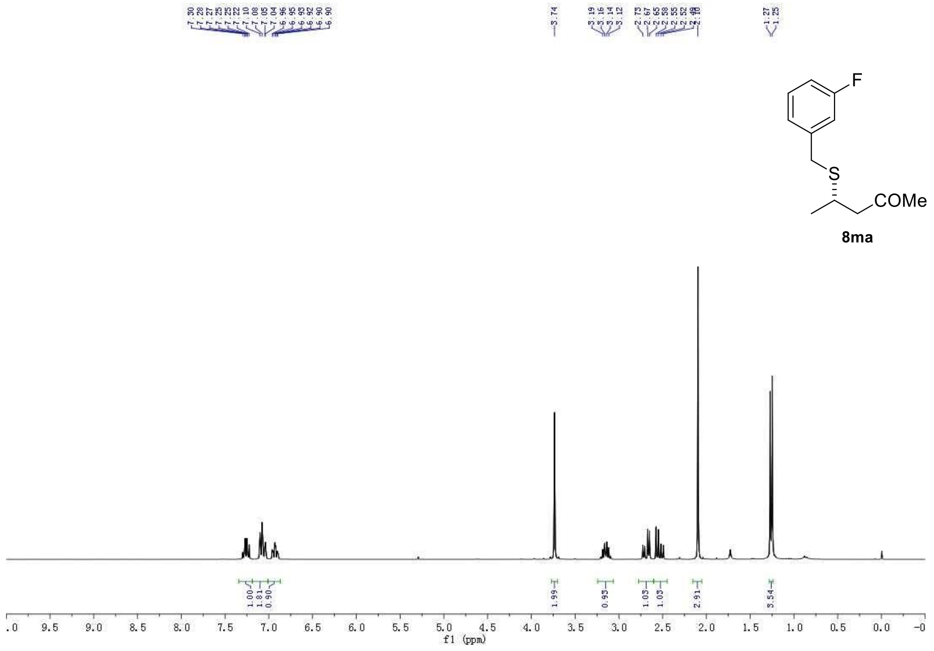

8ma

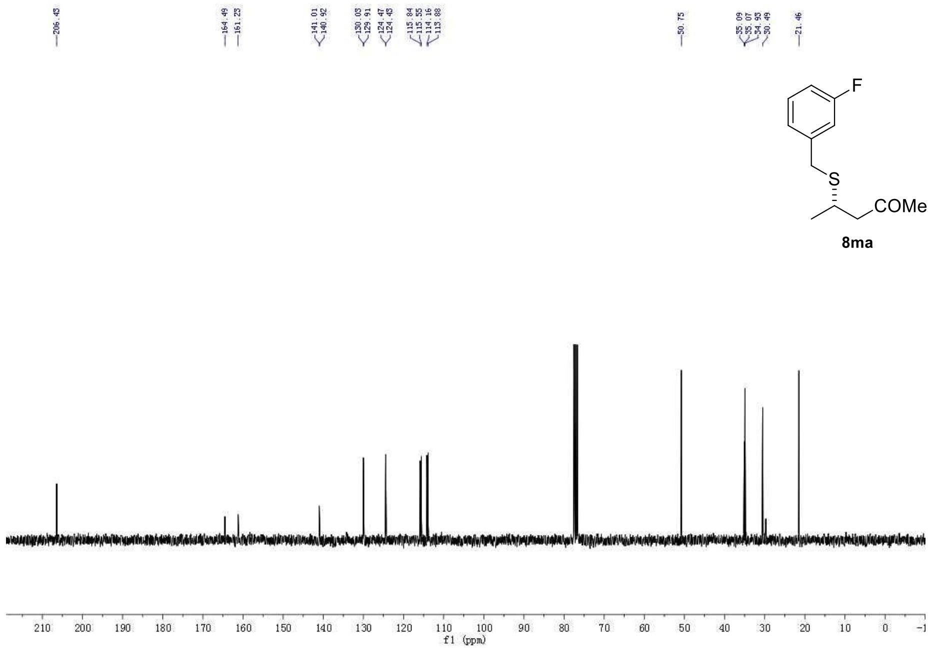

8ma

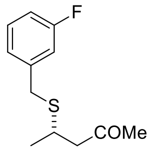

8ma

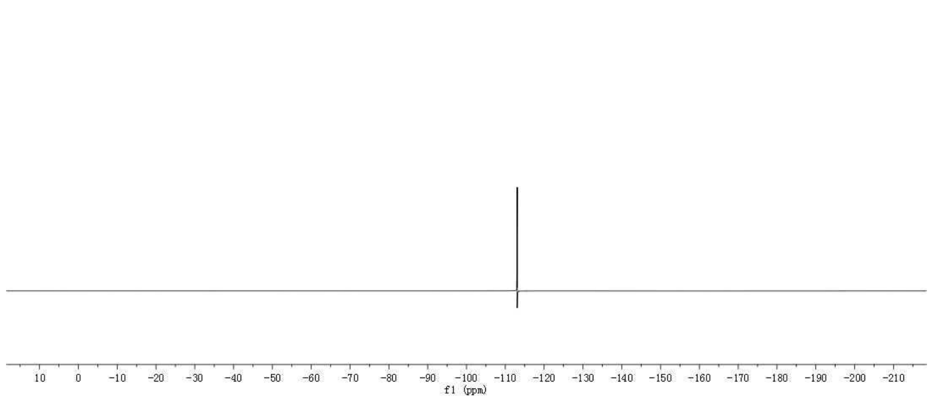

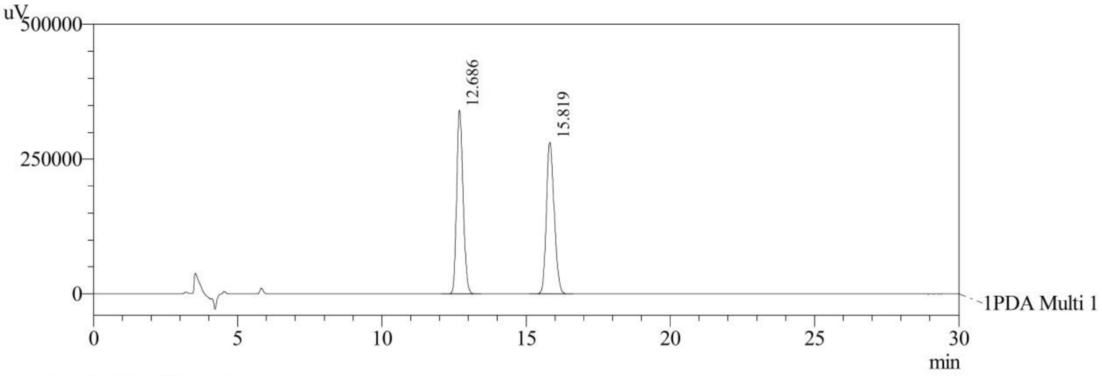

1 PDA Multi 1 / 210nm 4nm

PeakTable

PDA Ch1 210nm 4nm

Peak#	Ret. Time	Area	Height	Area %	Height %
1	12.686	5309220	341726	49.882	54.850
2	15.819	5334277	281289	50.118	45.150
Total		10643497	623016	100.000	100.000

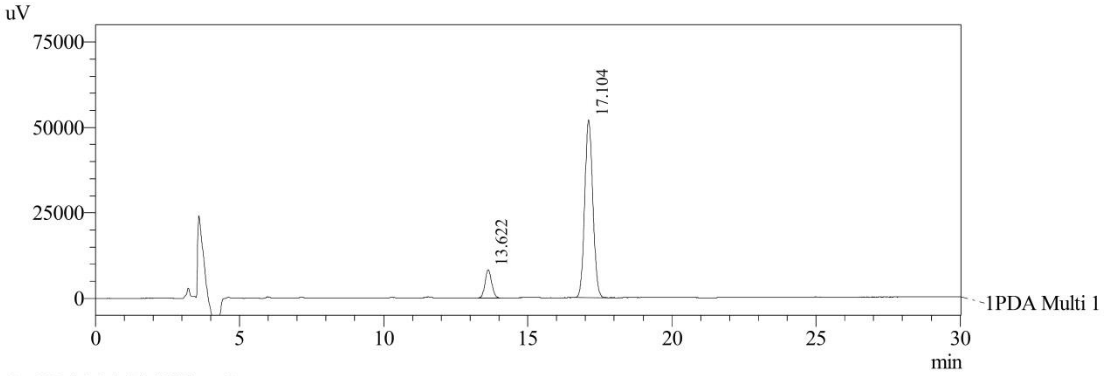

1 PDA Multi 1 / 210nm 4nm

PeakTable

PDA Ch1 210nm 4nm

Peak#	Ret. Time	Area	Height	Area %	Height %
1	13.622	129267	8167	11.235	13.570
2	17.104	1021294	52021	88.765	86.430
Total		1150561	60188	100.000	100.000

(S)-4-((4-methylbenzyl)thio)pentan-2-one (8na)

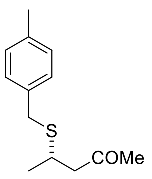

8na

The title compound was prepared according to the general procedure C and purified by flash column chromatography (20:1 hexanes : EtOAc) to afford **8na** (22 mg, 99%) as a colorless oil. Analytical data: IR (KBr, cm^{-1}) 2960, 2922, 1717, 1513, 1456, 1419, 1360, 1158, 819, 743; ^{1}H NMR (300 MHz, $CDCl_3$) δ 7.21 (d, *J* = 8.0 Hz, 2H), 7.12 (d, *J* = 7.9 Hz, 2H), 3.73 (s, 2H), 3.26 – 3.07 (m, 1H), 2.69 (dd, *J* = 16.8, 5.8 Hz, 1H), 2.52 (dd, *J* = 16.8, 8.1 Hz, 1H), 2.32 (d, *J* = 8.2 Hz, 3H), 2.08 (d, *J* = 7.0 Hz, 3H), 1.31 – 1.24 (d, J = 6.7 Hz, 3H); ^{13}C NMR (75 MHz, $CDCl_3$) δ 206.65 (s), 136.64 (s), 135.14 (s), 129.24 (s), 128.71 (s), 50.81 (s), 35.16 (s), 34.79 (s), 30.47 (s), 21.43 (s), 21.11 (s). HPLC (AD-H, 2.5% EtOH in hexanes, 1.0 mL/min, 210 nm): t_{major} = 8.2 min, t_{minor} = 7.5 min, 90% ee; $^{25}[\alpha]_D$ = 19.0 ° (c = 1.0 in $CHCl_3$); HRMS (ESI+) Calcd for $C_{13}H_{18}ONaS^+$ $(M+Na)^+$: 245.0976, Found: 245.0970.

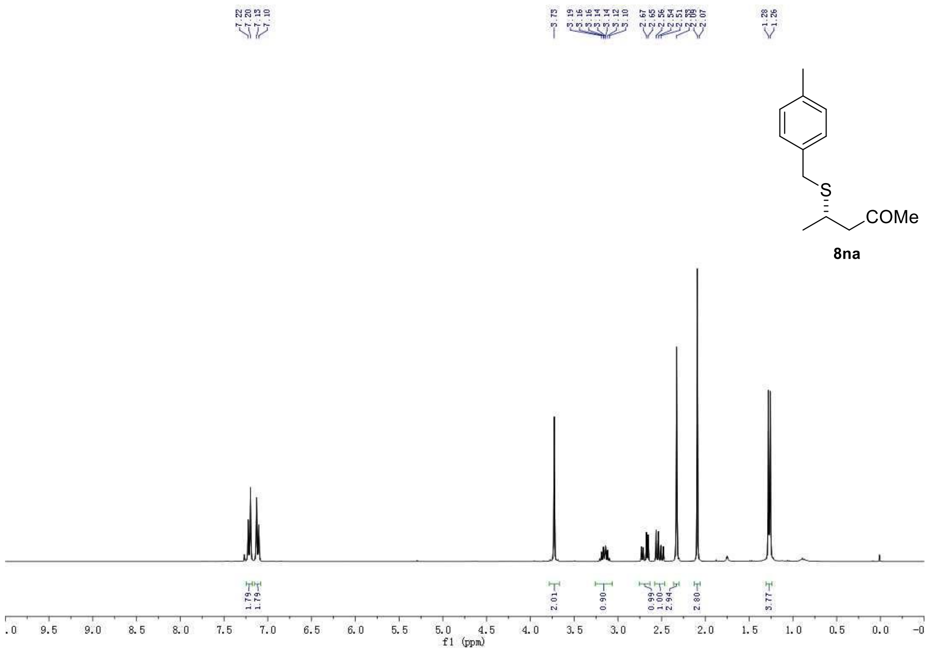

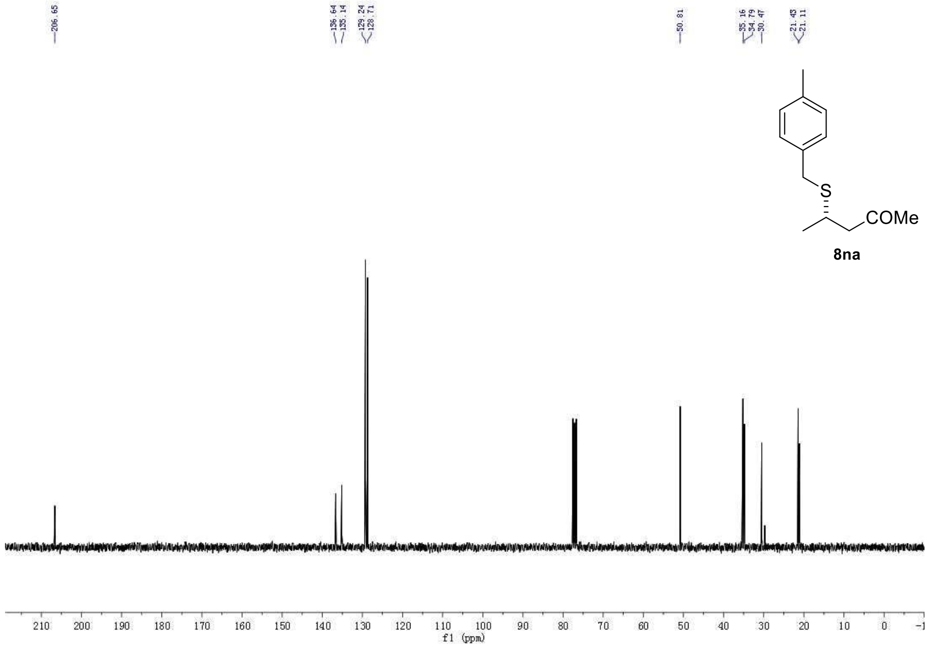

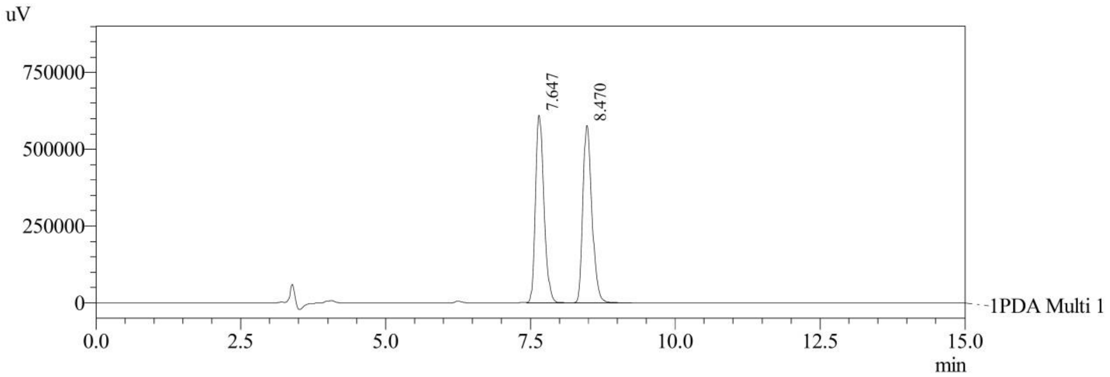

1 PDA Multi 1 / 210nm 4nm

PeakTable

PDA Ch1 210nm 4nm

Peak#	Ret. Time	Area	Height	Area %	Height %
1	7.647	6256852	611081	49.748	51.392
2	8.470	6320181	577983	50.252	48.608
Total		12577033	1189063	100.000	100.000

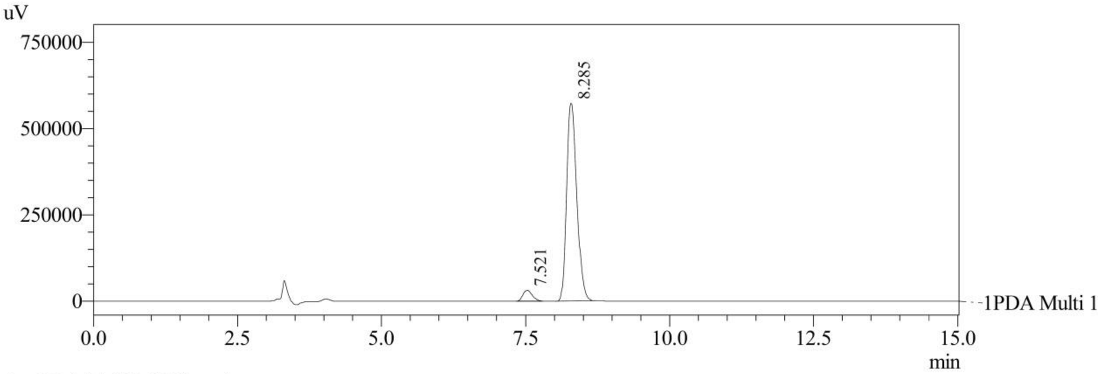

1 PDA Multi 1 / 210nm 4nm

PeakTable

PDA Ch1 210nm 4nm

Peak#	Ret. Time	Area	Height	Area %	Height %
1	7.521	361469	31514	4.899	5.218
2	8.285	7016209	572406	95.101	94.782
Total		7377678	603920	100.000	100.000

(S)-4-((2-methylbenzyl)thio)pentan-2-one (8pa)

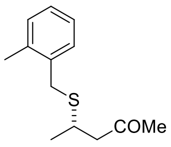

8pa

The title compound was prepared according to the general procedure C and purified by flash column chromatography (20:1 hexanes : EtOAc) to afford **8pa** (21 mg, 95%) as a colorless oil. Analytical data: IR (KBr, cm^{-1}) 2961, 2925, 1715, 1493, 1456, 1360, 1158, 1040, 768; ^{1}H NMR (400 MHz, $CDCl_3$) δ 7.23 (t, *J* = 5.7 Hz, 1H), 7.20 – 7.12 (m, 3H), 3.87 – 3.71 (m, 2H), 3.34 – 3.18 (m, 1H), 2.72 (dd, *J* = 16.8, 5.9 Hz, 1H), 2.57 (dd, *J* = 16.8, 8.0 Hz, 1H), 2.42 (s, 3H), 2.12 (s, 3H), 1.33 (d, J = 6.8 Hz, 3H); ^{13}C NMR (100 MHz, $CDCl_3$) δ 206.60 (s), 136.64 (s), 135.82 (s), 130.62 (s), 129.57 (s), 127.34 (s), 125.96 (s), 50.92 (s), 35.40 (s), 33.63 (s), 30.44 (s), 21.62 (s), 19.14 (s). HPLC (OJ-H, 5% EtOH in hexanes, 1.0 mL/min, 210 nm): t_{major} = 13.8 min, t_{minor} = 11.3 min, 89% ee; $^{25}[\alpha]_D$ = 25.8 ° (c = 1.0 in $CHCl_3$); HRMS (ESI+) Calcd for $C_{13}H_{18}ONaS^+$ $(M+Na)^+$: 245.0976, Found: 245.0972.

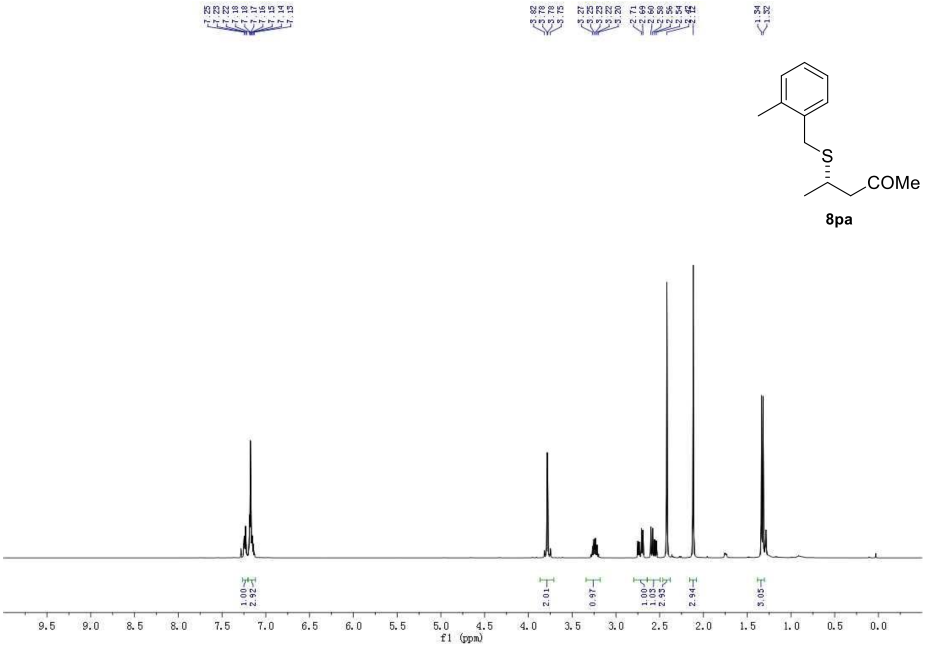

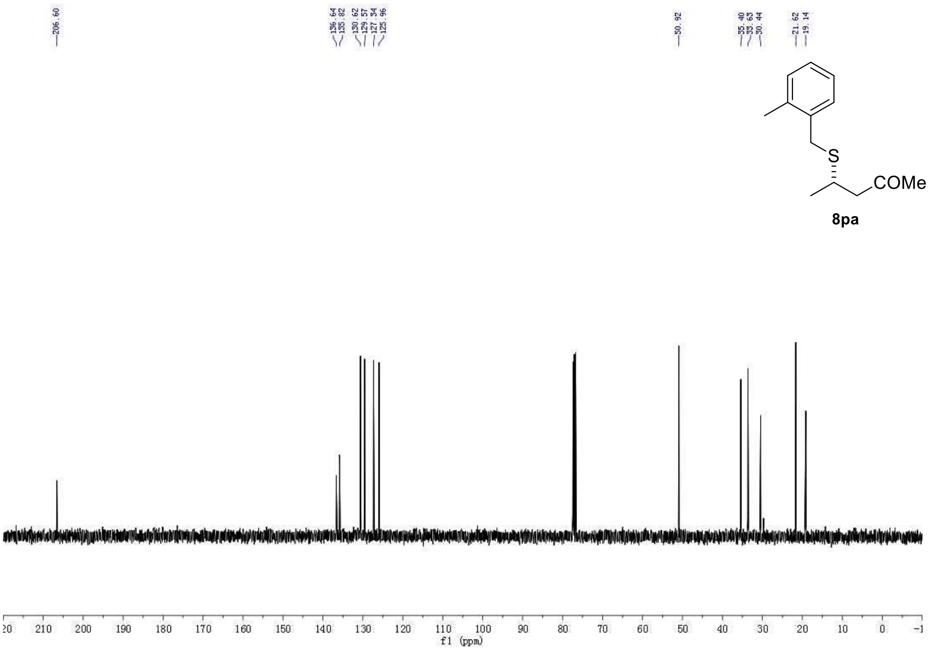

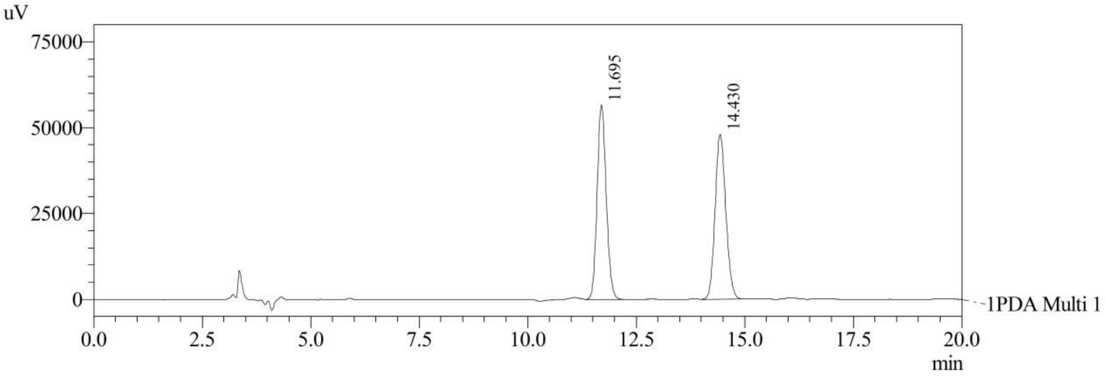

1 PDA Multi 1 / 210nm 4nm

PeakTable

PDA Ch1 210nm 4nm

Peak#	Ret. Time	Area	Height	Area %	Height %
1	11.695	828201	56739	49.953	54.102
2	14.430	829750	48135	50.047	45.898
Total		1657951	104875	100.000	100.000

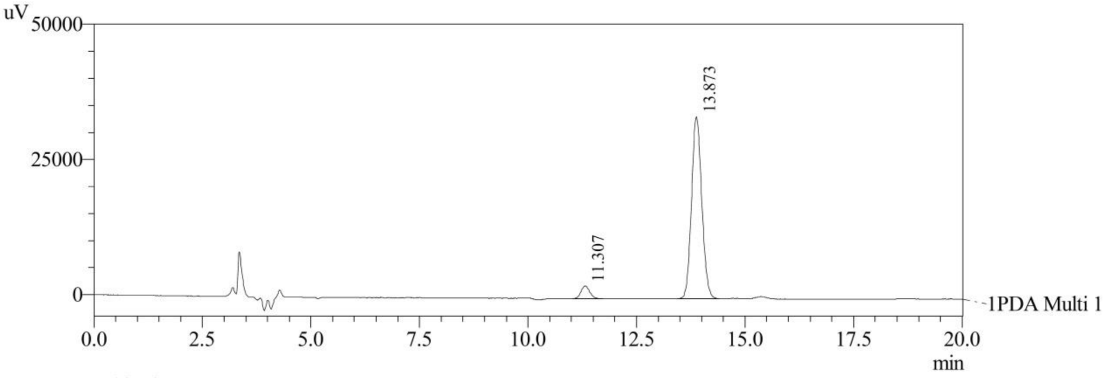

1 PDA Multi 1 / 210nm 4nm

PeakTable

PDA Ch1 210nm 4nm

Peak#	Ret. Time	Area	Height	Area %	Height %
1	11.307	33385	2388	5.696	6.618
2	13.873	552772	33688	94.304	93.382
Total		586157	36076	100.000	100.000

(S)-4-(benzylthio)-6-(5-methylfuran-2-yl)hexan-2-one (8lb)

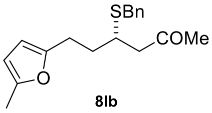

8lb

The title compound was prepared according to the general procedure C and purified by flash column chromatography (20:1 hexanes : EtOAc) to afford **8lb** (29 mg, 96%) as a colorless oil. Analytical data: IR (KBr, cm^{-1}) 2921, 2853, 1718, 1569, 1495, 1361, 1218, 738, 704; ^{1}H NMR (300 MHz, $CDCl_3$) δ 7.44 – 7.16 (m, 5H), 5.83 (dd, *J* = 6.8, 1.8 Hz, 2H), 3.85 – 3.65 (m, 2H), 3.18 – 2.98 (m, 1H), 2.80 – 2.52 (m, 4H), 2.25 (s, 3H), 2.08 (d, *J* = 12.8 Hz, 3H), 1.97 – 1.68 (m, 2H); ^{13}C NMR (75 MHz, $CDCl_3$) δ 206.64 (s), 153.26 (s), 150.38 (s), 138.43 (s), 128.96 (s), 128.54 (s), 127.07 (s), 105.90 (s), 105.81 (s), 49.59 (s), 39.76 (s), 35.70 (s), 33.37 (s), 30.36 (s), 25.28 (s), 13.56 (s). HPLC (OJ-H, 10% EtOH in hexanes, 1.0 mL/min, 210 nm): t_{major} = 24.3 min, t_{minor} = 14.0 min, 89% ee; $^{25}[\alpha]_D$ = 2.8 ° (c = 1.0 in $CHCl_3$); HRMS (ESI+) Calcd for $C_{18}H_{22}O_2NaS^+$ $(M+Na)^+$: 325.1238, Found: 325.1234.

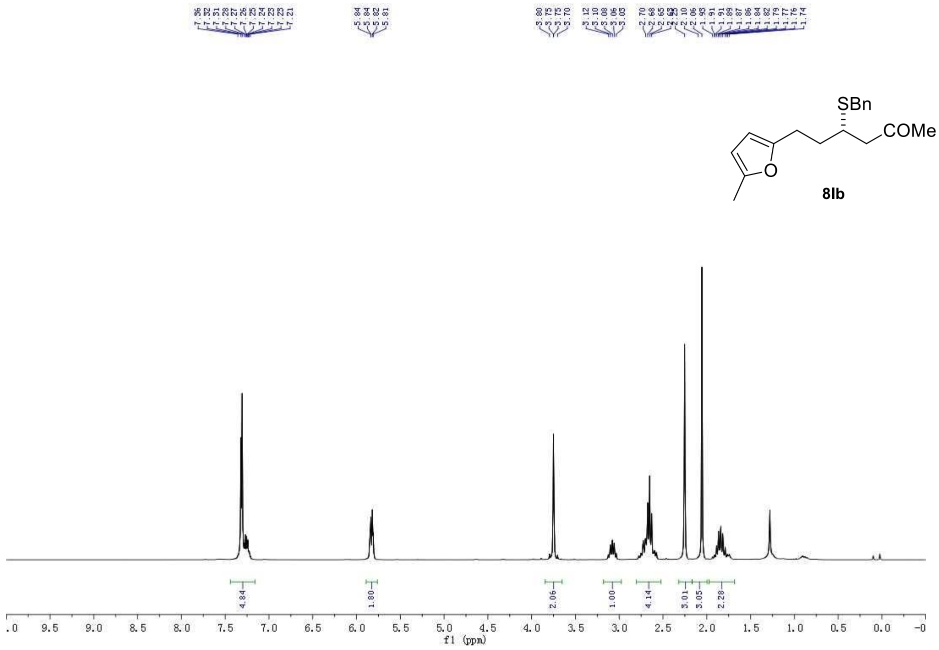

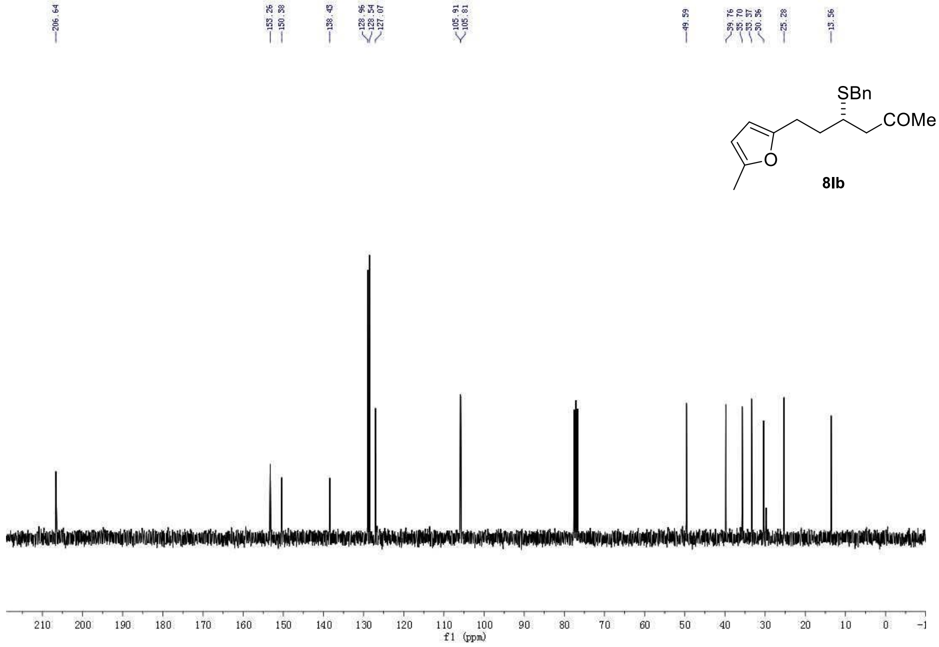

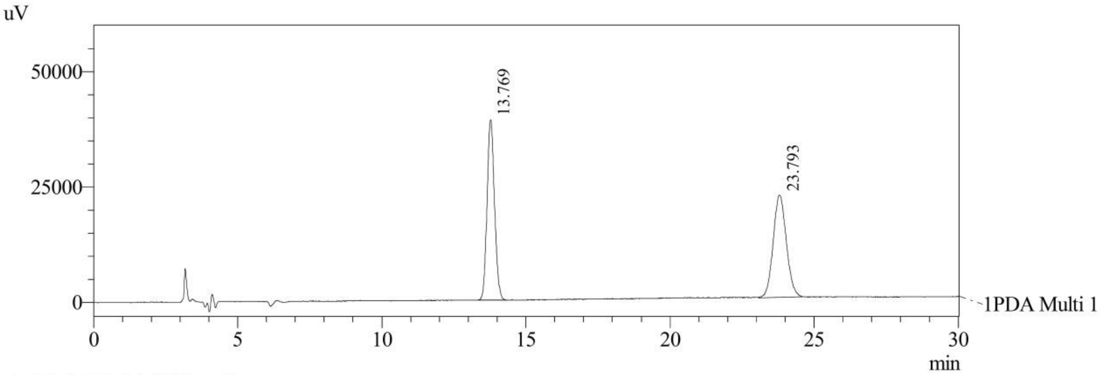

1 PDA Multi 1 / 210nm 4nm

PeakTable

PDA Ch1 210nm 4nm

Peak#	Ret. Time	Area	Height	Area %	Height %
1	13.769	702828	39073	49.994	63.857
2	23.793	702995	22115	50.006	36.143
Total		1405823	61188	100.000	100.000

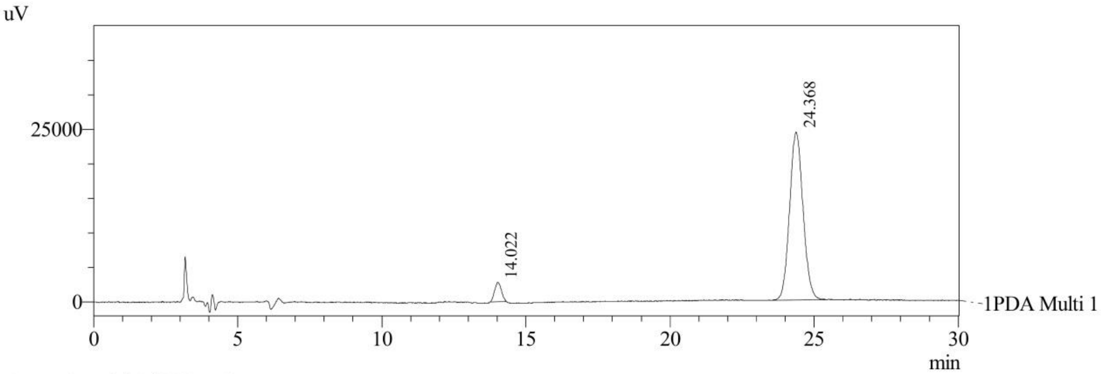

1 PDA Multi 1 / 210nm 4nm

PeakTable

PDA Ch1 210nm 4nm

Peak#	Ret. Time	Area	Height	Area %	Height %
1	14.022	46464	2832	5.561	10.440
2	24.368	789084	24298	94.439	89.560
Total		835547	27130	100.000	100.000

(S)-5-(benzylthio)hexan-3-one (8lc)

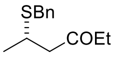

8lc

The title compound was prepared according to the general procedure C and purified by flash column chromatography (20:1 hexanes : EtOAc) to afford **8lc** (22 mg, 99%) as a colorless oil. Analytical data matched previously reported values.[5] ^{1}H NMR (300 MHz, $CDCl_3$) δ 7.45 – 7.14 (m, 5H), 3.85 – 3.65 (m, 2H), 3.28 – 3.08 (m, 1H), 2.66 (dd, *J* = 16.6, 6.0 Hz, 1H), 2.50 (dd, *J* = 16.6, 8.0 Hz, 1H), 2.42 – 2.29 (m, 2H), 1.26 (d, *J* = 6.7 Hz, 3H), 1.03 (t, *J* = 7.3 Hz, 3H); ^{13}C NMR (75 MHz, $CDCl_3$) δ 209.28 (s), 138.35 (s), 128.82 (s), 128.54 (s), 127.02 (s), 49.58 (s), 36.59 (s), 35.57 (s), 35.08 (s), 21.54 (s), 7.64 (s). HPLC (OJ-H, 2.5% EtOH in hexanes, 1.0 mL/min, 210 nm): t_{major} = 17.2min, t_{minor} = 13.3 min, 83% ee; $^{25}[\alpha]_D$ = 18.4 ° (c = 1.0 in $CHCl_3$); HRMS (ESI+) Calcd for $C_{13}H_{18}ONaS^+$ $(M+Na)^+$: 245.0976, Found: 245.0973. The absolute stereochemistry was assigned as (S) by comparison to the sign of the specific rotation in the literature.[5]

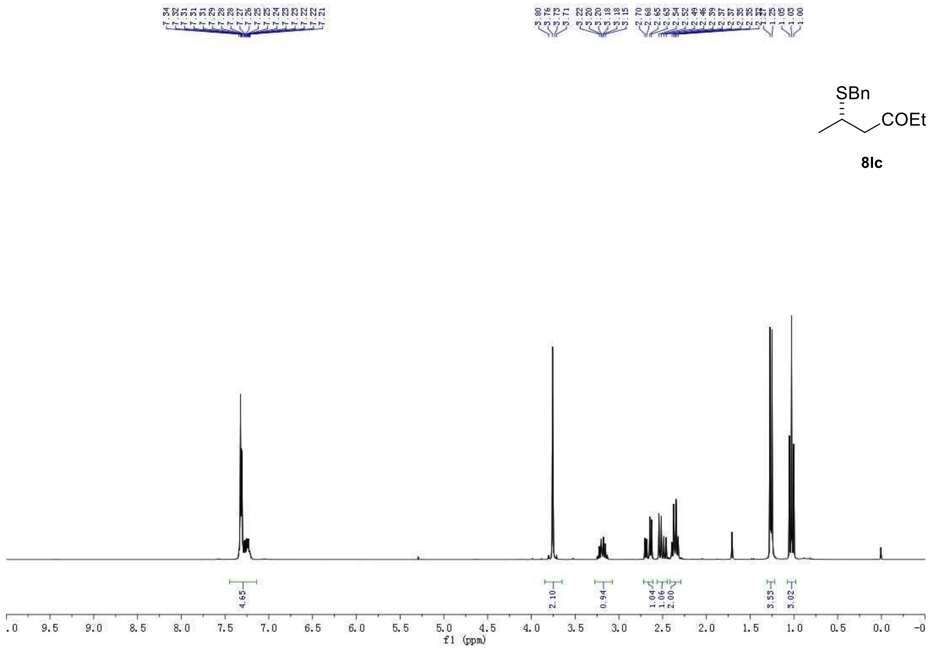

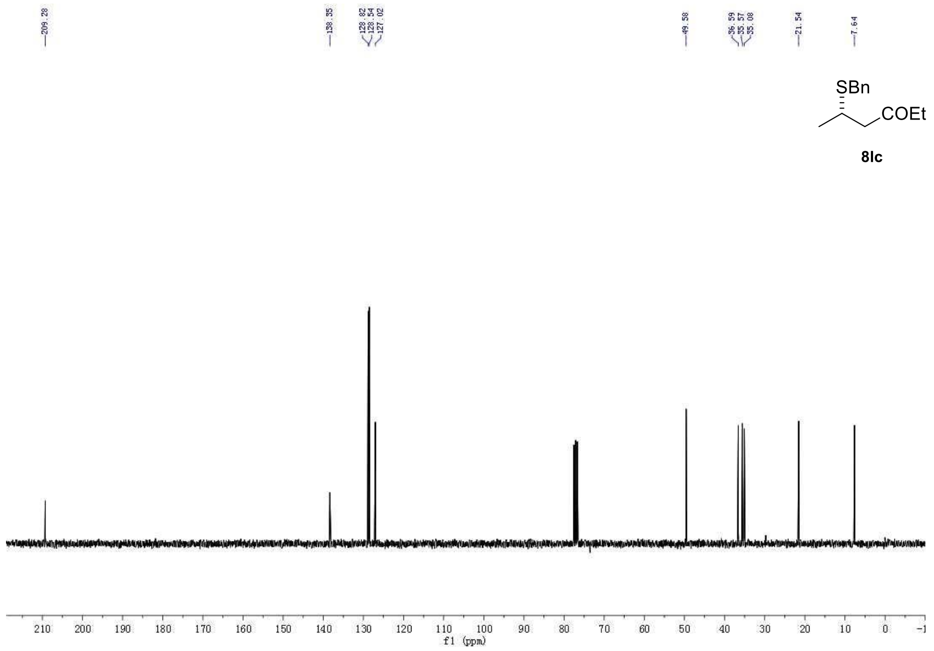

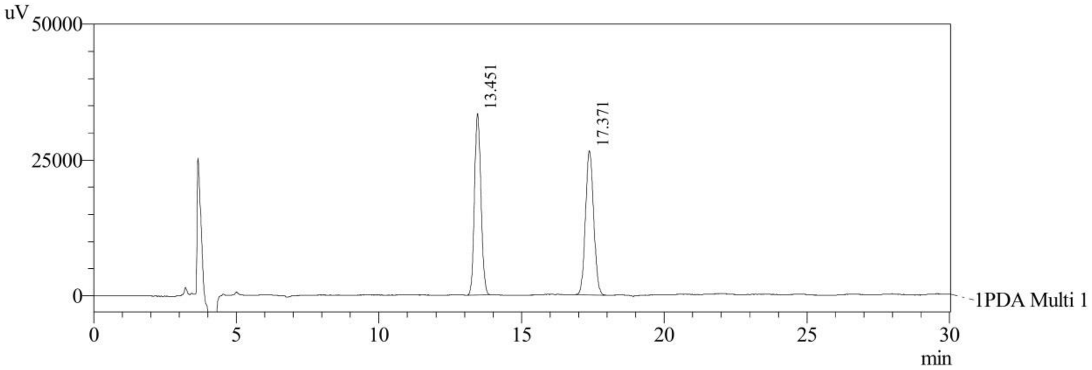

1 PDA Multi 1 / 210nm 4nm

PeakTable

PDA Ch1 210nm 4nm

Peak#	Ret. Time	Area	Height	Area %	Height %
1	13.451	525136	33445	49.813	55.690
2	17.371	529073	26611	50.187	44.310
Total		1054209	60055	100.000	100.000

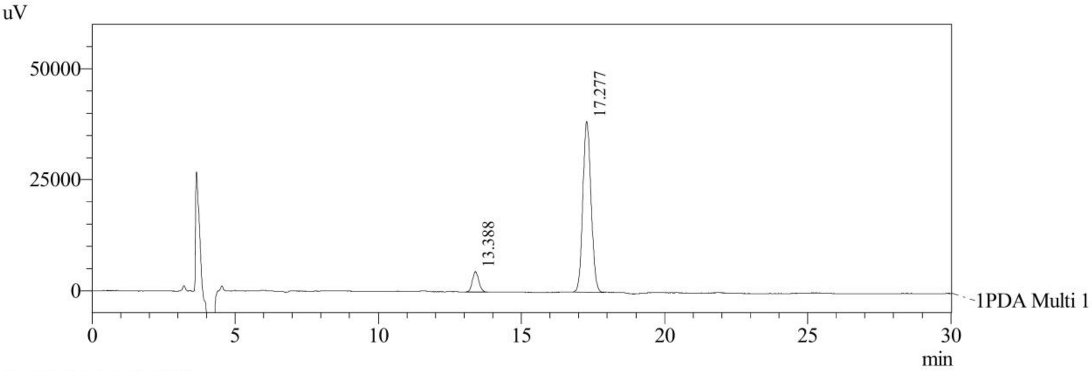

1 PDA Multi 1 / 210nm 4nm

PeakTable

PDA Ch1 210nm 4nm

Peak#	Ret. Time	Area	Height	Area %	Height %
1	13.388	72050	4590	8.550	10.639
2	17.277	770637	38553	91.450	89.361
Total		842687	43143	100.000	100.000

(S)-3-(benzylthio)-1-phenylbutan-1-one (8ld)

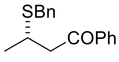

8ld

The title compound was prepared according to the general procedure C and purified by flash column chromatography (20:1 hexanes : EtOAc) to afford **8ld** (24 mg, 90%) as a colorless oil. Analytical data: IR (KBr, cm^{-1}) 2965, 2921, 1684, 1598, 1495, 1448, 1354,, 1181, 1071, 989, 753, 690; ^{1}H NMR (300 MHz, $CDCl_3$) δ 7.94 – 7.86 (m, 2H), 7.62 – 7.51 (m, 1H), 7.51 – 7.40 (m, 2H), 7.40 – 7.19 (m, 5H), 3.89 – 3.75 (m, 2H), 3.48 – 3.33 (m, 1H), 3.27 (dd, *J* = 16.7, 5.0 Hz, 1H), 3.06 (dd, *J* = 16.7, 8.6 Hz, 1H), 1.35 (d, *J* = 6.7 Hz, 3H); ^{13}C NMR (75 MHz, $CDCl_3$) δ 198.09 (s), 138.38 (s), 136.86 (s), 133.23 (s), 128.85 (s), 128.65 (s), 128.58 (s), 128.11 (s), 127.04 (s), 46.08 (s), 35.66 (s), 35.46 (s), 21.54 (s). HPLC (AD-H, 1% EtOH in hexanes, 1.0 mL/min, 210 nm): t_{major} = 12.6 min, t_{minor} = 13.8 min, 84% ee; $^{25}[\alpha]_D$ = -29.2 ° (c = 1.0 in $CHCl_3$); HRMS (ESI+) Calcd for $C_{17}H_{18}ONaS^+$ $(M+Na)^+$: 293.0976, Found: 293.0969.

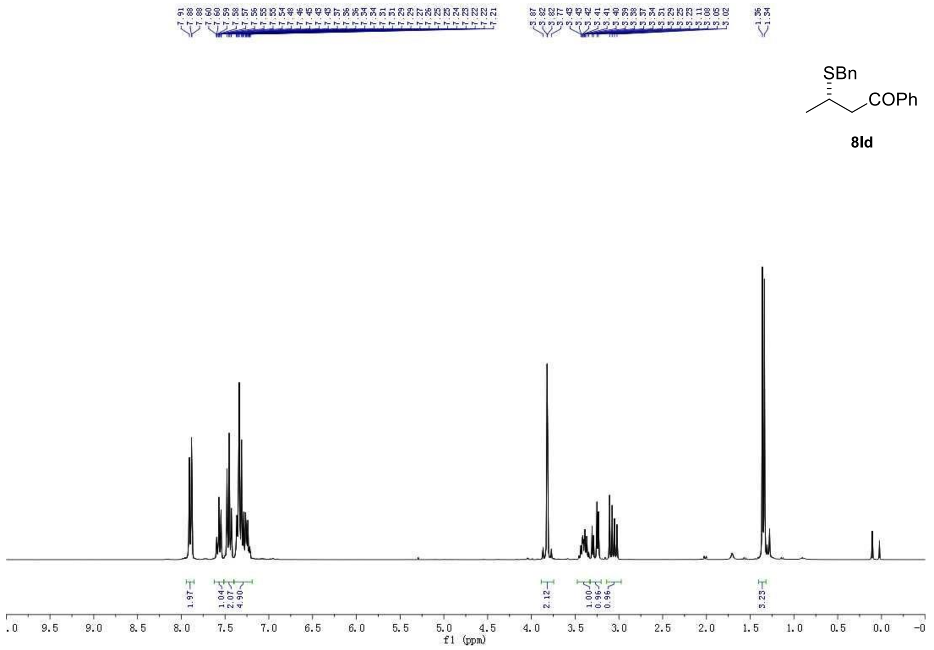

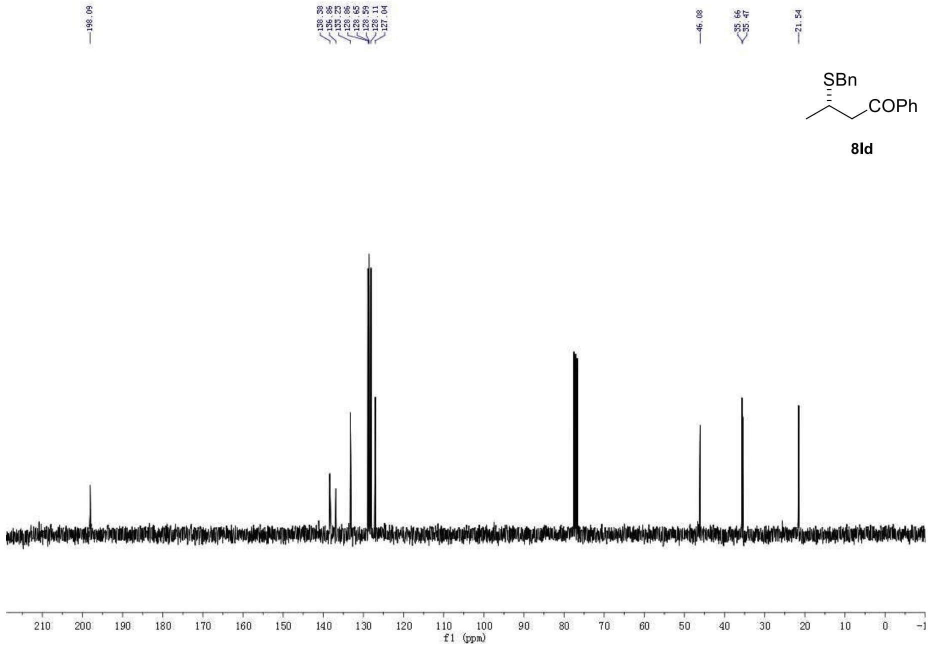

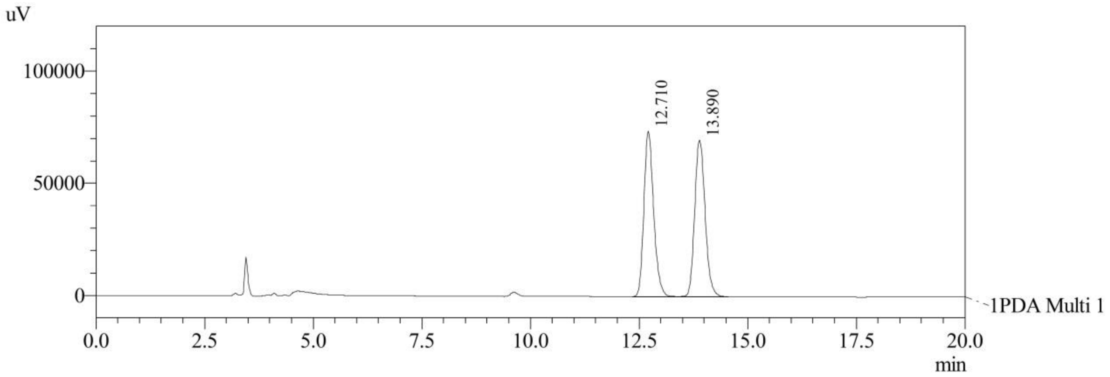

1 PDA Multi 1 / 210nm 4nm

PeakTable

PDA Ch1 210nm 4nm

Peak#	Ret. Time	Area	Height	Area %	Height %
1	12.710	1151077	73753	49.834	51.422
2	13.890	1158730	69674	50.166	48.578
Total		2309807	143427	100.000	100.000

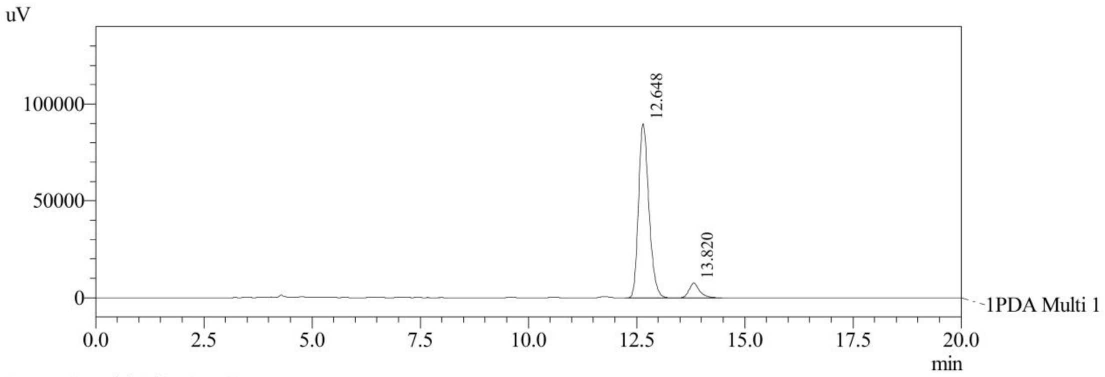

1 PDA Multi 1 / 254nm 4nm

PeakTable

PDA Ch1 254nm 4nm

Peak#	Ret. Time	Area	Height	Area %	Height %
1	12.648	1502972	89959	92.099	92.109
2	13.820	128928	7707	7.901	7.891
Total		1631900	97666	100.000	100.000

(S)-3-(benzylthio)-1-(3-fluorophenyl)butan-1-one (8le)

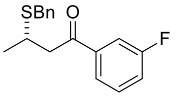

8le

The title compound was prepared according to the general procedure C and purified by flash column chromatography (20:1 hexanes : EtOAc) to afford **8le** (27 mg, 95%) as a colorless oil. Analytical data: IR (KBr, cm^{-1}) 2963, 2924, 1684, 1589, 1495, 1453, 1253, 1166, 896, 788, 707; ^{1}H NMR (400 MHz, $CDCl_3$) δ 7.69 – 7.64 (m, 1H), 7.62 – 7.55 (m, 1H), 7.44 (td, *J* = 8.0, 5.5 Hz, 1H), 7.40 – 7.22 (m, 5H), 3.90 – 3.76 (m, 2H), 3.46 – 3.32 (m, 1H), 3.24 (dd, *J* = 16.8, 5.2 Hz, 1H), 3.04 (dd, *J* = 16.8, 8.4 Hz, 1H), 1.36 (d, *J* = 6.7 Hz, 3H); ^{19}F NMR (376 MHz, $CDCl_3$) δ -111.65 (s, 1F); ^{13}C NMR (100 MHz, $CDCl_3$) δ 196.77 (s), 162.82 (d, J = 246 Hz), 138.92 (d, J = 6.2 Hz), 138.92 (d, J = 7.0 Hz), 138.29 (s), 128.80 (s), 128.56 (s), 127.05 (s), 123.80 (d, J = 3.0 Hz), 120.18 (d, J = 22.0 Hz), 114.79 (d, J = 22.0 Hz), 46.22 (s), 35.64 (s), 35.32 (s), 21.50 (s). HPLC (AD-H, 2.5% EtOH in hexanes, 1.0 mL/min, 210 nm): t_{major} = 9.2 min, t_{minor} = 10.6 min, 82% ee; $^{25}[\alpha]_D$ = -16.6 ° (c = 1.0 in $CHCl_3$); HRMS (ESI+) Calcd for $C_{17}H_{17}OFNaS^+$ $(M+Na)^+$: 311.0882, Found: 311.0877.

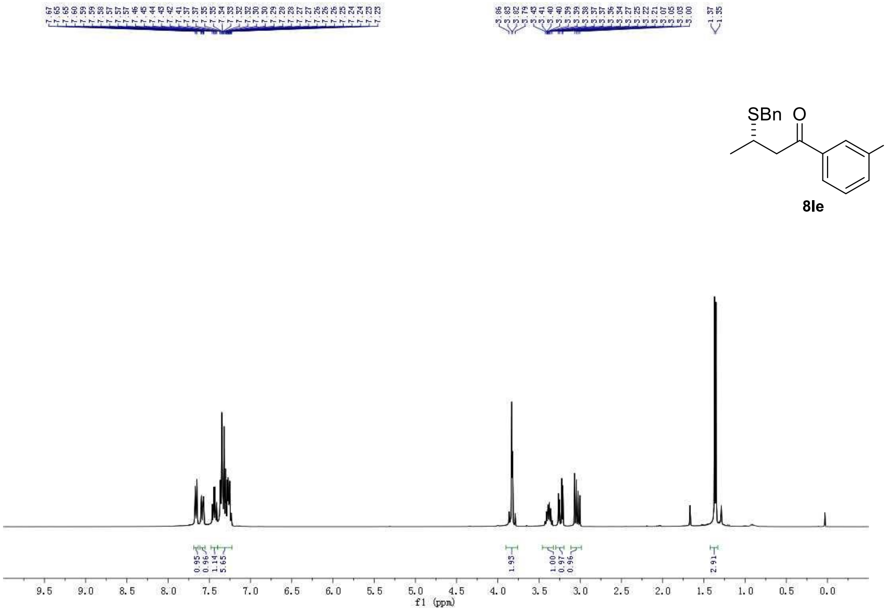

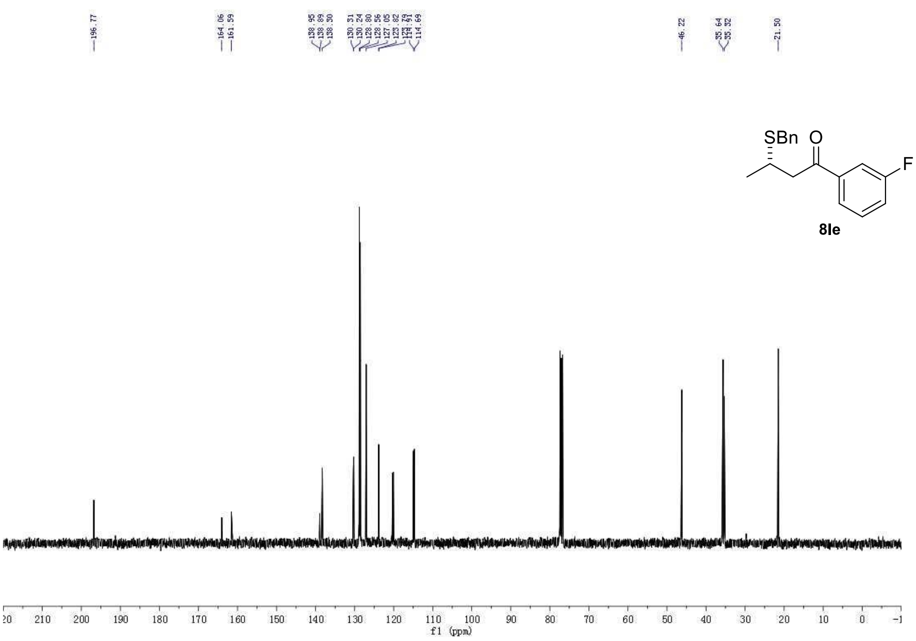

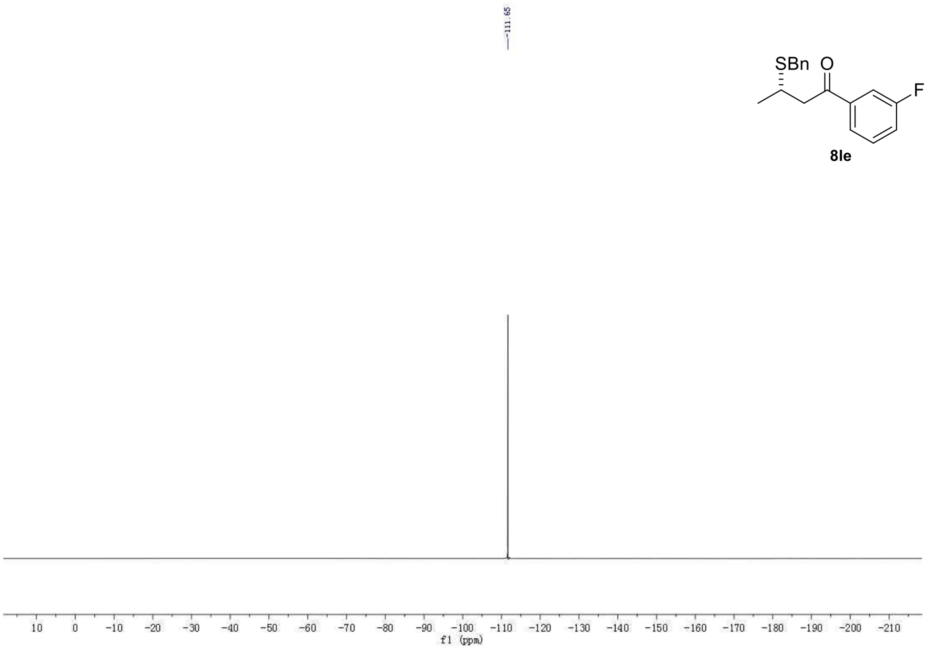

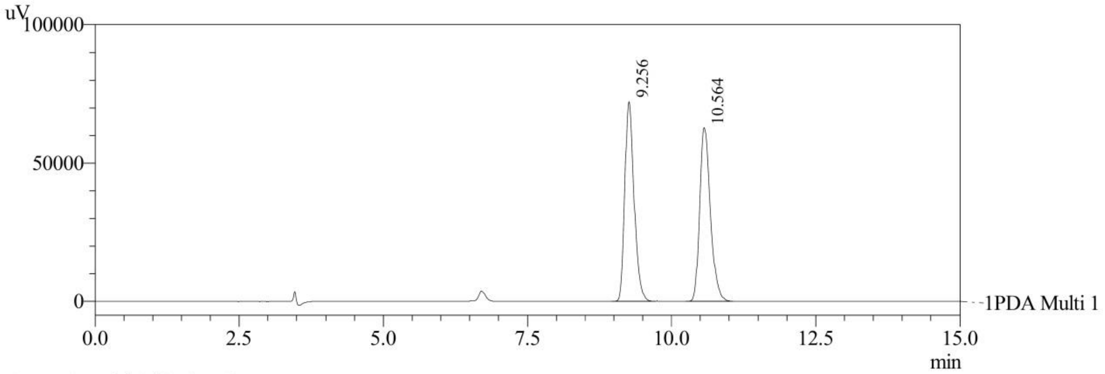

1 PDA Multi 1 / 254nm 4nm

PeakTable

PDA Ch1 254nm 4nm

Peak#	Ret. Time	Area	Height	Area %	Height %
1	9.256	809336	72214	49.978	53.500
2	10.564	810055	62764	50.022	46.500
Total		1619391	134978	100.000	100.000

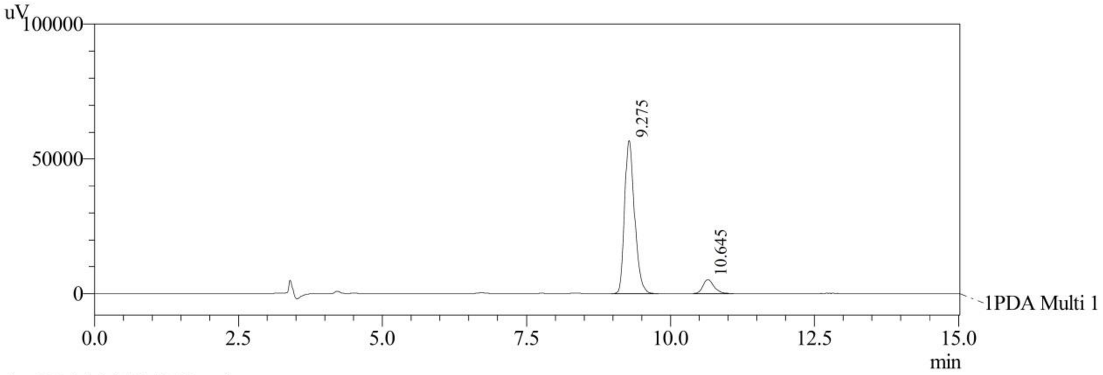

1 PDA Multi 1 / 254nm 4nm

PeakTable

PDA Ch1 254nm 4nm

Peak#	Ret. Time	Area	Height	Area %	Height %
1	9.275	685430	56941	90.861	91.746
2	10.645	68939	5123	9.139	8.254
Total		754369	62064	100.000	100.000

(S)-3-(benzylthio)-1-(4-fluorophenyl)butan-1-one (8lf)

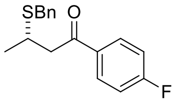

8lf

The title compound was prepared according to the general procedure C and purified by flash column chromatography (20:1 hexanes : EtOAc) to afford **8lf** (28 mg, 99%) as a colorless oil. Analytical data: IR (KBr, cm^{-1}) 2966, 2923, 1684, 1598, 1506, 1453, 1409, 1231, 1156, 988, 833, 769; ^{1}H NMR (400 MHz, $CDCl_3$) δ 7.97 – 7.87 (m, 2H), 7.40 – 7.21 (m, 5H), 7.17 – 7.09 (m, 2H), 3.90 – 3.77 (m, 2H), 3.46 – 3.32 (m, 1H), 3.24 (dd, *J* = 16.6, 5.2 Hz, 1H), 3.03 (dd, *J* = 16.6, 8.5 Hz, 1H), 1.36 (d, *J* = 6.7 Hz, 3H); ^{19}F NMR (376 MHz, $CDCl_3$) δ -104.96 (s, 1F); ^{13}C NMR (100 MHz, $CDCl_3$) δ 196.42 (s), 165.76 (d, *J* = 253.0 Hz), 138.34 (s), 133.29 (d, *J* = 2.8 Hz), 130.72 (d, *J* = 9.3 Hz), 128.82 (s), 128.56 (s), 127.03 (s), 115.69 (d, *J* = 22.0 Hz), 45.98 (s), 35.64 (s), 35.43 (s), 21.52 (s). HPLC (AD-H, 1% EtOH in hexanes, 1.0 mL/min, 210 nm): t_{major} = 13.5 min, t_{minor} = 15.0 min, 88% ee; $^{25}[\alpha]_D$ = -26.4 ° (c = 1.0 in $CHCl_3$); HRMS (ESI+) Calcd for $C_{17}H_{17}OFNaS^+$ $(M+Na)^+$: 311.0882, Found: 311.0878.

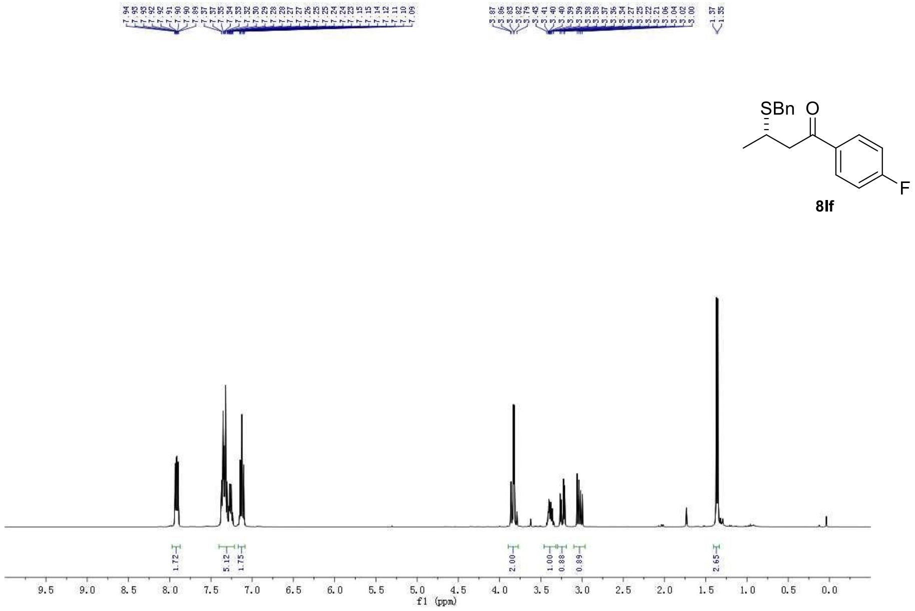

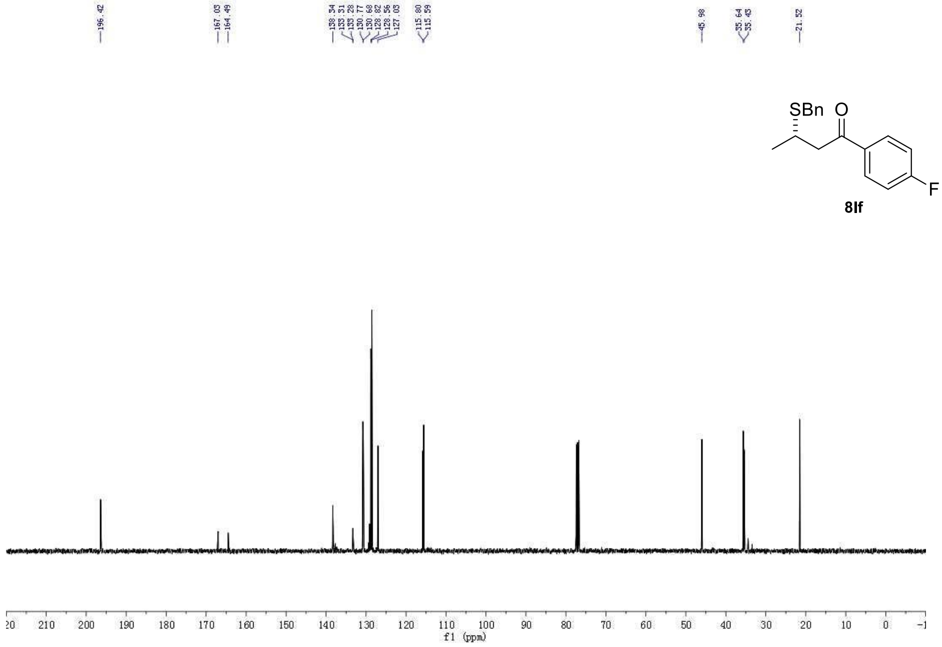

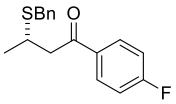

8lf

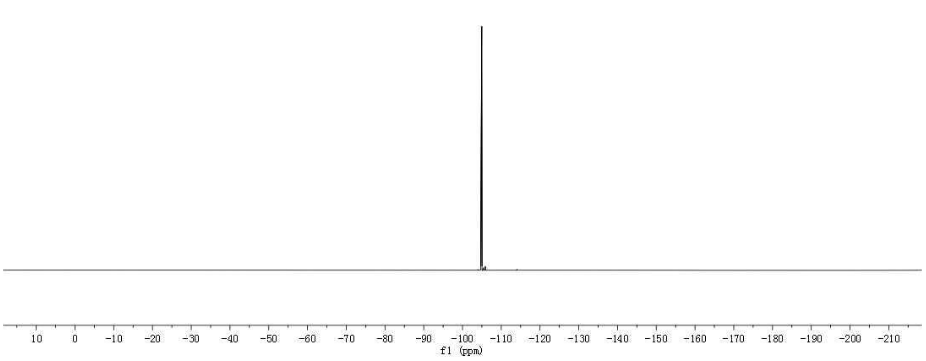

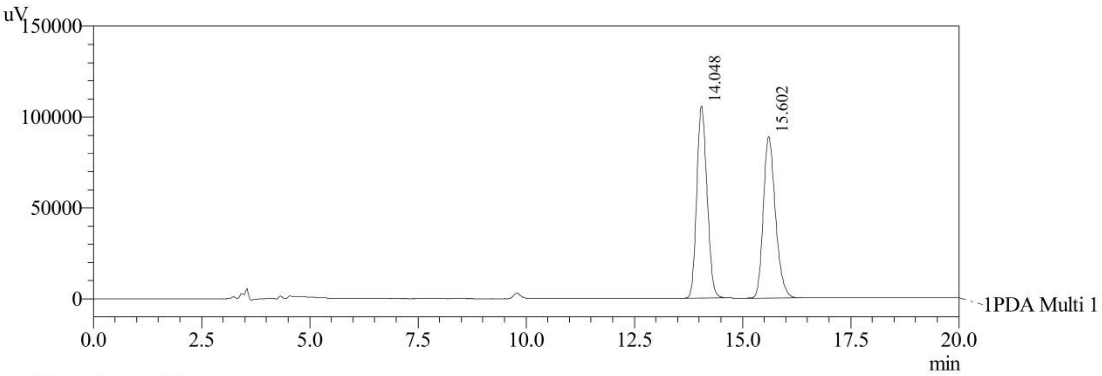

1 PDA Multi 1 / 210nm 4nm

PeakTable

PDA Ch1 210nm 4nm

Peak#	Ret. Time	Area	Height	Area %	Height %
1	14.048	1746230	105706	50.058	54.343
2	15.602	1742210	88811	49.942	45.657
Total		3488440	194516	100.000	100.000

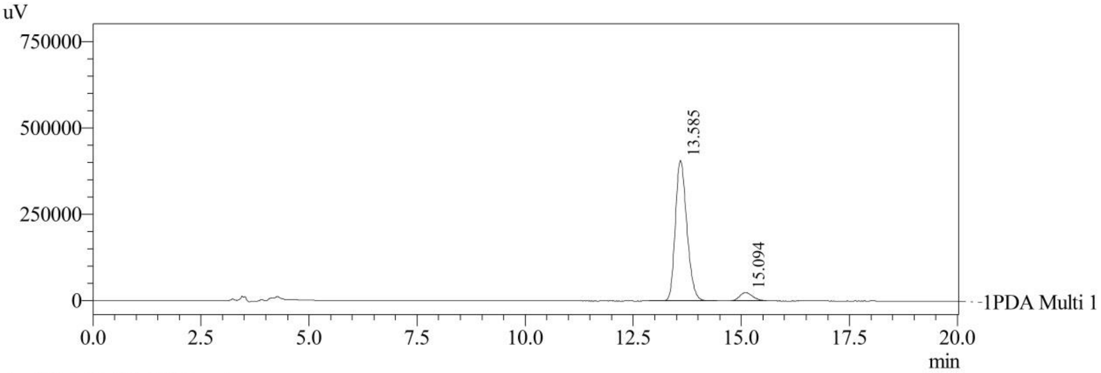

1 PDA Multi 1 / 210nm 4nm

PeakTable

PDA Ch1 210nm 4nm

Peak#	Ret. Time	Area	Height	Area %	Height %
1	13.585	7544315	405806	93.925	94.466
2	15.094	487945	23775	6.075	5.534
Total		8032260	429581	100.000	100.000

(S)-3-(benzylthio)-1-(p-tolyl)butan-1-one (8lg)

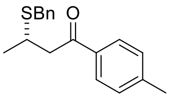

8lg

The title compound was prepared according to the general procedure C and purified by flash column chromatography (20:1 hexanes : EtOAc) to afford **8lg** (25 mg, 89%) as a colorless oil. Analytical data: IR (KBr, cm^{-1}) 2962, 2921, 1672, 1604, 1507, 1452, 1179, 807, 767; ^{1}H NMR (400 MHz, $CDCl_3$) δ 7.81 (d, J = 8.2 Hz, 2H), 7.38 – 7.22 (m, 7H), 3.90 – 3.76 (m, 2H), 3.46 – 3.34 (m, 1H), 3.25 (dd, J = 16.6, 5.0 Hz, 1H), 3.05 (dd, J = 16.6, 8.7 Hz, 1H), 2.43 (s, 3H), 1.34 (d, J = 6.7 Hz, 3H); ^{13}C NMR (100 MHz, $CDCl_3$) δ 197.71 (s), 144.01 (s), 138.37 (s), 134.40 (s), 129.28 (s), 128.81 (s), 128.53 (s), 128.20 (s), 126.97 (s), 45.92 (s), 35.63 (s), 35.55 (s), 21.64 (s), 21.47 (s). HPLC (AD-H, 2.5% EtOH in hexanes, 1.0 mL/min, 210 nm): t_{major} = 10.2 min, t_{minor} = 11.1 min, 92% ee; $^{25}[\alpha]_D$ = -38.9 ° (c = 1.0 in $CHCl_3$); HRMS (ESI+) Calcd for $C_{18}H_{20}ONaS^+$ $(M+Na)^+$: 307.1133, Found: 307.1127.

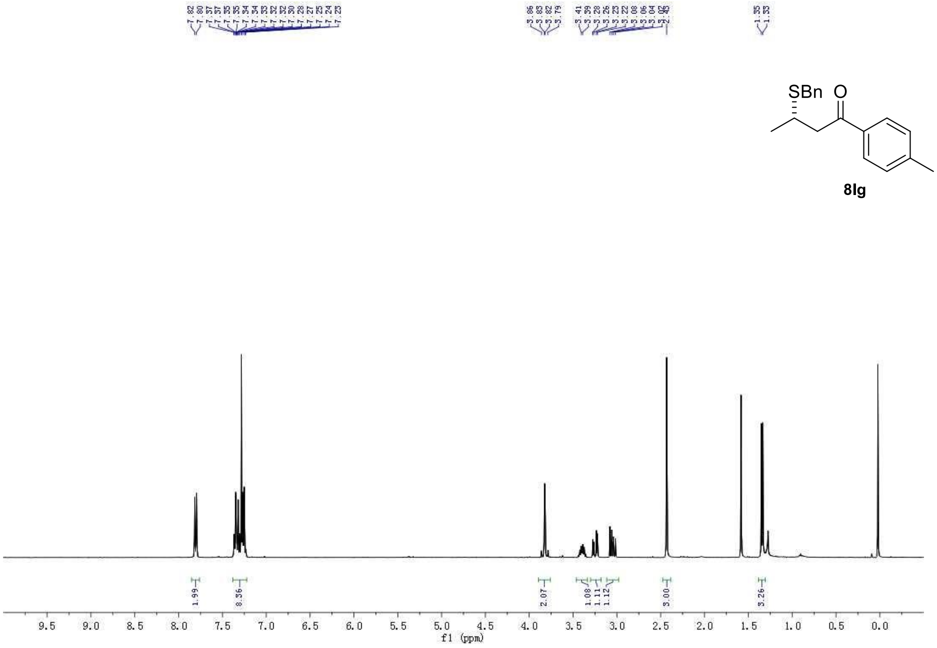

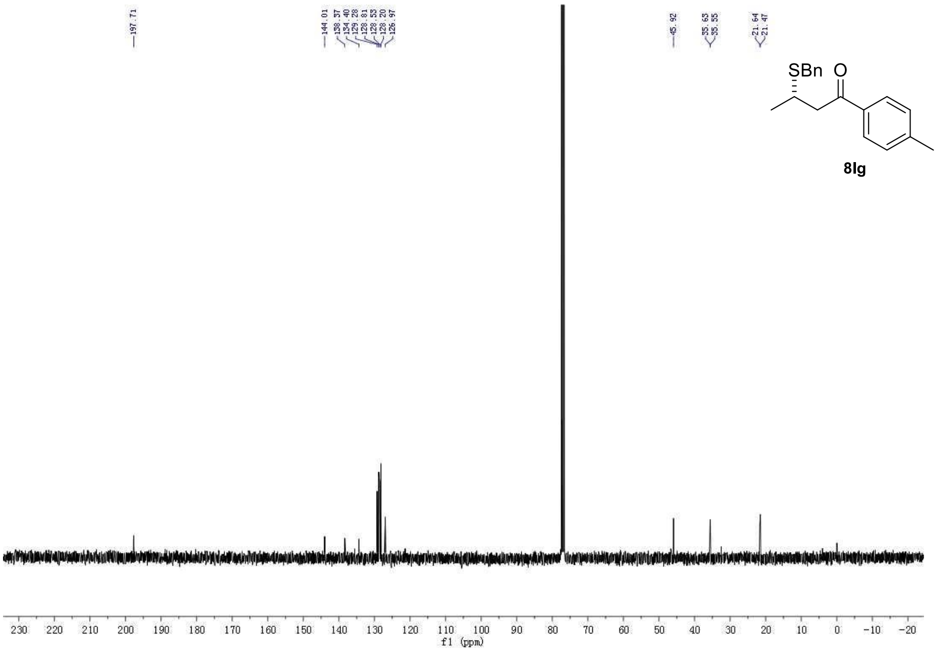

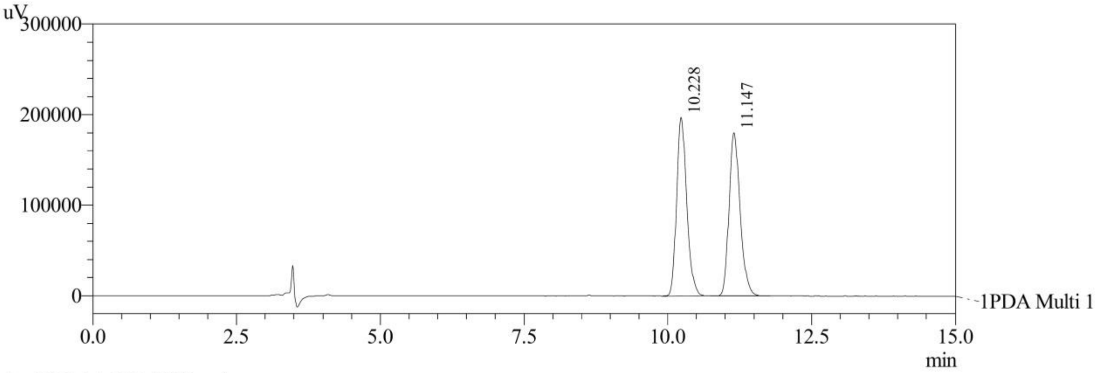

1 PDA Multi 1 / 210nm 4nm

PeakTable

PDA Ch1 210nm 4nm

Peak#	Ret. Time	Area	Height	Area %	Height %
1	10.228	2431390	197222	49.967	52.243
2	11.147	2434599	180288	50.033	47.757
Total		4865989	377510	100.000	100.000

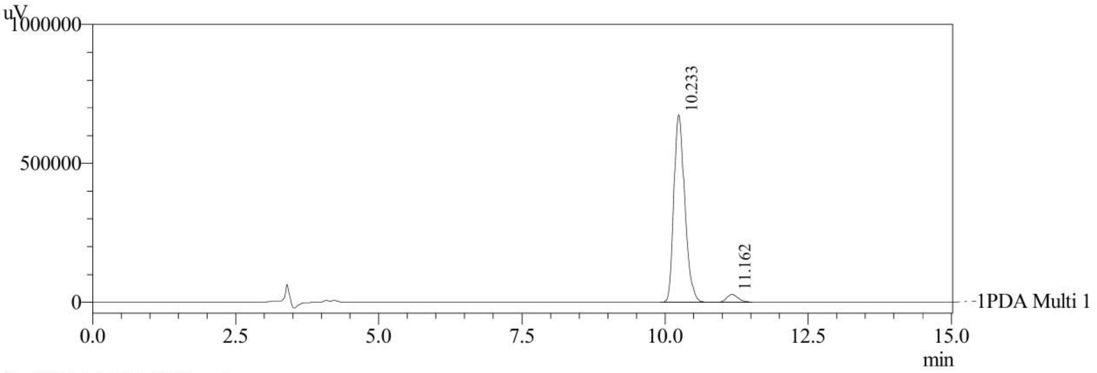

1 PDA Multi 1 / 210nm 4nm

PeakTable

PDA Ch1 210nm 4nm

Peak#	Ret. Time	Area	Height	Area %	Height %
1	10.233	9012214	676251	95.846	95.973
2	11.162	390617	28375	4.154	4.027
Total		9402830	704627	100.000	100.000

(S)-3-(benzylthio)-1-(4-methoxyphenyl)butan-1-one (8lh)

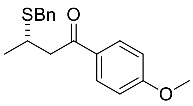

8lh

The title compound was prepared according to the general procedure C and purified by flash column chromatography (20:1 hexanes : EtOAc) to afford **8lh** (27 mg, 90%) as a colorless oil. Analytical data: IR (KBr, cm^{-1}) 2964, 2924, 1672, 1601, 1510, 1453, 1260, 1170, 1028, 831, 705; ^{1}H NMR (400 MHz, $CDCl_3$) δ 7.95 – 7.85 (m, 2H), 7.40 – 7.21 (m, 5H), 6.97 – 6.89 (m, 2H), 3.89 (s, 3H), 3.87 – 3.77 (m, 2H), 3.52 – 3.33 (m, 1H), 3.23 (dd, J = 16.4, 5.0 Hz, 1H), 3.02 (dd, J = 16.4, 8.7 Hz, 1H), 1.35 (d, J = 6.7 Hz, 3H); ^{13}C NMR (100 MHz, $CDCl_3$) δ 196.59 (s), 163.55 (s), 138.40 (s), 130.38 (s), 129.99 (s), 128.82 (s), 128.53 (s), 126.97 (s), 113.74 (s), 55.47 (s), 45.68 (s), 35.68 (s), 35.63 (s), 21.50 (s). HPLC (OD-H, 2.5% EtOH in hexanes, 1.0 mL/min, 210 nm): t_{major} = 11.5 min, t_{minor} = 12.6 min, 91% ee; $^{25}[\alpha]_D$ = -35.0 ^{o} (c = 1.0 in $CHCl_3$); HRMS (ESI+) Calcd for $C_{18}H_{20}O_2NaS^+$ $(M+Na)^+$: 323.1082, Found: 323.1076.

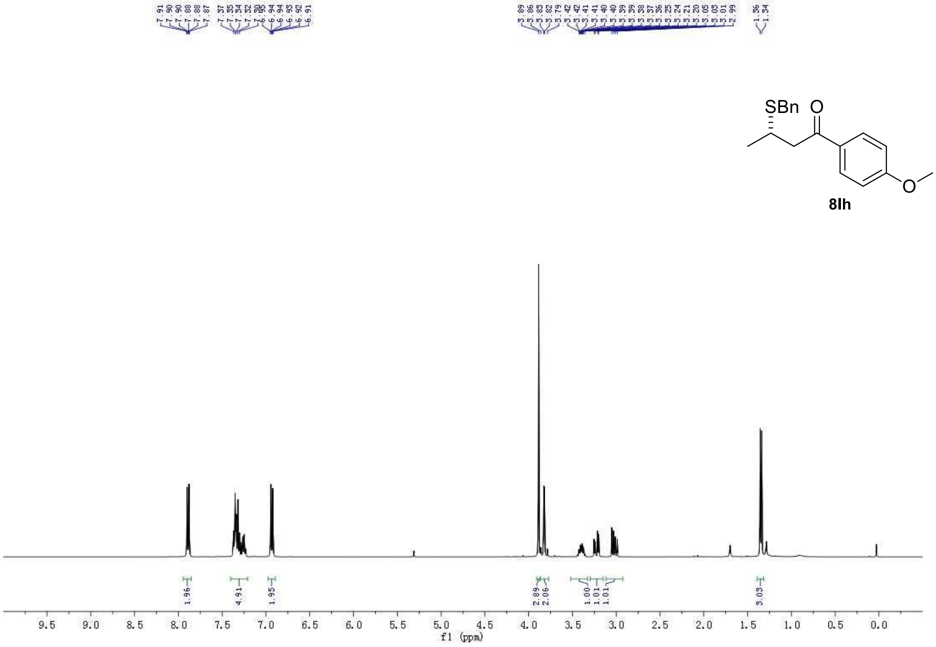

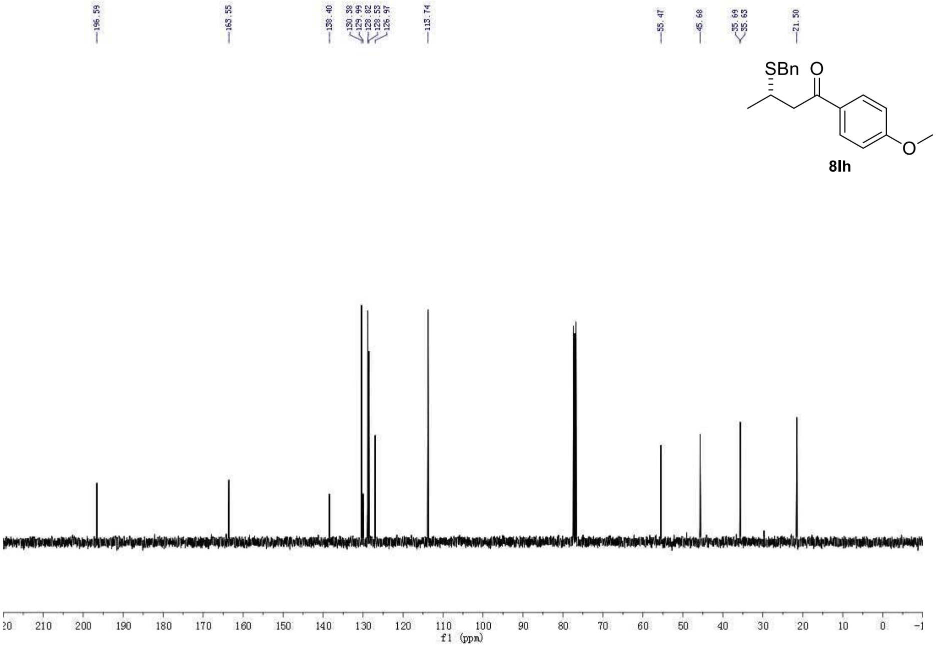

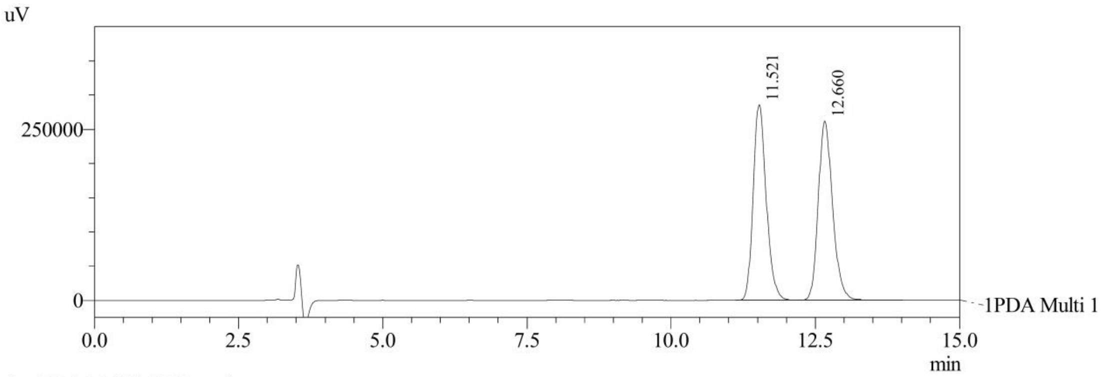

1 PDA Multi 1 / 210nm 4nm

PeakTable

PDA Ch1 210nm 4nm

Peak#	Ret. Time	Area	Height	Area %	Height %
1	11.521	4512400	286001	49.770	52.192
2	12.660	4554110	261980	50.230	47.808
Total		9066509	547982	100.000	100.000

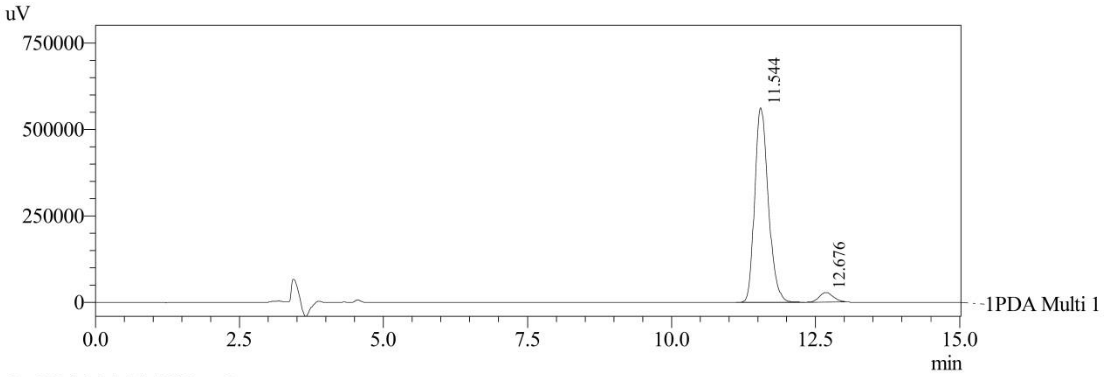

1 PDA Multi 1 / 210nm 4nm

PeakTable

PDA Ch1 210nm 4nm

Peak#	Ret. Time	Area	Height	Area %	Height %
1	11.544	9256880	561811	95.218	95.308
2	12.676	464938	27657	4.782	4.692
Total		9721818	589468	100.000	100.000

(R)-3-(benzylthio)-4-methyl-1-phenylpentan-1-one (8li)

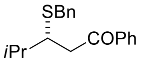

8li

The title compound was prepared according to the general procedure C and purified by flash column chromatography (20:1 hexanes : EtOAc) to afford **8li** (29 mg, 99%) as a colorless oil. Analytical data: IR (KBr, cm^{-1}t) 2959, 2927, 1684, 1597, 1494, 1148, 752; ^{1}H NMR (400 MHz, $CDCl_3$) δ 8.01 – 7.91 (m, 2H), 7.63 – 7.55 (m, 1H), 7.53 – 7.45 (m, 2H), 7.37 – 7.18 (m, 5H), 3.85 – 3.72 (m, 2H), 3.43 – 3.27 (m, 2H), 3.21 – 3.10 (m, 1H), 2.00 – 1.89 (m, 1H), 0.98 (dd, *J* = 7.9, 6.8 Hz, 6H); ^{13}C NMR (100 MHz, $CDCl_3$) δ 198.78 (s), 138.62 (s), 137.20 (s), 133.07 (s), 129.03 (s), 128.61 (s), 128.38 (s), 128.15 (s), 126.91 (s), 47.77 (s), 42.81 (s), 37.28 (s), 32.40 (s), 19.85 (s), 18.66 (s). HPLC (AD-H, 2.5% EtOH in hexanes, 1.0 mL/min, 210 nm): t_{major} = 5.4 min, t_{minor} = 6.3 min, 94% ee; $^{25}[\alpha]_D$ = -58.8 ° (c = 1.0 in $CHCl_3$); HRMS (ESI+) Calcd for $C_{19}H_{22}ONaS^+$ $(M+Na)^+$: 321.1289, Found: 321.1281.

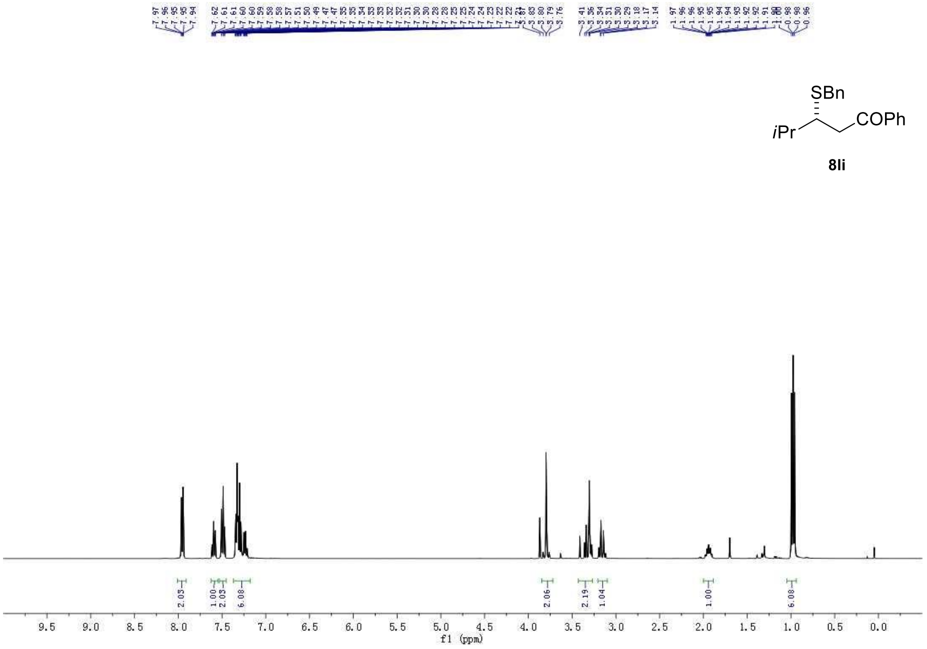

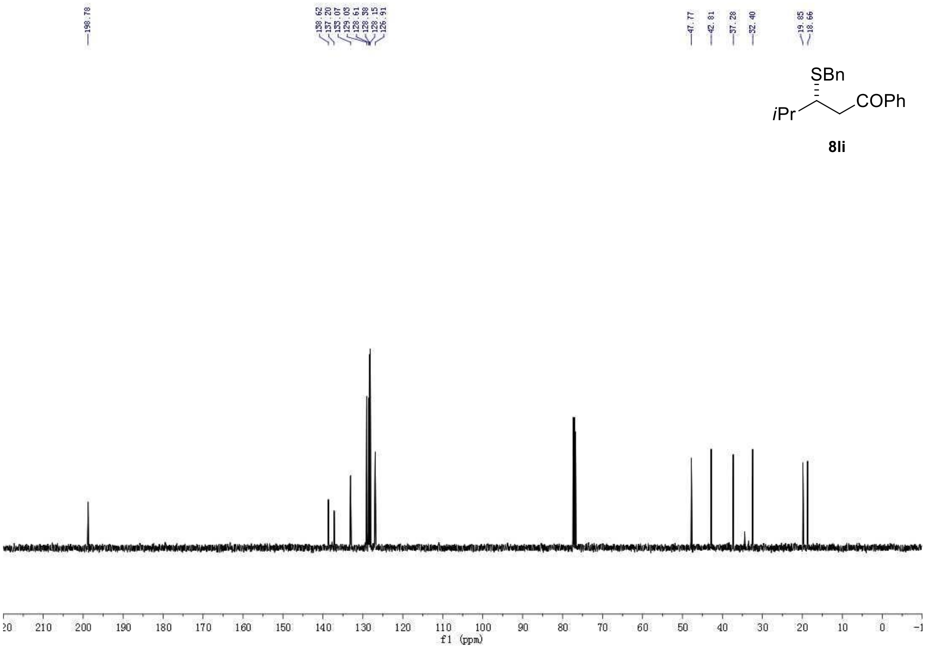

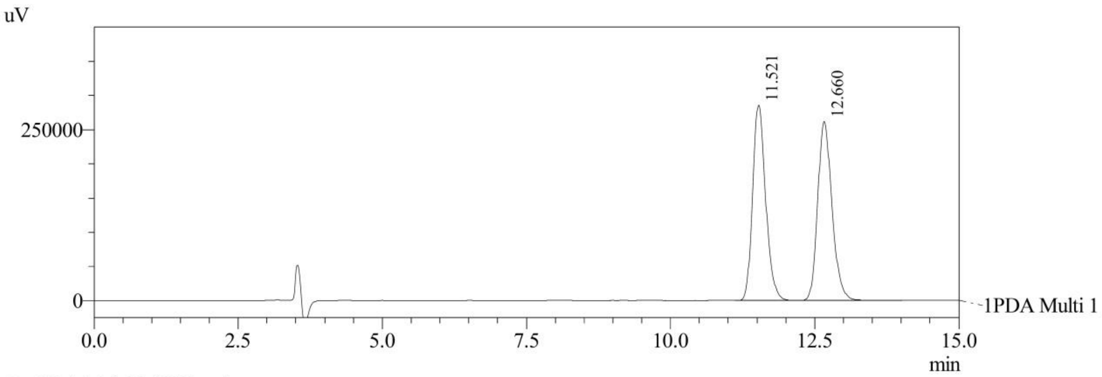

1 PDA Multi 1 / 210nm 4nm

PeakTable

PDA Ch1 210nm 4nm

Peak#	Ret. Time	Area	Height	Area %	Height %
1	11.521	4512400	286001	49.770	52.192
2	12.660	4554110	261980	50.230	47.808
Total		9066509	547982	100.000	100.000

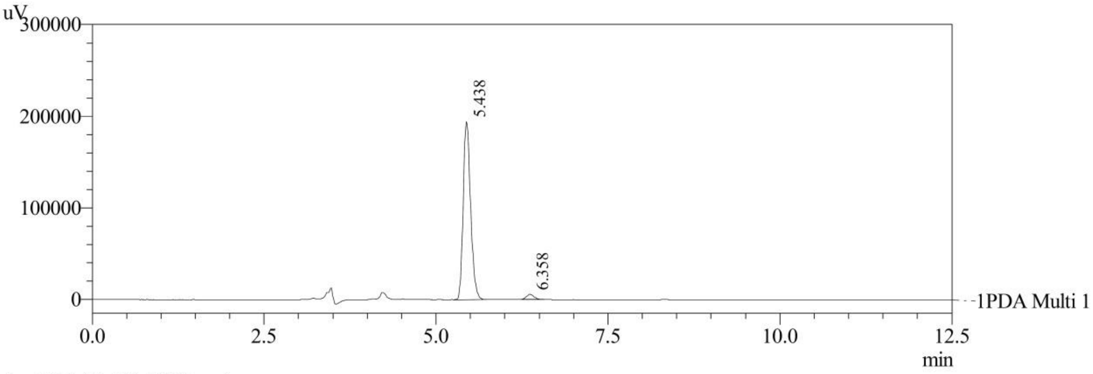

1 PDA Multi 1 / 210nm 4nm

PeakTable

PDA Ch1 210nm 4nm

Peak#	Ret. Time	Area	Height	Area %	Height %
1	5.438	1451995	194500	97.059	97.267
2	6.358	43996	5465	2.941	2.733
Total		1495991	199965	100.000	100.000

(R)-3-(benzylthio)-3-cyclohexyl-1-phenylpropan-1-one (8lj)

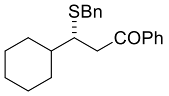

8lj

The title compound was prepared according to the general procedure C and purified by flash column chromatography (20:1 hexanes : EtOAc) to afford **8lj** (33 mg, 99%) as a colorless oil. Analytical data: IR (KBr, cm^{-1}) 2925, 2852, 1684, 1598, 1448, 1180, 754; ^{1}H NMR (400 MHz, $CDCl_3$) δ 7.99 – 7.91 (m, 2H), 7.59 (t, *J* = 7.4 Hz, 1H), 7.48 (t, *J* = 7.6 Hz, 2H), 7.37 – 7.18 (m, 5H), 3.90 – 3.68 (m, 2H), 3.38 – 3.15 (m, 3H), 1.84 – 1.71 (m, 3H), 1.71 – 1.61 (m, 2H), 1.54 (td, *J* = 11.1, 2.9 Hz, 1H), 1.35 – 1.04 (m, 5H); ^{13}C NMR (100 MHz, $CDCl_3$) δ 198.79 (s), 138.62 (s), 137.17 (s), 133.06 (s), 129.03 (s), 128.60 (s), 128.38 (s), 128.15 (s), 126.90 (s), 47.09 (s), 42.81 (s), 42.65 (s), 37.38 (s), 30.23 (s), 29.39 (s), 26.42 (s), 26.39 (s), 26.35 (s). HPLC (AD-H, 1% EtOH in hexanes, 1.0 mL/min, 210 nm): t_{major} = 7.8 min, t_{minor} = 10.0 min, 91% ee; $^{25}[\alpha]_D$ = -29.3 ° (c = 1.0 in $CHCl_3$); HRMS (ESI+) Calcd for $C_{22}H_{26}ONaS^+$ $(M+Na)^+$: 361.1602, Found: 361.1596.

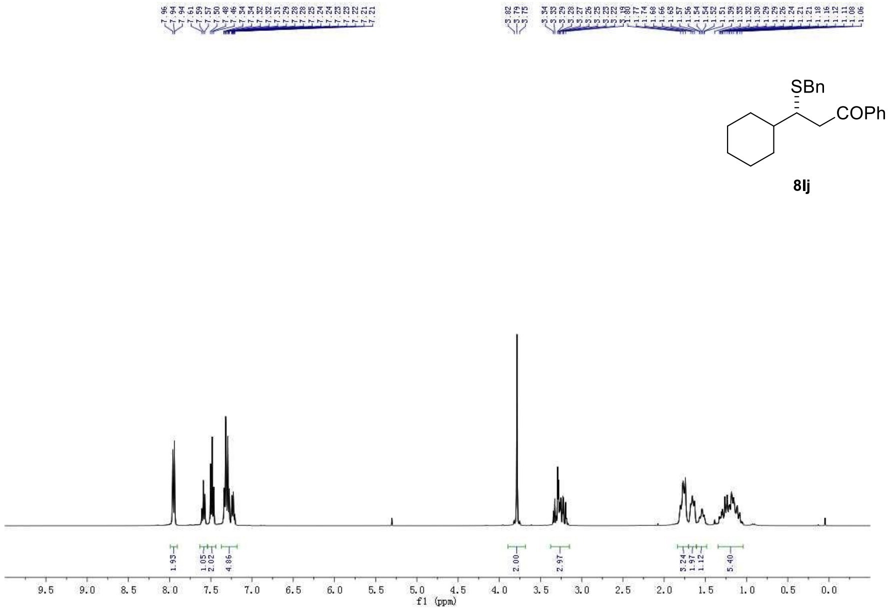

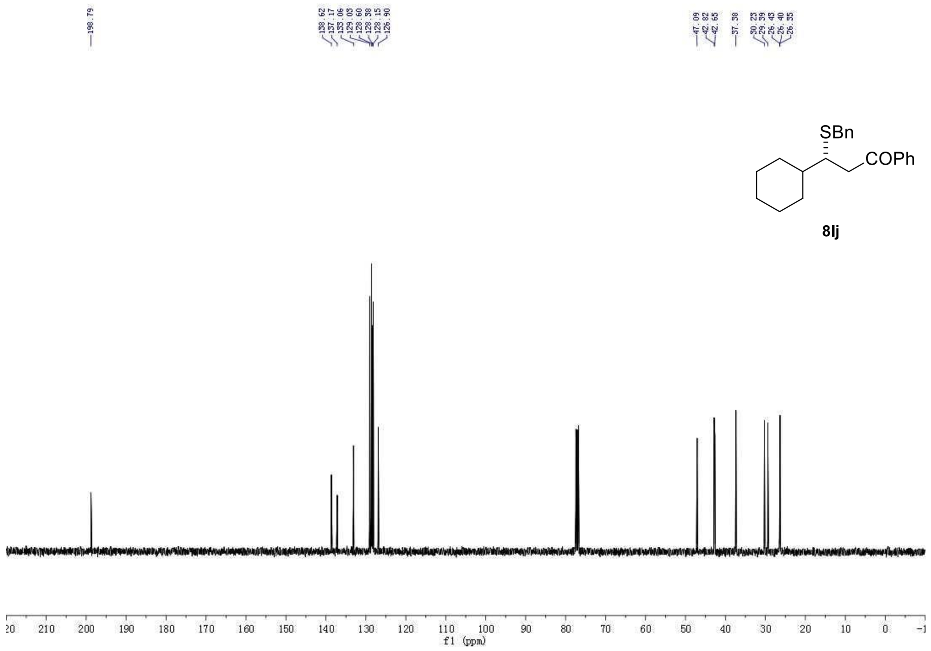

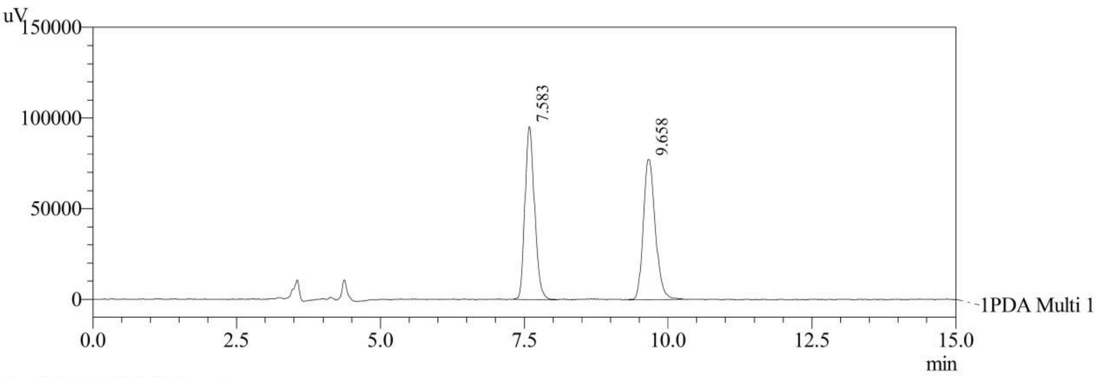

1 PDA Multi 1 / 210nm 4nm

PeakTable

PDA Ch1 210nm 4nm

Peak#	Ret. Time	Area	Height	Area %	Height %
1	7.583	1119828	95384	50.016	55.160
2	9.658	1119107	77540	49.984	44.840
Total		2238934	172923	100.000	100.000

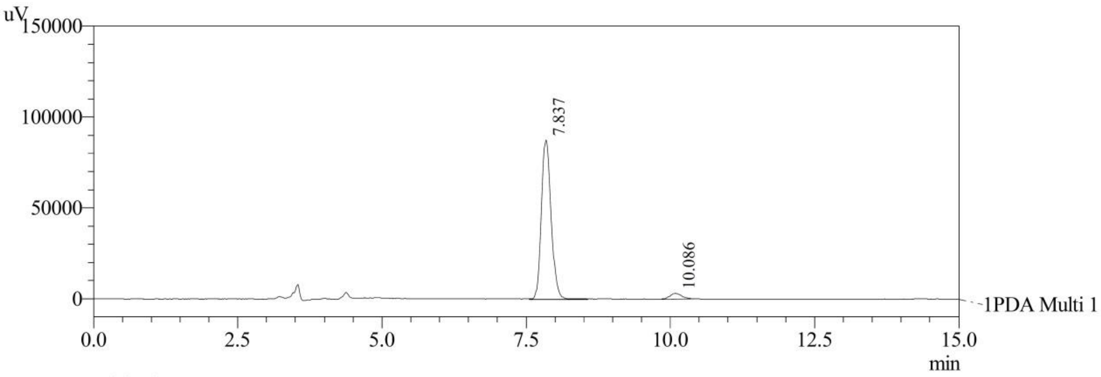

1 PDA Multi 1 / 210nm 4nm

PeakTable

PDA Ch1 210nm 4nm

Peak#	Ret. Time	Area	Height	Area %	Height %
1	7.837	1027199	87537	95.463	96.416
2	10.086	48817	3254	4.537	3.584
Total		1076015	90791	100.000	100.000

(S)-3-(benzylthio)-1-phenyloctan-1-one (8lk)

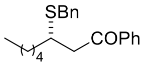

8lk

The title compound was prepared according to the general procedure C and purified by flash column chromatography (20:1 hexanes : EtOAc) to afford **8lk** (32 mg, 99%) as a colorless oil. Analytical data: IR (KBr, cm^{-1}) 2963, 2924, 1717, 1616, 1488, 1447, 1361, 1159, 944, 788; ^{1}H NMR (400 MHz, $CDCl_3$) δ 7.95 – 7.88 (m, 2H), 7.63 – 7.55 (m, 1H), 7.53 – 7.44 (m, 2H), 7.38 – 7.20 (m, 5H), 3.91 – 3.68 (m, 2H), 3.38 – 3.23 (m, 2H), 3.23 – 3.08 (m, 1H), 1.68 – 1.52 (m, 2H), 1.51 – 1.17 (m, 6H), 0.89 (t, *J* = 7.1 Hz, 3H); ^{13}C NMR (100 MHz, $CDCl_3$) δ 198.48 (s), 138.61 (s), 137.03 (s), 133.10 (s), 128.93 (s), 128.59 (s), 128.46 (s), 128.10 (s), 126.94 (s), 45.09 (s), 40.97 (s), 35.93 (s), 35.12 (s), 31.53 (s), 26.37 (s), 22.54 (s), 14.04 (s). HPLC (AD-H, 2.5% EtOH in hexanes, 1.0 mL/min, 210 nm): t_{major} = 5.8 min, t_{minor} = 7.0 min, 87% ee; $^{25}[\alpha]_D$ = -34.6 ^{o} (c = 1.0 in $CHCl_3$); HRMS (ESI+) Calcd for $C_{21}H_{26}ONaS^{+}$ $(M+Na)^{+}$: 349.1602, Found: 349.1594.

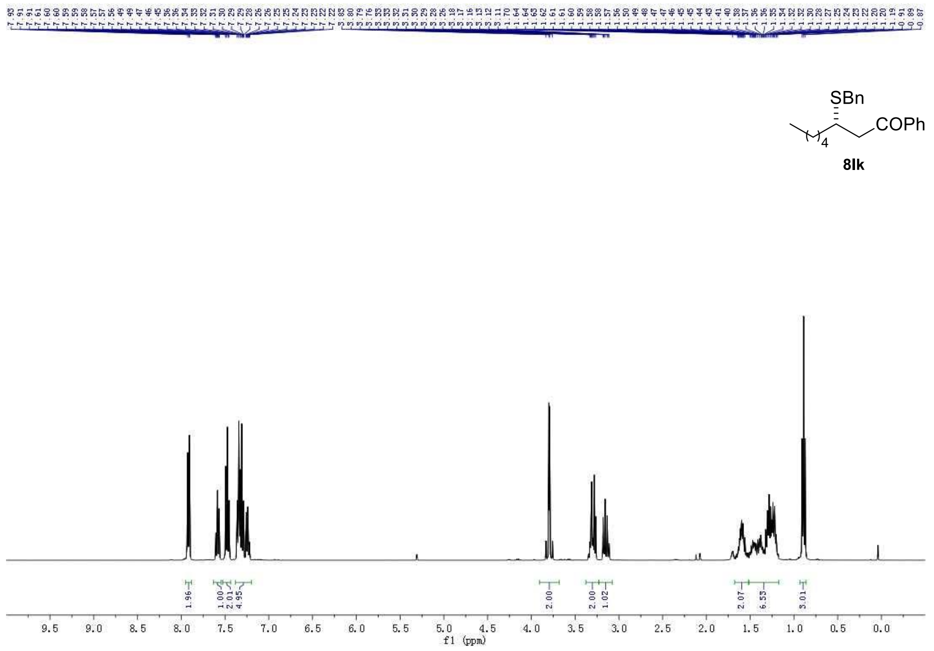

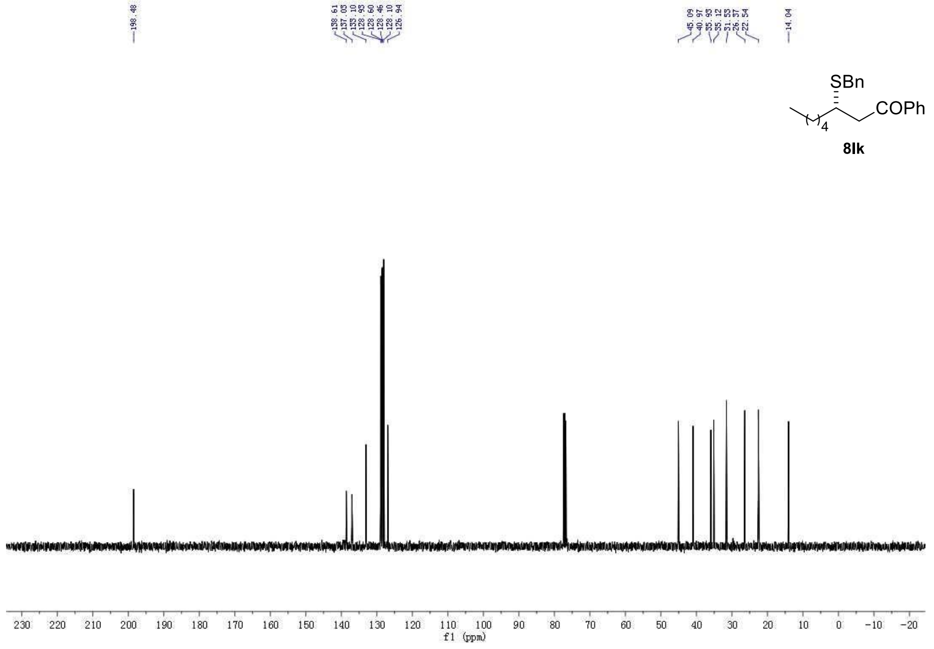

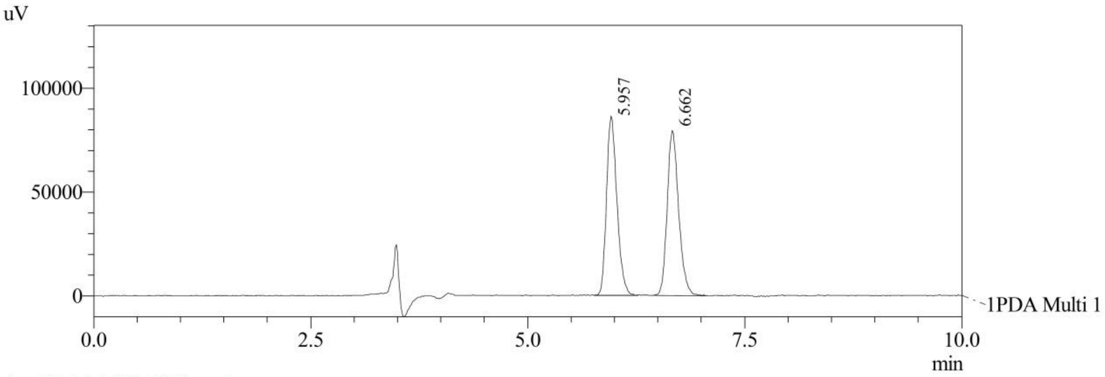

1 PDA Multi 1 / 210nm 4nm

PeakTable

PDA Ch1 210nm 4nm

Peak#	Ret. Time	Area	Height	Area %	Height %
1	5.957	717608	86202	49.522	52.088
2	6.662	731469	79290	50.478	47.912
Total		1449077	165492	100.000	100.000

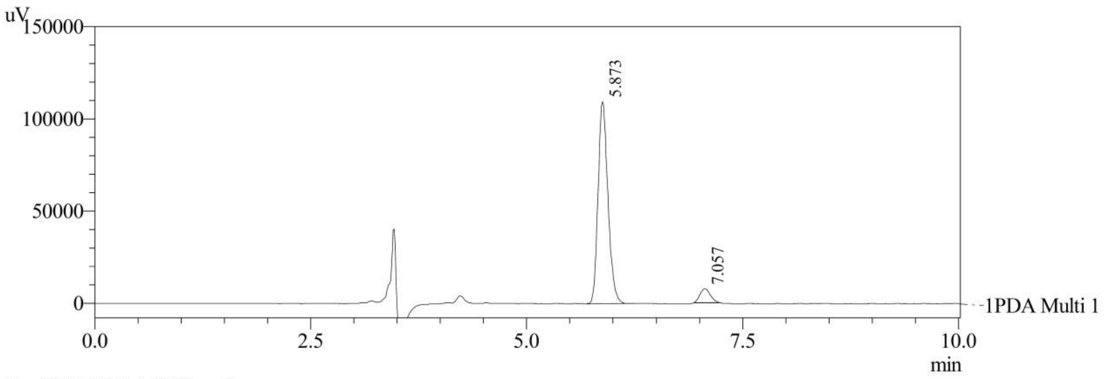

1 PDA Multi 1 / 210nm 4nm

PeakTable

PDA Ch1 210nm 4nm

Peak#	Ret. Time	Area	Height	Area %	Height %
1	5.873	874011	109723	93.220	93.525
2	7.057	63563	7597	6.780	6.475
Total		937574	117320	100.000	100.000

(R)-3,3,3-trifluoro-2-(phenethylthio)-2-phenylpropan-1-amine (11)

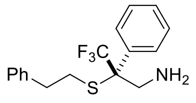

11

The title compound was prepared according to the reported procedure[7] and purified by flash column chromatography (4:1 hexanes : EtOAc) to afford **11** (65 mg, 99%) as a yellow solid. Analytical data: IR (KBr, cm^{-1}) 3063, 3028, 2926, 2853, 1496, 1454, 1245, 1149, 803, 752, 712, 697; ^{1}H NMR (400 MHz, $CDCl_3$) δ 7.61 (d, J = 7.9 Hz, 2H), 7.47 – 7.16 (m, 6H), 7.12 (d, J = 7.0 Hz, 2H), 3.39 (q, J = 14.7 Hz, 2H), 2.87 – 2.71 (m, 3H), 2.72 – 2.57 (m, 1H), 1.39 – 1.23 (brs, 2H); ^{19}F NMR (376 MHz, $CDCl_3$) δ -65.90 (s, 3F); ^{13}C NMR (100 MHz, $CDCl_3$) δ 139.76 (s), 134.04 (s), 128.77 (s), 128.49 (s), 128.44 (s), 128.34 (s), 127.49 (q, J = 283.0 Hz), 126.53 (s), 62.21 (q, J = 24.0 Hz), 46.12 (s), 35.15 (s), 31.20 (s). HPLC (AD-H, 1% EtOH in hexanes, 1.0 mL/min, 210 nm): t_{major} = 20.4 min, t_{minor} = 10.6 min, 92% ee; $^{25}[\alpha]_D$ = -12.2 ° (c = 1.0 in $CHCl_3$); HRMS (ESI+) Calcd for $C_{17}H_{19}NF_3S^+$ (M+H)$^+$: 326.1185, Found: 326.1182.

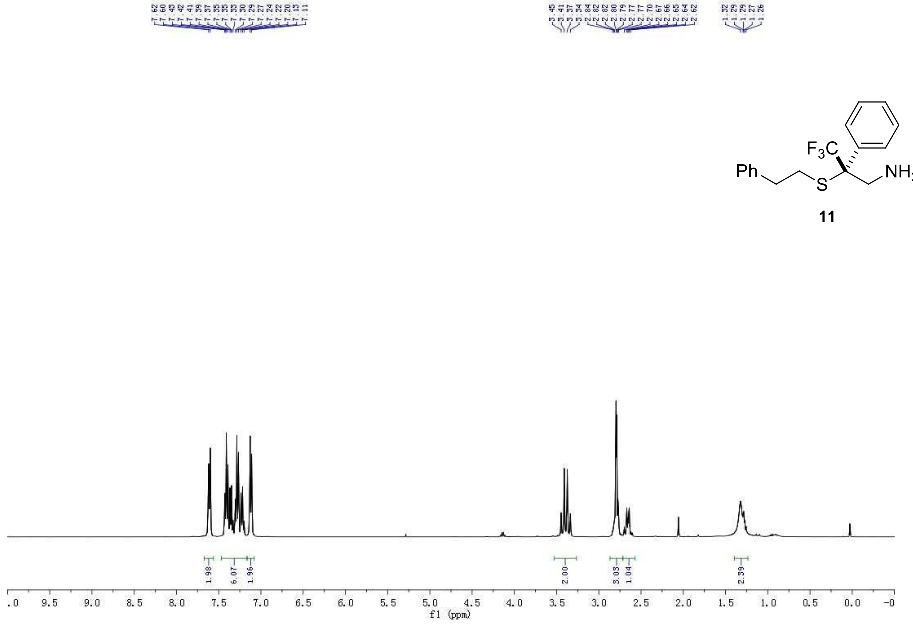

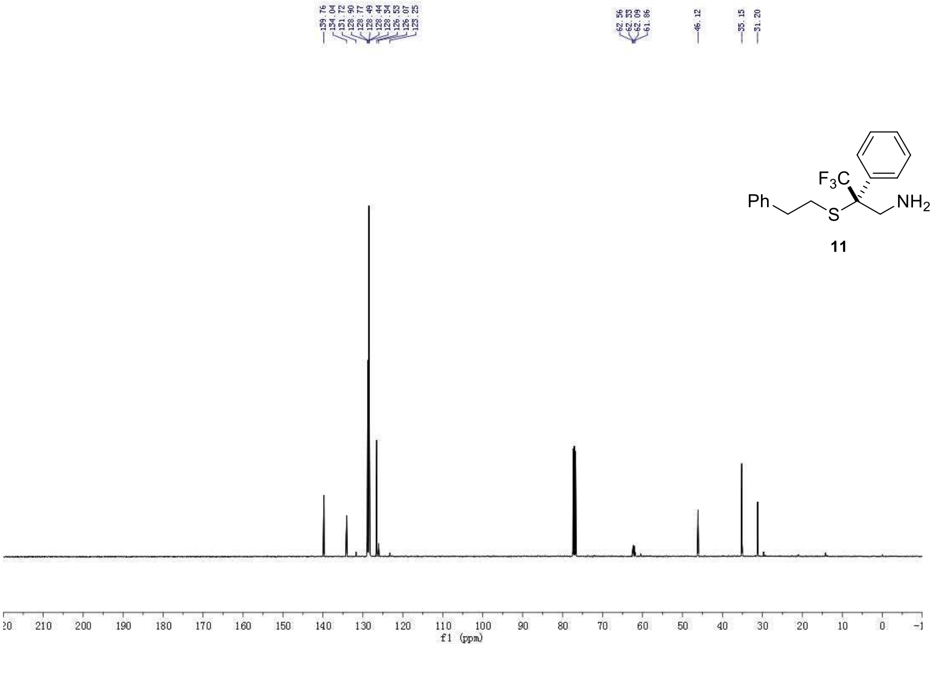

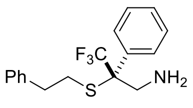

11

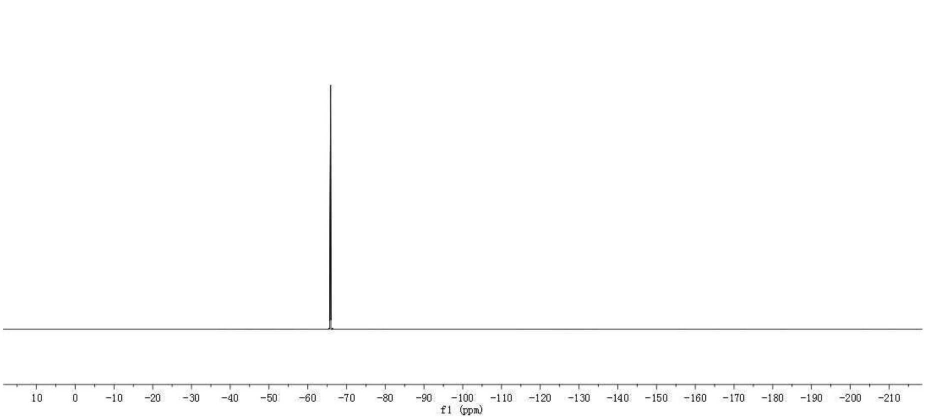

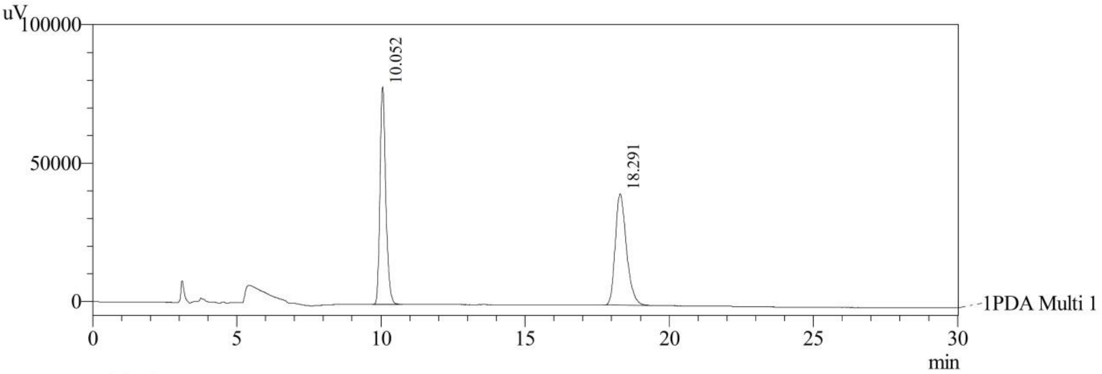

1 PDA Multi 1 / 210nm 4nm

PeakTable

PDA Ch1 210nm 4nm

Peak#	Ret. Time	Area	Height	Area %	Height %
1	10.052	1092695	78499	50.089	66.092
2	18.291	1088810	40273	49.911	33.908
Total		2181505	118772	100.000	100.000

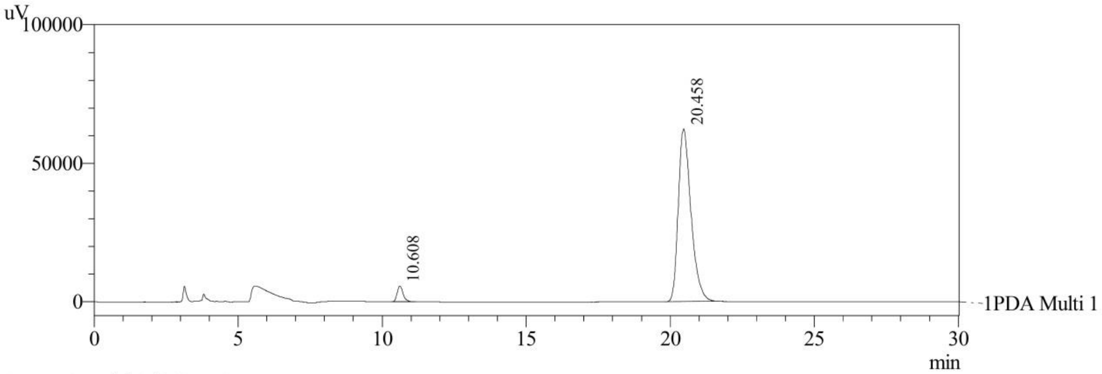

1 PDA Multi 1 / 210nm 4nm

PeakTable

PDA Ch1 210nm 4nm

Peak#	Ret. Time	Area	Height	Area %	Height %
1	10.608	81935	5729	4.100	8.420
2	20.458	1916566	62312	95.900	91.580
Total		1998501	68041	100.000	100.000

X-Ray single crystal structure of 3aa's derivative (11)

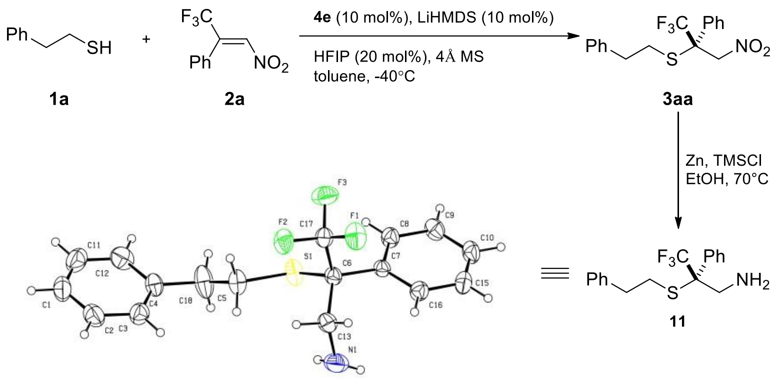

Supplementary References

(1) Gao, J. R.; Wu, H.; Xiang, B.; Yu, W. B.; Han, L.; Jia, Y. X. *J. Am. Chem. Soc.* **2013**, *135*, 2983.

(2) Bizet, V.; Pannecoucke, X.; Renaud, J. L.; Cahard, D. *Angew. Chem. Int. Ed.* **2012**, *51*, 6467.

(3) Chen, W.; Jing, Z.; Chin, K. F.; Qiao, B.; Zhao, Y.; Yan, L.; Tan, C.-H.; Jiang, Z. *Adv. Synth. Catal.* **2014**, *356*, 1292.

(4) White, J. D.; Shaw, S. *Chem. Sci.* **2014**, *5*, 2200.

(5) Ricci, P.; Carlone, A.; Bartoli, G.; Bosco, M.; Sambri, L.; Melchiorre, P. *Adv. Synth. Catal.***2008**, *350*, 49.

(6) (a) Liu, Y.; Sun, B.; Wang, B.; Wakem, M.; Deng, L. *J. Am. Chem. Soc.* **2009**, *131*, 418. (b) Abe, A. M.; Sauerland, S. J.; Koskinen, A. M. *J. Org. Chem.* **2007**, *72*, 5411.

(7) F.-L. Liu, J.-R. Chen, B. Feng, X.-Q. Hu, L.-H. Ye, L.-Q. Lu, W.-J. Xiao, *Org. Biomol. Chem.* **2014**, *12*, 1057.
